# Regioselectivity and Mechanism of Synthesizing N-Substituted 2-Pyridones and 2-Substituted Pyridines *via* Metal-Free C-O and C-N Bond-Cleaving of Oxazoline[3,2-a]pyridiniums

**DOI:** 10.1038/srep41287

**Published:** 2017-01-25

**Authors:** Bo Li, Susu Xue, Yang Yang, Jia Feng, Peng Liu, Yong Zhang, Jianming Zhu, Zhijian Xu, Adrian Hall, Bo Zhao, Jiye Shi, Weiliang Zhu

**Affiliations:** 1 CAS Key Laboratory of Receptor Research, Drug Discovery and Design Center, Shanghai Institute of Materia Medica, Chinese Academy of Sciences, 555 Zuchongzhi Road, Shanghai, 201203, China; 2 State Key Laboratory of Natural and Biomimetic Drugs, Peking University, 38 Xueyuan Road, Beijing, 100191, China; 3 College of Chemistry and Environmental Science, Nanjing Normal University, 1 Wenyuan Road, Nanjing 210097, China; 4 University of Chinese Academy of Sciences, No. 19A Yuquan Road, Beijing 100049, China; 5 Nano Science and Technology Institute, University of Science and Technology of China, 166 Renai Road, Suzhou, 215123, China; 6 UCB Biopharma SPRL, Chemin du Foriest, Braine-l’Alleud, Belgium; 7 Kellogg College, University of Oxford, 60-62 Banbury Road, Oxford, OX2 6PN, United Kingdom

## Abstract

Novel intermediate oxazoline[3,2-a]pyridiniums were facilely prepared from 2-(2,2-dimethoxyethoxy)-pyridines *via* acid promoted intramolecular cyclization. Sequentially, the quaternary ammonium salts were treated with different nucleophiles for performing regioselective metal-free C-O and C-N bond-cleaving to afford prevalent heterocyclic structures of N-substituted pyridones and 2-substituted pyridines. The reaction mechanism and regioselectivity were then systematically explored by quantum chemistry calculations at B3LYP/6-31 g(d) level. The calculated free energy barrier of the reactions revealed that aniline and aliphatic amines (e.g., methylamine) prefer to attack C8 of intermediate **4a**, affording N-substituted pyridones, while phenylmethanamine, 2-phenylethan-1-amine and 3-phenylpropan-1-amine favor to attack C2 of the intermediate to form 2-substituted pyridines. With the optimized geometries of the transition states, we found that the aromatic ring of the phenyl aliphatic amines may form cation-π interaction with the pyridinium of the intermediates, which could stabilize the transition states and facilitate the formation of 2-substituted pyridines.

Pyridine and pyridone are important moieties in the structures of many drugs with a wide range of functions, such as loratadine (allergies), lansoprazole (ulcers), mirtazapine (depression), crizotinib (lung cancer), pioglitazone (diabetes), eszopiclone (insomnia), pirfenidone (ifibrosis), ciclopirox (antifungal), perampanel (epilepsy), arthpyrone (antiacetylcholinesterase) and ricinine (CNS stimulant) (Supporting Information, Figure S1). Especially, N-substituted 2-pyridone is a prevalent core structure in both natural products and chemical drugs^1^. Several synthetic approaches have been developed to construct *N*-substituted 2-pyridone derivatives^2^. In recent years, significant effort has been expended to develop synthetic strategies *via* alkyl migration or rearrangement reactions from *O*-substituted pyridines to *N*-substituted pyridones, such as C(sp^3^)-O bond cleavage^3^,^4^ and Claisen rearrangement^5^,^6^. Meanwhile, direct *N*-substitution of 2-pyridones has also been explored, despite the competing process existing between O and N alkylation^7^. Although these methods are undisputedly of importance to synthesize *N*-substituted 2-pyridones, they usually require expensive transition-metal catalysts, impractical substrates or display limited substrate scope (Fig. 1A1).

Our group previously discovered multiple *N*-substituted 3-arylisoquinolone derivatives as antitumor agents. These derivatives were prepared from *O*-substituted 3-arylisoquinolines *via* [2,3] or [3,3] rearrangement^8^. The conversion of acetal *O*-substituents to 2′-hydroxyl-1′-methoxylethyl *N*-substituents was promoted by dilute hydrochloric acid, which is distinct from the reported [2,3] rearrangements^9^. Herein we report a new approach to synthesize *N*-substituted 2-pyridones *via* an acid mediated rearrangement of 2-(2,2-dimethoxyethoxy) pyridines (Fig. 1B).

We detail the optimization of this procedure in addition to its substrate scope. In particular, aromatic and alkyl amines add to the 8-position of pyridinium derivatives **4** to give pyridone derivatives **5** (Fig. 1B), thereby providing a facile approach to prepare substituted amino compounds which have great potential, especially in drug discovery and development^10^. Alternatively, reaction with certain nucleophiles (e.g., phenylmethanamine, 2-phenylethan-1-amine and 3-phenylpropan-1-amine) was found to proceed via nucleophilic displacement at the C-2 position of the pyridinium ring to deliver 2-substituted pyridines **6** (Fig. 1B).

In addition, we isolated and characterized novel intermediates, namely, oxazoline[3,2-a]pyridinium salts (Fig. 1B). To the best of our knowledge, there are only two reports on the synthesis of oxazoline[3,2-a]quinolinium derivatives (Fig. 1A2); one is the synthesis of 1-bromomethyl-oxazoline[3,2-a]quinolinium bromides (2b) from N-allylquinolones *via* oxidation of the olefin by bromine and trapping *via* the quinolone O-atom^11^, the other is the synthesis of dihydrooxazolo[3,2-a]pyridinium methanesulfonate (2d) by the treatment of the hydroxyethyl pyridones with methanesulfonic anhydride and triethylamine in dichloromethane^12^. Furthermore, we found the nucleophilic reaction occurs primarily at the sp^3^-C (at the 8-position of compound **4**, Fig. 1B) of the novel oxazoline[3,2-a]pyridinium derivatives, which differs from the familiar pyridine quaternary ammonium salt with the nucleophilic reaction commonly occurring at the α-position of the nitrogen of pyridinium ylide^13^.

## Results and Discussion

### Optimization of Reaction Conditions

Initially, the 2-(2,2-dimethoxyethoxy) pyridine (**3a**) was prepared from 2-halogenated pyridine by treatment with sodium 2,2-dimethoxyethanolate^14^. Subsequently we employed **3a** as a test substrate to optimize the reaction conditions. After reacting with an acid, the solvent was evaporated and the residue was treated with a saturated sodium bicarbonate solution to provide *N*-substituted pyridone **5a** (Table 1). Varying first the acid (Table 1, entries 1–9) revealed trifluoroacetic acid (Table 1, entry 9) as optimal.

Reducing the equivalents of trifluoroacetic acid was detrimental (Table 1, entries 10–12), therefore 3 equivalents was considered optimal. Similarly, reducing the reaction temperature to room temperature (Table 1, entry 13) was also detrimental. Finally, solvent screening (Table 1, entries 14–17) revealed toluene at 80 °C gave a somewhat improved yield (Table 1, entry 17). Furthermore, the reaction temperature could be reduced to 50 °C with no impact on yield (Table 1, entry 18). Therefore, we chose the conditions described entry 18 for further exploration.

### Investigation of Different 2-(2,2-dimethoxyethoxy) Pyridines

**Figure 2:**
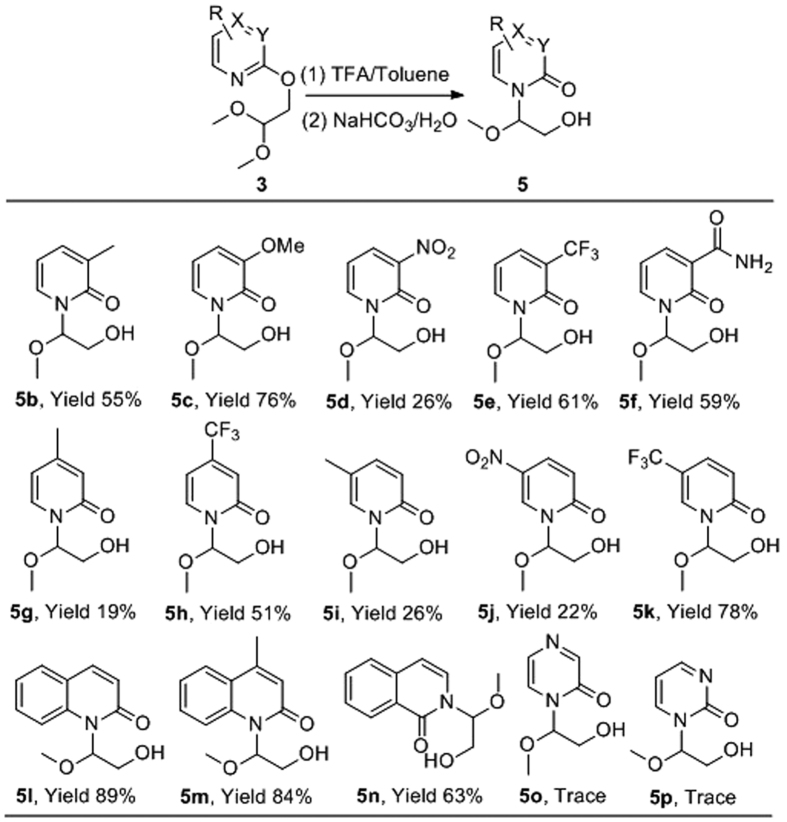
Investigation of the scope of the pyridyl substitution and related heterocycles.

Results shown in Fig. 2 demonstrate that a range of substituents, both electron donating and withdrawing, were tolerated on the pyridine moiety (**5b**–**5k**). Interestingly the 4- and 5-trifluoromethyl derivatives gave higher yields than the corresponding methyl derivatives, compared **5h** to **5g** and **5k** to **5i**. Quinolinyl and isoquinolinyl groups also worked smoothly, delivering products **5l**, **5m** and **5n** in 89%, 84% and 63% yields, respectively. However, pyrazine and pyrimidine substrates failed to yield the desired products (**5o** and **5p**) under the same conditions.

### Oxazoline[3,2-a]pyridinium Structure Elucidation

**Figure 3:**
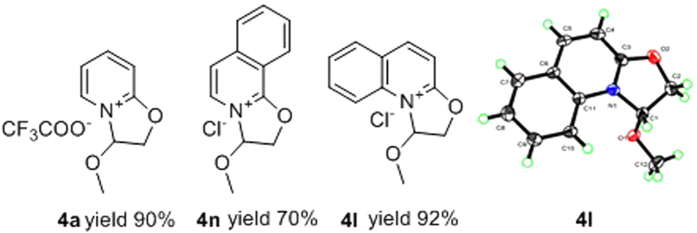
The structures of selected oxazoline[3,2-a]pyridinium derivatives.

As mentioned above, the residues from the reaction of 2-(2,2-dimethoxyethoxy) pyridines with acid were treated with saturated sodium bicarbonate solution to afford the 2′-hydroxyl-1′-methoxylethyl group *N*-substituted pyridones (**5a**-**5n**). In order to understand the reaction pathway, we purified intermediate, **4a**, and elucidated its structure by ^1^H-NMR, ^13^C-NMR and mass spectrometry (Fig. 3). During the experiment, we found most of the intermediates were oils, except for the isoquinolinyl and quinolinyl derivatives that are solids. Therefore, in order to confirm the structure of the intermediate, we prepared isoquinolinyl compound **4n** and quinolinyl compound **4l**, both as solids, in good yields by reaction of requisite precursor **3** with hydrochloric acid in acetone. Finally, we chose **4l** for crystallization and X-ray crystal structure determination (Fig. 3, details shown in Supporting Information). The results clearly show that the structure of the intermediate (**4l**) is the oxazoline[3,2-a]pyridinium species. Therefore, we conclude that the intermediates of the reaction are most likely the novel quaternary ammonium salts.

### Proposed mechanism for the formation of intermediate oxazoline[3,2-a]pyridinium

**Figure 4:**
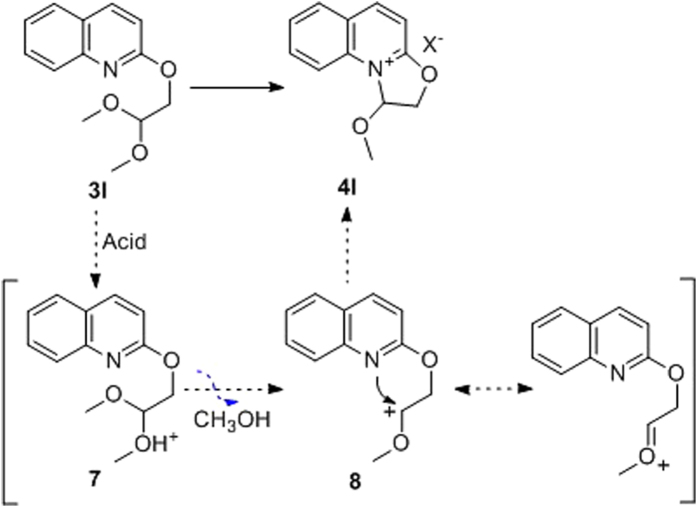
Proposed mechanism for the formation of oxazoline[3,2-a]pyridinium intermediate.

Based on the isolation of oxazoline[3,2-a]pyridinium derivatives, we propose a reaction mechanism as shown in Fig. 4. Protonation of the acetal group of **3l** followed by loss of methanol results in the formation of **8** which undergoes facile intramolecular cyclization to give **4l**. Due to the conjugative effect of aromatic ring, the quaternary ammonium salt is relatively stable.

### Investigation of nucleophile scope

**Figure 5:**
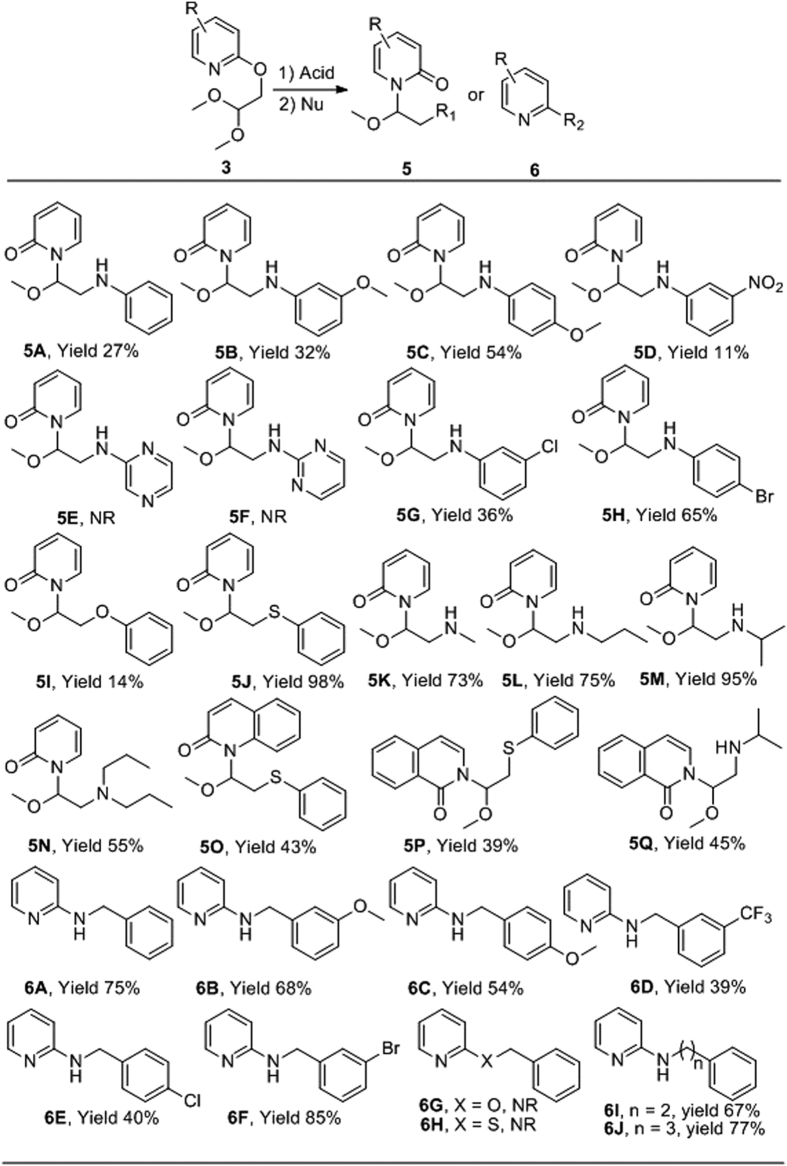
Reaction with different nucleophiles (NR = no reaction).

Encouraged by the novel structure and reactivity towards aqueous sodium bicarbonate to give 1-(2-hydroxy-1-methoxyethyl)pyridin-2(1 H)-ones (**5a**-**5n**), we were intrigued to investigate if the oxazoline[3,2-a]pyridinium derivatives would undergo reaction with other nucleophiles (Fig. 5).

It was found that the reaction was tolerant of a variety of nucleophiles. Anilines with electron donating and withdrawing groups gave good yields of the corresponding pyridones, whereas aromatic heterocyclic amines failed to yield any product (**5A**-**H**). Sodium phenolate gave a low yield (**5I**) whereas sodium thiophenolate (**5J**) gave a near quantitative yield, perhaps reflecting the matched reactivity with the soft electrophilic center. Alkyl amines (**5K**-**N**) also furnished products in good yields. Quinoline (**5O**) and iosquinoline (**5P**-**Q**) also served as good substrates, albeit with lower yields than the corresponding pyridyl derivatives. Surprisingly, phenylmethanamines were found to react at the 2-postion of the pyridine to yield **6A**-**F**. Phenylmethanol and phenylmethanethiol failed to react, thus **6G** and **6H** were not observed. Similar to the phenylmethanamines, homologation to 2-phenylethan-1-amine or 3-phenylpropan-1-amine furnished only the 2-aminopyridine derivatives **6I** and **6J** respectively.

Taken together the results suggest that the nucleophilicity has a strong impact on the course of the reaction, e. g., weak nucleophiles such as phenylmethanol and phenylmethanethiol gave no products at all (**6G**, **6H**) under these conditions. However, when the nucleophile is an aliphatic amine with aromatic substituent, such as phenylmethanamine, 2-phenylethan-1-amine and 3-phenylpropan-1-amine, formation of the N-substituted pyridones was not observed, and the reaction pathway shifted to produce 2-substituted pyridines. We postulated that the reason may involve pyridinium−π interaction (cation−π interaction) between the benzene ring and the pyridinium moiety. The proposed cation−π interaction is an intermolecular interaction which is different from the reported intramolecular cation−π interaction^15^.

### Mechanistic Investigation by Quantum Chemistry Calculations

**Figure 6:**
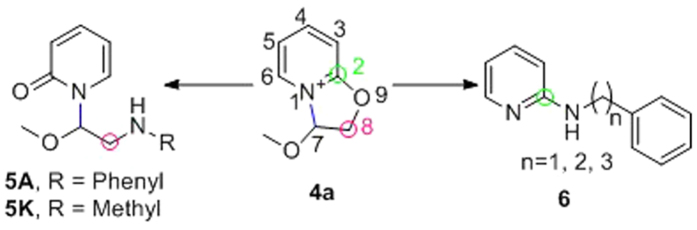
Examples of nucleophile-dependent regioselective reaction and the proposed mechanisms.

**Figure 7:**
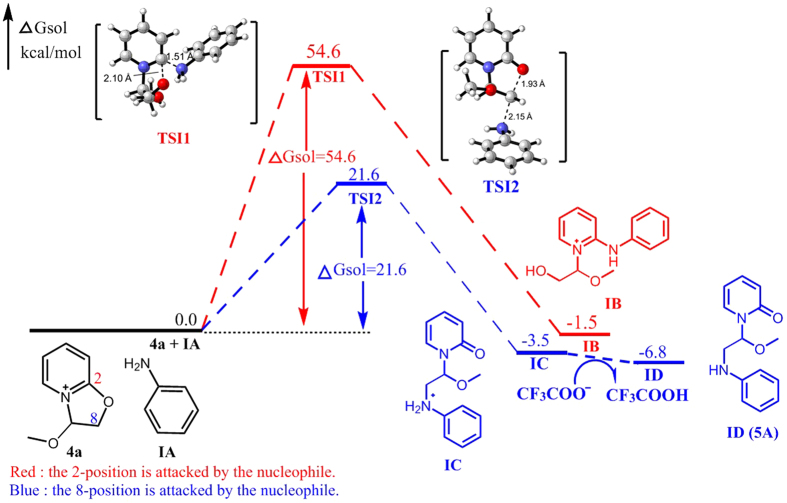
Energy profile of the nucleophilic reaction of aniline. Numbers represent relative free energies with zero-point energy correction reported in kcal/mol at the B3LYP/6-31 g(d) level in benzene.

**Figure 8:**
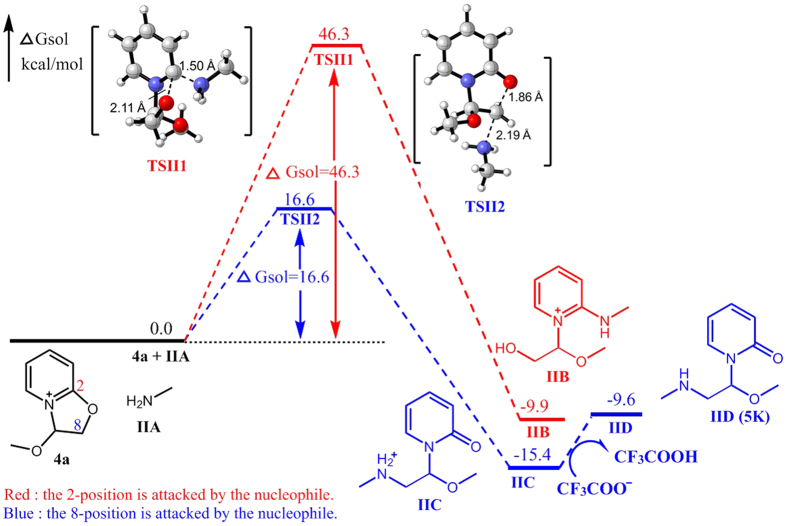
Energy profile of the nucleophilic reaction of methylamine. Numbers represent relative free energies with zero-point energy correction reported in kcal/mol at the B3LYP/6-31 g(d) level in benzene.

**Figure 9:**
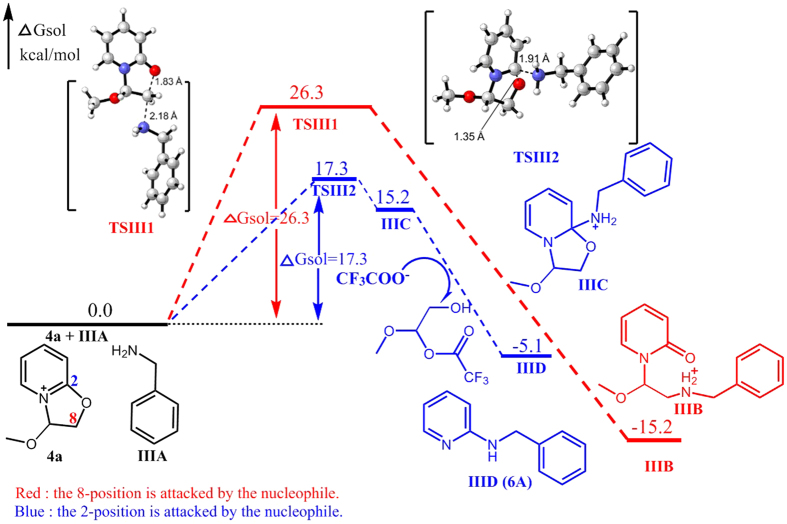
Energy profile of the nucleophilic reaction of phenylmethanamine. Numbers represent relative free energies with zero-point energy correction reported in kcal/mol at the B3LYP/6-31 g(d) level in benzene.

**Figure 10:**
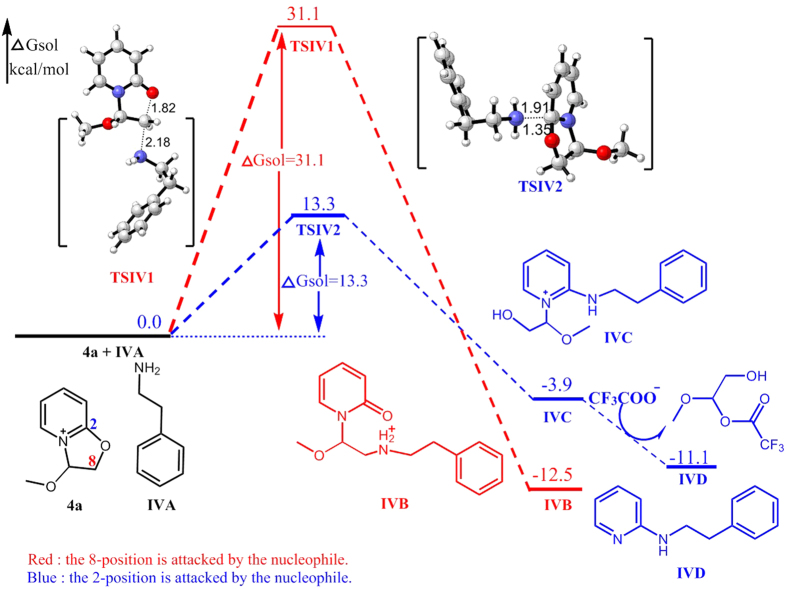
Energy profile of the nucleophilic reaction of 2-phenylethan-1-amine. Numbers represent relative free energies with zero-point energy correction reported in kcal/mol at the B3LYP/6-31 g(d) level in benzene.

**Figure 11:**
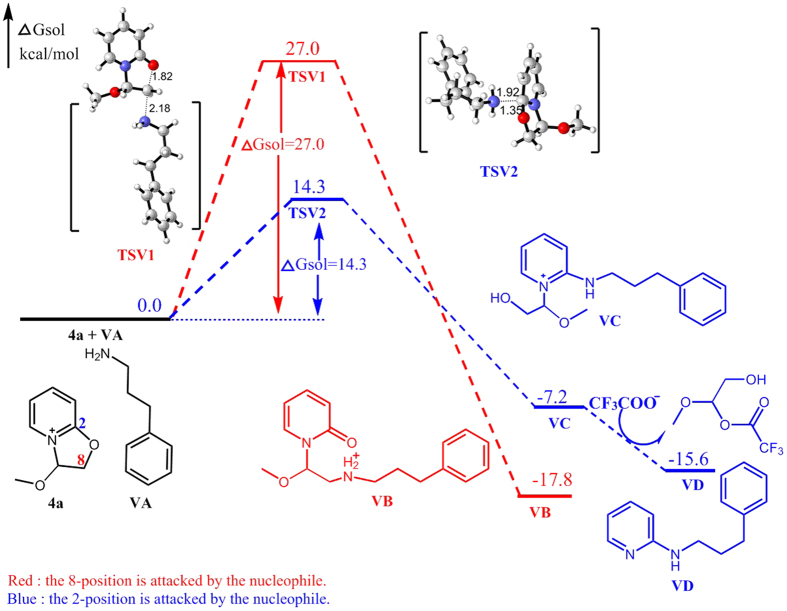
Energy profile of the nucleophilic reaction of 3-phenylpropan-1-amine. Numbers represent relative free energies with zero-point energy correction reported in kcal/mol at the B3LYP/6-31 g(d) level in benzene.

The above experimental results revealed a novel nucleophilic reaction with good regioselectivity to produce two different types of products. The reaction pathway was found to depend on the type of nucleophile used (Fig. 6). Accordingly, we proposed two possible reaction pathways and performed quantum chemistry (DFT) calculations for validation (Figs 7, 8, 9, 10 and 11).

The B3LYP/6-31 G(d) calculations revealed that the free energy barrier of aniline attacking the C8 of **4a** to form transition state TSI2 is only 21.6 kcal/mol, which is much lower than the barrier needed for the aniline to attack the C2 of **4a** (54.6 kcal/mol), indicating that the nucleophilic reaction favors the former pathway to a large extent to form **5A**. Furthermore, **IC** is more stable than **IB** by about 2.0 kcal/mol, demonstrating that **ID** (**5A**) should be the final product of the reaction between **4a** and **IA** (Fig. 7).

Calculations showed that the transition state of methylamine interacting with **4a** is similar to that of aniline. When methylamine attacks C8 of **4a**, the calculated free energy barrier to transition state TSII2 is only 16.6 kcal/mol, which is much lower than that of attacking C2 of **4a** (46.3 kcal/mol for transition state TSII1). Thus, **IID** (**5K**) should be the final product of the reaction between **4a** and methylamine (Fig. 8).

However, calculations demonstrated that the free energy barrier of attack at C2 of **4a** by phenylmethanamine to form intermediate **IIIC** via transition state TSIII2 is 17.3 kcal/mol, which is lower than that of the transition state (**TSIII1**) by 9 kcal/mol. As shown in Fig. 9, the trifluoroacetate anion then interacts with **IIIC** to afford the product **IIID** (**6A**) which has been proven by experiment. Moreover, the free energy barrier of formation TSIII1 (26.3 kcal/mol) is closed to that of TSI2 (21.6 kcal/mol, Fig. 7). But the free energy barrier of formation TSIII2 (17.3 kcal/mol) is much lower than that of TSI1 (54.6 kcal/mol, Fig. 7), and which is also lower than that of TSIII1. Therefore, the calculation results also demonstrated that phenylmethanamine nucleophile preferred to attack the C2 of **4a** but aniline favors the C8 of **4a**.

Similarly, when **6I** and **6J** attack C2 of **4a**, the calculated free energy barriers are 13.3 and 14.3 kcal/mol, respectively. In contrast, the energy of attacking C8 of **4a** by **6I** and **6J** are much higher (31.1 and 27.0 kcal/mol). As shown in Figs 10 and 11, the benzene ring and the pyridinium are almost parallel to each other, indicating the effect of pyridinium−π interaction (cation−π interaction)^15^ between them, which may contribute to reduce the free energy barrier of transition state.

Therefore, it could be concluded that the regioselectivity observed in this study could be interpreted by the calculated free energy barriers of forming various transition state structures at the first step from **4a** with different nucleophiles.

## Conclusion

We have discovered a novel approach to prepare the prevalent heterocyclic structures of N-substituted pyridones and 2-substituted pyridines from 2-(2,2-dimethoxyethoxy)-pyridines *via* regioselectively metal-free C-O and C-N bond-cleaving of novel intermediate oxazoline[3,2-a]pyridiniums. We have shown that this intermediate can undergo nucleophilic addition with anilines, alkylamines, phenol sodium and thiophenol sodium to afford N-substituted pyridones, while the phenylalkylamines gave 2-substituted pyridines. Furthermore, we proposed two different mechanisms to interpret the nucleophile-dependent regioselectivity of the reactions, which have been validated by quantum chemistry calculations at B3LYP/6-31 G(d) level. Interestingly, we found that the intermolecular cation−π interaction between the benzene ring and the pyridinium moiety should contribute to the regioselective formation of the 2-amino substituted pyridines. In particular, the sp^3^-C amination of the oxazoline[3,2-a]pyridinium moiety with aromatic amines and alkylamines to afford N-substituted pyridones may have great application in drug development. Further study to explore the potential application of the novel quaternary ammonium moiety is undergoing in our laboratory.

## Methods

### General procedure for synthesis of oxazoline[3,2-a]pyridinium (4)

2-(2,2-dimethoxyethoxy) pyridine **3** (1 mmol) was dissolved in toluene (5 ml) and the acid (3 mmol) was added to this mixture under stirring. The temperature was warmed to 50 °C. After reaction for hours, the reaction mixture was cooled to room temperature and the solvent was evaporated. The residue was washed by petroleum ether and dried under vacuum to give the oxazoline[3,2-a]pyridinium **4**.

### General procedure for preparing *N*-substituted pyridone (5)

2-(2,2-dimethoxyethoxy) pyridine **3** (1 mmol) was dissolved in toluene (5 ml) and the acid (3 mmol) was added to this mixture under stirring. The temperature was then warmed to 50 °C. After reaction for hours, the reaction mixture was cooled to room temperature and the saturated sodium bicarbonate solution was added. After stirring for another two hours, the solvent was evaporated and the residue was purified through silica gel chromatography to give the *N*-substituted pyridone **5**.

### General procedure for synthesis of *N*-substituted pyridine 5 or *O*-substituted pyridine (6)

2-(2,2-dimethoxyethoxy) pyridine **3** (1 mmol) was dissolved in toluene (5 ml) and the trifluoroacetic acid (3 mmol) was added to this mixture under stirring. The temperature was warmed to 50 °C. After reaction for hours, the reaction mixture was cooled to room temperature and the solvent was evaporated. The residue was dispersed in toluene (5 ml), and then the amine (3 mmol) was added. The reaction mixture was stirred at room temperature overnight. Then after evaporating the solvent, the residue was purified through silica gel to give the product **5** or **6**, respectively.

## Additional Information

**How to cite this article**: Li, B. *et al*. Regioselectivity and Mechanism of Synthesizing N-Substituted 2-Pyridones and 2-Substituted Pyridines via Metal-Free C-O and C-N Bond-Cleaving of Oxazoline[3,2-a]pyridiniums. *Sci. Rep.* **7**, 41287; doi: 10.1038/srep41287 (2017).

**Publisher's note:** Springer Nature remains neutral with regard to jurisdictional claims in published maps and institutional affiliations.

## Supplementary Material

Supporting Information

## Figures and Tables

**Figure 1:**
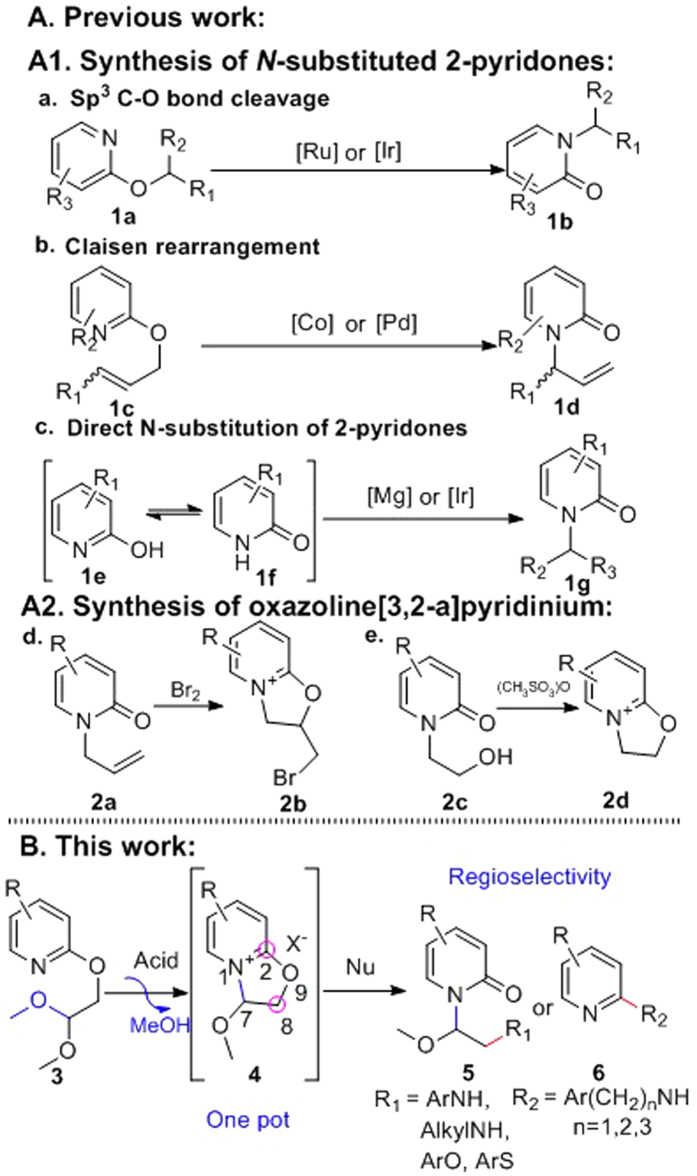
Synthesis of N-substituted 2-pyridones and oxazoline[3,2-a]pyridinium.

**Table 1:** Optimization of reaction conditions.

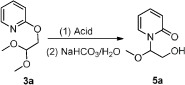					
**Entry**	**Acid**	**Eq.**	**Solvent**	**T(°C)**	**Yield%**
1	con. HCl	3.0	CH_2_Cl_2_	40	37%
2	CH_3_SO_3_H	3.0	CH_2_Cl_2_	40	46%
3	TsOH	3.0	CH_2_Cl_2_	40	33%
4	AlCl_3_	3.0	CH_2_Cl_2_	40	40%
5	ZnCl_2_	3.0	CH_2_Cl_2_	40	0
6	FeCl_3_	3.0	CH_2_Cl_2_	40	53%
7	TiCl_4_	3.0	CH_2_Cl_2_	40	32%
8	BF_3_ · Et_2_O	3.0	CH_2_Cl_2_	40	56%
9	CF_3_COOH	3.0	CH_2_Cl_2_	40	78%
10	CF_3_COOH	1.5	CH_2_Cl_2_	40	73%
11	CF_3_COOH	1	CH_2_Cl_2_	40	70%
12	CF_3_COOH	0.02	CH_2_Cl_2_	40	Trace
13	CF_3_COOH	3.0	CH_2_Cl_2_	25	Trace
14	CF_3_COOH	3.0	THF	reflux	Trace
15	CF_3_COOH	3.0	Dioxane	80	Trace
16	CF_3_COOH	3.0	Acetone	reflux	71%
17	CF_3_COOH	3.0	Toluene	80	83%
18	**CF**_**3**_**COOH**	**3**.**0**	**Toluene**	**50**	**83%**

Reaction conditions: 3a (1 mmol), acid and solvent (5 ml) at the indicated temperature for 12 h, after evaporating the solvent, the residue was treated with saturated sodium bicarbonate solution; isolated yields.
