# Supplementary material for: Regioselectivity and Mechanism of Synthesizing N-Substituted 2-Pyridones and 2-Substituted Pyridines via Metal-Free C-O and C-N Bond-Cleaving of Oxazoline[3,2-a]pyridiniums
Source: Sci Rep. 2017 Jan 25;7:41287. doi: 10.1038/srep41287 (PMC5264182; doi:10.1038/srep41287)

# Supporting Information

## Regioselectivity and Mechanism of Synthesizing N-Substituted 2-Pyridones and 2-Substituted Pyridines *via* Metal-Free C-O and C-N Bond-Cleaving of Oxazoline[3,2-a]pyridiniums

Bo Li,[+] Susu Xue,[+] Yang Yang,[+] Jia Feng,[+] Peng Liu, Yong Zhang, Jianming Zhu, Zhijian Xu, Adrian Hall, Bo Zhao,* Jiye Shi,* Weiliang Zhu,*

*corresponding authors: wlzhu@simm.ac.cn, zhaobo@njnu.edu.cn, Jiye.Shi@ucb.com

[+]these authors contributed equally to this work.

# Table of Contents

Drugs contained pyridine or 2-pyridone moieties ........................................................ 3

Supplementary Methods ........................................................................................ 4

X-ray crystal structure analysis ............................................................................ 14

Theoretical calculation ........................................................................................ 20

NMR spectra of products ...................................................................................... 73

# Drugs contained pyridine or 2-pyridone moieties

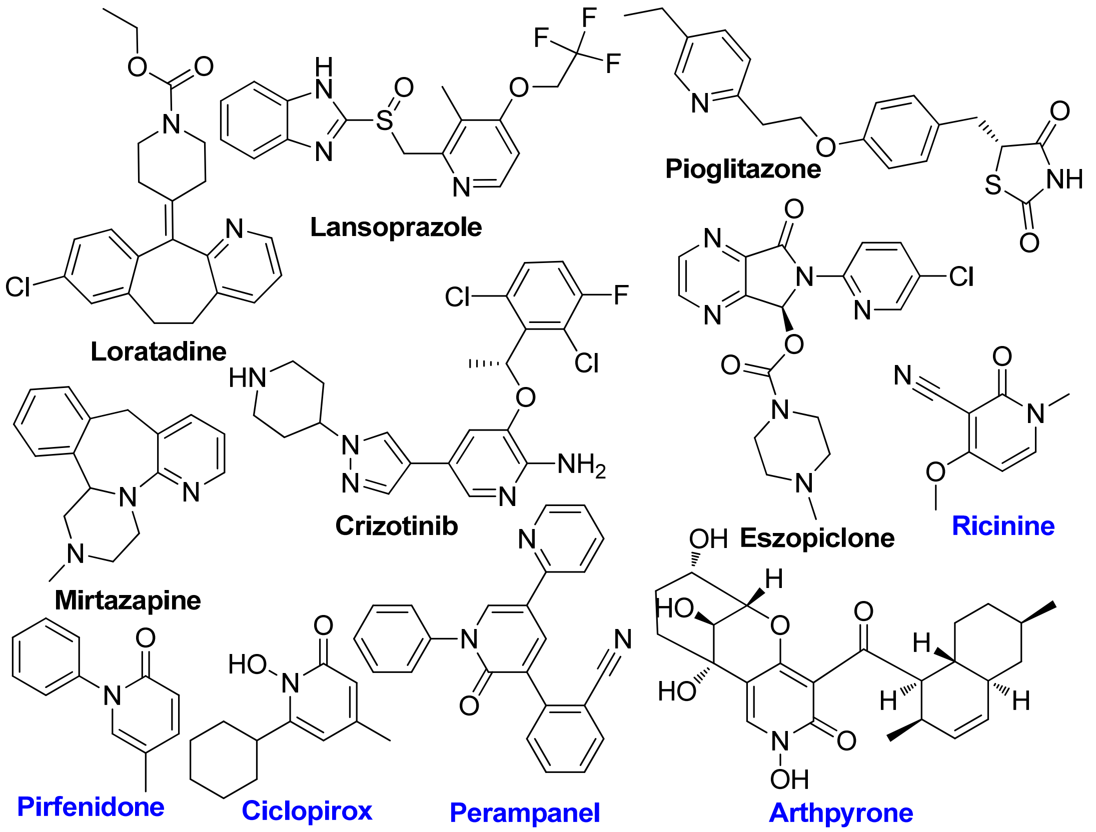


***Figure S1.*** **Examples of pyridines and 2-pyridones.**


**References of Figure S1:** (a) Cuboni, S.; Devigny, C.; Hoogeland, B.; Strasser, A.; Pomplun, S.; Hauger, B.; Höfner, G.; Wanner, K.T.; Eder, M.; Buschauer, A.; Holsboer, F.; Hausch, F. J. Med. Chem. 2014, 57, 9473. (b) Zimmermann, A.E.; Katona, B.G. Pharmacotherapy. 1997, 17, 308. (c) Graves, S.M.; Rafeyan, R.; Watts, J.; Napier, T.C. Pharmacol Ther. 2012, 136, 343. (d) Cameron, L.; Solomon, B. Drugs. 2015, 75, 1059. (e) Belfort, R.; Harrison, S.A.; Brown, K.; Darland, C.; Finch, J.; Hardies, J.; Balas, B.; Gastaldelli, A.; Tio, F.; Pulcini, J.; Berria, R.; Ma, J.Z.; Dwivedi, S.; Havranek, R.; Fincke, C.; DeFronzo, R.; Bannayan, G.A.; Schenker, S.; Cusi, K. N. Engl. J. Med. 2006, 355, 2297. (f) Brielmaier, B.D. Proc (Bayl Univ Med Cent). 2006, 19, 54. (g) King, T.E. J.; Bradford, W.Z.; Castro-Bernardini, S.; Fagan, E.A.; Glaspole, I.; Glassberg, M.K.; Gorina, E.; Hopkins, P.M.; Kardatzke, D.; Lancaster, L.; Lederer, D.J.; Nathan, S.D.; Pereira, C.A.; Sahn, S.A.; Sussman, R.; Swigris, J.J.; Noble, P.W.; ASCEND Study Group. N. Engl. J. Med. 2014, 370, 2083. (h) Carlson-Banning, K.M.; Chou, A.; Liu, Z.; Hamill, R.J.; Song, Y.; Zechiedrich, L. PLoS. One. 2013, 8, e69646. (i) Rogawski, M.A.; Hanada, T. Acta. Neurol. Scand. Suppl. 2013, 197, 19. (j) Wang, J.; Wei, X.; Qin, X.; Lin, X.; Zhou, X.; Liao, S.; Yang, B.; Liu, J.; Tu, Z.; Liu, Y. Org. Lett. 2015, 17, 656. (k) Souza, K.M.; Guilhon, G.M.; Santos, L.S.; Cascaes, M.M.; Secco, R.S.; Brasil, D.S.; Andrade, E.H.; Marinho, P.S.; Freire, L.R.; Muller, A.H. Nat. Prod. Res. 2013, 27, 364.

# Supplementary Methods

**General Information.** All starting materials were obtained from commercial suppliers and used without further purification. Anhydrous THF was distilled from sodium-benzophenon ketyl, other solvents were routinely distilled prior to use. The $^{1}H$ and $^{13}C$ spectra were taken on Bruker Avance-500 or 400, Varian-MERCURY Plus-400 or 300 NMR spectrometer operating at 400 MHz or 300 MHz for $^{1}H$, 125 MHz or 100 MHz for $^{13}C$ NMR, using TMS as the internal standard and $MeOD_4$, $CDCl_3$ or DMSO-$d_6$ as the solvent. Chemical shifts were reported in $\delta$ values (ppm) relative to that of the internal TMS. The abbreviations s = singlet, d = doublet, t = triplet, q = quadruplet, m = multiplet, and b = broad were used throughout. $^{13}C$ NMR spectra were recorded with complete proton decoupling. The ESI-MS or EI-MS was recorded on Finnigan LCQ/DECA or Thermo-DFS, respectively. The HRMS were obtained from Micromass Ultra Q-TOF (ESI) or Thermo-DFS (EI) spectrometer. The IR spectra were measured with PerkineElmer spectrum on FT-IR spectrophotometer. Silica gel F254 was used in analytical thin-layer chromatography (TLC) and silica gel was used in column chromatography, respectively, and the visualization was accomplished with UV light (254 nm).

**General procedure for preparing *N*-substituted pyridone (5)**

2-(2,2-dimethoxyethoxy) pyridine **3** (1 mmol) was dissolved in toluene (5 ml) and the acid (3 mmol) was added to this mixture under stirring. The temperature was then warmed to 50℃. After reaction for hours, the reaction mixture was cooled to room temperature and the saturated sodium bicarbonate solution was added. After stirring for another two hours, the solvent was evaporated and the residue was purified through silica gel chromatography to give the *N*-substituted pyridone **5**.

***1-(2-hydroxy-1-methoxyethyl)pyridin-2(1H)-one (5a).*** 2-(2,2-dimethoxyethoxy) pyridine **3a** give an oil of **5a** in 84% yield. $^{1}H$ NMR (400 MHz, Chloroform-*d*) $\delta$ 7.50 (dd, $J$ = 7.0, 2.1 Hz, 1H), 7.37 (ddd, $J$ = 8.9, 6.5, 2.0 Hz, 1H), 6.57 (ddd, $J$ = 9.2, 1.4, 0.7 Hz, 1H), 6.33 – 6.26 (m, 1H), 6.02 (dd, $J$ = 5.5, 4.4 Hz, 1H), 3.77 (p, $J$ = 11.7, 10.9 Hz, 2H), 3.37 (d, $J$ = 0.6 Hz, 4H). $^{13}C$ NMR (125MHz, Chloroform-*d*) $\delta$ 162.71, 139.34, 131.71, 120.23, 106.04, 85.76, 63.59, 56.75. HRMS (EI) calcd. for $C_8H_{11}NO_3$ $[M]^+$: 169.0739, found: 169.0743. IR (KBr): 1656 (C=O).

***1-(2-hydroxy-1-methoxyethyl)-3-methylpyridin-2(1H)-one (5b).***

2-(2,2-dimethoxyethoxy)-3-methylpyridine **3b** give an oil of **5b** in 55% yield. $^1$H NMR (400 MHz, Chloroform-*d*) δ 7.37 (dt, *J* = 7.2, 1.3 Hz, 1H), 7.26 (ddd, *J* = 6.7, 2.1, 1.1 Hz, 1H), 6.23 (t, *J* = 6.8 Hz, 1H), 6.08 (dd, *J* = 5.6, 4.4 Hz, 1H), 3.79 (qd, *J* = 11.7, 5.0 Hz, 2H), 3.39 (s, 3H), 2.18 (t, *J* = 0.9 Hz, 3H). $^{13}$C NMR (125MHz, Chloroform-*d*) δ 163.00, 136.66, 129.17, 128.87,105.84,86.07,63.78,56.75,16.57. HRMS (ESI) calcd for $C_9H_{13}NO_3$ [M+Na]$^+$: 206.0793, found 206.0791.

***1-(2-hydroxy-1-methoxyethyl)-3-methoxypyridin-2(1H)-one (5c).***

2-(2,2-dimethoxyethoxy)-3-methoxypyridine **3c** give an oil of **5c** in 76% yield. $^1$H NMR (400 MHz, Chloroform-*d*) δ 7.10 (dd, *J* = 7.1, 1.6 Hz, 1H), 6.63 (dd, *J* = 7.4, 1.6 Hz, 1H), 6.21 (t, *J* = 7.2 Hz, 1H), 6.07 (t, *J* = 5.1 Hz, 1H), 3.81 (s, 3H), 3.78 – 3.70 (m, 2H), 3.33 (s, 3H). $^{13}$C NMR (125 MHz, Chloroform-*d*) δ 157.99, 149.17, 122.35, 111.97, 104.97, 86.06, 63.33, 56.67, 55.40. HRMS (EI) calcd for $C_9H_{13}NO_4$ [M]$^+$: 199.0845, found 199.0848. IR (KBr): 1655 (C=O).

***1-(2-hydroxy-1-methoxyethyl)-3-nitropyridin-2(1H)-one (5d).***

2-(2,2-dimethoxyethoxy)-3-nitropyridine **3d** give an oil of **5d** in 26% yield. $^1$H NMR (400 MHz, Chloroform-*d*) δ 8.40 (dd, *J* = 7.6, 2.1 Hz, 1H), 7.92 (dd, *J* = 6.8, 2.1 Hz, 1H), 6.45 (t, *J* = 7.2 Hz, 1H), 6.11 (t, *J* = 3.9 Hz, 1H), 3.95 (dd, *J* = 12.2, 3.5 Hz, 1H), 3.81 (dd, *J* = 12.2, 4.2 Hz, 1H), 3.45 (s, 3H). $^{13}$C NMR (125MHz, Acetone) δ 153.88, 139.82, 137.59, 102.57, 87.06, 61.93, 56.22. HRMS (EI) calcd for $C_8H_{10}N_2O_5$ [M]$^+$: 214.0590, found 214.0595.

***1-(2-hydroxy-1-methoxyethyl)-3-(trifluoromethyl)pyridin-2(1H)-one (5e).***

2-(2,2-dimethoxyethoxy)-3-(trifluoromethyl)pyridine **3e** give an oil of **5e** in 61% yield. $^1$H NMR (400 MHz, Chloroform-*d*) δ 7.85 – 7.77 (m, 1H), 7.74 (dd, *J* = 7.0, 2.0 Hz, 1H), 6.38 (t, *J* = 7.0 Hz, 1H), 6.03 (dd, *J* = 5.0, 4.0 Hz, 1H), 3.87 – 3.80 (m, 1H), 3.75 (dd, *J* = 12.1, 4.9 Hz, 1H), 3.39 (s, 3H), 3.28 – 3.17 (m, 1H). $^{13}$C NMR (125 MHz, Chloroform-*d*) δ 158.47, 138.79, 138.75, 136.37, 123.21, 121.06, 119.93, 119.68, 104.15, 86.37, 86.28, 62.89, 57.06, 56.98. HRMS (ESI) calcd for $C_9H_{10}NO_3F_3$ [M+Na]$^+$: 260.0510, found 260.0509.

***1-(2-hydroxy-1-methoxyethyl)-2-oxo-1,2-dihydropyridine-3-carboxamide (5f).***

2-(2,2-dimethoxyethoxy)nicotinamide **3f** give an oil of **5f** in 59% yield. $^1$H NMR (400 MHz, DMSO-$d_6$) δ 8.99 (d, *J* = 4.2 Hz, 1H), 8.36 (dd, *J* = 7.2, 2.2 Hz, 1H), 7.88 (dd, *J* = 6.7, 2.2 Hz, 1H), 7.65 (d, *J* = 4.2 Hz, 1H), 6.58 (t, *J* = 6.9 Hz, 1H), 5.94 (t, *J* = 4.6 Hz, 1H), 5.21 (t, *J* = 6.1 Hz, 1H),

3.60 (ddd, $J$ = 6.7, 4.7, 2.3 Hz, 2H), 3.25 (s, 3H). $^{13}$C NMR (125 MHz, DMSO-$d_6$) $\delta$ 164.56, 161.69,143.78, 138.11, 106.30,86.67, 61.60, 56.60, 38.81, 38.60. HRMS (EI) calcd for $C_9H_{12}N_2O_4$ $[M]^+$: 212.0797, found 212.0799.

***1-(2-hydroxy-1-methoxyethyl)-4-methylpyridin-2(1H)-one (5g).***

2-(2,2-dimethoxyethoxy)-4-methylpyridine **3g** give an oil of **5g** in 19% yield. $^1$H NMR (400 MHz, Chloroform-$d$) $\delta$ 7.38 (d, $J$ = 7.1 Hz, 1H), 6.44 – 6.37 (m, 1H), 6.16 (dd, $J$ = 7.2, 1.8 Hz, 1H), 6.01 (t, $J$ = 5.0 Hz, 1H), 3.83 – 3.66 (m, 2H), 3.37 (s, 3H), 2.28 – 2.17 (m, 3H). $^{13}$C NMR (125MHz, Chloroform-$d$) $\delta$ 162.76, 151.26, 130.63, 118.55, 108.85, 85.54, 63.71, 56.68, 20.82. HRMS (ESI) calcd for $C_9H_{13}NO_3$ $[M+Na]^+$: 206.0793, found 206.0799.

***1-(2-hydroxy-1-methoxyethyl)-4-(trifluoromethyl)pyridin-2(1H)-one (5h).***

2-(2,2-dimethoxyethoxy)-4-(trifluoromethyl)pyridine **3h** give an oil of **5h** in 51% yield. $^1$H NMR (400 MHz, Chloroform-$d$) $\delta$ 7.71 – 7.63 (m, 1H), 6.85 (dd, $J$ = 1.9, 1.0 Hz, 1H), 6.43 (dd, $J$ = 7.3, 2.0 Hz, 1H), 5.98 (t, $J$ = 4.4 Hz, 1H), 3.88 – 3.73 (m, 2H), 3.41 (s, 3H), 2.97 – 2.89 (m, 1H). $^{13}$C NMR (125 MHz, Chloroform-$d$) $\delta$ 161.56,141.17, 140.90, 133.83, 124.84, 122.66, 120.48, 117.73, 117.70, 117.66, 101.18, 101.16, 86.29, 63.01, 57.02, HRMS (ESI) calcd for $C_9H_{10}NO_3F_3$ $[M+Na]^+$: 260.0510, found 260.0508. IR (KBr): 1678 (C=O).

***1-(2-hydroxy-1-methoxyethyl)-5-methylpyridin-2(1H)-one (5i).***

2-(2,2-dimethoxyethoxy)-5-methylpyridine **3i** give an oil of **5i** in 26% yield. $^1$H NMR (400 MHz, Chloroform-$d$) $\delta$ 7.27 – 7.20 (m, 2H), 6.57 – 6.51 (m, 1H), 6.03 (t, $J$ = 5.1 Hz, 1H), 3.77 (dd, $J$ = 5.0, 1.3 Hz, 2H), 3.37 (s, 3H), 2.12 (d, $J$ = 1.0 Hz, 3H). $^{13}$C NMR (125 MHz, Chloroform-$d$) $\delta$ 162.07, 142.15, 128.91, 119.78, 115.33, 85.73, 63.57, 56.76. HRMS (EI) calcd for $C_9H_{13}NO_3$ $[M]^+$: 183.0895, found 183.0892.

***1-(2-hydroxy-1-methoxyethyl)-5-nitropyridin-2(1H)-one (5j).***

2-(2,2-dimethoxyethoxy)-5-nitropyridine **3j** give an oil of **5j** in 22% yield. $^1$H NMR (400 MHz, Chloroform-$d$) $\delta$ 8.78 (d, $J$ = 3.1 Hz, 1H), 8.16 (dd, $J$ = 10.0, 3.1 Hz, 1H), 6.60 (d, $J$ = 10.0 Hz, 1H), 5.96 (t, $J$ = 3.7 Hz, 1H), 3.96 (dd, $J$ = 12.2, 3.4 Hz, 1H), 3.78 (dd, $J$ = 12.2, 4.1 Hz, 1H), 3.48 (s, 4H). $^{13}$C NMR (125 MHz, Chloroform-$d$) $\delta$ 161.35, 134.63, 133.02,130.92,129.42,129.25,119.21, 86.77, 62.95, 57.49. HRMS (ESI) calcd for $C_8H_{10}N_2O_5$ $[M+Na]^+$: 237.0487, found 237.0485.

***1-(2-hydroxy-1-methoxyethyl)-5-(trifluoromethyl)pyridin-2(1H)-one (5k).***

2-(2,2-dimethoxyethoxy)-5-(trifluoromethyl)pyridine **3k** give an oil of **5k** in 78% yield. $^1$H NMR (400 MHz, Chloroform-*d*) $\delta$ 7.92 – 7.88 (m, 1H), 7.50 (dd, $J$ = 9.6, 2.7 Hz, 1H), 6.67 – 6.60 (m, 1H), 5.97 (t, $J$ = 4.3 Hz, 1H), 3.85 (dd, $J$ = 12.1, 3.9 Hz, 1H), 3.76 (dd, $J$ = 12.0, 4.8 Hz, 1H), 3.42 (s, 3H), 2.93 (s, 1H). $^{13}$C NMR (125 MHz, Chloroform-*d*) $\delta$ 161.98, 135.01, 134.99, 132.02, 131.97, 131.93, 131.89, 126.05, 123.90, 121.76, 120.74, 110.28, 110.00, 109.72, 109.45, 86.36, 63.02, 62.90, 57.13. HRMS (ESI) calcd for $C_9H_{10}NO_3F_3$ [M+Na]$^+$: 260.0510, found 260.0513.

***1-(2-hydroxy-1-methoxyethyl)quinolin-2(1H)-one (5l).*** 2-(2,2-dimethoxyethoxy)quinoline **3l** give a solid of **5l** in 89% yield. $^1$H NMR (400 MHz, Chloroform-*d*) $\delta$ 8.12 (d, $J$ = 8.7 Hz, 1H), 7.69 (d, $J$ = 9.4 Hz, 1H), 7.55 (dd, $J$ = 7.8, 1.7 Hz, 1H), 7.51 – 7.46 (m, 1H), 7.26 – 7.22 (m, 1H), 6.76 – 6.71 (m, 1H), 6.68 (d, $J$ = 9.4 Hz, 1H), 4.27 (dd, $J$ = 11.8, 7.2 Hz, 1H), 4.00 (dd, $J$ = 11.8, 6.0 Hz, 1H), 3.39 (s, 3H). $^{13}$C NMR (125 MHz, Chloroform-*d*) $\delta$ 163.35, 139.89, 137.26, 129.68, 128.55, 122.22, 120.89, 120.76, 116.21, 86.77, 61.65, 56.41. HRMS (EI) calcd for $C_{12}H_{13}NO_3$ [M]$^+$: 219.0895, found 219.0898. IR (KBr): 1645 (C=O).

***1-(2-hydroxy-1-methoxyethyl)-4-methylquinolin-2(1H)-one (5m).*** 2-(2,2-dimethoxyethoxy)-4-methylquinoline **3m** give a solid of **5m** in 84% yield. $^1$H NMR (400 MHz, Chloroform-*d*) $\delta$ 8.14 (dd, $J$ = 8.7, 1.0 Hz, 1H), 7.70 (dd, $J$ = 8.0, 1.6 Hz, 1H), 7.49 (ddd, $J$ = 8.7, 7.1, 1.6 Hz, 1H), 7.28 – 7.23 (m, 1H), 6.79 – 6.70 (m, 1H), 6.56 (d, $J$ = 1.4 Hz, 1H), 4.27 (dd, $J$ = 11.7, 7.2 Hz, 1H), 3.99 (dd, $J$ = 11.8, 6.0 Hz, 1H), 3.39 (s, 3H), 2.47 (d, $J$ = 1.1 Hz, 3H). $^{13}$C NMR (125 MHz, Chloroform-*d*) $\delta$ 163.17, 147.44, 137.06, 129.49, 124.81, 122.09, 121.65, 120.14, 116.51, 115.97, 86.66, 61.71, 56.33, 18.69. HRMS (EI) calcd for $C_{13}H_{15}NO_3$ [M]$^+$: 233.1052, found 233.1056.

***2-(2-hydroxy-1-methoxyethyl)isoquinolin-1(2H)-one (5n).*** 1-(2,2-dimethoxyethoxy)isoquinoline **3n** give a solid of **5n** in 63% yield. $^1$H NMR (400 MHz, DMSO-$d_6$) $\delta$ 8.28 – 8.21 (m, 1H), 7.73 (ddd, $J$ = 8.2, 7.0, 1.3 Hz, 1H), 7.70 – 7.64 (m, 1H), 7.53 (ddd, $J$ = 8.1, 7.0, 1.4 Hz, 1H), 7.35 (d, $J$ = 7.6 Hz, 1H), 6.69 (d, $J$ = 7.6 Hz, 1H), 5.97 (t, $J$ = 5.4 Hz, 1H), 5.19 (s, 1H), 4.14 (s, 1H), 3.66 (dd, $J$ = 11.7, 5.6 Hz, 1H), 3.58 (dd, $J$ = 11.7, 5.3 Hz, 1H), 3.22 (s, 3H). $^{13}$C NMR (125 MHz, DMSO-$d_6$) $\delta$ 161.53, 136.62, 132.61, 127.21, 126.69, 126.12, 125.22, 105.45, 85.32, 85.32, 61.86, 55.94. HRMS (EI) calcd for $C_{12}H_{13}NO_3$ [M]$^+$: 219.0895, found 219.0894. IR (KBr): 1653 (C=O).

**General procedure for synthesis of oxazoline[3,2-a]pyridinium (4)**

2-(2,2-dimethoxyethoxy) pyridine **3** (1 mmol) was dissolved in toluene (5 ml) and the acid (3

mmol) was added to this mixture under stirring. The temperature was warmed to 50℃. After reaction for hours, the reaction mixture was cooled to room temperature and the solvent was evaporated. The residue was washed by petroleum ether and dried under vacuum to give the oxazoline[3,2-a]pyridinium **4**.

***3-methoxy-2,3-dihydrooxazolo[3,2-a]pyridin-4-ium 2,2,2-trifluoroacetate (4a).*** 2-(2,2-dimethoxyethoxy) pyridine **3a** give an oil of **4a** in 90% yield. $^1$H NMR (400 MHz, Chloroform-*d*) $\delta$ 7.62 (dd, $J$ = 7.0, 2.0 Hz, 1H), 7.52 (ddt, $J$ = 8.7, 6.6, 2.7 Hz, 1H), 6.75 (d, $J$ = 9.1 Hz, 1H), 6.47 (q, $J$ = 7.1 Hz, 1H), 6.08 – 5.97 (m, 1H), 3.78 (q, $J$ = 11.5, 9.8 Hz, 2H), 3.37 (s, 3H). $^{13}$C NMR (125 MHz, Chloroform-*d*) $\delta$ 159.91, 149.11, 137.12, 119.27, 110.64, 93.01, 75.74, 58.15. HRMS (EI) m/z: calcd for $C_8H_{10}O_2N^+$ 152.0706, found 152.0710. IR (KBr): 1512 (C=N+).

***3-methoxy-2,3-dihydrooxazolo[2,3-a]isoquinolin-4-ium chloride (4n).*** 1-(2,2-dimethoxyethoxy)isoquinoline **3n** give a solid of **4n** in 70% yield. $^1$H NMR (400 MHz, Chloroform-*d*) $\delta$ 8.48 – 8.41 (m, 1H), 7.70 (ddd, $J$ = 8.3, 7.0, 1.3 Hz, 1H), 7.60 – 7.49 (m, 2H), 7.28 (d, $J$ = 7.6 Hz, 1H), 6.62 (d, $J$ = 7.6 Hz, 1H), 6.26 (t, $J$ = 5.2 Hz, 1H), 3.78 (d, $J$ = 5.2 Hz, 2H), 3.43 (s, 3H). $^{13}$C NMR (125 MHz, Chloroform-*d*) $\delta$ 161.99, 136.33, 132.38, 127.52, 126.69, 125.63, 125.41, 124.76, 106.37, 84.25, 56.88, 44.19. HRMS（EI）m/z: calcd for $C_{12}H_{12}O_2N^+$ 202.0863, found 202.0868. IR (KBr): 1537 (C=N+).

***1-methoxy-1,2-dihydrooxazolo[3,2-a]quinolin-10-ium chloride (4l).*** 2-(2,2-dimethoxyethoxy)quinoline **3l** give a solid of **4l** in 92% yield. $^1$H NMR (400 MHz, Methanol-*d$_4$*) $\delta$ 9.10 (d, J = 9.3 Hz, 1H), 8.33 (d, J = 8.1 Hz, 1H), 8.26 – 8.16 (m, 2H), 7.91 (ddd, J = 8.2, 5.4, 2.6 Hz, 1H), 7.69 (d, J = 9.2 Hz, 1H), 7.16 (dd, J = 6.6, 2.5 Hz, 1H), 5.43 (dd, J = 11.5, 2.5 Hz, 1H), 5.29 (dd, J = 11.5, 6.6 Hz, 1H), 3.52 (d, J = 1.0 Hz, 3H). $^{13}$C NMR (125 MHz, Methanol-*d$_4$*) $\delta$ 162.43, 151.61, 135.22, 133.58, 130.40, 128.10, 125.05, 116.94, 108.39, 89.66, 75.23, 53.39. HRMS (EI) m/z: calcd for $C_{12}H_{12}O_2N^+$ 202.0863, found 202.0867. IR (KBr): 1536 (C=N+).

**General procedure for synthesis of *N*-substituted pyridine 5 or *O*-substituted pyridine (6)**

2-(2,2-dimethoxyethoxy) pyridine **3** (1 mmol) was dissolved in toluene (5 ml) and the trifluoroacetic acid (3 mmol) was added to this mixture under stirring. The temperature was warmed to 50℃. After reaction for hours, the reaction mixture was cooled to room temperature and the solvent was evaporated. The residue was dispersed in toluene (5 ml), and then the amine

(3 mmol) was added. The reaction mixture was stirred at room temperature overnight. Then after evaporating the solvent, the residue was purified through silica gel to give the product **5** or **6**, respectively.

***1-(1-methoxy-2-(phenylamino)ethyl)pyridin-2(1H)-one (5A).*** 2-(2,2-dimethoxyethoxy) pyridine **3a** give an oil of **5A** in 27% yield. $^1$H NMR (400 MHz, Chloroform-*d*) δ 7.50 (dd, *J* = 7.0, 2.0 Hz, 1H), 7.36 (ddd, *J* = 8.9, 6.4, 2.0 Hz, 1H), 7.19 (t, *J* = 7.7 Hz, 2H), 6.73 (dd, *J* = 11.2, 7.7 Hz, 3H), 6.60 (d, *J* = 9.2 Hz, 1H), 6.26 (t, *J* = 6.8 Hz, 1H), 6.12 (dd, *J* = 6.7, 4.0 Hz, 1H), 3.55 (dd, *J* = 13.7, 3.9 Hz, 1H), 3.36 (s, 3H), 3.35 – 3.29 (m, 1H). $^{13}$C NMR (125 MHz, Chloroform-*d*) δ 162.99, 147.34, 139.68, 131.66, 129.30, 120.82, 117.97, 113.08, 106.44, 85.36, 57.30, 47.95. HRMS (EI) calcd for $C_{14}H_{16}N_2O_2$ $[M]^+$: 244.1212, found 244.1218. IR (KBr): 1656 (C=O).

***1-(1-methoxy-2-((3-methoxyphenyl)amino)ethyl)pyridin-2(1H)-one (5B).*** 2-(2,2-dimethoxyethoxy) pyridine **3a** give an oil of **5B** in 32% yield. $^1$H NMR (400 MHz, Chloroform-*d*) δ 7.49 (dd, *J* = 7.0, 2.0 Hz, 1H), 7.37 (ddd, *J* = 8.9, 6.5, 2.0 Hz, 1H), 7.08 (t, *J* = 8.3 Hz, 1H), 6.60 (d, *J* = 9.1 Hz, 1H), 6.35 – 6.23 (m, 4H), 6.12 (dd, *J* = 6.7, 4.0 Hz, 1H), 4.21 (s, 1H), 3.79 (s, 3H), 3.54 (dd, *J* = 13.5, 4.0 Hz, 1H), 3.39 – 3.26 (m, 4H). $^{13}$C NMR (125 MHz, Chloroform-*d*) δ 162.54, 160.37, 148.29, 139.23, 131.16, 129.57, 120.34, 106.01, 105.54, 103.12, 98.33, 84.86, 56.85, 54.67, 47.51. HRMS (EI) calcd for $C_{15}H_{18}N_2O_3$ $[M]^+$: 274.1317, found 274.1329.

***1-(1-methoxy-2-((4-methoxyphenyl)amino)ethyl)pyridin-2(1H)-one (5C).*** 2-(2,2-dimethoxyethoxy) pyridine **3a** give an oil of **5C** in 53% yield. $^1$H NMR (400 MHz, Chloroform-*d*) δ 7.53 – 7.44 (m, 1H), 7.36 (ddd, *J* = 8.7, 6.6, 2.1 Hz, 1H), 6.78 (d, *J* = 8.5 Hz, 2H), 6.69 (d, *J* = 8.7 Hz, 2H), 6.59 (d, *J* = 9.2 Hz, 1H), 6.26 (t, *J* = 6.8 Hz, 1H), 6.09 (dd, *J* = 6.9, 3.8 Hz, 1H), 4.12 (s, 1H), 3.74 (s, 3H), 3.49 (dd, *J* = 13.5, 3.7 Hz, 1H), 3.34 (s, 3H), 3.26 (dd, *J* = 13.5, 6.8 Hz, 1H). $^{13}$C NMR (125 MHz, Chloroform-*d*) δ 162.48, 152.13, 140.70, 139.32, 131.28, 120.24, 114.21, 106.12, 84.88, 56.79, 55.26, 48.51. HRMS (EI) calcd for $C_{15}H_{18}N_2O_3$ $[M]^+$: 274.1317, found 274.1326.

***1-(1-methoxy-2-((3-nitrophenyl)amino)ethyl)pyridin-2(1H)-one (5D).*** 2-(2,2-dimethoxyethoxy) pyridine **3a** give an oil of **5D** in 11% yield. $^1$H NMR (400 MHz, Chloroform-*d*) δ 7.56 – 7.46 (m, 3H), 7.42 – 7.34 (m, 1H), 7.28 (d, *J* = 16.2 Hz, 1H), 7.04 – 6.97 (m, 1H), 6.61 (d, *J* = 9.1 Hz, 1H), 6.29 (t, *J* = 6.8 Hz, 1H), 6.13 – 6.06 (m, 1H), 4.74 – 4.53 (m, 1H), 3.63 – 3.53 (m, 1H), 3.42 (dd, *J*

= 13.5, 6.3 Hz, 1H), 3.38 (d, *J* = 1.1 Hz, 3H). $^{13}$C NMR (125 MHz, Chloroform-*d*) *δ* 162.59, 148.80, 147.76, 139.39, 130.80, 129.36, 120.42, 118.17, 111.94, 106.49, 106.27, 84.90, 56.96, 47.24. HRMS (EI) calcd for $C_{14}H_{15}N_3O_4$ $[M]^+$: 289.1063, found 289.1058.

***1-(2-((3-chlorophenyl)amino)-1-methoxyethyl)pyridin-2(1H)-one (5G).*** 2-(2,2-dimethoxyethoxy) pyridine **3a** give an oil of **5G** in 36% yield. $^1$H NMR (400 MHz, Chloroform-*d*) *δ* 7.51 – 7.45 (m, 1H), 7.38 (ddd, *J* = 8.9, 6.5, 2.0 Hz, 1H), 7.08 (t, *J* = 8.2 Hz, 1H), 6.69 (dd, *J* = 5.1, 2.6 Hz, 2H), 6.60 (t, *J* = 9.2 Hz, 2H), 6.28 (t, *J* = 6.8 Hz, 1H), 6.09 (dd, *J* = 6.5, 4.2 Hz, 1H), 4.26 (s, 1H), 3.57 – 3.45 (m, 1H), 3.35-3.28 (m, 4H). $^{13}$C NMR (125 MHz, Chloroform-*d*) *δ* 163.01, 148.51, 139.73, 135.00, 131.45, 130.26, 120.89, 117.81, 112.88, 111.19, 106.54, 85.31, 57.37, 47.76. HRMS (ESI) calcd for $C_{14}H_{15}ClN_2O_2$ $[M+1]^+$: 279.0895, found 279.0896.

***1-(2-((4-bromophenyl)amino)-1-methoxyethyl)pyridin-2(1H)-one (5H).*** 2-(2,2-dimethoxyethoxy) pyridine **3a** give an oil of **5H** in 65% yield. $^1$H NMR (400 MHz, Chloroform-*d*) *δ* 7.47 (dd, *J* = 6.8, 1.9 Hz, 1H), 7.42 – 7.33 (m, 1H), 7.25 (dd, *J* = 7.8, 2.3 Hz, 2H), 6.59 (dd, *J* = 8.7, 1.4 Hz, 3H), 6.27 (t, *J* = 6.7 Hz, 1H), 6.07 (dd, *J* = 6.7, 4.0 Hz, 1H), 4.20 (s, 1H), 3.58 – 3.44 (m, 1H), 3.41 – 3.20 (m, 4H). $^{13}$C NMR (125MHz, Chloroform-*d*) *δ* 162.96, 146.34, 139.75,131.97, 131.46, 120.86, 114.64, 109.59, 106.53, 85.30, 57.38, 47.90. HRMS (ESI) calcd for $C_{14}H_{15}BrN_2O_2$ $[M+1]^+$: 323.0317, found 323.0320.

***1-(1-methoxy-2-phenoxyethyl)pyridin-2(1H)-one (5I).*** 2-(2,2-dimethoxyethoxy) pyridine **3a** give an oil of **5I** in 14% yield. $^1$H NMR (400 MHz, Chloroform-*d*) *δ* 7.61 (dd, *J* = 7.0, 2.1 Hz, 1H), 7.39 (ddd, *J* = 8.9, 6.5, 2.1 Hz, 1H), 7.32 – 7.24 (m, 4H), 7.02 – 6.91 (m, 3H), 6.64 – 6.57 (m, 1H), 6.29 (dt, *J* = 5.8, 2.6 Hz, 2H), 4.26 (dd, *J* = 10.6, 3.1 Hz, 1H), 4.16 (dd, *J* = 10.6, 5.4 Hz, 1H), 3.44 (d, *J* = 0.6 Hz, 3H). $^{13}$C NMR (125 MHz, Chloroform-*d*) *δ* 162.77, 158.14, 139.80, 132.49, 129.47, 121.41, 120.80, 114.70, 106.23, 84.34, 68.33, 57.41. HRMS (EI) calcd for $C_{14}H_{15}NO_3$ $[M]^+$: 245.1052, found 245.1051.

***1-(1-methoxy-2-(phenylthio)ethyl)pyridin-2(1H)-one (5J).*** 2-(2,2-dimethoxyethoxy) pyridine **3a** give an oil of **5J** in 98% yield. $^1$H NMR (400 MHz, Chloroform-*d*) *δ* 7.49 (dd, *J* = 7.0, 2.0 Hz, 1H), 7.43 (dd, *J* = 8.0, 1.4 Hz, 2H), 7.33 (td, *J* = 6.6, 3.2 Hz, 1H), 7.31 – 7.24 (m, 2H), 7.22 – 7.14 (m, 1H), 6.57 – 6.51 (m, 1H), 6.26 – 6.20 (m, 1H), 6.08 (dd, *J* = 7.1, 3.9 Hz, 1H), 3.38 – 3.32 (m, 4H), 3.21 (dd, *J* = 14.2, 7.1 Hz, 1H). $^{13}$C NMR (125 MHz, Chloroform-*d*) *δ* 162.67, 139.61, 135.36, 131.52, 129.47, 128.95, 126.40, 120.81, 106.31, 85.59, 57.39, 38.53. HRMS (EI) calcd for

$C_{14}H_{15}NSO_2$ $[M]^+$: 261.0823, found 261.0828. IR (KBr): 1658 (C=O).

***1-(1-methoxy-2-(methylamino)ethyl)pyridin-2(1H)-one (5K).*** 2-(2,2-dimethoxyethoxy) pyridine **3a** give an oil of **5K** in 73% yield. $^1$H NMR (400 MHz, Chloroform-*d*) $\delta$ 9.57 (s, 1H), 7.99 – 7.91 (m, 1H), 7.87 (ddd, *J* = 8.8, 6.9, 1.6 Hz, 1H), 6.98 – 6.85 (m, 2H), 6.15 (dd, *J* = 7.0, 4.9 Hz, 1H), 4.04 (dd, *J* = 12.5, 4.8 Hz, 1H), 3.60 (dd, *J* = 12.5, 6.9 Hz, 1H), 3.34 (s, 3H), 3.06 (d, *J* = 4.2 Hz, 3H). $^{13}$C NMR (125 MHz, Chloroform-*d*) δ 154.26 , 142.58 , 133.52 , 112.37 , 111.16 , 92.17 , 63.76 , 58.10 , 30.07 . HRMS (ESI) calcd for $C_9H_{14}N_2O_2$ $[M+1]^+$: 183.1128, found 183.1128. IR (KBr): 1649 (C=O).

***1-(1-methoxy-2-(propylamino)ethyl)pyridin-2(1H)-one (5L).*** 2-(2,2-dimethoxyethoxy) pyridine **3a** give an oil of **5L** in 75% yield. $^1$H NMR (400 MHz, Chloroform-*d*) $\delta$ 9.75 (s, 1H), 7.95 (d, *J* = 6.8 Hz, 1H), 7.86 – 7.76 (m, 1H), 6.94 (d, *J* = 9.2 Hz, 1H), 6.87 (t, *J* = 6.9 Hz, 1H), 6.30 (dd, *J* = 8.3, 4.5 Hz, 1H), 4.20 (dd, *J* = 12.5, 4.4 Hz, 1H), 3.58 (dd, *J* = 12.5, 8.1 Hz, 1H), 3.46 – 3.32 (m, 5H), 1.76 (p, *J* = 7.4 Hz, 2H), 1.04 – 0.96 (m, 3H). $^{13}$C NMR (125 MHz, Chloroform-*d*) $\delta$ 153.86, 142.17, 133.71, 112.16, 111.33, 92.15, 63.78, 58.00, 45.50, 21.29, 11.22. HRMS (EI) calcd for $C_{11}H_{18}N_2O_2$ $[M]^+$: 210.1368, found 210.1374. IR (KBr): 1656 (C=O).

***1-(2-(isopropylamino)-1-methoxyethyl)pyridin-2(1H)-one (5M).*** 2-(2,2-dimethoxyethoxy) pyridine **3a** give an oil of **5M** in 95% yield. $^1$H NMR (400 MHz, Chloroform-*d*) $\delta$ 9.16 (s, 1H), 8.02 – 7.92 (m, 1H), 7.81 (t, *J* = 8.0 Hz, 1H), 6.97 (d, *J* = 9.3 Hz, 1H), 6.87 (t, *J* = 6.9 Hz, 1H), 6.47 (dd, *J* = 8.6, 4.4 Hz, 1H), 4.21 (dd, *J* = 12.5, 4.4 Hz, 1H), 3.88 (h, *J* = 6.6 Hz, 1H), 3.58 – 3.50 (m, 1H), 3.41 (s, 3H), 1.48 – 1.33 (m, 6H). $^{13}$C NMR (125MHz, Chloroform-*d*) $\delta$ 153.08, 142.74, 134.33, 112.57, 111.67, 92.31, 63.47, 57.93, 46.97, 21.81, 21.30. HRMS (EI) calcd for $C_{11}H_{18}N_2O_2$ $[M]^+$: 210.1368, found 210.1372.

***1-(2-(dipropylamino)-1-methoxyethyl)pyridin-2(1H)-one (5N).*** 2-(2,2-dimethoxyethoxy) pyridine **3a** give an oil of **5N** in 33% yield. $^1$H NMR (400 MHz, Chloroform-*d*) $\delta$ 7.46 (dd, *J* = 7.0, 2.1 Hz, 1H), 7.37 – 7.31 (m, 1H), 6.55 (ddt, *J* = 9.2, 1.3, 0.7 Hz, 1H), 6.25 (td, *J* = 6.6, 6.0, 1.3 Hz, 1H), 6.06 (dd, *J* = 6.8, 3.8 Hz, 1H), 3.32 (d, *J* = 0.7 Hz, 3H), 2.76 – 2.56 (m, 6H), 1.01 (td, *J* = 7.1, 0.8 Hz, 6H). $^{13}$C NMR (125 MHz, Chloroform-*d*) $\delta$ 162.76, 139.32, 132.32, 120.62, 105.90, 85.32, 56.87, 56.53, 47.96, 11.74. HRMS (EI) calcd for $C_{14}H_{24}N_2O_2$ $[M]^+$: 252.1838, found 252.1796. IR (KBr): 1658 (C=O).

***1-(1-methoxy-2-(phenylthio)ethyl)quinolin-2(1H)-one (5O).*** 2-(2,2-dimethoxyethoxy) pyridine **3l**

give an oil of **5O** in 43% yield. $^{1}$H NMR (400 MHz, Chloroform-*d*) $\delta$ 8.09 (d, $J$ = 8.6 Hz, 1H), 7.64 – 7.57 (m, 1H), 7.53 – 7.43 (m, 2H), 7.42 – 7.34 (m, 2H), 7.26 – 7.13 (m, 4H), 6.80 (t, $J$ = 6.9 Hz, 1H), 6.63 (d, $J$ = 9.4 Hz, 1H), 3.73 – 3.53 (m, 2H), 3.37 (s, 3H). $^{13}$C NMR (125MHz, Chloroform-*d*) $\delta$ 163.40, 140.08, 137.46, 134.92, 130.15, 130.05, 129.09, 128.88, 126.61, 122.56, 121.41, 121.35, 116.56, 85.95, 77.34, 77.09, 76.83, 56.76, 36.47. HRMS (ESI) calcd for $C_{18}H_{17}NO_2S$ $[M+Na]^+$: 334.0872, found 334.0869.

***2-(1-methoxy-2-(phenylthio)ethyl)isoquinolin-1(2H)-one (5P).*** 2-(2,2-dimethoxyethoxy) pyridine **3n** give an oil of **5P** in 39% yield. $^{1}$H NMR (400 MHz, Chloroform-*d*) $\delta$ 8.49 – 8.41 (m, 1H), 7.72 – 7.64 (m, 1H), 7.56 – 7.49 (m, 2H), 7.47 – 7.41 (m, 2H), 7.31 – 7.21 (m, 3H), 7.20 – 7.13 (m, 1H), 6.56 (d, $J$ = 7.6 Hz, 1H), 6.25 (dd, $J$ = 6.8, 5.1 Hz, 1H), 3.41 – 3.26 (m, 5H). $^{13}$C NMR (125 MHz, Chloroform-*d*) $\delta$ 162.41, 136.76, 135.32, 132.60, 129.90, 128.94, 127.97, 126.98, 126.52, 125.97, 125.95, 125.13, 106.81, 85.08, 77.29, 77.24, 77.03, 76.78, 57.07, 38.69. HRMS (ESI) calcd for $C_{18}H_{17}NO_2S$ $[M+Na]^+$: 334.0872, found 334.0872.

***2-(2-(isopropylamino)-1-methoxyethyl)isoquinolin-1(2H)-one (5Q).*** 2-(2,2-dimethoxyethoxy) pyridine **3n** give an oil of **5Q** in 45% yield. $^{1}$H NMR (400 MHz, Chloroform-*d*) $\delta$ 8.43 (d, $J$ = 8.0 Hz, 1H), 7.72 – 7.61 (m, 1H), 7.58 – 7.47 (m, 2H), 7.27 (d, $J$ = 7.6 Hz, 1H), 6.58 (d, $J$ = 7.6 Hz, 1H), 6.20 (dd, $J$ = 6.6, 5.3 Hz, 1H), 3.34 (s, 3H), 3.04 – 2.81 (m, 3H), 2.09 (s, 1H), 1.08 (dd, $J$ = 6.1, 1.6 Hz, 6H). HRMS (ESI) calcd for $C_{15}H_{20}N_2O_2$ $[M+1]^+$: 261.1595, found 261.1598.

***N-benzylpyridin-2-amine (6A).*** 2-(2,2-dimethoxyethoxy) pyridine **3a** give an oil of **6A** in 75% yield. $^{1}$H NMR (400 MHz, Chloroform-*d*) $\delta$ 8.17 – 8.08 (m, 1H), 7.47 – 7.29 (m, 6H), 6.66 – 6.56 (m, 1H), 6.40 (d, $J$ = 8.4 Hz, 1H), 4.88 (s, 1H), 4.53 (d, $J$ = 5.8 Hz, 2H). $^{13}$C NMR (125 MHz, Chloroform-*d*) $\delta$ 158.64, 148.22, 139.18, 137.49, 128.64, 127.41, 127.25, 113.17, 106.80, 46.33. HRMS (EI) calcd for $C_{12}H_{12}N_2$ $[M]^+$: 184.1000, found 184.0998. IR (KBr): 1599 (C=N).

***N-(3-methoxybenzyl)pyridin-2-amine (6B).*** 2-(2,2-dimethoxyethoxy) pyridine **3a** give an oil of **6B** in 68% yield. $^{1}$H NMR (400 MHz, Chloroform-*d*) $\delta$ 8.12 (s, 1H), 7.41 (s, 1H), 7.27 (s, 1H), 6.98 (s, 2H), 6.82 (s, 1H), 6.60 (s, 1H), 6.40 (s, 1H), 5.08 (s, 1H), 4.51 (s, 2H), 3.80 (s, 3H). HRMS (EI) calcd for $C_{13}H_{14}N_2O$ $[M]^+$: 214.1106, found 214.1105.

***N-(4-methoxybenzyl)pyridin-2-amine (6C).*** 2-(2,2-dimethoxyethoxy) pyridine **3a** give an oil of **6C** in 54% yield. $^{1}$H NMR (400 MHz, Chloroform-*d*) $\delta$ 8.13 (s, 1H), 7.42 (s, 1H), 7.30 (s, 3H), 6.91 (s, 2H), 6.59 (s, 1H), 6.40 (s, 1H), 4.83 (s, 1H), 4.44 (s, 2H), 3.82 (s, 3H). HRMS (EI) calcd

for $C_{13}H_{14}N_2O$ $[M]^+$: 214.1106, found 214.1107.

***N-(3-(trifluoromethyl)benzyl)pyridin-2-amine (6D).*** 2-(2,2-dimethoxyethoxy) pyridine **3a** give an oil of **6D** in 39% yield. $^1$H NMR (400 MHz, Chloroform-*d*) $\delta$ 8.16 – 8.10 (m, 1H), 7.64 (s, 1H), 7.56 (dd, $J$ = 11.8, 7.8 Hz, 2H), 7.50 – 7.40 (m, 2H), 6.68 – 6.60 (m, 1H), 6.40 (d, $J$ = 8.4 Hz, 1H), 5.01 (d, $J$ = 8.1 Hz, 1H), 4.62 (d, $J$ = 5.7 Hz, 2H). $^{13}$C NMR (125 MHz, Chloroform-*d*) $\delta$ 158.23, 148.13, 140.45, 137.63, 130.62, 129.06, 124.04, 113.57, 107.09, 45.73. HRMS (ESI) calcd for $C_{13}H_{12}N_2F_3$ $[M+H]^+$: 253.0947, found 253.0941.

***N-(4-chlorobenzyl)pyridin-2-amine (6E).*** 2-(2,2-dimethoxyethoxy) pyridine **3a** give an oil of **6E** in 40% yield. $^1$H NMR (400 MHz, Chloroform-*d*) $\delta$ 8.12 (ddd, $J$ = 5.1, 1.9, 0.9 Hz, 1H), 7.43 (ddd, $J$ = 8.4, 7.2, 1.9 Hz, 1H), 7.32 (s, 4H), 6.63 (ddd, $J$ = 7.2, 5.1, 1.0 Hz, 1H), 6.37 (dt, $J$ = 8.3, 0.9 Hz, 1H), 4.92 (s, 1H), 4.51 (d, $J$ = 5.9 Hz, 2H). $^{13}$C NMR (125 MHz, Chloroform-*d*) $\delta$ 158.37, 148.19, 137.55, 132.90, 128.74, 113.40, 106.92, 45.56. HRMS (ESI) calcd for $C_{12}H_{12}N_2Cl$ $[M+H]^+$: 219.0684, found 219.0682.

***N-(3-bromobenzyl)pyridin-2-amine (6F).*** 2-(2,2-dimethoxyethoxy) pyridine **3a** give an oil of **6F** in 85% yield. $^1$H NMR (400 MHz, Chloroform-*d*) $\delta$ 8.15 – 8.09 (m, 1H), 7.53 (s, 1H), 7.46 – 7.39 (m, 2H), 7.31 (d, $J$ = 7.8 Hz, 1H), 7.22 (t, $J$ = 7.8 Hz, 1H), 6.66 – 6.60 (m, 1H), 6.38 (d, $J$ = 8.4 Hz, 1H), 4.96 (s, 1H), 4.53 (d, $J$ = 5.8 Hz, 2H). $^{13}$C NMR (125 MHz, Chloroform-*d*) $\delta$ 158.26, 148.14, 141.78, 137.60, 130.30, 125.86, 122.74, 113.47, 107.01, 45.59. HRMS (ESI) calcd for $C_{12}H_{12}N_2Br$ $[M+H]^+$: 263.0178, found 263.0184.

***N-phenethylpyridin-2-amine (6I).*** 2-(2,2-dimethoxyethoxy) pyridine **3a** give an oil of **6I** in 67% yield. $^1$H NMR (400 MHz, Chloroform-*d*) $\delta$ 8.10 (s, 1H), 7.44 (s, 1H), 7.34 (s, 2H), 7.27 (s, 3H), 6.59 (s, 1H), 6.38 (s, 1H), 4.58 (s, 1H), 3.57 (s, 2H), 2.95 (s, 2H). $^{13}$C NMR (125 MHz, Chloroform-*d*) $\delta$ 158.53, 148.08, 139.18, 137.44, 128.79, 128.58, 126.40, 112.86, 106.80,43.30, 35.64. HRMS (EI) calcd for $C_{13}H_{14}N_2$ $[M]^+$: 198.1157, found 198.1158. IR (KBr): 1601 (C=N).

***N-(3-phenylpropyl)pyridin-2-amine (6J).*** 2-(2,2-dimethoxyethoxy) pyridine **3a** give an oil of **6J** in 77% yield. $^1$H NMR (400 MHz, Chloroform-*d*) $\delta$ 8.13 – 8.06 (m, 1H), 7.43 (ddd, $J$ = 8.8, 7.2, 1.9 Hz, 1H), 7.34 – 7.29 (m, 2H), 7.26 – 7.18 (m, 3H), 6.62 – 6.54 (m, 1H), 6.36 (d, $J$ = 8.4 Hz, 1H), 4.57 (s, 1H), 3.37 – 3.26 (m, 2H), 2.76 (t, $J$ = 7.7 Hz, 2H), 2.03 – 1.93 (m, 2H). HRMS (EI) calcd for $C_{14}H_{16}N_2$ $[M]^+$: 212.1313, found 212.1315. IR (KBr): 1601 (C=N).

# X-ray crystal structure analysis

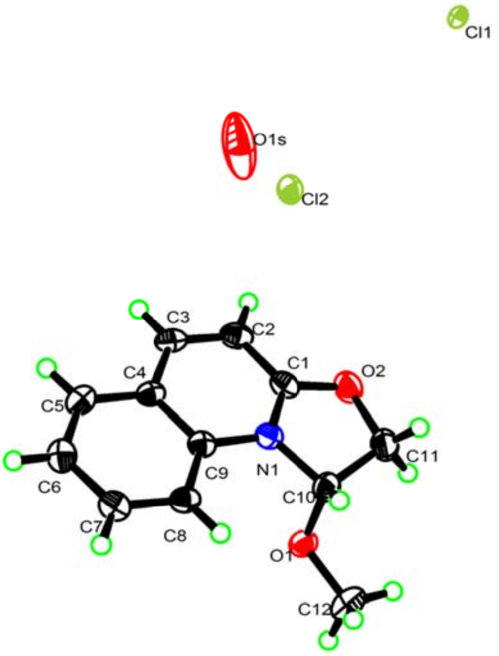


Displacement ellipsoids are drawn at the 50% probability level.

Crystal data

| | |
|---|---|
| $C_{12}H_{12}Cl_{0.79}NO_3$ | $V$ = 613.21 (2) Å$^3$ |
| $M_r$ = 246.30 | $Z$ = 2 |
| Triclinic, $P\bar{1}$ | $F$(000) = 256.9 |
| $a$ = 7.0519 (1) Å | $D_x$ = 1.334 Mg m$^{-3}$ |
| $b$ = 9.3447 (2) Å | Mo $K$ radiation, = 0.71073 Å |
| $c$ = 9.4759 (2) Å | = 0.26 mm$^{-1}$ |
| = 85.109 (1)° | $T$ = 296 K |
| = 80.694 (1)° | Plate, colourless |
| = 86.389 (1)° | 0.15 × 0.1 × 0.05 mm |

Data collection

| | |
|---|---|
| Radiation source: fine-focus sealed tube | $R_{int}$ = 0.030 |
| graphite | $_{max}$ = 27.7°, $_{min}$ = 2.2° |
| 10619 measured reflections | $h$ = -9 9 |
| 2849 independent reflections | $k$ = -12 12 |
| 2309 reflections with $I > 2\ (I)$ | $l$ = -12 12 |

Refinement

| | |
|---|---|
| Refinement on $F^2$ | Secondary atom site location: difference Fourier map |
| Least-squares matrix: full | Hydrogen site location: inferred from neighbouring sites |
| $R[F^2 > 2\ (F^2)] = 0.057$ | H atoms treated by a mixture of independent and constrained refinement |
| $wR(F^2) = 0.182$ | $w = 1/[\ ^2(F_o^2) + (0.103P)^2 + 0.6005P]$ where $P = (F_o^2 + 2F_c^2)/3$ |
| $S$ = 1.09 | $(\ /\ )_{max} < 0.001$ |
| 2849 reflections | $_{max}$ = 0.86 e Å$^{-3}$ |
| 167 parameters | $_{min}$ = -0.46 e Å$^{-3}$ |
| 0 restraints | Extinction correction: *SHELXL*, $Fc^*=kFc[1+0.001xFc^2\ ^3/\sin(2\ )]^{-1/4}$ |
| Primary atom site location: structure-invariant direct methods | Extinction coefficient: 0.060 (12) |

Special details

**Geometry.** All esds (except the esd in the dihedral angle between two l.s. planes) are estimated using the full covariance matrix. The cell esds are taken into account individually in the estimation of esds in distances, angles and torsion angles; correlations between esds in cell parameters are only used when they are defined by crystal symmetry. An approximate (isotropic) treatment of cell esds is used for estimating esds involving l.s. planes.

**Refinement.** Refinement of $F^2$ against ALL reflections. The weighted R-factor wR and goodness of fit S are based on $F^2$, conventional R-factors R are based on F, with F set to zero for negative $F^2$. The

threshold expression of $F^2 > 2sigma(F^2)$ is used only for calculating R-factors(gt) etc. and is not relevant to the choice of reflections for refinement. R-factors based on $F^2$ are statistically about twice as large as those based on F, and R- factors based on ALL data will be even larger.

Fractional atomic coordinates and isotropic or equivalent isotropic displacement parameters (Å$^2$)

| | *x* | *y* | *z* | $U_{iso}$*/$U_{eq}$ | Occ. (<1) |
|---|---|---|---|---|---|
| O1 | 0.0550 (2) | 0.83752 (19) | 0.84167 (18) | 0.0237 (4) | |
| O2 | 0.2123 (3) | 0.6134 (2) | 0.6052 (2) | 0.0294 (5) | |
| C11 | 0.2466 (4) | 0.6235 (3) | 0.7531 (3) | 0.0297 (6) | |
| H11A | 0.3725 | 0.5812 | 0.7658 | 0.036* | |
| H11B | 0.1496 | 0.5743 | 0.8212 | 0.036* | |
| C6 | 0.2776 (4) | 1.2742 (3) | 0.4693 (3) | 0.0252 (5) | |
| H6 | 0.2819 | 1.3704 | 0.4350 | 0.030* | |
| C7 | 0.2983 (3) | 1.2344 (3) | 0.6129 (3) | 0.0236 (5) | |
| H7 | 0.3179 | 1.3046 | 0.6722 | 0.028* | |
| C8 | 0.2900 (3) | 1.0925 (3) | 0.6668 (3) | 0.0211 (5) | |
| H8 | 0.3044 | 1.0666 | 0.7616 | 0.025* | |
| C9 | 0.2596 (3) | 0.9888 (3) | 0.5765 (3) | 0.0189 (5) | |
| N1 | 0.2485 (3) | 0.8446 (2) | 0.6238 (2) | 0.0205 (5) | |
| C10 | 0.2343 (4) | 0.7856 (3) | 0.7737 (3) | 0.0231 (5) | |
| H10 | 0.3402 | 0.8138 | 0.8192 | 0.028* | |
| C12 | 0.0287 (4) | 0.8092 (3) | 0.9945 (3) | 0.0330 (6) | |
| H12A | 0.0326 | 0.7073 | 1.0182 | 0.049* | |
| H12B | -0.0937 | 0.8507 | 1.0358 | 0.049* | |
| H12C | 0.1294 | 0.8509 | 1.0320 | 0.049* | |
| C1 | 0.2210 (3) | 0.7434 (3) | 0.5393 (3) | 0.0226 (5) | |
| C5 | 0.2511 (3) | 1.1719 (3) | 0.3799 (3) | 0.0231 (5) | |
| H5 | 0.2397 | 1.1991 | 0.2848 | 0.028* | |
| C4 | 0.2408 (3) | 1.0260 (3) | 0.4308 (3) | 0.0193 (5) | |
| C3 | 0.2119 (3) | 0.9151 (3) | 0.3429 (3) | 0.0221 (5) | |
| H3 | 0.1988 | 0.9394 | 0.2476 | 0.027* | |
| C2 | 0.2030 (4) | 0.7750 (3) | 0.3942 (3) | 0.0247 (5) | |

| | | | | | |
|---|---|---|---|---|---|
| H2 | 0.1857 | 0.7032 | 0.3360 | 0.030* | |
| O1S | 0.5674 (6) | 0.6720 (3) | 0.0814 (4) | 0.0795 (11) | |
| Cl1 | 0.5000 | 0.0000 | 0.0000 | 0.0137 (3) | 0.4149 (17) |
| Cl2 | 0.23947 (16) | 0.49214 (11) | 0.15464 (11) | 0.0263 (4) | 0.5851 (17) |

Atomic displacement parameters (Å$^2$)

| | $U^{11}$ | $U^{22}$ | $U^{33}$ | $U^{12}$ | $U^{13}$ | $U^{23}$ |
|---|---|---|---|---|---|---|
| O1 | 0.0241 (9) | 0.0279 (9) | 0.0192 (9) | 0.0004 (7) | -0.0040 (7) | -0.0023 (7) |
| O2 | 0.0367 (11) | 0.0211 (9) | 0.0319 (11) | 0.0015 (8) | -0.0096 (8) | -0.0046 (8) |
| C11 | 0.0375 (15) | 0.0237 (13) | 0.0292 (14) | 0.0030 (11) | -0.0105 (11) | -0.0020 (10) |
| C6 | 0.0213 (12) | 0.0242 (12) | 0.0300 (14) | -0.0035 (9) | -0.0048 (10) | 0.0012 (10) |
| C7 | 0.0204 (11) | 0.0245 (12) | 0.0271 (13) | -0.0017 (9) | -0.0058 (10) | -0.0057 (10) |
| C8 | 0.0179 (11) | 0.0273 (12) | 0.0187 (11) | 0.0000 (9) | -0.0039 (9) | -0.0050 (9) |
| C9 | 0.0130 (10) | 0.0227 (12) | 0.0212 (12) | -0.0003 (8) | -0.0027 (8) | -0.0025 (9) |
| N1 | 0.0194 (10) | 0.0223 (10) | 0.0205 (10) | 0.0009 (8) | -0.0051 (8) | -0.0041 (8) |
| C10 | 0.0232 (12) | 0.0250 (12) | 0.0217 (12) | -0.0003 (9) | -0.0063 (9) | -0.0001 (9) |
| C12 | 0.0380 (15) | 0.0411 (16) | 0.0201 (13) | -0.0090 (12) | -0.0047 (11) | 0.0023 (11) |
| C1 | 0.0189 (11) | 0.0222 (12) | 0.0271 (13) | 0.0016 (9) | -0.0035 (9) | -0.0062 (10) |
| C5 | 0.0171 (11) | 0.0312 (14) | 0.0208 (12) | -0.0032 (9) | -0.0039 (9) | 0.0018 (10) |
| C4 | 0.0136 (10) | 0.0263 (12) | 0.0182 (12) | -0.0009 (8) | -0.0018 (8) | -0.0034 (9) |
| C3 | 0.0166 (10) | 0.0329 (13) | 0.0175 (11) | -0.0002 (9) | -0.0022 (8) | -0.0063 (10) |
| C2 | 0.0212 (12) | 0.0301 (13) | 0.0245 (13) | -0.0002 (10) | -0.0047 (9) | -0.0102 (10) |
| O1S | 0.135 (3) | 0.0264 (13) | 0.098 (2) | 0.0036 (15) | -0.082 (2) | -0.0074 (14) |
| Cl1 | 0.0155 (5) | 0.0143 (5) | 0.0118 (5) | -0.0026 (3) | -0.0039 (3) | 0.0008 (3) |
| Cl2 | 0.0335 (6) | 0.0187 (5) | 0.0265 (6) | -0.0018 (4) | -0.0044 (4) | -0.0013 (4) |

Geometric parameters (Å, º)

| | | | |
|---|---|---|---|
| O1—C10 | 1.400 (3) | C9—C4 | 1.420 (3) |
| O1—C12 | 1.434 (3) | N1—C1 | 1.332 (3) |
| O2—C1 | 1.318 (3) | N1—C10 | 1.470 (3) |
| O2—C11 | 1.472 (3) | C10—H10 | 0.9800 |

| | | | |
|---|---|---|---|
| C11—C10 | 1.539 (4) | C12—H12A | 0.9600 |
| C11—H11A | 0.9700 | C12—H12B | 0.9600 |
| C11—H11B | 0.9700 | C12—H12C | 0.9600 |
| C6—C5 | 1.371 (4) | C1—C2 | 1.406 (4) |
| C6—C7 | 1.408 (4) | C5—C4 | 1.409 (3) |
| C6—H6 | 0.9300 | C5—H5 | 0.9300 |
| C7—C8 | 1.381 (4) | C4—C3 | 1.428 (3) |
| C7—H7 | 0.9300 | C3—C2 | 1.358 (4) |
| C8—C9 | 1.394 (3) | C3—H3 | 0.9300 |
| C8—H8 | 0.9300 | C2—H2 | 0.9300 |
| C9—N1 | 1.386 (3) | | |
| | | | |
| C10—O1—C12 | 113.0 (2) | C9—N1—C10 | 125.7 (2) |
| C1—O2—C11 | 107.9 (2) | O1—C10—N1 | 105.27 (18) |
| O2—C11—C10 | 105.1 (2) | O1—C10—C11 | 114.4 (2) |
| O2—C11—H11A | 110.7 | N1—C10—C11 | 100.3 (2) |
| C10—C11—H11A | 110.7 | O1—C10—H10 | 112.0 |
| O2—C11—H11B | 110.7 | N1—C10—H10 | 112.0 |
| C10—C11—H11B | 110.7 | C11—C10—H10 | 112.0 |
| H11A—C11—H11B | 108.8 | O2—C1—N1 | 113.5 (2) |
| C5—C6—C7 | 120.3 (2) | O2—C1—C2 | 124.2 (2) |
| C5—C6—H6 | 119.8 | N1—C1—C2 | 122.3 (2) |
| C7—C6—H6 | 119.8 | C6—C5—C4 | 120.6 (2) |
| C8—C7—C6 | 120.9 (2) | C6—C5—H5 | 119.7 |
| C8—C7—H7 | 119.6 | C4—C5—H5 | 119.7 |
| C6—C7—H7 | 119.6 | C5—C4—C9 | 118.0 (2) |
| C7—C8—C9 | 118.8 (2) | C5—C4—C3 | 123.0 (2) |
| C7—C8—H8 | 120.6 | C9—C4—C3 | 119.0 (2) |
| C9—C8—H8 | 120.6 | C2—C3—C4 | 122.0 (2) |
| N1—C9—C8 | 121.8 (2) | C2—C3—H3 | 119.0 |
| N1—C9—C4 | 116.8 (2) | C4—C3—H3 | 119.0 |
| C8—C9—C4 | 121.4 (2) | C3—C2—C1 | 117.0 (2) |

| | | | |
|---|---|---|---|
| C1—N1—C9 | 122.9 (2) | C3—C2—H2 | 121.5 |
| C1—N1—C10 | 110.7 (2) | C1—C2—H2 | 121.5 |
| | | | |
| C1—O2—C11—C10 | 11.7 (3) | C11—O2—C1—C2 | 177.3 (2) |
| C5—C6—C7—C8 | -0.8 (4) | C9—N1—C1—O2 | -179.2 (2) |
| C6—C7—C8—C9 | -0.3 (4) | C10—N1—C1—O2 | -8.0 (3) |
| C7—C8—C9—N1 | -179.6 (2) | C9—N1—C1—C2 | 0.7 (4) |
| C7—C8—C9—C4 | 1.1 (4) | C10—N1—C1—C2 | 171.8 (2) |
| C8—C9—N1—C1 | -179.5 (2) | C7—C6—C5—C4 | 1.1 (4) |
| C4—C9—N1—C1 | -0.2 (3) | C6—C5—C4—C9 | -0.3 (3) |
| C8—C9—N1—C10 | 10.7 (4) | C6—C5—C4—C3 | 179.5 (2) |
| C4—C9—N1—C10 | -170.0 (2) | N1—C9—C4—C5 | 179.9 (2) |
| C12—O1—C10—N1 | -170.8 (2) | C8—C9—C4—C5 | -0.8 (3) |
| C12—O1—C10—C11 | 80.1 (3) | N1—C9—C4—C3 | 0.0 (3) |
| C1—N1—C10—O1 | -104.7 (2) | C8—C9—C4—C3 | 179.3 (2) |
| C9—N1—C10—O1 | 66.1 (3) | C5—C4—C3—C2 | 179.8 (2) |
| C1—N1—C10—C11 | 14.2 (3) | C9—C4—C3—C2 | -0.4 (4) |
| C9—N1—C10—C11 | -174.9 (2) | C4—C3—C2—C1 | 0.8 (4) |
| O2—C11—C10—O1 | 97.1 (2) | O2—C1—C2—C3 | 178.9 (2) |
| O2—C11—C10—N1 | -15.0 (2) | N1—C1—C2—C3 | -1.0 (4) |
| C11—O2—C1—N1 | -2.8 (3) | | |

# Theoretical calculation

### Computational methods

Gaussian 09[1] was performed at the B3LYP[2] level of density functional theory (DFT) for geometry optimizations. A basis set of 6-31g(d) was employed for all the atoms.[3] Frequency calculations were performed for all stationary points to determine them either as local minima or transition states and to obtain the thermochemical corrections to Gibbs free energies (Table S1). Each transition structure contained one imaginary frequency, exhibiting atom displacements consistent with the anticipated reaction pathway. For each saddle point, intrinsic reaction coordinate (IRC) analysis[4] was carried out to confirm whether it connected the correct configurations of reactant and product on the potential energy surface. Solvent effects in toluene solvent ($\varepsilon$ = 2.37) were evaluated by structure optimizations and frequency calculations in the solvent utilizing the self-consistent reaction field method[5] with PCM solvation model.[6] The solvation single-point energies with Gibbs free energy corrections were used to describe the reaction energetics throughout the study.

### Calculated energies and Cartesian coordinates for the relevant intermediates and transition states

**Table S1.** Calculated energy values (energies in Hartree)

| Species | $E^{a,b}$ | $ZPE^{a,c}$ | $H_{298}{}^{a,d}$ | $G_{298}{}^{a,e}$ | Frequency[a,f] |
|---|---|---|---|---|---|
| **4a** | -515.866368 | -515.690782 | -515.680673 | -515.725444 | |
| $CF3COO^{-}$ | -526.290971 | -526.264894 | -526.257966 | -526.295676 | |
| aniline | -287.602729 | -287.486496 | -287.479990 | -287.515514 | |
| Ⅰ**A**(**C8**) | -1329.851976 | -1329.530196 | -1329.504502 | -1329.591923 | |
| **TS**Ⅰ**2** | -1329.820881 | -1329.499327 | -1329.474758 | -1329.557566 | -499.33 |
| Ⅰ**C** | -1329.863591 | -1329.540245 | -1329.515683 | -1329.59744 | |
| CF3COOH | -526.785459 | -526.746503 | -526.739314 | -526.777942 | |
| Ⅰ**D** | -803.061298 | -802.779528 | -802.762368 | -802.824871 | |
| Ⅰ**A**(**C2**) | -1329.843937 | -1329.522551 | -1329.496730 | -1329.585015 | |
| **TS**Ⅰ**1** | -1329.762588 | -1329.441875 | -1329.418024 | -1329.498008 | -339.47 |
| Ⅰ**B** | -1329.855498 | -1329.532026 | -1329.508178 | -1329.587413 | |
| benzylamine | -326.909213 | -326.762546 | -326.754452 | -326.794350 | |
| Ⅲ**A**(**C2**) | -1369.157356 | -1368.805985 | -1368.779123 | -1368.868097 | |

| | | | | | |
|---|---|---|---|---|---|
| TSⅢ2 | -1369.133358 | -1368.781728 | -1368.756422 | -1368.840461 | -184.88 |
| ⅢC | -1369.137743 | -1368.785419 | -1368.759933 | -1368.843755 | |
| ⅢD | -1369.164386 | -1368.813403 | -1368.786729 | -1368.876143 | |
| ⅢA(C8) | -1369.153799 | -1368.802717 | -1368.775640 | -1368.866151 | |
| TSⅢ1 | -1369.115105 | -1368.764555 | -1368.738696 | -1368.824309 | -510.85 |
| ⅢB | -1369.183120 | -1368.829904 | -1368.803936 | -1368.890385 | |
| methylamine | -95.855268 | -95.790859 | -95.786535 | -95.813756 | |
| ⅡA(C8) | -1138.100297 | -1137.831332 | -1137.808386 | -1137.887993 | |
| TSⅡ2 | -1138.077396 | -1137.808389 | -1137.786655 | -1137.861541 | -500.24 |
| ⅡC | -1138.130410 | -1137.859191 | -1137.837584 | -1137.912562 | |
| ⅡD | -611.3139316 | -611.084745 | -611.070618 | -611.125178 | |
| ⅡA(C2) | -1138.093821 | -1137.825107 | -1137.80196 | -1137.882874 | |
| TSⅡ1 | -1138.026911 | -1137.758131 | -1137.737451 | -1137.809049 | -342.29 |
| ⅡB | -1138.116772 | -1137.845796 | -1137.824028 | -1137.898639 | |
| phenylethylamine | -366.081801 | -366.081801 | -366.0383960 | -366.081801 | |
| ⅣA(C2) | -1408.46796 | -1408.088563 | -1408.060169 | -1408.153708 | |
| TSⅣ2 | -1408.451954 | -1408.071549 | -1408.044919 | -1408.132535 | -197.31 |
| ⅣC | -1408.478221 | -1408.097928 | -1408.070056 | -1408.159889 | |
| ⅣD | -613.3242694 | -613.079704 | -613.066116 | -613.121119 | |
| ⅣA(C8) | -1408.473095 | -1408.093167 | -1408.064931 | -1408.158008 | |
| TSⅣ1 | -1408.426296 | -1408.046943 | -1408.019861 | -1408.108489 | -510.2 |
| ⅣB | -1408.497877 | -1408.115608 | -1408.088491 | -1408.177936 | |
| benzedrine | -405.536352 | -405.332671 | -405.321835 | -405.369197 | |
| ⅤA(C2) | -1447.775633 | -1447.367420 | -1447.337584 | -1447.435089 | |
| TSⅤ2 | -1447.758119 | -1447.349171 | -1447.321178 | -1447.412261 | -195.16 |
| ⅤC | -1447.791986 | -1447.382874 | -1447.35382 | -1447.446526 | |
| ⅤD | -652.6397955 | -652.366284 | -652.351491 | -652.409612 | |
| ⅤA(C8) | -1447.783579 | -1447.374938 | -1447.34539 | -1447.440421 | |

| | | | | | |
|---|---|---|---|---|---|
| TS V 1 | -1447.739777 | -1447.33220 | -1447.303589 | -1447.397422 | -509.42 |
| V B | -1447.816195 | -1447.405414 | -1447.376839 | -1447.468853 | |

[a]With B3LYP functional; 6-31(d)for C,H,O,N,F

[b]Electronic energies

[c]Sum of electronic and zero-point energies

[d]Sum of electronic and thermal enthalpies

[e]Sum of electronic and thermal free energies

[f]The only imaginary frequency of transition state

**Cartesian coordinates**

**4a:**

1 1 (charge, spin multiplicity)

| | | | |
|---|---|---|---|
| **C** | **-1.98720131** | **-1.58124859** | **0.22291479** |
| **C** | **-0.66593972** | **-1.38890960** | **0.54957330** |
| **N** | **-0.11058803** | **-0.15912927** | **0.36849858** |
| **C** | **-0.82054027** | **0.89010659** | **-0.10916556** |
| **C** | **-2.16196744** | **0.74911563** | **-0.46539270** |
| **C** | **-2.73375783** | **-0.50184123** | **-0.29561768** |
| **O** | **-0.11306033** | **2.00408869** | **-0.17816964** |
| **C** | **1.34479038** | **0.23761312** | **0.50326311** |
| **C** | **1.19167029** | **1.76470888** | **0.42777984** |
| **H** | **-2.43891453** | **-2.55438855** | **0.37047707** |
| **H** | **-0.02512605** | **-2.15810000** | **0.96113110** |
| **H** | **-2.70912994** | **1.60301029** | **-0.84449707** |
| **H** | **-3.77687917** | **-0.64892809** | **-0.55574118** |
| **H** | **1.94892007** | **2.20632260** | **-0.21732650** |
| **H** | **1.18857472** | **2.23573519** | **1.41241621** |
| **O** | **2.10235577** | **-0.20246353** | **-0.55730902** |
| **C** | **2.61417466** | **-1.54156406** | **-0.47798665** |
| **H** | **3.40072476** | **-1.60608488** | **-1.22983700** |
| **H** | **3.03606967** | **-1.73804558** | **0.51505560** |
| **H** | **1.83788346** | **-2.27981489** | **-0.70940204** |
| **H** | **1.71025707** | **-0.12668696** | **1.46985248** |

$CF3COO^{-}$

-1 1(charge, spin multiplicity)

| | | | |
|---|---|---|---|
| **C** | **1.05487793** | **0.01011139** | **-0.01232693** |
| **O** | **1.58768587** | **1.13789736** | **-0.00496519** |
| **O** | **1.52981541** | **-1.14525671** | **-0.00572795** |

| | | | |
|---|---|---|---|
| C | -0.51691597 | 0.01299275 | -0.00388767 |
| F | -1.01006603 | -0.46490570 | 1.17602011 |
| F | -1.04129987 | -0.78064527 | -0.97916074 |
| F | -1.07838766 | 1.23668985 | -0.17654463 |

aniline

0 1(charge, spin multiplicity)

| | | | |
|---|---|---|---|
| H | 2.84182470 | -0.86100372 | 0.00045501 |
| H | 2.84180413 | 0.86101709 | 0.00042528 |
| H | -1.70495256 | 2.15011815 | 0.00006605 |
| H | 0.76126570 | 2.15462932 | -0.00004464 |
| H | 0.76123810 | -2.15464835 | -0.00004385 |
| H | -1.70495898 | -2.15011370 | 0.00007225 |
| H | -2.97101167 | 0.00001515 | 0.00010759 |
| N | 2.32023285 | 0.00000000 | -0.00018424 |
| C | -1.17211643 | 1.20207592 | 0.00003936 |
| C | 0.22072141 | 1.21041853 | -0.00002117 |
| C | 0.94336867 | -0.00001076 | -0.00006283 |
| C | 0.22071943 | -1.21042204 | -0.00001915 |
| C | -1.17213151 | -1.20206569 | 0.00004342 |
| C | -1.88503480 | 0.00000142 | 0.00006238 |

Ⅰ A(C8):

0 1 (charge, spin multiplicity)

| | | | |
|---|---|---|---|
| C | 0.61350220 | -2.06976953 | 2.55099577 |
| C | -0.03894245 | -1.87201876 | 1.35149073 |
| N | 0.68489078 | -1.95459404 | 0.20608751 |
| C | 2.01423626 | -2.19753871 | 0.18864826 |
| C | 2.71661803 | -2.41568137 | 1.37257026 |
| C | 1.99259500 | -2.35032436 | 2.55525027 |
| O | 2.52515511 | -2.19376600 | -1.03306482 |
| C | 0.15162883 | -1.91544602 | -1.20732978 |
| C | 1.47797893 | -1.74999568 | -1.95930654 |
| O | -0.46311517 | -3.12022917 | -1.51370142 |
| C | -1.90291256 | -3.08161852 | -1.55877300 |
| C | 1.66937764 | 2.22830862 | -0.92170306 |
| C | 1.45891867 | 2.03598402 | 0.45717128 |
| C | 2.38406085 | 2.51135025 | 1.38459925 |
| C | 3.53497521 | 3.18737704 | 0.96963449 |
| C | 3.74434985 | 3.38438375 | -0.39774310 |

| | | | |
|---|---|---|---|
| C | 2.82736292 | 2.91187542 | -1.33518009 |
| H | 0.05488914 | -1.99677685 | 3.47618556 |
| H | -1.09686984 | -1.61259133 | 1.24156223 |
| H | 3.78121761 | -2.60954739 | 1.34039846 |
| H | 2.50583735 | -2.50628411 | 3.49902246 |
| H | -0.51362288 | -1.04741650 | -1.27391949 |
| H | 1.66651736 | -0.69920778 | -2.19497478 |
| H | 1.52805118 | -2.38488045 | -2.84219173 |
| H | -2.22521313 | -4.12177697 | -1.62945195 |
| H | -2.32686106 | -2.61281604 | -0.66555121 |
| H | -2.23997490 | -2.53389997 | -2.44682492 |
| H | 0.55589105 | 1.52649324 | 0.78248021 |
| H | 2.19622131 | 2.35992913 | 2.44524455 |
| H | 4.25038799 | 3.55994978 | 1.69723082 |
| H | 4.62969394 | 3.91393295 | -0.74188386 |
| H | 3.00329405 | 3.06967902 | -2.39751150 |
| N | 0.77386680 | 1.69276110 | -1.85506929 |
| H | -0.16038785 | 1.51051646 | -1.48499561 |
| H | 0.73907122 | 2.20079665 | -2.73210109 |
| C | -2.55617521 | 0.28553799 | 0.18528165 |
| O | -2.83023323 | -0.74607474 | 0.83685287 |
| O | -1.57590650 | 0.52056281 | -0.56382360 |
| C | -3.60520372 | 1.42924531 | 0.32050164 |
| F | -3.86896333 | 1.70182956 | 1.61879966 |
| F | -3.20976201 | 2.58181470 | -0.25676059 |
| F | -4.77692688 | 1.07221917 | -0.25943886 |

TS Ⅰ 2:

0 1(charge, spin multiplicity)

| | | | |
|---|---|---|---|
| C | -3.77321434 | -1.51705497 | -1.34491989 |
| C | -2.45196076 | -1.19801318 | -1.16129154 |
| N | -2.12786545 | -0.07073982 | -0.46453365 |
| C | -3.05345727 | 0.82041924 | 0.02947357 |
| C | -4.42507421 | 0.49190076 | -0.13129714 |
| C | -4.76347368 | -0.66108193 | -0.80574682 |
| O | -2.57017237 | 1.87151175 | 0.55945042 |
| C | -0.72958721 | 0.28468898 | -0.07784659 |
| C | -0.70673336 | 1.78831049 | 0.05972591 |
| O | -0.39594529 | -0.25100943 | 1.16722422 |

| | | | |
|---|---|---|---|
| C | -0.07717660 | -1.64661578 | 1.16473050 |
| C | 2.25420821 | 1.88371766 | 0.64508734 |
| C | 2.34274237 | 2.90827863 | 1.59656564 |
| C | 3.17070750 | 2.75707636 | 2.70907445 |
| C | 3.91139398 | 1.58635450 | 2.88363359 |
| C | 3.82399858 | 0.56882464 | 1.92998813 |
| C | 3.00173981 | 0.71087251 | 0.81322309 |
| H | -4.04023499 | -2.40554651 | -1.90366923 |
| H | -1.61250610 | -1.75696232 | -1.56172940 |
| H | -5.16623852 | 1.17485468 | 0.26573096 |
| H | -5.81243200 | -0.90857973 | -0.94170371 |
| H | -0.09674881 | -0.10310904 | -0.88330183 |
| H | -0.91747402 | 2.41513616 | -0.79281567 |
| H | -0.42990485 | 2.22960618 | 1.00293806 |
| H | 0.41813618 | -1.84419732 | 2.11699392 |
| H | -0.98556704 | -2.26004900 | 1.10106102 |
| H | 0.58767980 | -1.89868321 | 0.33223563 |
| H | 1.77056865 | 3.82380869 | 1.46207700 |
| H | 3.23627912 | 3.55903092 | 3.43934300 |
| H | 4.55544902 | 1.47070982 | 3.75055375 |
| H | 4.40701129 | -0.34039584 | 2.04931047 |
| H | 2.95033334 | -0.06486009 | 0.05542809 |
| N | 1.36692800 | 2.01019186 | -0.46340668 |
| H | 1.37106331 | 2.95629359 | -0.84111390 |
| H | 1.58402757 | 1.33702578 | -1.22821922 |
| C | 1.49367581 | -0.94102441 | -2.77762361 |
| O | 2.04100221 | 0.15358414 | -2.49956205 |
| O | 0.50130755 | -1.50204873 | -2.26322194 |
| C | 2.20688039 | -1.73632192 | -3.91278660 |
| F | 2.63631757 | -0.93043683 | -4.90796235 |
| F | 3.29555648 | -2.38026158 | -3.42139306 |
| F | 1.41139893 | -2.67126395 | -4.47265549 |

Ⅰ C:

0 1(charge, spin multiplicity)

| | | | |
|---|---|---|---|
| C | 3.82747227 | -1.07283602 | 0.92811126 |
| C | 2.49674360 | -0.80358906 | 0.79985970 |
| N | 1.66700585 | -1.61367373 | 0.05990986 |
| C | 2.13295401 | -2.75597884 | -0.64344446 |

| | | | |
|---|---|---|---|
| C | 3.54054704 | -3.03592352 | -0.46367484 |
| C | 4.35062696 | -2.22806016 | 0.28347005 |
| O | 1.35890201 | -3.40919282 | -1.35024204 |
| C | 0.21435416 | -1.31903413 | 0.05474294 |
| C | -0.35212340 | -1.22854389 | -1.36672212 |
| O | -0.53007880 | -2.29026508 | 0.73852259 |
| C | -0.37272535 | -2.26544330 | 2.15441182 |
| C | -2.63316693 | -0.34924765 | -0.64352219 |
| C | -3.63190780 | -1.20550608 | -1.10404063 |
| C | -4.78473319 | -1.37560643 | -0.33809532 |
| C | -4.92843663 | -0.69321949 | 0.87234727 |
| C | -3.91888475 | 0.16206218 | 1.31653757 |
| C | -2.75971457 | 0.34385804 | 0.55865348 |
| H | 4.45788746 | -0.41147613 | 1.50997208 |
| H | 2.02413298 | 0.06672856 | 1.24394612 |
| H | 3.91504590 | -3.91580859 | -0.97507804 |
| H | 5.40699015 | -2.46468591 | 0.38314569 |
| H | 0.12649402 | -0.34169968 | 0.54478748 |
| H | 0.41943571 | -0.93401811 | -2.07896980 |
| H | -0.77767807 | -2.18388048 | -1.66459315 |
| H | -1.06200827 | -3.01307277 | 2.55128296 |
| H | 0.65026957 | -2.52792744 | 2.45231205 |
| H | -0.62714408 | -1.27943208 | 2.56644180 |
| H | -3.51714813 | -1.73378328 | -2.04716934 |
| H | -5.56900649 | -2.03906146 | -0.68974860 |
| H | -5.82813295 | -0.82656278 | 1.46583724 |
| H | -4.03046057 | 0.69807938 | 2.25428679 |
| H | -1.96646367 | 1.00406505 | 0.89779170 |
| N | -1.42124848 | -0.15588015 | -1.45789088 |
| H | -1.69938108 | -0.10480545 | -2.44238250 |
| H | -0.94674010 | 0.82811489 | -1.25885109 |
| C | 0.30213441 | 2.43668887 | -0.09864487 |
| O | -0.22404209 | 2.14771500 | -1.21445407 |
| O | 0.29008448 | 1.79483307 | 0.96644422 |
| C | 1.01477851 | 3.81802134 | -0.07065436 |
| F | 1.55688605 | 4.14180100 | -1.26093144 |
| F | 0.12674425 | 4.78923607 | 0.24944347 |
| F | 2.00049983 | 3.85291082 | 0.84645225 |

CF3COOH

0 1(charge, spin multiplicity)

| | | | |
|---|---|---|---|
| H | 1.28833294 | 1.06824672 | -1.79545613 |
| C | 0.99307831 | -0.46623039 | -2.84435512 |
| O | 1.25914351 | 0.84075574 | -2.74544884 |
| O | 0.81911337 | -1.23334763 | -1.93329963 |
| C | 0.93703330 | -0.87962440 | -4.33133913 |
| F | -0.01211001 | -0.17921020 | -4.97618827 |
| F | 2.11819565 | -0.63972631 | -4.92719591 |
| F | 0.65857159 | -2.18027059 | -4.43760694 |

Ⅰ D

0 1(charge, spin multiplicity)

| | | | |
|---|---|---|---|
| C | 4.43147863 | 0.98676517 | 0.49396493 |
| C | 3.09810688 | 1.03267862 | 0.77416832 |
| N | 2.17523290 | 0.27978002 | 0.09180505 |
| C | 2.54791705 | -0.60712911 | -0.95893449 |
| C | 3.97229787 | -0.65079583 | -1.22691816 |
| C | 4.87096234 | 0.11186980 | -0.53892059 |
| O | 1.69952112 | -1.25642977 | -1.57196766 |
| C | 0.74625576 | 0.35776024 | 0.50948315 |
| C | -0.20452736 | 0.73355459 | -0.64214502 |
| O | 0.30561768 | -0.83823526 | 1.08159787 |
| C | 0.84716394 | -1.12849722 | 2.36363998 |
| C | -2.66652299 | 0.62106662 | -0.16658180 |
| C | -3.86689300 | 1.35681811 | -0.21690970 |
| C | -5.09981305 | 0.71039112 | -0.21200222 |
| C | -5.17243592 | -0.68442314 | -0.16592030 |
| C | -3.98511761 | -1.41697915 | -0.11295356 |
| C | -2.74256201 | -0.78290539 | -0.10549358 |
| H | 5.12503058 | 1.60110512 | 1.05525436 |
| H | 2.69341198 | 1.67073685 | 1.55276766 |
| H | 4.27556047 | -1.32462021 | -2.02066109 |
| H | 5.92966714 | 0.05298838 | -0.77858213 |
| H | 0.71145277 | 1.16718613 | 1.25244625 |
| H | 0.30290089 | 1.47354886 | -1.27045162 |
| H | -0.38491290 | -0.14972980 | -1.25512832 |
| H | 0.34875156 | -2.03502763 | 2.71196104 |
| H | 1.92884784 | -1.31007330 | 2.31677491 |

| | | | |
|---|---|---|---|
| **H** | **0.64948741** | **-0.31163732** | **3.07323975** |
| **H** | **-3.82455762** | **2.44369394** | **-0.26485063** |
| **H** | **-6.01004059** | **1.30383088** | **-0.25421964** |
| **H** | **-6.13464241** | **-1.18837264** | **-0.16801474** |
| **H** | **-4.01950728** | **-2.50298086** | **-0.06805245** |
| **H** | **-1.83408752** | **-1.36828914** | **-0.02303836** |
| **N** | **-1.44809155** | **1.30592433** | **-0.14438445** |
| **H** | **-1.54632538** | **2.29398386** | **-0.33529260** |

Ⅰ**A**(**C2**)

0 1(charge, spin multiplicity)

| | | | |
|---|---|---|---|
| **H** | **0.80116382** | **3.58796908** | **-1.05015628** |
| **H** | **-0.36947361** | **2.94297447** | **-2.25151434** |
| **H** | **-2.11212504** | **1.16639822** | **3.66030205** |
| **C** | **-0.54034917** | **0.23662201** | **2.50483722** |
| **C** | **0.18714980** | **0.27680567** | **1.33401593** |
| **N** | **-0.08434430** | **1.26997534** | **0.44665598** |
| **C** | **-1.01685378** | **2.22334865** | **0.66598726** |
| **C** | **-1.78481882** | **2.21785472** | **1.82720303** |
| **C** | **-1.53491781** | **1.20421143** | **2.74182552** |
| **C** | **0.48772430** | **1.41830118** | **-0.93710496** |
| **C** | **0.01977713** | **2.84837622** | **-1.23918634** |
| **O** | **-1.07437243** | **3.12218028** | **-0.30828548** |
| **H** | **-0.33291081** | **-0.53997314** | **3.23091681** |
| **H** | **1.00546550** | **-0.40080106** | **1.05937859** |
| **H** | **-2.53318520** | **2.98420242** | **1.98315179** |
| **N** | **-3.17120860** | **0.59877859** | **-1.23916983** |
| **H** | **-3.71416418** | **1.17171596** | **-1.87497113** |
| **H** | **-2.24919201** | **0.39852457** | **-1.61686521** |
| **O** | **-0.11507839** | **0.50373476** | **-1.80058067** |
| **C** | **0.72454444** | **-0.59062902** | **-2.23641174** |
| **H** | **1.52601265** | **-0.21478532** | **-2.88071605** |
| **H** | **1.16119232** | **-1.12106542** | **-1.38605796** |
| **H** | **0.06966119** | **-1.24938212** | **-2.80837515** |
| **H** | **1.57905367** | **1.28774686** | **-0.86855303** |
| **C** | **3.51019196** | **-0.41455696** | **0.06089366** |
| **O** | **3.39996046** | **0.74112994** | **-0.40418974** |
| **O** | **2.63080517** | **-1.22608492** | **0.44295010** |

| | | | |
|---|---|---|---|
| C | 4.96782319 | -0.96608215 | 0.12567969 |
| F | 5.89274592 | 0.01225553 | 0.22309913 |
| F | 5.15960881 | -1.79844732 | 1.17252765 |
| F | 5.25200041 | -1.67115518 | -1.00034934 |
| C | -3.85846538 | -0.50999430 | -0.73231517 |
| C | -5.26084959 | -0.50288069 | -0.62740532 |
| C | -3.15587714 | -1.63355335 | -0.25994247 |
| C | -5.93439555 | -1.58632940 | -0.06603105 |
| H | -5.81887378 | 0.35848018 | -0.98899583 |
| C | -3.83920521 | -2.71148474 | 0.29954485 |
| H | -2.07119128 | -1.65438114 | -0.33571813 |
| C | -5.23265941 | -2.69962480 | 0.40301088 |
| H | -7.01932755 | -1.55914565 | 0.00085519 |
| H | -3.27444841 | -3.57064260 | 0.65327353 |
| H | -5.76141523 | -3.54297755 | 0.83738541 |

**TS I 1**

0 1(charge, spin multiplicity)

| | | | |
|---|---|---|---|
| H | 2.88764047 | 1.27677580 | 2.38482013 |
| H | 1.26386730 | 2.00528610 | 2.46985018 |
| H | 3.43367569 | -3.33774052 | -0.64829714 |
| C | 3.91836528 | -1.23720229 | -0.87542040 |
| C | 3.46854233 | 0.04770697 | -0.68898348 |
| N | 2.21803798 | 0.27616166 | -0.17896364 |
| C | 1.41670591 | -0.76376079 | 0.22670134 |
| C | 1.83262237 | -2.08691254 | 0.00881479 |
| C | 3.09305155 | -2.31672477 | -0.50578940 |
| C | 1.92429152 | 1.58027127 | 0.48028927 |
| C | 1.85570690 | 1.20254356 | 1.99971658 |
| O | 1.31621725 | -0.05228066 | 2.19851511 |
| H | 4.89850595 | -1.39517984 | -1.30879680 |
| H | 4.05655837 | 0.94050371 | -0.94718777 |
| H | 1.16482371 | -2.90071538 | 0.26250881 |
| N | -0.05826463 | -0.47243855 | 0.34697492 |
| H | -0.05808847 | -0.35414826 | 1.41755726 |
| H | -0.17736706 | 0.51292930 | 0.02564183 |
| O | 0.66968756 | 2.07613428 | 0.04277701 |
| C | 0.72589454 | 2.78691960 | -1.21358571 |
| H | 1.53471864 | 3.52138648 | -1.18726428 |

| | | | |
|---|---|---|---|
| **H** | **0.88942917** | **2.09179298** | **-2.04745394** |
| **H** | **-0.24343917** | **3.27544452** | **-1.32686897** |
| **H** | **2.71966756** | **2.29446049** | **0.23082792** |
| **C** | **4.73964445** | **3.52914827** | **-1.25793731** |
| **O** | **3.77443925** | **3.87154478** | **-0.53569617** |
| **O** | **5.13040667** | **2.39390127** | **-1.61033595** |
| **C** | **5.63120027** | **4.70934312** | **-1.75592038** |
| **F** | **6.60928943** | **4.97786275** | **-0.85222491** |
| **F** | **6.24416088** | **4.44215117** | **-2.93078007** |
| **F** | **4.93126499** | **5.85314425** | **-1.92918101** |
| **C** | **-1.01413619** | **-1.38760171** | **-0.27730591** |
| **C** | **-1.69960202** | **-2.29723065** | **0.52475489** |
| **C** | **-1.22622987** | **-1.31790393** | **-1.65382499** |
| **C** | **-2.62010698** | **-3.16124332** | **-0.07163891** |
| **H** | **-1.52274593** | **-2.32659055** | **1.59606424** |
| **C** | **-2.14771709** | **-2.18650989** | **-2.23783508** |
| **H** | **-0.68205422** | **-0.59786142** | **-2.25891140** |
| **C** | **-2.84265058** | **-3.10714565** | **-1.44882176** |
| **H** | **-3.16276299** | **-3.87231922** | **0.54330550** |
| **H** | **-2.32344009** | **-2.14111425** | **-3.30808216** |
| **H** | **-3.56017911** | **-3.78017992** | **-1.90818145** |

Ⅰ **B:**

0 1(charge, spin multiplicity)

| | | | |
|---|---|---|---|
| **H** | **0.27814860** | **-1.85903047** | **-0.67788792** |
| **H** | **-1.08636643** | **-0.77046889** | **-0.99316239** |
| **H** | **-0.49150830** | **5.11436756** | **-0.21361280** |
| **C** | **0.94842865** | **3.50218484** | **-0.07576316** |
| **C** | **1.09526136** | **2.16097892** | **0.17556996** |
| **N** | **0.01829498** | **1.36437425** | **0.45048280** |
| **C** | **-1.25999391** | **1.87121613** | **0.46652384** |
| **C** | **-1.43329971** | **3.25184745** | **0.22847581** |
| **C** | **-0.34323514** | **4.05271914** | **-0.04122813** |
| **C** | **0.31007200** | **-0.12188517** | **0.54633293** |
| **C** | **-0.00848060** | **-0.80378159** | **-0.79799022** |
| **O** | **0.65896728** | **-0.16713157** | **-1.84875109** |
| **H** | **1.82269979** | **4.10444421** | **-0.28948158** |
| **H** | **2.05442080** | **1.64435386** | **0.17533952** |
| **H** | **-2.43285232** | **3.66199760** | **0.28104881** |

| | | | |
|---|---|---|---|
| N | -2.28668667 | 1.02312899 | 0.73688331 |
| H | 1.62202128 | -0.42985344 | -1.78525916 |
| H | -2.02035794 | 0.13897173 | 1.16693440 |
| O | -0.41053943 | -0.72706174 | 1.58286266 |
| C | 0.16783387 | -0.56965310 | 2.88812882 |
| H | 1.19649590 | -0.94478319 | 2.89431630 |
| H | 0.15987539 | 0.48092707 | 3.20261831 |
| H | -0.44635905 | -1.16094852 | 3.56883928 |
| H | 1.38883212 | -0.17385346 | 0.72504088 |
| C | 3.84090504 | -0.54398187 | -0.54991221 |
| O | 3.20531660 | -0.97611272 | -1.53952183 |
| O | 3.47428871 | 0.18770709 | 0.39989927 |
| C | 5.32542996 | -1.00519285 | -0.44860329 |
| F | 5.76125145 | -1.65689825 | -1.54317159 |
| F | 6.15297497 | 0.04822578 | -0.25369264 |
| F | 5.49019396 | -1.84068811 | 0.60779842 |
| C | -3.67579849 | 1.31079563 | 0.62514408 |
| C | -4.52000009 | 0.95247078 | 1.68290724 |
| C | -4.21310684 | 1.87209283 | -0.54048881 |
| C | -5.89429074 | 1.16393512 | 1.57704301 |
| H | -4.09754701 | 0.51454371 | 2.58320497 |
| C | -5.58651078 | 2.09828457 | -0.62814895 |
| H | -3.56384658 | 2.10967665 | -1.37745994 |
| C | -6.43120006 | 1.74504784 | 0.42647714 |
| H | -6.54357207 | 0.88065069 | 2.40036216 |
| H | -5.99732462 | 2.53465924 | -1.53397314 |
| H | -7.50093722 | 1.91483908 | 0.34900844 |

**Benzylamine:**

0 1(charge, spin multiplicity)

| | | | |
|---|---|---|---|
| C | 2.31101965 | 0.00020538 | 0.22543504 |
| C | 1.62183201 | 1.20743466 | 0.08784178 |
| C | 0.25234148 | 1.20413218 | -0.18301121 |
| C | -0.45266589 | -0.00021777 | -0.31885261 |
| C | 0.25264621 | -1.20430494 | -0.18281324 |
| C | 1.62216908 | -1.20719326 | 0.08807842 |
| C | -1.95340822 | -0.00035728 | -0.55487055 |
| N | -2.78735064 | 0.00021369 | 0.66335092 |
| H | 3.37790666 | 0.00034016 | 0.43217155 |

| | | | |
|---|---|---|---|
| H | 2.15200300 | 2.15144015 | 0.18505099 |
| H | -0.27693640 | 2.14855555 | -0.29642763 |
| H | -0.27635866 | -2.14889755 | -0.29608962 |
| H | 2.15258564 | -2.15103443 | 0.18556393 |
| H | -2.23533381 | 0.87979539 | -1.14458692 |
| H | -2.23521099 | -0.88112789 | -1.14371916 |
| H | -2.53555936 | 0.80990568 | 1.23135516 |
| H | -2.53524743 | -0.80866669 | 1.23237961 |

ⅢA(C2):

0 1(charge, spin multiplicity)

| | | | |
|---|---|---|---|
| C | -3.81682421 | -0.04838024 | -2.25512955 |
| C | -2.94353111 | 0.24341973 | -1.22640629 |
| N | -2.87681201 | -0.61772794 | -0.17845485 |
| C | -3.59597401 | -1.76258511 | -0.11707764 |
| C | -4.50078021 | -2.09302301 | -1.12399376 |
| C | -4.60350112 | -1.21262311 | -2.19316188 |
| O | -3.32551863 | -2.48559094 | 0.95803371 |
| C | -2.12089770 | -0.42964598 | 1.12151915 |
| C | -2.17134178 | -1.87128958 | 1.62966903 |
| H | -3.87641081 | 0.62193075 | -3.10404776 |
| H | -2.25851665 | 1.10105515 | -1.20211983 |
| H | -5.07066898 | -3.01110853 | -1.05670683 |
| H | -5.29139991 | -1.44009050 | -3.00166422 |
| H | -2.34774446 | -1.92500192 | 2.70208733 |
| H | -1.27329535 | -2.41405729 | 1.31659468 |
| O | -2.81807570 | 0.41513634 | 1.97127110 |
| C | -2.40790865 | 1.79368428 | 1.95578591 |
| H | -2.59891004 | 2.26468134 | 0.98676608 |
| H | -3.00078390 | 2.28461526 | 2.72897919 |
| H | -1.34169458 | 1.88529188 | 2.18932415 |
| H | -1.11551230 | -0.07247019 | 0.87261058 |
| C | 0.11193876 | 2.05503905 | -0.44804492 |
| O | 0.41953030 | 0.89522490 | -0.07553976 |
| O | -0.90926713 | 2.47013620 | -1.03574709 |
| C | 1.12990057 | 3.14902745 | -0.00875054 |
| F | 0.97061294 | 4.32012312 | -0.65243367 |
| F | 2.41580968 | 2.76596626 | -0.19186096 |
| F | 0.97653533 | 3.39794510 | 1.32028633 |

| | | | |
|---|---|---|---|
| N | 0.80812674 | -2.11193690 | 0.10191302 |
| H | 1.00173695 | -2.69829506 | -0.71084621 |
| H | 0.70293423 | -1.15760168 | -0.25175922 |
| C | 1.98299045 | -2.14255810 | 0.99675608 |
| H | 2.03213851 | -3.13358346 | 1.46468739 |
| H | 1.78805332 | -1.42103468 | 1.79901577 |
| C | 3.31729735 | -1.82918784 | 0.33968141 |
| C | 3.58890793 | -0.53545756 | -0.13409177 |
| C | 4.28899520 | -2.82367849 | 0.17487215 |
| C | 4.80408841 | -0.25153947 | -0.75684188 |
| H | 2.84135680 | 0.24560787 | -0.01924250 |
| C | 5.50736338 | -2.54084587 | -0.44906293 |
| H | 4.09228399 | -3.82951197 | 0.54205724 |
| C | 5.76781357 | -1.25228840 | -0.91674087 |
| H | 5.00038406 | 0.75619647 | -1.11461263 |
| H | 6.25071908 | -3.32561054 | -0.56669781 |
| H | 6.71493385 | -1.02732767 | -1.40059188 |

TSIII2

0 1(charge, spin multiplicity)

| | | | |
|---|---|---|---|
| C | 3.67847172 | -3.42460444 | 0.63515280 |
| C | 4.11390928 | -2.18013909 | 0.28371056 |
| N | 3.66389488 | -1.61355982 | -0.87916999 |
| C | 2.54017553 | -2.11197863 | -1.52997242 |
| C | 2.31862118 | -3.54236408 | -1.38589079 |
| C | 2.84282852 | -4.14593946 | -0.28272695 |
| O | 2.42279599 | -1.52397896 | -2.74204706 |
| C | 4.25607354 | -0.49169565 | -1.63592789 |
| C | 3.15532204 | -0.26754907 | -2.68249765 |
| H | 4.01989263 | -3.87927406 | 1.55664159 |
| H | 4.81060784 | -1.59036496 | 0.86509962 |
| H | 1.72403042 | -4.06103395 | -2.12802654 |
| H | 2.65381982 | -5.20101748 | -0.10569152 |
| H | 3.56967005 | -0.08046940 | -3.67162533 |
| H | 2.47810556 | 0.53130849 | -2.37050267 |
| O | 5.44339034 | -0.84358521 | -2.28957066 |
| C | 6.62440278 | -0.78703629 | -1.49320789 |
| H | 6.68016277 | -1.62460242 | -0.78606267 |
| H | 7.46605269 | -0.85511865 | -2.18531693 |

| | | | |
|---|---|---|---|
| H | 6.68489050 | 0.15961539 | -0.93929516 |
| H | 4.40575225 | 0.36200200 | -0.96818114 |
| C | 2.46780451 | 1.54025324 | 0.45424974 |
| O | 1.52917958 | 1.43617147 | -0.38789671 |
| O | 3.00799911 | 0.66722602 | 1.15429168 |
| C | 3.11557360 | 2.95357552 | 0.52514967 |
| F | 3.61583412 | 3.23773411 | 1.74354116 |
| F | 2.27423917 | 3.95101093 | 0.19163657 |
| F | 4.16000341 | 3.00237708 | -0.35433815 |
| N | 1.09652435 | -1.32505584 | -0.56618854 |
| H | 1.17710709 | -1.69292326 | 0.38352994 |
| H | 1.25860998 | -0.28971498 | -0.49931757 |
| C | -0.23508680 | -1.61914566 | -1.13922729 |
| H | -0.26808005 | -2.68297141 | -1.39869695 |
| H | -0.30282563 | -1.05141566 | -2.07289329 |
| C | -1.38752982 | -1.28504229 | -0.21096090 |
| C | -1.54473168 | 0.01050749 | 0.30606876 |
| C | -2.31937930 | -2.27141263 | 0.13292799 |
| C | -2.61694423 | 0.30469110 | 1.14826563 |
| H | -0.81938121 | 0.78270596 | 0.06093259 |
| C | -3.39586004 | -1.97446876 | 0.97199739 |
| H | -2.20489473 | -3.27912845 | -0.26093055 |
| C | -3.54628035 | -0.68459364 | 1.48185855 |
| H | -2.72720090 | 1.31048014 | 1.54464452 |
| H | -4.11173152 | -2.75103675 | 1.22815785 |
| H | -4.38097790 | -0.45050902 | 2.13716616 |

ⅢC

0 1(charge, spin multiplicity)

| | | | |
|---|---|---|---|
| C | 1.07268463 | -3.49652798 | 1.84060650 |
| C | 1.75236340 | -2.34681092 | 1.58977595 |
| N | 1.54016393 | -1.65065826 | 0.42523878 |
| C | 0.46931914 | -1.96538270 | -0.46732660 |
| C | 0.02651752 | -3.38146391 | -0.36556033 |
| C | 0.27008112 | -4.05892760 | 0.78201971 |
| O | 0.86951473 | -1.57097068 | -1.73072455 |
| C | 2.49318615 | -0.73566425 | -0.18673601 |
| C | 1.85515027 | -0.52141438 | -1.58222155 |
| H | 1.22058438 | -4.02707149 | 2.77268688 |

| | | | |
|---|---|---|---|
| **H** | **2.50280634** | **-1.93025173** | **2.25145559** |
| **H** | **-0.47772147** | **-3.81909934** | **-1.21964078** |
| **H** | **-0.09170786** | **-5.07850444** | **0.88701559** |
| **H** | **2.58964097** | **-0.64999802** | **-2.37884157** |
| **H** | **1.36644385** | **0.45240461** | **-1.67149549** |
| **O** | **3.75133883** | **-1.37345219** | **-0.21307724** |
| **C** | **4.83873580** | **-0.47056784** | **-0.38327255** |
| **H** | **4.82939097** | **0.31662493** | **0.38383895** |
| **H** | **5.75087805** | **-1.06236876** | **-0.28565457** |
| **H** | **4.82698419** | **0.00373241** | **-1.37476197** |
| **H** | **2.55665364** | **0.20605253** | **0.37211622** |
| **C** | **0.31077871** | **2.18994666** | **-0.00818371** |
| **O** | **0.27857686** | **1.27608432** | **0.86860193** |
| **O** | **-0.20631321** | **2.22175945** | **-1.13662381** |
| **C** | **1.22006993** | **3.38408559** | **0.39589216** |
| **F** | **1.08140722** | **4.44903867** | **-0.41209283** |
| **F** | **2.52564370** | **2.99624650** | **0.32901881** |
| **F** | **0.99439081** | **3.79845650** | **1.65959265** |
| **N** | **-0.77171720** | **-1.01122299** | **-0.05884230** |
| **H** | **-1.21109139** | **-1.45282974** | **0.75406120** |
| **H** | **-0.39507369** | **-0.05655719** | **0.28374390** |
| **C** | **-1.80281073** | **-0.80989642** | **-1.13657572** |
| **H** | **-1.92774475** | **-1.76390629** | **-1.65507161** |
| **H** | **-1.37779047** | **-0.07947771** | **-1.82443484** |
| **C** | **-3.11317297** | **-0.33120304** | **-0.55758132** |
| **C** | **-3.35400097** | **1.03890686** | **-0.38403560** |
| **C** | **-4.09495104** | **-1.25508332** | **-0.17447200** |
| **C** | **-4.56098247** | **1.47274619** | **0.16578810** |
| **H** | **-2.59144652** | **1.75318956** | **-0.68328344** |
| **C** | **-5.30074543** | **-0.81949259** | **0.37673930** |
| **H** | **-3.91921699** | **-2.31967182** | **-0.31764192** |
| **C** | **-5.53468062** | **0.54662344** | **0.54771310** |
| **H** | **-4.74198920** | **2.53644291** | **0.29365611** |
| **H** | **-6.05752388** | **-1.54410146** | **0.66471508** |
| **H** | **-6.47437140** | **0.88827478** | **0.97326638** |

IIID

0 1(charge, spin multiplicity)

| | | | |
|---|---|---|---|
| **C** | **-2.30569036** | **3.97386139** | **-0.16766590** |

| | | | |
|---|---|---|---|
| C | -1.17749921 | 3.25654342 | 0.21961674 |
| N | -0.98108673 | 1.95895986 | -0.04030991 |
| C | -1.94202842 | 1.29017533 | -0.71702854 |
| C | -3.13151454 | 1.92730219 | -1.14228815 |
| C | -3.29833056 | 3.27599961 | -0.86443724 |
| O | 0.42361555 | -1.25192159 | 0.79124697 |
| C | 1.97499176 | 0.66048379 | 0.82104830 |
| C | 1.09444219 | -0.30827518 | 1.61091359 |
| H | -2.40730700 | 5.02681454 | 0.07320244 |
| H | -0.37913950 | 3.74601489 | 0.77669120 |
| H | -3.90425789 | 1.37182745 | -1.66039537 |
| H | -4.20671555 | 3.78135322 | -1.18301641 |
| H | 0.32304575 | 0.29611699 | 2.08974166 |
| H | 1.69715849 | -0.79553254 | 2.39026335 |
| O | 2.19846774 | 1.77904633 | 1.57282180 |
| C | 2.80347114 | 2.86890732 | 0.86754946 |
| H | 2.19946834 | 3.14212257 | -0.00653255 |
| H | 2.83535833 | 3.70413072 | 1.56861635 |
| H | 3.82035850 | 2.61527831 | 0.55107819 |
| C | 3.53031377 | -0.93029454 | -0.24991193 |
| O | 3.34907708 | 0.08706031 | 0.56237329 |
| O | 2.69560682 | -1.66620170 | -0.73841093 |
| C | 5.04048626 | -1.11460028 | -0.53322568 |
| F | 5.23923381 | -2.13928553 | -1.36507894 |
| F | 5.70236503 | -1.35261414 | 0.61206082 |
| F | 5.54760503 | -0.00084353 | -1.09162815 |
| N | -1.65932931 | -0.02656348 | -0.98375619 |
| H | -0.88643770 | -0.40579142 | -0.43703722 |
| H | 1.09378667 | -1.68016617 | 0.22201881 |
| C | -2.62531877 | -0.98081000 | -1.49875780 |
| H | -2.05026768 | -1.86180865 | -1.80869408 |
| H | -3.08151887 | -0.58268406 | -2.41328077 |
| C | -3.72154126 | -1.41112669 | -0.52828080 |
| C | -5.05254756 | -1.49881043 | -0.95008905 |
| C | -3.40667931 | -1.76383172 | 0.79122660 |
| C | -6.05362564 | -1.92973029 | -0.07561626 |
| H | -5.31004133 | -1.22818111 | -1.97249652 |
| C | -4.40384184 | -2.19242148 | 1.66711596 |
| H | -2.37522639 | -1.70171918 | 1.12996282 |

| | | | |
|---|---|---|---|
| C | -5.73121895 | -2.27660053 | 1.23675374 |
| H | -7.08335747 | -1.98779980 | -0.41897043 |
| H | -4.14526765 | -2.46291786 | 2.68773628 |
| H | -6.50746610 | -2.60891614 | 1.92095820 |
| H | 1.55055477 | 0.87527402 | -0.16535416 |

ⅢA(C8)

0 1(charge, spin multiplicity)

| | | | |
|---|---|---|---|
| C | 3.29488922 | -3.02840518 | -0.58403576 |
| C | 2.51459840 | -2.02652534 | -0.04725436 |
| N | 3.01480357 | -0.76469743 | -0.03208370 |
| C | 4.23597543 | -0.44060906 | -0.51524209 |
| C | 5.06672751 | -1.42243086 | -1.05653178 |
| C | 4.57677506 | -2.72043611 | -1.08044365 |
| O | 4.50530436 | 0.84886393 | -0.41418280 |
| C | 2.38669976 | 0.42086998 | 0.61384749 |
| C | 3.28386766 | 1.53297589 | 0.04840965 |
| H | 2.91098911 | -4.04062062 | -0.62153546 |
| H | 1.49210426 | -2.14361309 | 0.33638434 |
| H | 6.04215064 | -1.15174774 | -1.44045349 |
| H | 5.19272930 | -3.51001336 | -1.49958008 |
| H | 3.57811637 | 2.25655821 | 0.80527346 |
| H | 2.83235604 | 2.03303421 | -0.80793499 |
| O | 2.51839355 | 0.23242088 | 1.99349399 |
| C | 1.46982403 | 0.84392207 | 2.76137794 |
| H | 1.72884632 | 0.66854271 | 3.80692360 |
| H | 1.40411765 | 1.91773393 | 2.56134395 |
| H | 0.50178337 | 0.38381147 | 2.53427018 |
| H | 1.34467298 | 0.52438941 | 0.28868949 |
| C | -0.38380420 | 2.25907733 | -0.13598011 |
| O | -0.53001350 | 1.01485268 | -0.27020071 |
| O | 0.62175054 | 2.91398290 | 0.20020471 |
| C | -1.64164361 | 3.10179958 | -0.51421395 |
| F | -1.67394046 | 4.29670238 | 0.10948621 |
| F | -2.80385042 | 2.47087866 | -0.21519628 |
| F | -1.65915284 | 3.34636463 | -1.84909335 |
| N | -0.60724971 | -1.86714585 | 0.63801907 |
| H | -0.95683044 | -2.02172334 | 1.58453958 |
| H | -0.65757919 | -0.85669861 | 0.47664451 |

| | | | |
|---|---|---|---|
| C | -1.51610811 | -2.53455033 | -0.31617181 |
| H | -1.37968850 | -3.61913487 | -0.22441565 |
| H | -1.17510705 | -2.25837573 | -1.32189653 |
| C | -2.99183232 | -2.19997968 | -0.16222118 |
| C | -3.43242550 | -0.86926679 | -0.24488356 |
| C | -3.93482520 | -3.20775053 | 0.07212158 |
| C | -4.78444354 | -0.56249544 | -0.09537715 |
| H | -2.71490950 | -0.07055377 | -0.41567900 |
| C | -5.29091478 | -2.90188768 | 0.21849225 |
| H | -3.60570413 | -4.24328122 | 0.13978212 |
| C | -5.71916498 | -1.57655423 | 0.13549631 |
| H | -5.10574809 | 0.47382407 | -0.16045372 |
| H | -6.00877380 | -3.69808000 | 0.40004138 |
| H | -6.77255754 | -1.33414435 | 0.25087901 |

TSIII1

0 1(charge, spin multiplicity)

| | | | |
|---|---|---|---|
| C | 5.54245180 | -0.43060493 | -3.32706294 |
| C | 4.46431754 | -0.26248385 | -2.50016515 |
| N | 3.21348556 | -0.58571297 | -2.93676991 |
| C | 2.96365373 | -1.09504252 | -4.18519143 |
| C | 4.06282072 | -1.26898903 | -5.06640504 |
| C | 5.32564396 | -0.93607814 | -4.63330519 |
| O | 1.74463585 | -1.37974066 | -4.42537874 |
| C | 1.95261537 | -0.30404741 | -2.16351527 |
| C | 0.85119815 | -1.10159335 | -2.85146037 |
| H | 6.53618036 | -0.17968478 | -2.97740318 |
| H | 4.53568506 | 0.11576761 | -1.48765082 |
| H | 3.86807582 | -1.66562263 | -6.05522730 |
| H | 6.17195957 | -1.06940542 | -5.30042734 |
| H | 0.05994307 | -0.55937819 | -3.34078680 |
| H | 0.74424718 | -2.15962161 | -2.67196402 |
| O | 1.61491867 | 1.02755533 | -2.20678120 |
| C | 2.18007563 | 1.89597533 | -1.21082488 |
| H | 3.09525255 | 2.36772534 | -1.58859779 |
| H | 1.40657870 | 2.63436282 | -0.98607437 |
| H | 2.40857546 | 1.33530025 | -0.29428973 |
| H | 2.13882481 | -0.66040620 | -1.14471912 |
| C | -1.61401793 | 2.68856562 | -0.64081546 |

| | | | |
|---|---|---|---|
| O | -1.80054326 | 1.57827601 | -1.20902610 |
| O | -0.57114459 | 3.27586274 | -0.31022702 |
| C | -2.95396916 | 3.41506481 | -0.30323803 |
| F | -2.78318162 | 4.60496161 | 0.30697537 |
| F | -3.72324305 | 2.65634703 | 0.52722481 |
| F | -3.69212618 | 3.64231672 | -1.41703090 |
| N | -0.46275475 | -0.88929839 | -1.12177465 |
| H | -0.01454542 | -1.09340371 | -0.22717088 |
| H | -0.77349159 | 0.10426336 | -1.10521985 |
| C | -1.64228143 | -1.76639207 | -1.29494253 |
| H | -1.29822858 | -2.75627612 | -1.62258071 |
| H | -2.22898924 | -1.33944600 | -2.11518437 |
| C | -2.50050746 | -1.92241051 | -0.05256334 |
| C | -3.19380796 | -0.82417252 | 0.48084598 |
| C | -2.60384387 | -3.16532915 | 0.58414503 |
| C | -3.97052668 | -0.97625013 | 1.62906404 |
| H | -3.11100998 | 0.14680707 | -0.00052797 |
| C | -3.38556032 | -3.31729290 | 1.73238255 |
| H | -2.07366344 | -4.02401280 | 0.17596074 |
| C | -4.07052210 | -2.22104081 | 2.25791571 |
| H | -4.50018094 | -0.11656558 | 2.03131146 |
| H | -3.45825619 | -4.28973860 | 2.21284864 |
| H | -4.67931556 | -2.33506567 | 3.15117473 |

ⅢB

0 1(charge, spin multiplicity)

| | | | |
|---|---|---|---|
| C | -4.97853107 | 0.68245071 | -1.33592196 |
| C | -3.67125379 | 0.63022112 | -0.95674593 |
| N | -3.16798998 | -0.40761807 | -0.20695322 |
| C | -3.97662977 | -1.49759633 | 0.22464153 |
| C | -5.36491092 | -1.40783834 | -0.17684664 |
| C | -5.84293642 | -0.37099188 | -0.92493799 |
| O | -3.48878360 | -2.42762105 | 0.87097131 |
| C | -1.75521683 | -0.33062947 | 0.23450164 |
| C | -0.96311961 | -1.60340675 | -0.08344878 |
| H | -5.33623031 | 1.51409606 | -1.93053475 |
| H | -2.95692935 | 1.39987305 | -1.22844266 |
| H | -5.99831826 | -2.22577695 | 0.14805160 |
| H | -6.89121327 | -0.34676324 | -1.21112887 |

| | | | |
|---|---|---|---|
| **H** | **-1.20167159** | **-2.37382009** | **0.64600500** |
| **H** | **-1.19113520** | **-1.96777223** | **-1.08774688** |
| **O** | **-1.62807604** | **-0.11484623** | **1.60858221** |
| **C** | **-1.88865516** | **1.22447379** | **2.03477321** |
| **H** | **-2.93089686** | **1.50833452** | **1.84203231** |
| **H** | **-1.70856798** | **1.23819463** | **3.11084545** |
| **H** | **-1.20780475** | **1.93060571** | **1.54644317** |
| **H** | **-1.32243024** | **0.50317656** | **-0.33311193** |
| **C** | **1.26956047** | **1.59723858** | **-0.09917855** |
| **O** | **1.33753970** | **1.08711314** | **1.04099937** |
| **O** | **1.00668137** | **1.05156461** | **-1.20771073** |
| **C** | **1.43426555** | **3.14111836** | **-0.13742424** |
| **F** | **1.75424469** | **3.61118518** | **-1.35638695** |
| **F** | **2.37010980** | **3.57488146** | **0.72707509** |
| **F** | **0.25472400** | **3.72340455** | **0.21998153** |
| **N** | **0.49899349** | **-1.29835718** | **-0.02428939** |
| **H** | **0.75674396** | **-0.58305407** | **-0.76660718** |
| **H** | **0.71934353** | **-0.71077779** | **0.81005105** |
| **C** | **1.37523325** | **-2.51897344** | **-0.07521781** |
| **H** | **1.08097762** | **-3.08276845** | **-0.96449390** |
| **H** | **1.12726706** | **-3.11801381** | **0.80587551** |
| **C** | **2.84032371** | **-2.15948655** | **-0.11860619** |
| **C** | **3.51631815** | **-1.78443788** | **1.05014957** |
| **C** | **3.53806915** | **-2.19138011** | **-1.33184071** |
| **C** | **4.86753878** | **-1.44439277** | **1.00393546** |
| **H** | **2.98550385** | **-1.75601163** | **1.99861389** |
| **C** | **4.89136003** | **-1.85432919** | **-1.37860555** |
| **H** | **3.02136467** | **-2.48305149** | **-2.24321995** |
| **C** | **5.55711620** | **-1.47970904** | **-0.21047643** |
| **H** | **5.38157868** | **-1.15294872** | **1.91525452** |
| **H** | **5.42303062** | **-1.88423345** | **-2.32536555** |
| **H** | **6.61057507** | **-1.21637117** | **-0.24509067** |

**methylamine**

0 1(charge, spin multiplicity)

| | | | |
|---|---|---|---|
| C | -0.70658405 | -0.00000500 | 0.01736200 |
| H | -1.11834908 | 0.88275707 | -0.48415704 |
| H | -1.07973808 | -0.00061300 | 1.05604108 |
| H | -1.11851509 | -0.88207207 | -0.48525304 |

| | | | |
|---|---|---|---|
| N | 0.75395006 | -0.00000600 | -0.12866301 |
| H | 1.13921209 | 0.81030906 | 0.35492403 |
| H | 1.13925009 | -0.81030806 | 0.35491603 |

ⅡA(C8):

0 1(charge, spin multiplicity)

| | | | |
|---|---|---|---|
| **C** | **-2.12693274** | **-2.31904149** | **-1.33940484** |
| **C** | **-1.38508722** | **-1.58480736** | **-0.43592153** |
| **N** | **-1.89620470** | **-0.40636986** | **0.00236761** |
| **C** | **-3.06750597** | **0.10598201** | **-0.43953812** |
| **C** | **-3.85838164** | **-0.60400784** | **-1.34154973** |
| **C** | **-3.37026581** | **-1.82857676** | **-1.77924274** |
| **O** | **-3.32455317** | **1.30423273** | **0.05748890** |
| **C** | **-1.33267787** | **0.48376344** | **1.08628050** |
| **C** | **-2.12735668** | **1.75481626** | **0.78935126** |
| **O** | **-1.66218951** | **0.01083582** | **2.34765575** |
| **C** | **-0.86929600** | **-1.07973005** | **2.84015818** |
| **H** | **-1.73517680** | **-3.25938766** | **-1.70769168** |
| **H** | **-0.38747644** | **-1.84295826** | **-0.06356737** |
| **H** | **-4.79577835** | **-0.18455239** | **-1.68447821** |
| **H** | **-3.95361118** | **-2.40630177** | **-2.48952953** |
| **H** | **-0.25631428** | **0.56493269** | **0.89665251** |
| **H** | **-1.55825453** | **2.42827162** | **0.13416271** |
| **H** | **-2.46781556** | **2.24472793** | **1.69972917** |
| **H** | **-0.94257946** | **-1.04305464** | **3.92878465** |
| **H** | **-1.26737665** | **-2.03883734** | **2.48646965** |
| **H** | **0.17429174** | **-0.99397811** | **2.52226019** |
| **N** | **0.35100914** | **3.09645382** | **-0.95389914** |
| **H** | **0.23772936** | **3.20465412** | **-1.96143320** |
| **H** | **0.87048246** | **2.22471040** | **-0.82335162** |
| **C** | **1.98014427** | **-0.72641943** | **-0.01382280** |
| **O** | **1.37381746** | **0.31728257** | **-0.35635796** |
| **O** | **1.52846085** | **-1.79196353** | **0.46070697** |
| **C** | **3.52354087** | **-0.65599556** | **-0.21700486** |
| **F** | **4.05984305** | **0.35717707** | **0.50705150** |
| **F** | **4.16401069** | **-1.78508885** | **0.14410173** |
| **F** | **3.83142265** | **-0.41983509** | **-1.51598731** |
| **C** | **1.14260981** | **4.21984243** | **-0.43750704** |
| **H** | **0.59082349** | **5.15685583** | **-0.57551013** |

| | | | |
|---|---|---|---|
| H | 1.29849910 | 4.08496008 | 0.63834359 |
| H | 2.13440724 | 4.34103707 | -0.90335232 |

TS II 2:

0 1(charge, spin multiplicity)

| | | | |
|---|---|---|---|
| C | -3.72768104 | -1.54729307 | -1.34928921 |
| C | -2.41054636 | -1.21509382 | -1.15202158 |
| N | -2.10959928 | -0.07714885 | -0.46460387 |
| C | -3.04880951 | 0.81032405 | -0.00174791 |
| C | -4.41384417 | 0.47471925 | -0.17505506 |
| C | -4.73219174 | -0.69287100 | -0.83694905 |
| O | -2.57193285 | 1.87552828 | 0.51889970 |
| C | -0.72504951 | 0.31650700 | -0.06369367 |
| C | -0.76716184 | 1.82215189 | 0.07469544 |
| O | -0.39546513 | -0.21220350 | 1.18811643 |
| C | -0.06929348 | -1.60822280 | 1.19095498 |
| H | -3.97983504 | -2.44517705 | -1.89986757 |
| H | -1.55846609 | -1.76871868 | -1.53448567 |
| H | -5.16622223 | 1.15837963 | 0.19878984 |
| H | -5.77686779 | -0.95113114 | -0.98465431 |
| H | -0.06973398 | -0.05331825 | -0.85737758 |
| H | -0.92221207 | 2.43921560 | -0.79626896 |
| H | -0.47909182 | 2.27827163 | 1.00660123 |
| H | 0.41988217 | -1.80155273 | 2.14753016 |
| H | -0.97517807 | -2.22494067 | 1.12548849 |
| H | 0.59942592 | -1.85843145 | 0.36133816 |
| N | 1.35518045 | 2.03072095 | -0.44166412 |
| H | 1.45920370 | 2.98052893 | -0.79828634 |
| H | 1.56455701 | 1.38625686 | -1.22573922 |
| C | 1.46987642 | -0.91664951 | -2.79127675 |
| O | 1.94095426 | 0.23133341 | -2.63227090 |
| O | 0.57384869 | -1.52166520 | -2.15589395 |
| C | 2.14403251 | -1.74639255 | -3.92685866 |
| F | 2.60467123 | -0.97390137 | -4.93339995 |
| F | 3.20556706 | -2.43624358 | -3.43581515 |
| F | 1.30365912 | -2.65155206 | -4.47487363 |
| C | 2.28619535 | 1.80738397 | 0.67338476 |
| H | 2.05048645 | 2.48898033 | 1.49732677 |
| H | 2.16786906 | 0.78383444 | 1.03489424 |

| | | | |
|---|---|---|---|
| H | 3.33430555 | 1.95938285 | 0.38550516 |

ⅡC:

0 1(charge, spin multiplicity)

| | | | |
|---|---|---|---|
| C | -2.20990738 | -2.79990123 | 0.24878026 |
| C | -1.50886486 | -1.63717707 | 0.37568670 |
| N | -2.03244571 | -0.43259962 | -0.03512782 |
| C | -3.31155208 | -0.31579388 | -0.64202347 |
| C | -4.04449079 | -1.55937225 | -0.73502144 |
| C | -3.51541449 | -2.74658125 | -0.31398245 |
| O | -3.70865121 | 0.77857497 | -1.05375193 |
| C | -1.24926033 | 0.79520371 | 0.22875215 |
| C | -1.05182361 | 1.64130828 | -1.03415120 |
| O | -1.85092331 | 1.61872424 | 1.19715278 |
| C | -1.81654182 | 1.08692363 | 2.52083117 |
| H | -1.76842193 | -3.73407724 | 0.57404811 |
| H | -0.50316189 | -1.59316389 | 0.78134316 |
| H | -5.03243802 | -1.49150910 | -1.17694504 |
| H | -4.09360520 | -3.66180542 | -0.41350000 |
| H | -0.27195647 | 0.43876516 | 0.57971718 |
| H | -1.02581409 | 1.00467173 | -1.91977630 |
| H | -1.85757777 | 2.36577958 | -1.13689042 |
| H | -2.22485668 | 1.86166442 | 3.17263609 |
| H | -2.43422550 | 0.18471215 | 2.60779408 |
| H | -0.78905542 | 0.85047586 | 2.82927026 |
| N | 0.26797622 | 2.36272814 | -0.99636562 |
| H | 0.39135571 | 2.81775306 | -1.90474037 |
| H | 1.10762447 | 1.64483940 | -0.91974159 |
| C | 2.44410907 | -0.08298678 | -0.00973371 |
| O | 2.35340648 | 0.79608841 | -0.92015401 |
| O | 1.63312973 | -0.39360969 | 0.87613531 |
| C | 3.79852219 | -0.84553304 | -0.04245543 |
| F | 4.02267975 | -1.39108534 | -1.25899819 |
| F | 4.82572549 | -0.00347056 | 0.21777996 |
| F | 3.85162203 | -1.83917669 | 0.86183555 |
| C | 0.42245806 | 3.39583128 | 0.06946402 |
| H | -0.39040433 | 4.12078522 | 0.00259716 |
| H | 0.39806097 | 2.90781199 | 1.04169301 |
| H | 1.38641989 | 3.88623881 | -0.07454404 |

ⅡD

0 1(charge, spin multiplicity)

| | | | |
|---|---|---|---|
| C | 2.87901931 | 0.71817453 | -1.12495028 |
| C | 1.52129896 | 0.84073964 | -1.09638481 |
| N | 0.72836020 | 0.08509305 | -0.27003829 |
| C | 1.27155792 | -0.88604850 | 0.62020532 |
| C | 2.71850563 | -0.98368821 | 0.58620090 |
| C | 3.48586008 | -0.22449610 | -0.24896088 |
| O | 0.54226901 | -1.57357818 | 1.33571676 |
| C | -0.74318004 | 0.33549039 | -0.28108017 |
| C | -1.57534511 | -0.91408818 | -0.59871821 |
| O | -1.18248631 | 0.88343456 | 0.93105396 |
| C | -0.74134030 | 2.21168514 | 1.17916257 |
| H | 3.46510691 | 1.33315663 | -1.79713104 |
| H | 0.99278201 | 1.54383845 | -1.73151692 |
| H | 3.15065922 | -1.71244831 | 1.26317640 |
| H | 4.56709521 | -0.33800725 | -0.24699037 |
| H | -0.90441155 | 1.05176274 | -1.09865833 |
| H | -1.04454242 | -1.46917126 | -1.37950791 |
| H | -1.61978877 | -1.54748689 | 0.29497599 |
| H | -1.22741847 | 2.53266479 | 2.10268609 |
| H | 0.34708047 | 2.26180217 | 1.31331872 |
| H | -1.03294246 | 2.88905161 | 0.36257987 |
| N | -2.89375428 | -0.53291781 | -1.11741277 |
| H | -3.19853890 | -1.25440078 | -1.76576036 |
| C | -3.92093870 | -0.37641419 | -0.08467080 |
| H | -4.04487537 | -1.26734453 | 0.55679027 |
| H | -3.66202235 | 0.46371202 | 0.56373048 |
| H | -4.88131291 | -0.15933419 | -0.56452314 |

ⅡA(C2):

0 1(charge, spin multiplicity)

| | | | |
|---|---|---|---|
| H | -1.17433445 | -0.80926665 | -3.12191924 |
| H | -2.11247316 | -2.08146637 | -2.26668327 |
| H | -3.15644618 | 3.58677174 | 0.53626555 |
| C | -1.40673612 | 2.35619463 | 0.84837231 |
| C | -0.74109852 | 1.20844350 | 0.47310149 |
| N | -1.30302358 | 0.42783583 | -0.48746648 |

| | | | |
|---|---|---|---|
| **C** | **-2.47208974** | **0.72942884** | **-1.09264319** |
| **C** | **-3.18438070** | **1.87481439** | **-0.74411864** |
| **C** | **-2.63410150** | **2.68246137** | **0.23964233** |
| **C** | **-0.80672636** | **-0.91400410** | **-0.95867206** |
| **C** | **-1.69536375** | **-1.07796488** | **-2.20022716** |
| **O** | **-2.80368932** | **-0.13922340** | **-2.03757119** |
| **H** | **-0.96805788** | **2.99979191** | **1.60129774** |
| **H** | **0.23816343** | **0.88324538** | **0.84769912** |
| **H** | **-4.12096148** | **2.09774133** | **-1.23874531** |
| **N** | **-4.00688440** | **-1.14008868** | **1.00335951** |
| **H** | **-4.75407761** | **-1.77300039** | **0.72121905** |
| **H** | **-3.13583513** | **-1.64990862** | **0.85962035** |
| **O** | **-1.09364298** | **-1.89757139** | **-0.01569458** |
| **C** | **0.03439326** | **-2.38069047** | **0.74615334** |
| **H** | **0.73276527** | **-2.91094973** | **0.09027642** |
| **H** | **0.56188544** | **-1.56445004** | **1.24690447** |
| **H** | **-0.38462877** | **-3.07436389** | **1.47661745** |
| **H** | **0.27073604** | **-0.81913662** | **-1.16028214** |
| **C** | **2.63813478** | **-0.00024261** | **0.06191613** |
| **O** | **2.19473268** | **-0.35263035** | **-1.05270564** |
| **O** | **2.03580059** | **0.28581188** | **1.12662268** |
| **C** | **4.18797928** | **0.15749831** | **0.14289616** |
| **F** | **4.54137662** | **1.44983994** | **-0.08380965** |
| **F** | **4.67589351** | **-0.17769331** | **1.35774136** |
| **F** | **4.84106987** | **-0.59579687** | **-0.76715463** |
| **C** | **-4.15741890** | **-0.81305928** | **2.42561343** |
| **H** | **-5.09057647** | **-0.25866527** | **2.57601434** |
| **H** | **-4.16822963** | **-1.67829295** | **3.10989416** |
| **H** | **-3.33479179** | **-0.15779973** | **2.73216146** |

**TS II 1:**

0 1(charge, spin multiplicity)

| | | | |
|---|---|---|---|
| **H** | **2.88647354** | **1.28609072** | **2.36360480** |
| **H** | **1.26558660** | **2.02043745** | **2.44956675** |
| **H** | **3.44922513** | **-3.33884874** | **-0.63007570** |
| **C** | **3.91784793** | **-1.23830207** | **-0.88209323** |
| **C** | **3.46054308** | **0.04547652** | **-0.70061542** |
| **N** | **2.21062475** | **0.27107433** | **-0.19171773** |
| **C** | **1.41057640** | **-0.77021576** | **0.22020102** |

| | | | |
|---|---|---|---|
| C | 1.84074527 | -2.09185454 | 0.01741345 |
| C | 3.10152901 | -2.31857824 | -0.50013424 |
| C | 1.91706982 | 1.58052106 | 0.46153279 |
| C | 1.85244528 | 1.21126329 | 1.98375252 |
| O | 1.31092802 | -0.03940603 | 2.19613730 |
| H | 4.89823473 | -1.39302051 | -1.31598751 |
| H | 4.04592036 | 0.93959428 | -0.96160175 |
| H | 1.19490286 | -2.91445252 | 0.29720864 |
| N | -0.05255730 | -0.47931342 | 0.33790806 |
| H | -0.05715399 | -0.33522931 | 1.40505681 |
| H | -0.18091927 | 0.49101130 | -0.01326433 |
| O | 0.66569590 | 2.07821967 | 0.02085549 |
| C | 0.73153966 | 2.81838384 | -1.21786334 |
| H | 1.53189458 | 3.56049423 | -1.16432517 |
| H | 0.91278314 | 2.14559748 | -2.06578632 |
| H | -0.24110286 | 3.30015762 | -1.33267289 |
| H | 2.71594224 | 2.29073587 | 0.21099300 |
| C | 4.74178149 | 3.52704742 | -1.24785795 |
| O | 3.78939816 | 3.86953286 | -0.50904447 |
| O | 5.11608662 | 2.39240123 | -1.62005175 |
| C | 5.63835099 | 4.70499631 | -1.74178341 |
| F | 6.62229338 | 4.96109520 | -0.84089854 |
| F | 6.24382830 | 4.44289920 | -2.92155229 |
| F | 4.94406925 | 5.85404115 | -1.90302629 |
| C | -1.02869501 | -1.42553224 | -0.26715383 |
| H | -0.99378311 | -2.37946377 | 0.25726478 |
| H | -2.02133455 | -0.98860804 | -0.15198582 |
| H | -0.80283977 | -1.56660227 | -1.32505469 |

**ⅡB**

0 1(charge, spin multiplicity)

| | | | |
|---|---|---|---|
| H | 0.05469677 | -2.08462190 | 1.65646616 |
| H | 1.65622138 | -1.40724965 | 2.00887132 |
| H | 3.43404285 | 3.69424493 | -0.41460395 |
| C | 1.53846878 | 2.65298349 | -0.52158676 |
| C | 0.94021255 | 1.42026609 | -0.43917710 |
| N | 1.66831365 | 0.28493526 | -0.21596674 |
| C | 3.03400666 | 0.33354055 | -0.04244852 |
| C | 3.67064739 | 1.59401436 | -0.12208170 |

| | | | |
|---|---|---|---|
| C | 2.93044255 | 2.73413563 | -0.35560740 |
| C | 0.85900286 | -0.97331226 | 0.03181209 |
| C | 0.68574007 | -1.19013374 | 1.54768353 |
| O | 0.14285770 | -0.05528969 | 2.15690825 |
| H | 0.93099660 | 3.53035298 | -0.70538368 |
| H | -0.13177035 | 1.25886050 | -0.54584801 |
| H | 4.74444460 | 1.64484293 | -0.00021903 |
| N | 3.70977046 | -0.80782434 | 0.19984053 |
| H | -0.82767140 | -0.03019311 | 1.91505939 |
| H | 3.20094072 | -1.67332054 | 0.04962815 |
| O | 1.45159968 | -2.10008018 | -0.54872371 |
| C | 1.21895923 | -2.24398510 | -1.95770122 |
| H | 0.14409077 | -2.23963064 | -2.16735973 |
| H | 1.70538355 | -1.44157746 | -2.52561505 |
| H | 1.64792384 | -3.20581712 | -2.24274500 |
| H | -0.11811200 | -0.75882137 | -0.41288275 |
| C | -2.70600315 | 0.10240559 | 0.22086088 |
| O | -2.44055967 | -0.14166790 | 1.42108628 |
| O | -1.95044888 | 0.33154936 | -0.75266052 |
| C | -4.21932567 | 0.10348598 | -0.14785902 |
| F | -4.56830558 | 1.25610077 | -0.76696489 |
| F | -4.50113952 | -0.90893161 | -1.00564121 |
| F | -5.03174482 | -0.03916114 | 0.91613885 |
| C | 5.15093991 | -0.86468486 | 0.39294016 |
| H | 5.45821662 | -0.24470396 | 1.24141888 |
| H | 5.41826120 | -1.89930991 | 0.61142203 |
| H | 5.70031770 | -0.54329127 | -0.50101202 |

**Phenylethylamine**

0 1(charge, spin multiplicity)

| | | | |
|---|---|---|---|
| C | -0.80262900 | 1.20736600 | 0.18723600 |
| C | -0.10451800 | 0.00645700 | 0.37672500 |
| C | -0.80333400 | -1.19887000 | 0.21803300 |
| C | -2.15747800 | -1.20590200 | -0.12026900 |
| C | -2.83951900 | -0.00154800 | -0.30742300 |
| C | -2.15694300 | 1.20663600 | -0.15187800 |

| | | | |
|---|---|---|---|
| H | -0.27971200 | 2.15342500 | 0.31253700 |
| H | -0.28003600 | -2.14113700 | 0.36711900 |
| H | -2.68092500 | -2.15193500 | -0.23323600 |
| H | -3.89468000 | -0.00466300 | -0.56765400 |
| H | -2.67960300 | 2.14978100 | -0.29002500 |
| C | 1.37278200 | 0.00608600 | 0.70439400 |
| H | 1.62343000 | 0.90188300 | 1.28940200 |
| H | 1.62910100 | -0.85993400 | 1.32558100 |
| C | 2.25906100 | -0.02730100 | -0.55264800 |
| H | 2.02894500 | -0.93304000 | -1.12711900 |
| H | 1.99438100 | 0.82929700 | -1.19790400 |
| N | 3.67784900 | -0.07950400 | -0.17739400 |
| H | 4.24746100 | -0.14924700 | -1.01983900 |
| H | 3.94216600 | 0.80455500 | 0.25787800 |

ⅣA(C2)

0 1(charge, spin multiplicity)

| | | | |
|---|---|---|---|
| C | 4.04063600 | -0.73957600 | -1.88572100 |
| C | 3.15731400 | -0.70520300 | -0.82538700 |
| N | 2.94639200 | 0.48354000 | -0.20475700 |
| C | 3.54317400 | 1.63440500 | -0.58986400 |
| C | 4.45329800 | 1.64789800 | -1.64584600 |
| C | 4.69471400 | 0.43962700 | -2.28619300 |
| O | 3.15910400 | 2.68096000 | 0.12240900 |
| C | 2.14838900 | 0.72081300 | 1.05936300 |
| C | 2.06662900 | 2.24985700 | 1.01021700 |
| H | 4.21269400 | -1.67719900 | -2.40023400 |
| H | 2.57700900 | -1.56179200 | -0.45833800 |
| H | 4.92567800 | 2.57703400 | -1.93893000 |
| H | 5.39232600 | 0.41158500 | -3.11762500 |

| | | | |
|---|---|---|---|
| **H** | **2.24938300** | **2.69292100** | **1.98797400** |
| **H** | **1.12265100** | **2.59616000** | **0.56775700** |
| **O** | **2.87485900** | **0.29562100** | **2.16320400** |
| **C** | **2.46704800** | **-0.96813400** | **2.72101900** |
| **H** | **2.49734300** | **-1.77268100** | **1.98015400** |
| **H** | **3.17146900** | **-1.17220200** | **3.52912400** |
| **H** | **1.45098100** | **-0.89722800** | **3.12443500** |
| **H** | **1.18988500** | **0.19956600** | **0.94064200** |
| **C** | **0.18121800** | **-2.38424900** | **0.21343500** |
| **O** | **-0.13147100** | **-1.18657100** | **0.40657600** |
| **O** | **1.30866600** | **-2.91464800** | **0.08646100** |
| **C** | **-1.03847900** | **-3.34544800** | **0.06713000** |
| **F** | **-1.59312500** | **-3.21539800** | **-1.17138400** |
| **F** | **-2.01404800** | **-3.06759000** | **0.96054800** |
| **F** | **-0.71593200** | **-4.64450200** | **0.21820300** |
| **C** | **-2.68975800** | **2.59105800** | **1.21394400** |
| **H** | **-3.33902000** | **3.09645000** | **1.94024400** |
| **H** | **-1.95411400** | **2.01293000** | **1.78550800** |
| **C** | **-3.51846600** | **1.66288000** | **0.34567700** |
| **C** | **-3.05850500** | **0.38430600** | **-0.00060900** |
| **C** | **-4.76111600** | **2.08569900** | **-0.15319000** |
| **C** | **-3.81953200** | **-0.44603400** | **-0.83003100** |
| **H** | **-2.10654300** | **0.01621800** | **0.37573000** |
| **C** | **-5.52192700** | **1.25849200** | **-0.97913500** |
| **H** | **-5.13867200** | **3.07096800** | **0.11513500** |
| **C** | **-5.05073000** | **-0.01215100** | **-1.32236600** |
| **H** | **-3.43929800** | **-1.43198600** | **-1.08087900** |
| **H** | **-6.48447300** | **1.60243600** | **-1.35006000** |
| **H** | **-5.64382300** | **-0.65943000** | **-1.96353700** |
| **C** | **-1.94953800** | **3.67472300** | **0.41016500** |

| | | | |
|---|---|---|---|
| H | -2.68955900 | 4.23782800 | -0.18300800 |
| H | -1.48859300 | 4.38730100 | 1.10577200 |
| N | -0.87916100 | 3.08865000 | -0.41441400 |
| H | -1.27417600 | 2.33183000 | -0.97431000 |
| H | -0.54930700 | 3.78471400 | -1.08252200 |

TSⅣ2

0 1(charge, spin multiplicity)

| | | | |
|---|---|---|---|
| C | 4.22543500 | -3.83851600 | 0.07335100 |
| C | 4.62543700 | -2.55817100 | -0.17510800 |
| N | 4.02822000 | -1.84162200 | -1.17808800 |
| C | 2.84183200 | -2.27035700 | -1.76450000 |
| C | 2.60895600 | -3.70414900 | -1.74785100 |
| C | 3.25675500 | -4.43744400 | -0.80050600 |
| O | 2.60699500 | -1.55379700 | -2.88622900 |
| C | 4.53005600 | -0.61153700 | -1.84005000 |
| C | 3.33251200 | -0.29815400 | -2.74700000 |
| H | 4.68645000 | -4.40695600 | 0.87132100 |
| H | 5.41589300 | -2.05355200 | 0.36686000 |
| H | 1.90145900 | -4.12659000 | -2.45120600 |
| H | 3.06352700 | -5.50344100 | -0.72102300 |
| H | 3.64593700 | 0.01327200 | -3.74187600 |
| H | 2.67757800 | 0.44184900 | -2.27917300 |
| O | 5.66281400 | -0.84912700 | -2.62771800 |
| C | 6.91560100 | -0.74288500 | -1.95516800 |
| H | 7.12023800 | -1.62250800 | -1.33096400 |
| H | 7.67955100 | -0.68231500 | -2.73322500 |
| H | 6.95728300 | 0.16027200 | -1.33262800 |
| H | 4.69224500 | 0.15872300 | -1.07628900 |
| C | 2.81085600 | 1.53492900 | 0.65611200 |

| | | | |
|---|---|---|---|
| **O** | **1.84056200** | **1.13065900** | **-0.04861800** |
| **O** | **3.99915600** | **1.17574800** | **0.67190400** |
| **C** | **2.39937600** | **2.63575100** | **1.67883100** |
| **F** | **3.45015500** | **3.20027500** | **2.30244300** |
| **F** | **1.60249000** | **2.10489600** | **2.64832000** |
| **F** | **1.69672300** | **3.63044600** | **1.09324800** |
| **N** | **1.51022300** | **-1.56293200** | **-0.59237000** |
| **H** | **1.61801100** | **-2.06021100** | **0.29387100** |
| **H** | **1.74076800** | **-0.55595200** | **-0.40911200** |
| **C** | **0.12378800** | **-1.68326600** | **-1.08167100** |
| **H** | **-0.11387900** | **-2.74870700** | **-1.17684300** |
| **H** | **0.09502100** | **-1.24003700** | **-2.08159300** |
| **C** | **-0.89982200** | **-0.97809000** | **-0.16786500** |
| **H** | **-1.88404100** | **-1.07354800** | **-0.64394400** |
| **H** | **-0.65249600** | **0.08806300** | **-0.13533100** |
| **C** | **-0.94130700** | **-1.54198500** | **1.23795800** |
| **C** | **-0.24561100** | **-0.91240000** | **2.28083900** |
| **C** | **-1.65094100** | **-2.71922800** | **1.51900500** |
| **C** | **-0.25961100** | **-1.44916500** | **3.57091300** |
| **H** | **0.29997300** | **0.00628100** | **2.08217800** |
| **C** | **-1.66620100** | **-3.25604600** | **2.80684400** |
| **H** | **-2.20532100** | **-3.21357100** | **0.72330000** |
| **C** | **-0.96814300** | **-2.62180400** | **3.83790100** |
| **H** | **0.28053100** | **-0.94424500** | **4.36752500** |
| **H** | **-2.22814600** | **-4.16478100** | **3.00720900** |
| **H** | **-0.98259000** | **-3.03604300** | **4.84255400** |

ⅣC

0 1(charge, spin multiplicity)

| | | | |
|---|---|---|---|
| **C** | **4.11558100** | **1.38290900** | **0.03873300** |

| | | | |
|---|---|---|---|
| C | 2.94628800 | 2.02634800 | 0.33283800 |
| N | 1.73059300 | 1.54762900 | -0.06752100 |
| C | 1.62047000 | 0.41471100 | -0.85067400 |
| C | 2.82606400 | -0.29876900 | -1.11997800 |
| C | 4.03883100 | 0.17669500 | -0.68892400 |
| O | 1.27425000 | 4.19744600 | -0.95833800 |
| C | 0.50278100 | 2.35170000 | 0.35485100 |
| C | 0.14871000 | 3.36413700 | -0.72732000 |
| H | 5.05926700 | 1.79052200 | 0.37926300 |
| H | 2.88951200 | 2.93714200 | 0.91107900 |
| H | 2.76685000 | -1.23818700 | -1.64996800 |
| H | 4.94016500 | -0.38795300 | -0.90766600 |
| H | -0.71894000 | 3.92859300 | -0.36032800 |
| H | -0.15879400 | 2.82594300 | -1.63339600 |
| O | 0.76854600 | 3.01929600 | 1.54412500 |
| C | 0.58840600 | 2.21804500 | 2.72316700 |
| H | 1.37941100 | 1.46212500 | 2.81345700 |
| H | 0.65234400 | 2.90661900 | 3.56757100 |
| H | -0.38810600 | 1.72503900 | 2.70422600 |
| H | -0.31438500 | 1.63839600 | 0.49373900 |
| C | -2.57405600 | 0.52352700 | 0.11326700 |
| O | -2.12823000 | 0.89806800 | -1.00819200 |
| O | -2.00608800 | 0.48711200 | 1.22046200 |
| C | -4.01787600 | -0.05259700 | 0.05469200 |
| F | -4.60804100 | -0.11819600 | 1.26239200 |
| F | -3.97782600 | -1.32077000 | -0.44097700 |
| F | -4.82925800 | 0.66207000 | -0.75286100 |
| N | 0.42629700 | 0.05604400 | -1.33307400 |
| H | -0.45437000 | 0.55925900 | -1.09097400 |
| H | 1.03035300 | 4.84244500 | -1.63897200 |

| | | | |
|---|---|---|---|
| C | 0.15572900 | -1.09128200 | -2.20117900 |
| H | 1.07117100 | -1.40309500 | -2.70756400 |
| H | -0.53310400 | -0.73355500 | -2.97301400 |
| C | -0.50404100 | -2.28257600 | -1.47150500 |
| H | -0.71672700 | -3.04046300 | -2.23642500 |
| H | -1.47095700 | -1.95109000 | -1.08046900 |
| C | 0.33869400 | -2.88010500 | -0.36637700 |
| C | 0.19147600 | -2.44797900 | 0.95995000 |
| C | 1.29775500 | -3.86382800 | -0.64970400 |
| C | 0.98977900 | -2.98348200 | 1.97340700 |
| H | -0.55371100 | -1.69184100 | 1.19719400 |
| C | 2.09613700 | -4.39994200 | 0.36199100 |
| H | 1.41063000 | -4.22253400 | -1.67141500 |
| C | 1.94467700 | -3.95880000 | 1.67865400 |
| H | 0.85853700 | -2.64217500 | 2.99709600 |
| H | 2.82809600 | -5.16775200 | 0.12419300 |
| H | 2.56039600 | -4.37872900 | 2.46975300 |

ⅣD

0 1(charge, spin multiplicity)

| | | | |
|---|---|---|---|
| C | 3.41245400 | -1.58988800 | -0.27140200 |
| C | 3.68751400 | -0.52771600 | 0.58550100 |
| N | 2.89472000 | 0.53836400 | 0.74290200 |
| C | 1.74622000 | 0.58479400 | 0.03723700 |
| C | 1.36209400 | -0.45243500 | -0.84231800 |
| C | 2.21497800 | -1.53651200 | -0.99297100 |
| H | 4.09928100 | -2.42475400 | -0.36336400 |
| H | 4.60073500 | -0.52324600 | 1.18017400 |
| H | 0.41129700 | -0.42003700 | -1.36135400 |
| H | 1.93941300 | -2.34596600 | -1.66439200 |

| | | | |
|---|---|---|---|
| N | 0.97825100 | 1.70635200 | 0.26462700 |
| H | 1.44238300 | 2.36848200 | 0.87308600 |
| C | -0.05636400 | 2.23384900 | -0.60939100 |
| H | -0.00795600 | 1.72415300 | -1.57723000 |
| H | 0.16097900 | 3.29234400 | -0.80270800 |
| C | -1.48866700 | 2.13112200 | -0.03347600 |
| H | -2.15136700 | 2.70172100 | -0.69747100 |
| H | -1.50380000 | 2.63556400 | 0.94032200 |
| C | -1.99992900 | 0.71481500 | 0.10661500 |
| C | -1.81184800 | -0.00921100 | 1.29198800 |
| C | -2.65160900 | 0.08292200 | -0.96306700 |
| C | -2.25546700 | -1.32810000 | 1.40383900 |
| H | -1.31109600 | 0.46525300 | 2.13192800 |
| C | -3.09717900 | -1.23566400 | -0.85576700 |
| H | -2.81792500 | 0.63328000 | -1.88730900 |
| C | -2.89899800 | -1.94646200 | 0.33003600 |
| H | -2.09994500 | -1.87213400 | 2.33189700 |
| H | -3.60445300 | -1.70468800 | -1.69506800 |
| H | -3.24754200 | -2.97206200 | 0.41784300 |

ⅣA(C8)

0 1(charge, spin multiplicity)

| | | | |
|---|---|---|---|
| C | 3.47361400 | -0.15498100 | 2.50960700 |
| C | 2.99161300 | 0.20461600 | 1.26700000 |
| N | 2.96880600 | -0.73900600 | 0.29073200 |
| C | 3.35676900 | -2.02006000 | 0.49016200 |
| C | 3.86374500 | -2.42741500 | 1.72287500 |
| C | 3.92117900 | -1.47078100 | 2.72818700 |
| O | 3.18143700 | -2.78313300 | -0.57625800 |
| C | 2.63816800 | -0.53609600 | -1.17330800 |

| | | | |
|---|---|---|---|
| C | 2.43415300 | -1.99736100 | -1.57136100 |
| H | 3.48869000 | 0.58046700 | 3.30481100 |
| H | 2.56712900 | 1.18224100 | 1.00984200 |
| H | 4.17288500 | -3.45504800 | 1.86646500 |
| H | 4.30171400 | -1.75276900 | 3.70507100 |
| H | 2.85637000 | -2.22057300 | -2.54925400 |
| H | 1.37624200 | -2.27416200 | -1.49473200 |
| O | 3.72397100 | 0.00262400 | -1.84840400 |
| C | 3.77666800 | 1.43747400 | -1.87711500 |
| H | 4.08090900 | 1.84666300 | -0.90693500 |
| H | 4.52601800 | 1.69411000 | -2.62773400 |
| H | 2.80413500 | 1.85714300 | -2.15180500 |
| H | 1.73722800 | 0.08890600 | -1.22653300 |
| C | 0.34651800 | 2.28068800 | -0.05172800 |
| O | 0.21062900 | 1.33523900 | -0.86792000 |
| O | 1.34396500 | 2.64362800 | 0.60551900 |
| C | -0.95461800 | 3.09372700 | 0.22678500 |
| F | -0.71361700 | 4.39373400 | 0.48434100 |
| F | -1.59051200 | 2.58256100 | 1.32028100 |
| F | -1.83812600 | 3.04436700 | -0.79444300 |
| N | -0.86048000 | -1.48236000 | -1.00694000 |
| H | -1.30267800 | -1.83557800 | -0.15731200 |
| H | -0.54193100 | -0.53420700 | -0.78537600 |
| C | -1.88294100 | -1.37101600 | -2.05502200 |
| H | -2.26109900 | -2.37703100 | -2.27882900 |
| H | -1.39199500 | -1.00572000 | -2.96532300 |
| C | -3.07033900 | -0.43011200 | -1.73060700 |
| H | -3.71578200 | -0.37094000 | -2.61798100 |
| H | -2.67169000 | 0.57555300 | -1.55253000 |
| C | -3.88162300 | -0.88509100 | -0.53669800 |

| | | | |
|---|---|---|---|
| C | -3.69426400 | -0.31023000 | 0.72912100 |
| C | -4.81582400 | -1.92570200 | -0.66082600 |
| C | -4.41782300 | -0.76252300 | 1.83606400 |
| H | -2.98981100 | 0.50943700 | 0.84547100 |
| C | -5.53995600 | -2.37975000 | 0.44167300 |
| H | -4.98133100 | -2.37949800 | -1.63623500 |
| C | -5.34182800 | -1.79921500 | 1.69736800 |
| H | -4.26177200 | -0.29806200 | 2.80663400 |
| H | -6.26279600 | -3.18285600 | 0.32035100 |
| H | -5.90770600 | -2.14837000 | 2.55721900 |

TSⅣ1

0 1(charge, spin multiplicity)

| | | | |
|---|---|---|---|
| C | 5.60193700 | -0.50407200 | -3.43001500 |
| C | 4.52605600 | -0.18643800 | -2.64470200 |
| N | 3.27240300 | -0.57013200 | -3.01917400 |
| C | 3.01831000 | -1.28822000 | -4.15864800 |
| C | 4.11374600 | -1.62085000 | -4.99687500 |
| C | 5.37977100 | -1.22687600 | -4.62837200 |
| O | 1.79623000 | -1.60379800 | -4.34352600 |
| C | 2.01277500 | -0.15789200 | -2.30419600 |
| C | 0.90872600 | -1.05359900 | -2.85357800 |
| H | 6.59794800 | -0.20369600 | -3.12921800 |
| H | 4.60196200 | 0.35991600 | -1.71259500 |
| H | 3.91474200 | -2.18290600 | -5.90105300 |
| H | 6.22414800 | -1.48039200 | -5.26224300 |
| H | 0.11916000 | -0.60020500 | -3.42857500 |
| H | 0.78507800 | -2.05950900 | -2.48490900 |
| O | 1.68264800 | 1.15171000 | -2.56311400 |
| C | 2.27297300 | 2.16493600 | -1.72978800 |

| | | | |
|---|---|---|---|
| H | 3.18087900 | 2.56315100 | -2.19875000 |
| H | 1.50974500 | 2.93774700 | -1.60439000 |
| H | 2.52022100 | 1.75441800 | -0.74142600 |
| H | 2.19524900 | -0.34352200 | -1.24034300 |
| C | -1.39339400 | 3.17758600 | -0.93312100 |
| O | -1.62893800 | 1.99216000 | -1.28932000 |
| O | -0.33473400 | 3.82546200 | -0.88796500 |
| C | -2.68191300 | 3.92734100 | -0.46857100 |
| F | -2.45957200 | 5.20422500 | -0.09866400 |
| F | -3.25257800 | 3.30347400 | 0.60222800 |
| F | -3.62190900 | 3.95460700 | -1.44422200 |
| N | -0.41126800 | -0.51337500 | -1.20332900 |
| H | -0.01837500 | -0.69395300 | -0.27910100 |
| H | -0.58190400 | 0.50822200 | -1.26636700 |
| C | -1.71666100 | -1.19968500 | -1.34223500 |
| H | -1.68675200 | -1.85270700 | -2.22667200 |
| H | -2.47741500 | -0.43930300 | -1.53885500 |
| C | -2.10240000 | -2.05914500 | -0.13235200 |
| H | -3.06882200 | -2.53066400 | -0.36322000 |
| H | -1.38807900 | -2.88982000 | -0.03936600 |
| C | -2.19634800 | -1.34143300 | 1.20705800 |
| C | -2.02076600 | -2.07914700 | 2.38822300 |
| C | -2.48284800 | 0.02764900 | 1.31293000 |
| C | -2.14058500 | -1.47629100 | 3.64043900 |
| H | -1.79148400 | -3.14155100 | 2.32411300 |
| C | -2.59524400 | 0.63431800 | 2.56714300 |
| H | -2.59493800 | 0.64576000 | 0.42705600 |
| C | -2.42904600 | -0.11244900 | 3.73389500 |
| H | -2.00303800 | -2.06960400 | 4.54113400 |
| H | -2.80702800 | 1.69848000 | 2.61720600 |

| | | | |
|---|---|---|---|
| **H** | **-2.51721400** | **0.36305500** | **4.70736700** |

ⅣB

0 1(charge, spin multiplicity)

| | | | |
|---|---|---|---|
| C | 4.02539700 | -0.26969600 | 2.27129500 |
| C | 2.93386900 | 0.08998500 | 1.53990900 |
| N | 2.71875400 | -0.38348100 | 0.26630300 |
| C | 3.61853900 | -1.28114100 | -0.37466500 |
| C | 4.77835400 | -1.63276900 | 0.41733400 |
| C | 4.97147000 | -1.15399500 | 1.68103800 |
| O | 3.37968500 | -1.71203100 | -1.50540700 |
| C | 1.55591400 | 0.14225000 | -0.48642400 |
| C | 0.70807700 | -0.96020900 | -1.13126900 |
| H | 4.15706700 | 0.11966900 | 3.27337900 |
| H | 2.17405900 | 0.76261100 | 1.92309100 |
| H | 5.47968900 | -2.31018100 | -0.05697900 |
| H | 5.85314300 | -1.44925900 | 2.24397400 |
| H | 1.16630700 | -1.27499100 | -2.06567500 |
| H | 0.60672200 | -1.81667500 | -0.46369000 |
| O | 1.92478500 | 1.01268100 | -1.51539100 |
| C | 2.31636800 | 2.31982600 | -1.09162100 |
| H | 3.22468600 | 2.28584100 | -0.47684500 |
| H | 2.52404400 | 2.88209100 | -2.00339500 |
| H | 1.50743700 | 2.81294800 | -0.54061600 |
| H | 0.94408400 | 0.66190400 | 0.26277100 |
| C | -1.42089800 | 2.20278300 | -0.16565600 |
| O | -1.10960400 | 2.25143900 | -1.38220700 |
| O | -1.55682100 | 1.20269000 | 0.58360500 |
| C | -1.55775700 | 3.58523400 | 0.52972200 |
| F | -0.32536600 | 3.99984800 | 0.93950600 |

| | | | |
|---|---|---|---|
| F | -2.34619700 | 3.54865500 | 1.61918900 |
| F | -2.04239200 | 4.53293000 | -0.29353400 |
| N | -0.65429200 | -0.41067500 | -1.40810800 |
| H | -1.15832500 | -0.21528400 | -0.50881400 |
| H | -0.59825500 | 0.58558500 | -1.74987900 |
| C | -1.50398800 | -1.20281900 | -2.34991400 |
| H | -0.98990400 | -1.21790200 | -3.31503100 |
| H | -2.42600100 | -0.62710200 | -2.46599500 |
| C | -1.79524400 | -2.63066700 | -1.87123900 |
| H | -2.46374000 | -3.07204300 | -2.62195300 |
| H | -0.87593700 | -3.22584800 | -1.90034700 |
| C | -2.42145400 | -2.71992600 | -0.49165800 |
| C | -1.77572100 | -3.41689900 | 0.53822600 |
| C | -3.65750700 | -2.11490300 | -0.21742700 |
| C | -2.34523900 | -3.50722600 | 1.81047300 |
| H | -0.82421400 | -3.90602900 | 0.33959100 |
| C | -4.22741700 | -2.20096800 | 1.05237600 |
| H | -4.18424000 | -1.57706200 | -1.00261800 |
| C | -3.57216900 | -2.89699300 | 2.07106200 |
| H | -1.82925300 | -4.05466500 | 2.59457000 |
| H | -5.18425500 | -1.72437600 | 1.24630800 |
| H | -4.01666800 | -2.96247200 | 3.06017900 |

Benzedrine

0 1(charge, spin multiplicity)

| | | | |
|---|---|---|---|
| **C** | **-1.38456500** | **1.21686600** | **0.19140500** |
| **C** | **-0.68022200** | **0.03601700** | **0.46357600** |
| **C** | **-1.32906200** | **-1.18802500** | **0.24641200** |
| **C** | **-2.63924600** | **-1.23255000** | **-0.23229000** |
| **C** | **-3.32787000** | **-0.04759000** | **-0.50169600** |

| | | | |
|---|---|---|---|
| C | -2.69558800 | 1.17865400 | -0.28727400 |
| H | -0.90087200 | 2.17690900 | 0.36049300 |
| H | -0.80148100 | -2.11585600 | 0.45842600 |
| H | -3.12392100 | -2.19267800 | -0.39052300 |
| H | -4.34943600 | -0.07978900 | -0.87110900 |
| H | -3.22433700 | 2.10704600 | -0.48821200 |
| C | 0.75507400 | 0.07830900 | 0.94449600 |
| H | 0.93009100 | 1.01073500 | 1.49731500 |
| H | 0.93053100 | -0.74235400 | 1.65308600 |
| C | 1.77781100 | -0.02759800 | -0.20368700 |
| H | 1.62402700 | -0.96185300 | -0.75733400 |
| H | 1.60146700 | 0.78943600 | -0.91851700 |
| C | 3.22661000 | 0.02624700 | 0.28480600 |
| H | 3.37909500 | 0.95857300 | 0.85989000 |
| H | 3.40364900 | -0.80169300 | 0.98402400 |
| N | 4.15915900 | -0.13499800 | -0.83967800 |
| H | 4.08297000 | 0.67541400 | -1.45479100 |
| H | 5.11645000 | -0.12087600 | -0.48948600 |

V A(C2)

0 1(charge, spin multiplicity)

| | | | |
|---|---|---|---|
| C | 4.61389100 | -1.92581700 | 1.90564900 |
| C | 4.03607600 | -0.72102400 | 1.58254000 |
| N | 3.57944000 | -0.52368100 | 0.32331800 |
| C | 3.66472100 | -1.46304800 | -0.64003800 |
| C | 4.25728000 | -2.70238700 | -0.37398400 |
| C | 4.72389500 | -2.92042700 | 0.90974700 |
| O | 3.18844500 | -1.06745100 | -1.79968100 |
| C | 2.93994000 | 0.72001500 | -0.20052600 |
| C | 2.59337300 | 0.26682500 | -1.63493500 |

| | | | |
|---|---|---|---|
| **H** | **4.97839600** | **-2.09565200** | **2.91109900** |
| **H** | **3.91908300** | **0.10759800** | **2.26953200** |
| **H** | **4.32674100** | **-3.44134800** | **-1.16209300** |
| **H** | **5.18363600** | **-3.87278200** | **1.15427300** |
| **H** | **3.04289700** | **0.92096200** | **-2.38094100** |
| **H** | **1.52097000** | **0.18332900** | **-1.78519700** |
| **O** | **3.88657200** | **1.73989800** | **-0.12754800** |
| **C** | **3.35773200** | **3.01460700** | **0.27995200** |
| **H** | **2.97761900** | **2.96761600** | **1.30843000** |
| **H** | **4.19782500** | **3.70960500** | **0.23349600** |
| **H** | **2.55399200** | **3.33689100** | **-0.38702000** |
| **H** | **2.02984500** | **0.92849200** | **0.37308200** |
| **C** | **-0.19936300** | **2.07145900** | **-0.19515700** |
| **O** | **0.49497200** | **2.31697500** | **-1.20327800** |
| **O** | **-0.07346700** | **1.17424300** | **0.67802600** |
| **C** | **-1.36610800** | **3.07673800** | **0.05388000** |
| **F** | **-2.31787500** | **2.59548600** | **0.88734500** |
| **F** | **-1.98669600** | **3.43575700** | **-1.08959000** |
| **F** | **-0.88797900** | **4.21444300** | **0.62283000** |
| **C** | **-2.40974400** | **-1.34088200** | **-0.59840500** |
| **H** | **-2.33144800** | **-1.21148600** | **-1.68831500** |
| **H** | **-1.89661100** | **-0.48528000** | **-0.14611200** |
| **C** | **-3.86849500** | **-1.31135800** | **-0.18640900** |
| **C** | **-4.33629000** | **-0.33897200** | **0.70802600** |
| **C** | **-4.78555400** | **-2.24934100** | **-0.68545700** |
| **C** | **-5.67681900** | **-0.30916300** | **1.10048200** |
| **H** | **-3.64504100** | **0.41241300** | **1.08116500** |
| **C** | **-6.12482900** | **-2.22397800** | **-0.29551500** |
| **H** | **-4.44952300** | **-3.00559700** | **-1.39197200** |
| **C** | **-6.57584300** | **-1.25387100** | **0.60342800** |

| | | | |
|---|---|---|---|
| H | -6.01789400 | 0.45670500 | 1.79273000 |
| H | -6.81815400 | -2.95954000 | -0.69640500 |
| H | -7.61922800 | -1.23199600 | 0.90750200 |
| C | -1.67761700 | -2.64188400 | -0.21385900 |
| H | -2.14110700 | -3.49031800 | -0.73681900 |
| H | -1.80777600 | -2.83421400 | 0.86092400 |
| C | -0.17296200 | -2.61759100 | -0.54145600 |
| H | -0.03486100 | -2.31231200 | -1.58839700 |
| H | 0.22682100 | -3.63751400 | -0.46497700 |
| N | 0.66566300 | -1.74694300 | 0.29596600 |
| H | 0.29901100 | -0.78920800 | 0.30350400 |
| H | 0.59016400 | -2.06189100 | 1.26434000 |

TSⅤ2

0 1(charge, spin multiplicity)

| | | | |
|---|---|---|---|
| C | 3.61984200 | -3.73239300 | 0.39207200 |
| C | 4.07289900 | -2.45037800 | 0.27616100 |
| N | 3.66561300 | -1.68515800 | -0.78446100 |
| C | 2.57356900 | -2.05917100 | -1.55992300 |
| C | 2.33687900 | -3.48890800 | -1.66784500 |
| C | 2.81461900 | -4.27711800 | -0.66430000 |
| O | 2.50328900 | -1.27092200 | -2.65370800 |
| C | 4.30816200 | -0.47186000 | -1.33623800 |
| C | 3.23890500 | -0.05105600 | -2.35268200 |
| H | 3.92536500 | -4.34204500 | 1.23314300 |
| H | 4.75639400 | -1.97909000 | 0.97128700 |
| H | 1.76306100 | -3.86633300 | -2.50543500 |
| H | 2.61046500 | -5.34400600 | -0.67415600 |
| H | 3.68033700 | 0.30497800 | -3.28181600 |
| H | 2.55516200 | 0.68535900 | -1.92439400 |

| | | | |
|---|---|---|---|
| O | 5.49923500 | -0.75282400 | -2.01576700 |
| C | 6.66270700 | -0.85846900 | -1.19878500 |
| H | 6.67856100 | -1.79792800 | -0.63142300 |
| H | 7.51791300 | -0.84363500 | -1.87733400 |
| H | 6.73638800 | -0.01270600 | -0.50201700 |
| H | 4.46173900 | 0.25530800 | -0.53463600 |
| C | 2.49643600 | 1.43433700 | 0.80688800 |
| O | 1.44539000 | 1.23936200 | 0.13181600 |
| O | 3.15978400 | 0.64212700 | 1.49765700 |
| C | 3.10257100 | 2.85762200 | 0.63063000 |
| F | 3.87668200 | 3.23575000 | 1.66490800 |
| F | 2.17663900 | 3.81757600 | 0.44546600 |
| F | 3.90058100 | 2.85444500 | -0.48203400 |
| N | 1.07048400 | -1.45074800 | -0.52492300 |
| H | 1.01799200 | -2.10660200 | 0.25534700 |
| H | 1.27871100 | -0.50153100 | -0.13593000 |
| C | -0.19564500 | -1.40411600 | -1.28640600 |
| H | -0.15267900 | -2.15844900 | -2.08199300 |
| H | -0.22797500 | -0.42364300 | -1.76984100 |
| C | -1.46689300 | -1.62922000 | -0.45406600 |
| H | -2.31232000 | -1.45256400 | -1.13106500 |
| H | -1.52857100 | -2.68518600 | -0.15925000 |
| C | -1.61621000 | -0.73431200 | 0.80639700 |
| H | -2.68456400 | -0.53083800 | 0.95000600 |
| H | -1.13389800 | 0.23404800 | 0.63198700 |
| C | -1.07312300 | -1.35944900 | 2.08120000 |
| C | 0.11301300 | -0.90923400 | 2.67761500 |
| C | -1.76342500 | -2.41960700 | 2.69131800 |
| C | 0.59830000 | -1.50684900 | 3.84466500 |
| H | 0.66651100 | -0.09142600 | 2.22630600 |

| | | | |
|---|---|---|---|
| **C** | **-1.28330700** | **-3.01561700** | **3.85742300** |
| **H** | **-2.69365500** | **-2.77502900** | **2.25152100** |
| **C** | **-0.09645700** | **-2.56071300** | **4.43902100** |
| **H** | **1.52212900** | **-1.14068200** | **4.28459000** |
| **H** | **-1.83827800** | **-3.83050400** | **4.31579900** |
| **H** | **0.27854100** | **-3.02187400** | **5.34906500** |

Ⅴc

0 1(charge, spin multiplicity)

| | | | |
|---|---|---|---|
| **C** | **-2.61091500** | **3.99519900** | **-0.31517200** |
| **C** | **-2.91983300** | **2.75146000** | **0.16361400** |
| **N** | **-2.12142200** | **1.66547200** | **-0.06035000** |
| **C** | **-0.97200900** | **1.75660600** | **-0.82140200** |
| **C** | **-0.63103900** | **3.04810400** | **-1.32113200** |
| **C** | **-1.43005400** | **4.13722400** | **-1.07098100** |
| **O** | **-4.68774900** | **0.52718000** | **-0.58854000** |
| **C** | **-2.58545200** | **0.34885500** | **0.55200500** |
| **C** | **-3.57924800** | **-0.33563300** | **-0.37845100** |
| **H** | **-3.26716100** | **4.83211100** | **-0.11120000** |
| **H** | **-3.79376100** | **2.54008700** | **0.76183300** |
| **H** | **0.26093900** | **3.15125800** | **-1.92248200** |
| **H** | **-1.15018300** | **5.10721000** | **-1.47109400** |
| **H** | **-3.87234800** | **-1.27387900** | **0.11137100** |
| **H** | **-3.06668600** | **-0.59099200** | **-1.31370800** |
| **O** | **-3.19054800** | **0.59990100** | **1.77863500** |
| **C** | **-2.26701500** | **0.71050300** | **2.87034400** |
| **H** | **-1.65108000** | **1.61545700** | **2.78148500** |
| **H** | **-2.87310200** | **0.77852500** | **3.77544900** |
| **H** | **-1.62107900** | **-0.17232300** | **2.91344800** |
| **H** | **-1.70530200** | **-0.28598200** | **0.67894900** |

| | | | |
|---|---|---|---|
| C | -0.60604100 | -2.52993400 | 0.14827300 |
| O | -0.92668700 | -1.98350400 | -0.94777000 |
| O | -0.61369800 | -2.06155400 | 1.29912000 |
| C | -0.05752600 | -3.97560400 | -0.02165100 |
| F | 0.06245100 | -4.63641600 | 1.14402700 |
| F | 1.18022600 | -3.93128700 | -0.58701100 |
| F | -0.83578300 | -4.72498900 | -0.83294700 |
| N | -0.22023200 | 0.67340800 | -1.05509600 |
| H | -0.58982300 | -0.29484300 | -0.92072000 |
| H | -5.27702200 | 0.09999300 | -1.22797500 |
| C | 1.04076200 | 0.74168300 | -1.80924600 |
| H | 1.62085300 | 1.59933200 | -1.45290400 |
| H | 0.82616000 | 0.90364200 | -2.87517400 |
| C | 1.87436300 | -0.53241800 | -1.63970700 |
| H | 2.69888700 | -0.47018800 | -2.36090900 |
| H | 1.27104300 | -1.40401000 | -1.91040200 |
| C | 2.44922400 | -0.74445400 | -0.22063200 |
| H | 1.62919200 | -0.81390600 | 0.50195900 |
| H | 2.94460000 | -1.72299500 | -0.21006700 |
| C | 3.42565600 | 0.33010500 | 0.21159500 |
| C | 3.06830900 | 1.29419700 | 1.16429300 |
| C | 4.71372400 | 0.39523400 | -0.34280800 |
| C | 3.96451700 | 2.29426400 | 1.55103600 |
| H | 2.07924000 | 1.25238900 | 1.61518500 |
| C | 5.61278900 | 1.39097100 | 0.03918000 |
| H | 5.01715900 | -0.35012300 | -1.07539600 |
| C | 5.24032900 | 2.34683900 | 0.98836300 |
| H | 3.66637800 | 3.02777600 | 2.29606300 |
| H | 6.60743900 | 1.41754500 | -0.39880600 |
| H | 5.94139200 | 3.12060700 | 1.28991600 |

VD

0 1(charge, spin multiplicity)

| | | | |
|---|---|---|---|
| **C** | **-4.61241700** | **-1.18937300** | **-0.00107400** |
| **C** | **-4.38942700** | **-0.13986100** | **-0.89024400** |
| **N** | **-3.28607200** | **0.61504900** | **-0.90373500** |
| **C** | **-2.32121000** | **0.34132400** | **-0.00175100** |
| **C** | **-2.45278900** | **-0.69345900** | **0.95084700** |
| **C** | **-3.61170700** | **-1.45835600** | **0.93731400** |
| **H** | **-5.53145600** | **-1.76484400** | **-0.03804700** |
| **H** | **-5.14137400** | **0.11652000** | **-1.63609600** |
| **H** | **-1.66955500** | **-0.88852100** | **1.67461600** |
| **H** | **-3.73468600** | **-2.26072900** | **1.66052600** |
| **N** | **-1.19162000** | **1.12628100** | **-0.09604600** |
| **H** | **-1.34627700** | **1.95239500** | **-0.66273900** |
| **C** | **-0.20835700** | **1.25037100** | **0.97100500** |
| **H** | **0.20917700** | **0.25943500** | **1.18378900** |
| **H** | **-0.67086800** | **1.61253600** | **1.90432500** |
| **C** | **0.92428600** | **2.19827000** | **0.56727200** |
| **H** | **1.58677700** | **2.31934500** | **1.43307800** |
| **H** | **0.50376300** | **3.19219600** | **0.35782600** |
| **C** | **1.75376700** | **1.73991500** | **-0.65412800** |
| **H** | **1.09099100** | **1.63082600** | **-1.52001300** |
| **H** | **2.46263600** | **2.54065500** | **-0.89928200** |
| **C** | **2.51202100** | **0.44784900** | **-0.42966200** |
| **C** | **1.98459300** | **-0.78332400** | **-0.84440000** |
| **C** | **3.75551800** | **0.45272900** | **0.21967100** |
| **C** | **2.67683800** | **-1.97481300** | **-0.61595700** |
| **H** | **1.02297600** | **-0.80455400** | **-1.35174100** |
| **C** | **4.45128300** | **-0.73480100** | **0.44960200** |

| | | | |
|---|---|---|---|
| **H** | **4.18612300** | **1.39921700** | **0.54106600** |
| **C** | **3.91309700** | **-1.95452500** | **0.03217000** |
| **H** | **2.25088300** | **-2.91791200** | **-0.94882400** |
| **H** | **5.41641100** | **-0.70751000** | **0.94919400** |
| **H** | **4.45535000** | **-2.88004800** | **0.20679000** |

Ⅴ**A**(**C8**)

0 1(charge, spin multiplicity)

| | | | |
|---|---|---|---|
| **C** | **-4.11586900** | **0.31416700** | **-2.51442600** |
| **C** | **-3.47203300** | **0.52523800** | **-1.31226200** |
| **N** | **-3.55590800** | **-0.44066600** | **-0.36207800** |
| **C** | **-4.21888600** | **-1.60397200** | **-0.54758000** |
| **C** | **-4.89361300** | **-1.85941300** | **-1.74048000** |
| **C** | **-4.83327800** | **-0.87828100** | **-2.72090500** |
| **O** | **-4.13708400** | **-2.42016000** | **0.49108000** |
| **C** | **-3.04629600** | **-0.35963500** | **1.05672200** |
| **C** | **-3.20722200** | **-1.83083000** | **1.46277000** |
| **H** | **-4.05205100** | **1.06920300** | **-3.28851900** |
| **H** | **-2.85715800** | **1.39794900** | **-1.06755500** |
| **H** | **-5.42239400** | **-2.79507000** | **-1.87112600** |
| **H** | **-5.34083800** | **-1.04293400** | **-3.66638000** |
| **H** | **-3.65735400** | **-1.93030200** | **2.44918700** |
| **H** | **-2.24899900** | **-2.35641300** | **1.38646100** |
| **O** | **-3.86316000** | **0.47196100** | **1.80741200** |
| **C** | **-3.29376800** | **1.74343900** | **2.17338500** |
| **H** | **-2.90004100** | **2.27940900** | **1.30454700** |
| **H** | **-4.10686500** | **2.30982700** | **2.63052700** |
| **H** | **-2.49143400** | **1.60304400** | **2.90804500** |
| **H** | **-2.00309900** | **-0.03543500** | **1.00317400** |
| **C** | **-0.45896600** | **1.88204400** | **-0.27667600** |

| | | | |
|---|---|---|---|
| O | -0.41080400 | 0.62912800 | -0.25473100 |
| O | -1.42940800 | 2.64357800 | -0.48880600 |
| C | 0.90162200 | 2.57708500 | 0.03788200 |
| F | 0.83619100 | 3.92012100 | 0.03696700 |
| F | 1.85396600 | 2.22257800 | -0.86571300 |
| F | 1.36401200 | 2.19880800 | 1.25797000 |
| N | 0.07435000 | -2.14477500 | 0.95980100 |
| H | 0.41198700 | -3.05779000 | 0.65295800 |
| H | 0.00228500 | -1.55624700 | 0.12977400 |
| C | 1.06130500 | -1.52116900 | 1.85852800 |
| H | 0.94967500 | -1.95793700 | 2.86044700 |
| H | 0.78875100 | -0.46327100 | 1.93007800 |
| C | 2.53654700 | -1.62724700 | 1.43199100 |
| H | 3.13934800 | -1.03423400 | 2.13334500 |
| H | 2.88244600 | -2.66667900 | 1.53596100 |
| C | 2.80206200 | -1.14059300 | -0.00892700 |
| H | 2.36177200 | -1.85916900 | -0.71317500 |
| H | 2.27880900 | -0.19344900 | -0.17138900 |
| C | 4.27440300 | -0.96915700 | -0.31472400 |
| C | 4.84593600 | 0.30993000 | -0.37016600 |
| C | 5.10689700 | -2.07821200 | -0.52718300 |
| C | 6.20914500 | 0.47645700 | -0.62412700 |
| H | 4.20849800 | 1.17889800 | -0.22621700 |
| C | 6.46970800 | -1.91683900 | -0.78035200 |
| H | 4.68075400 | -3.07939900 | -0.49807400 |
| C | 7.02698200 | -0.63648100 | -0.82820800 |
| H | 6.63088600 | 1.47768100 | -0.66710100 |
| H | 7.09631600 | -2.79012900 | -0.94511100 |
| H | 8.08769700 | -0.50826800 | -1.02813100 |

TSⅤ1

0 1(charge, spin multiplicity)

| | | | |
|---|---|---|---|
| **C** | **5.52301600** | **-0.51291200** | **-3.17264700** |
| **C** | **4.42500100** | **-0.33469700** | **-2.37441600** |
| **N** | **3.17865700** | **-0.59739100** | **-2.86088500** |
| **C** | **2.95154100** | **-1.04933800** | **-4.13503900** |
| **C** | **4.07168500** | **-1.23307100** | **-4.98713600** |
| **C** | **5.33099500** | **-0.96401000** | **-4.50238000** |
| **O** | **1.73091400** | **-1.27739900** | **-4.42460500** |
| **C** | **1.90350300** | **-0.30387200** | **-2.11509700** |
| **C** | **0.79832000** | **-1.02742300** | **-2.87621300** |
| **H** | **6.51303000** | **-0.31036000** | **-2.78337500** |
| **H** | **4.47601400** | **0.00658700** | **-1.34767900** |
| **H** | **3.89513100** | **-1.58604800** | **-5.99570600** |
| **H** | **6.19331900** | **-1.10549600** | **-5.14693300** |
| **H** | **0.04350400** | **-0.43188100** | **-3.36154500** |
| **H** | **0.63925300** | **-2.08580700** | **-2.74336500** |
| **O** | **1.61852500** | **1.04005300** | **-2.10360600** |
| **C** | **2.15391400** | **1.83394500** | **-1.03150900** |
| **H** | **3.09642200** | **2.30375700** | **-1.33794400** |
| **H** | **1.38785300** | **2.57986200** | **-0.80066300** |
| **H** | **2.32827900** | **1.21395200** | **-0.14125000** |
| **H** | **2.04168700** | **-0.71511200** | **-1.10932600** |
| **C** | **-1.54702000** | **2.83198400** | **-0.78180600** |
| **O** | **-1.67800800** | **1.69479100** | **-1.30631100** |
| **O** | **-0.55322400** | **3.40936300** | **-0.30838600** |
| **C** | **-2.89434700** | **3.61915400** | **-0.73504200** |
| **F** | **-2.79321400** | **4.82921700** | **-0.14784400** |
| **F** | **-3.84192500** | **2.92727700** | **-0.04718000** |
| **F** | **-3.39086800** | **3.82544100** | **-1.98138600** |

| | | | |
|---|---|---|---|
| N | -0.56066300 | -0.83447500 | -1.17750300 |
| H | -0.16687400 | -1.18491300 | -0.30304800 |
| H | -0.75104000 | 0.18354900 | -1.08637800 |
| C | -1.82868600 | -1.52112700 | -1.48474800 |
| H | -1.60514800 | -2.46843900 | -1.99688800 |
| H | -2.36322200 | -0.88316000 | -2.19566300 |
| C | -2.72341900 | -1.82246400 | -0.27317800 |
| H | -3.61622600 | -2.34569300 | -0.64162800 |
| H | -2.20954000 | -2.53543400 | 0.38938200 |
| C | -3.15749400 | -0.58927500 | 0.54702700 |
| H | -2.27374700 | -0.12031000 | 0.99477000 |
| H | -3.58389200 | 0.16738300 | -0.11941500 |
| C | -4.14352000 | -0.95474200 | 1.63635000 |
| C | -5.52231500 | -0.96828900 | 1.38097900 |
| C | -3.70508700 | -1.31794400 | 2.91752500 |
| C | -6.43445000 | -1.33624800 | 2.37130700 |
| H | -5.88315300 | -0.67767000 | 0.39655200 |
| C | -4.61218900 | -1.68745000 | 3.91243900 |
| H | -2.63982700 | -1.30187400 | 3.14014600 |
| C | -5.98198300 | -1.69897300 | 3.64197100 |
| H | -7.49938900 | -1.33259600 | 2.15262500 |
| H | -4.24942800 | -1.95934500 | 4.90069400 |
| H | -6.69088100 | -1.98097600 | 4.41607500 |

VB

0 1(charge, spin multiplicity)

| | | | |
|---|---|---|---|
| C | 5.88048800 | 1.09314000 | 0.45641100 |
| C | 4.53607800 | 0.92935200 | 0.31534100 |
| N | 3.97172400 | -0.29815500 | 0.05169200 |
| C | 4.75393800 | -1.48126400 | -0.09222400 |
| C | 6.18044100 | -1.27429900 | 0.04739300 |

| | | | |
|---|---|---|---|
| C | 6.71862400 | -0.04857600 | 0.31253900 |
| O | 4.21847800 | -2.57128300 | -0.30331000 |
| C | 2.51023900 | -0.36285100 | -0.16228600 |
| C | 1.81638200 | -1.40335500 | 0.72263000 |
| H | 6.28629900 | 2.07481700 | 0.66800500 |
| H | 3.83838800 | 1.75473000 | 0.40741600 |
| H | 6.79294200 | -2.16208500 | -0.06403600 |
| H | 7.79506000 | 0.06041600 | 0.41584600 |
| H | 2.05942800 | -2.40525100 | 0.37544200 |
| H | 2.10832200 | -1.28915400 | 1.76901700 |
| O | 2.16398500 | -0.67665400 | -1.48118000 |
| C | 2.25466500 | 0.41564800 | -2.40165400 |
| H | 3.27965600 | 0.80181800 | -2.46033000 |
| H | 1.96945600 | 0.01360700 | -3.37516300 |
| H | 1.55784400 | 1.21258600 | -2.11786200 |
| H | 2.13141900 | 0.63117000 | 0.10847800 |
| C | -0.69883200 | 1.71254000 | 0.21791800 |
| O | -0.70800300 | 1.10997900 | -0.87307800 |
| O | -0.26011000 | 1.31986000 | 1.33982000 |
| C | -1.34703300 | 3.12428800 | 0.20329300 |
| F | -1.12977700 | 3.82233000 | 1.33229200 |
| F | -2.69080000 | 3.01570100 | 0.04723300 |
| F | -0.88274800 | 3.86250800 | -0.82715900 |
| N | 0.34565800 | -1.17190600 | 0.61822300 |
| H | 0.10187700 | -0.20805500 | 1.04228300 |
| H | 0.11599900 | -1.02863700 | -0.37467400 |
| C | -0.51059900 | -2.24670300 | 1.21395100 |
| H | -0.18874700 | -2.36261000 | 2.25277200 |
| H | -0.29053000 | -3.17763700 | 0.68135400 |
| C | -1.99642700 | -1.88781700 | 1.14447000 |
| H | -2.53924600 | -2.65199300 | 1.71371500 |
| H | -2.16221500 | -0.93608300 | 1.66348300 |
| C | -2.57196300 | -1.80899500 | -0.28629800 |
| H | -2.03698300 | -1.04201800 | -0.85752900 |
| H | -2.40840400 | -2.77093500 | -0.79059800 |
| C | -4.04909800 | -1.47079600 | -0.28641700 |
| C | -5.01882400 | -2.47839800 | -0.19393800 |
| C | -4.47215600 | -0.13535100 | -0.34712300 |
| C | -6.37835500 | -2.16340500 | -0.15963800 |

| | | | |
|---|---|---|---|
| H | -4.70613500 | -3.52024300 | -0.15533400 |
| C | -5.83110900 | 0.18242400 | -0.31127800 |
| H | -3.73454400 | 0.65926700 | -0.42820300 |
| C | -6.78856800 | -0.82980800 | -0.21701700 |
| H | -7.11608200 | -2.95906700 | -0.09325500 |
| H | -6.14123600 | 1.22290100 | -0.36263500 |
| H | -7.84645300 | -0.58183000 | -0.19336800 |

**References for quantum chemistry calculations:**

1. Gaussian 09, Revision D.01, M. J. Frisch, G. W. Trucks, H. B. Schlegel, G. E. Scuseria, M. A. Robb, J. R. Cheeseman, G. Scalmani, V. Barone, B. Mennucci, G. A. Petersson, H. Nakatsuji, M. Caricato, X. Li, H. P. Hratchian, A. F. Izmaylov, J. Bloino, G. Zheng, J. L. Sonnenberg, M. Hada, M. Ehara, K. Toyota, R. Fukuda, J. Hasegawa, M. Ishida, T. Nakajima, Y. Honda, O. Kitao, H. Nakai, T. Vreven, J. A. Montgomery, Jr., J. E. Peralta, F. Ogliaro, M. Bearpark, J. J. Heyd, E. Brothers, K. N. Kudin, V. N. Staroverov, R. Kobayashi, J. Normand, K. Raghavachari, A. Rendell, J. C. Burant, S. S. Iyengar, J. Tomasi, M. Cossi, N. Rega, J. M. Millam, M. Klene, J. E. Knox, J. B. Cross, V. Bakken, C. Adamo, J. Jaramillo, R. Gomperts, R. E. Stratmann, O. Yazyev, A. J. Austin, R. Cammi, C. Pomelli, J. W. Ochterski, R. L. Martin, K. Morokuma, V. G. Zakrzewski, G. A. Voth, P. Salvador, J. J. Dannenberg, S. Dapprich, A. D. Daniels, Ö. Farkas, J. B. Foresman, J. V. Ortiz, J. Cioslowski, and D. J. Fox, Gaussian, Inc., Wallingford CT, 2009.2. Becke, A. D. *J. Chem. Phys.* **1993**, *98*, 5648.

2. Wadt, W. R.; Hay, P. J. *J. Chem. Phys.* **1985**, *82*, 299.

3. Hratchian, H. P.; Schlegel, H. B. in *Theory and Applications of Computational Chemistry:The First 40 Years*, Ed. Dykstra, C. E.; Frenking, G.; Kim, K. S.; Scuseria G., Elsevier:Amsterdam, **2005**, 195–249.

4. Cossi, M.; Rega, N.; Scalmani, G.; Barone, V. J. *Comput. Chem.* **2003**, *24*, 669.

5. Tomasi, J.; Mennucci, B.; Cammi, R. *Chem. Rev.* **2005**, *105*, 2999.

# NMR spectra of products

### 1-(2-hydroxy-1-methoxyethyl)pyridin-2(1H)-one 5a

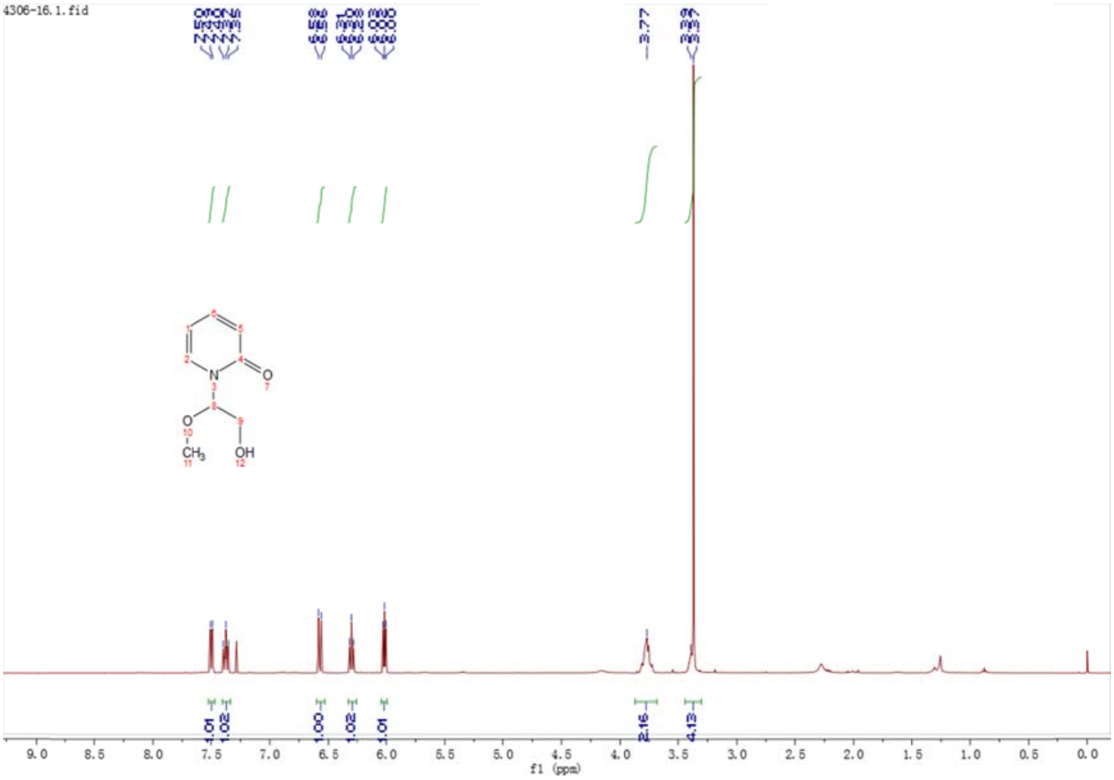

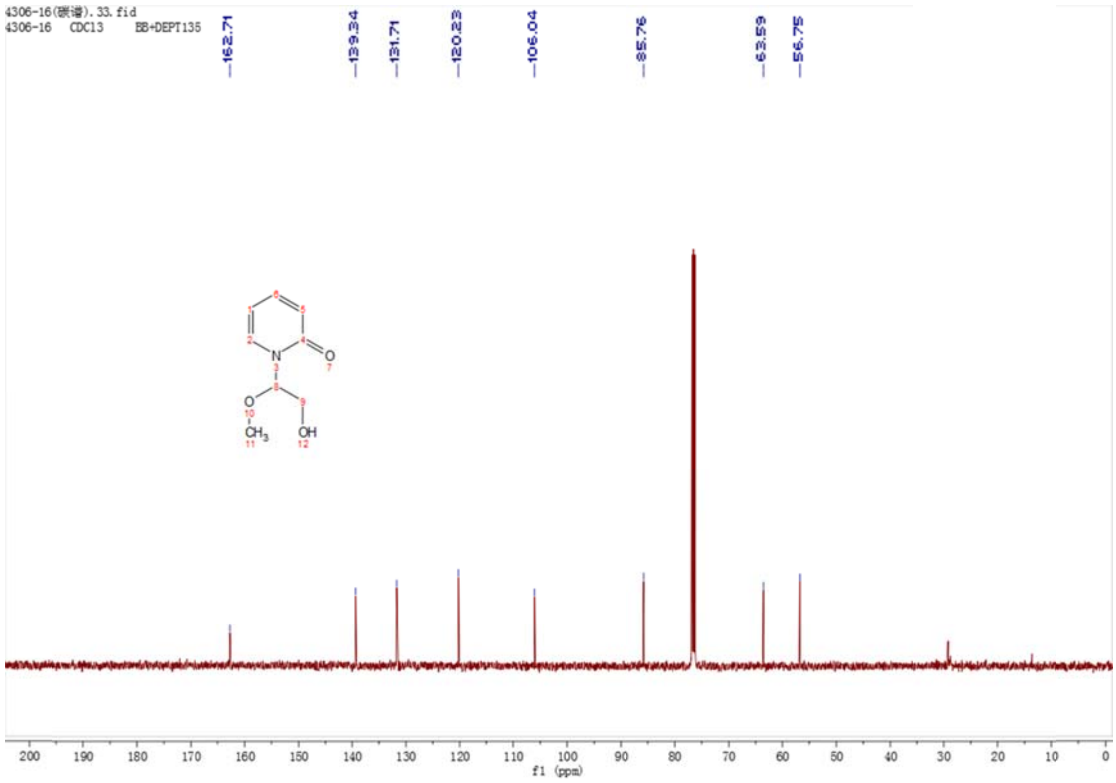


**1-(2-hydroxy-1-methoxyethyl)-3-methylpyridin-2(1H)-one 5b**

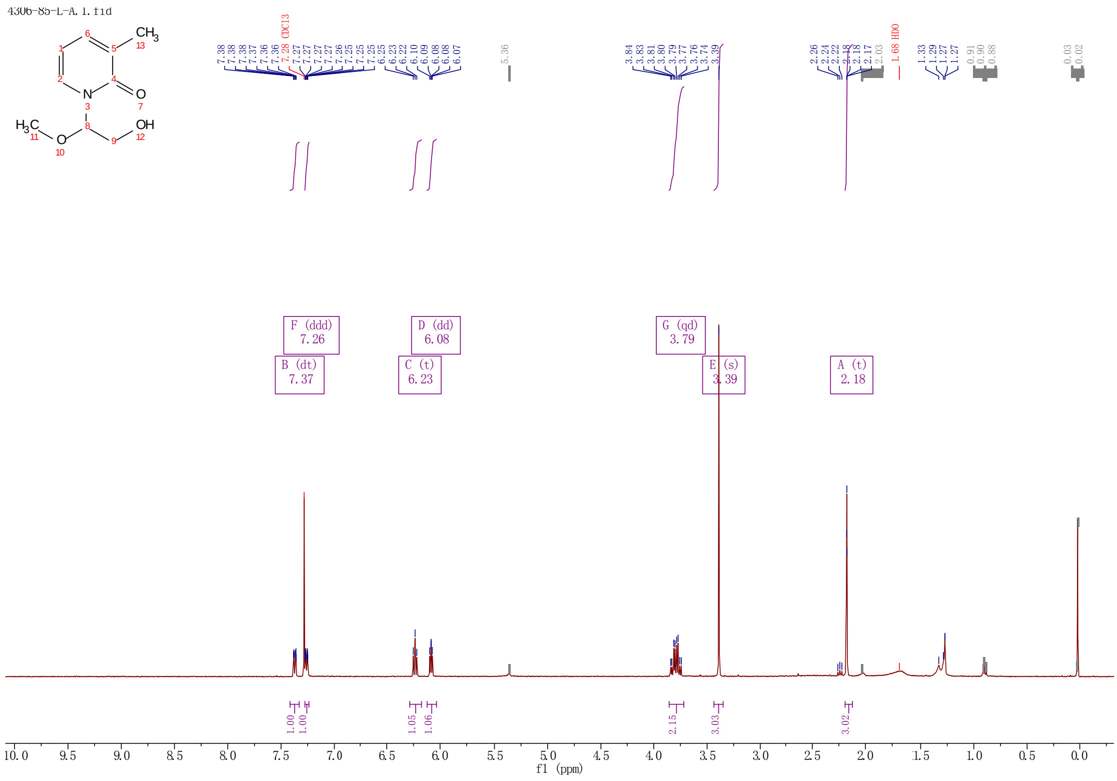

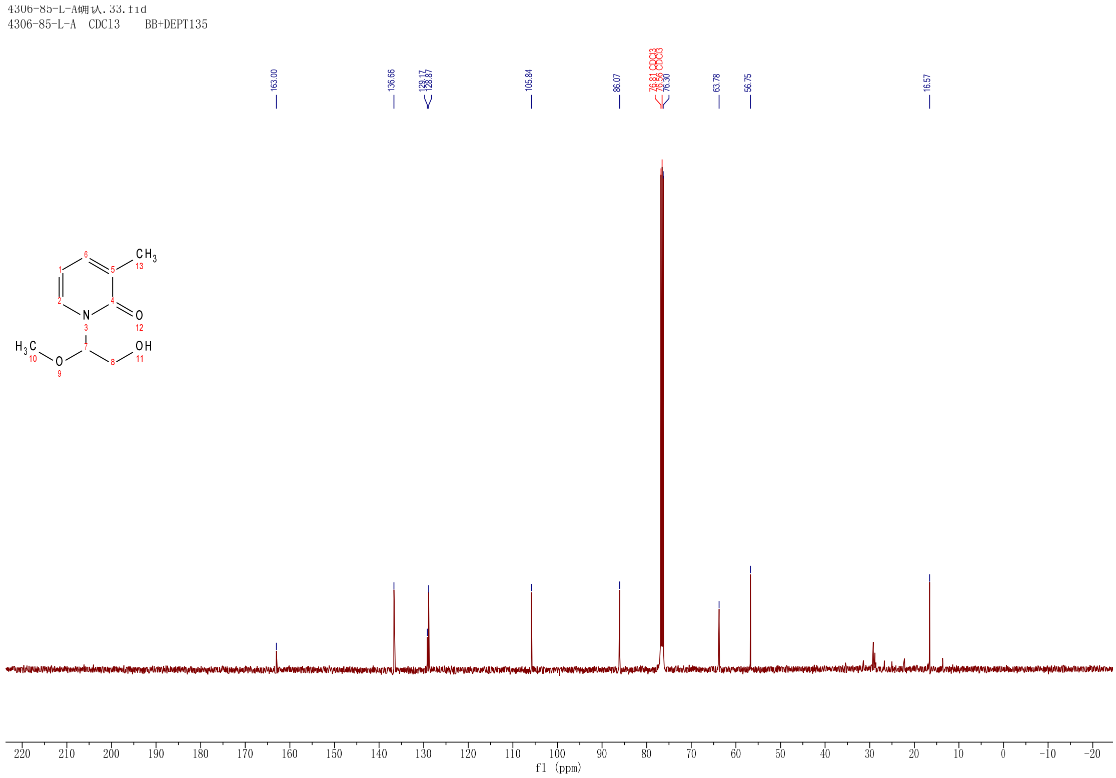


# 1-(2-hydroxy-1-methoxyethyl)-3-methoxypyridin-2(1H)-one 5c

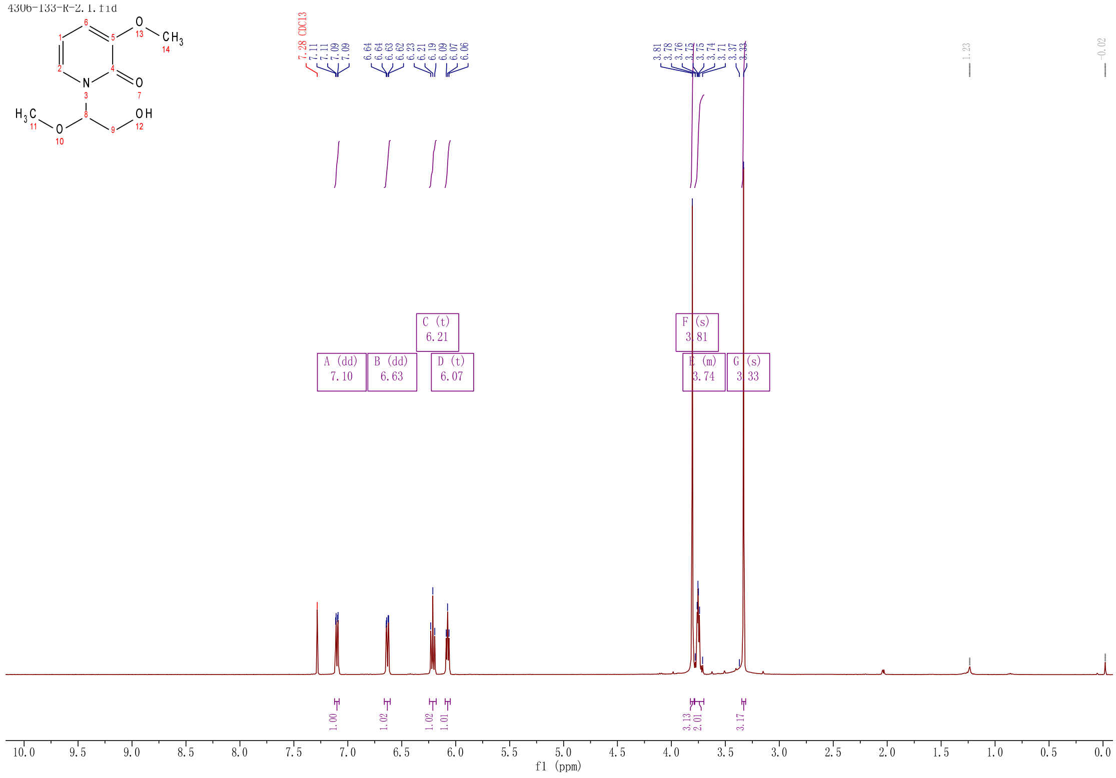

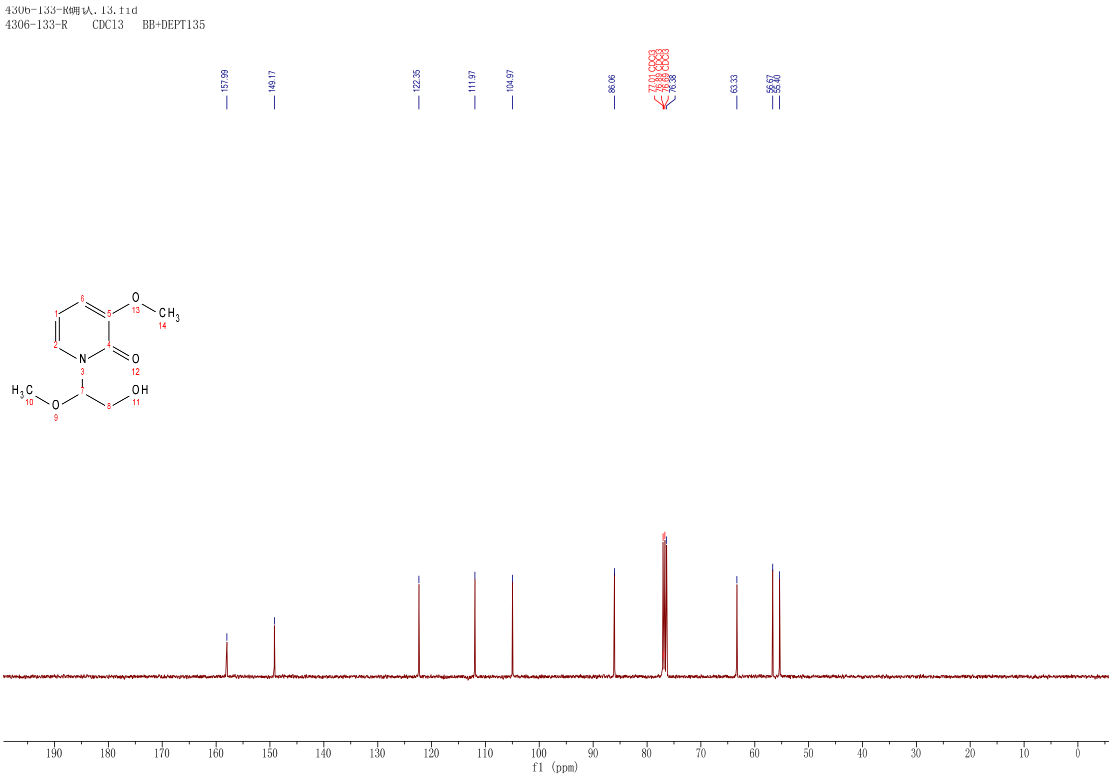


# 1-(2-hydroxy-1-methoxyethyl)-3-nitropyridin-2(1H)-one 5d

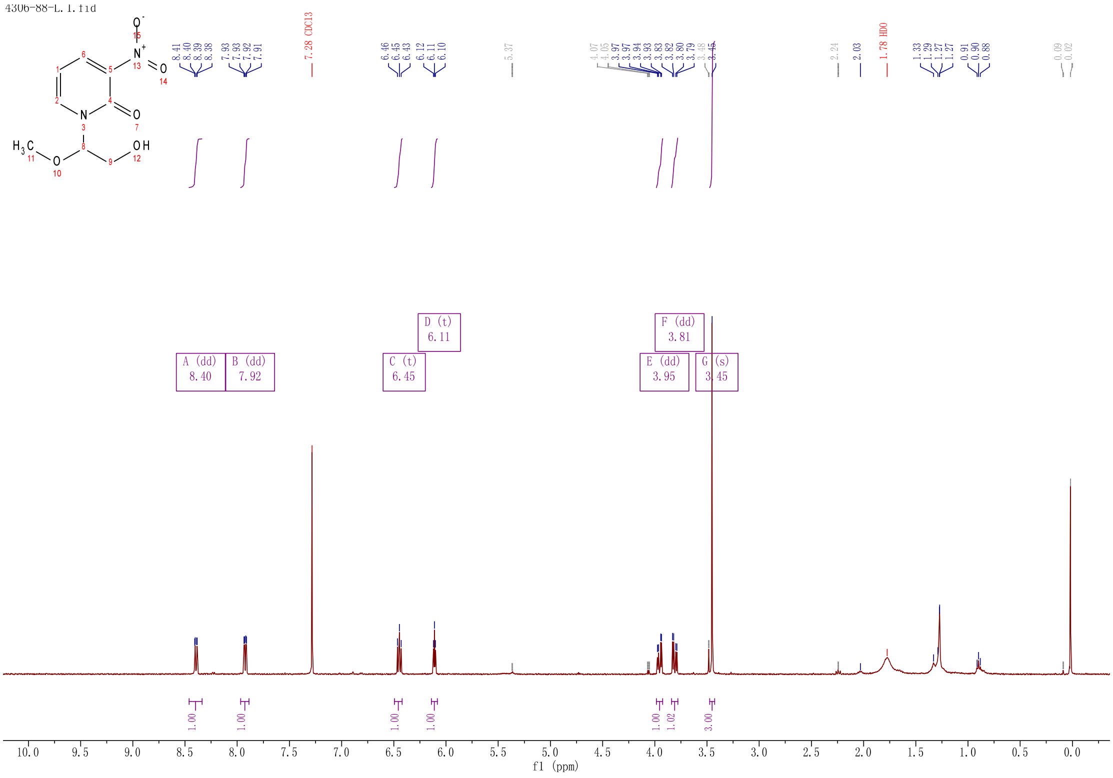

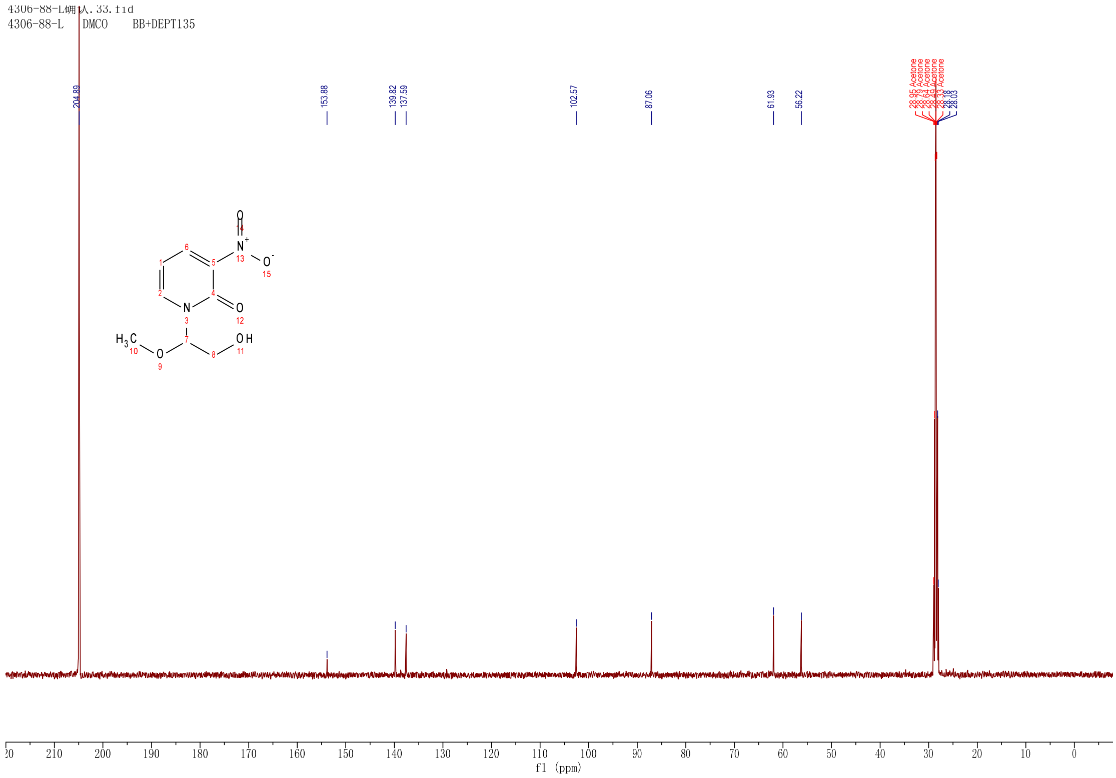

# 1-(2-hydroxy-1-methoxyethyl)-3-(trifluoromethyl)pyridin-2(1H)-one 5e

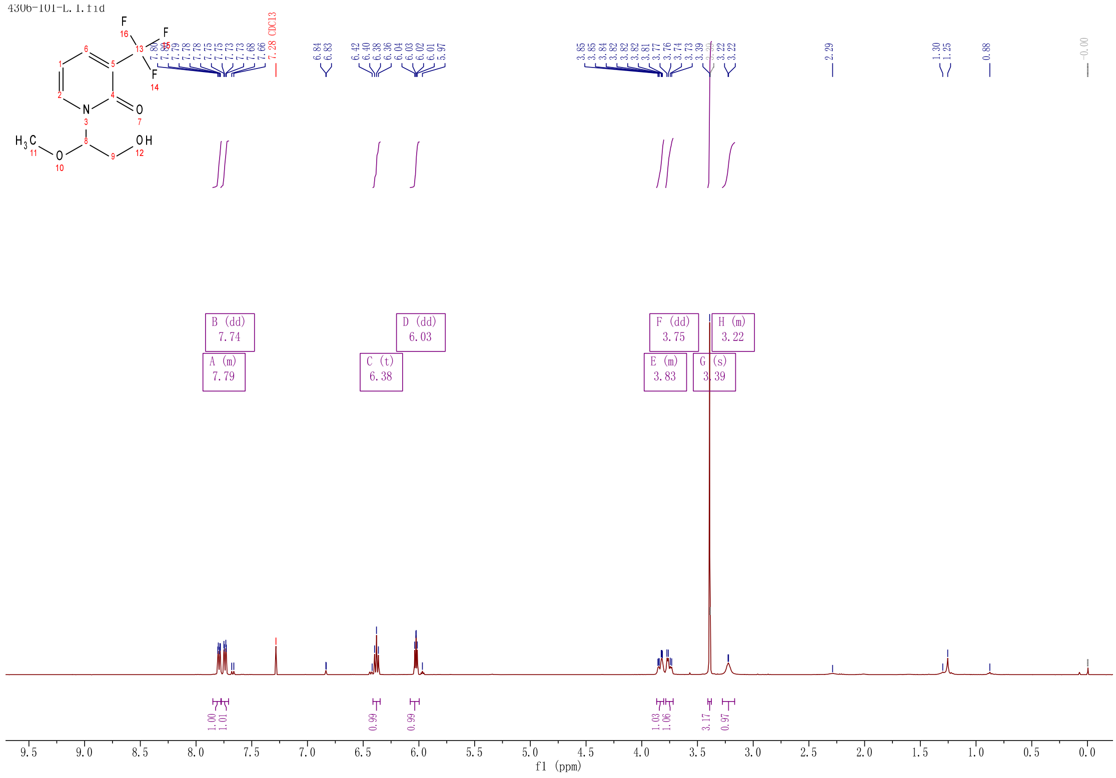

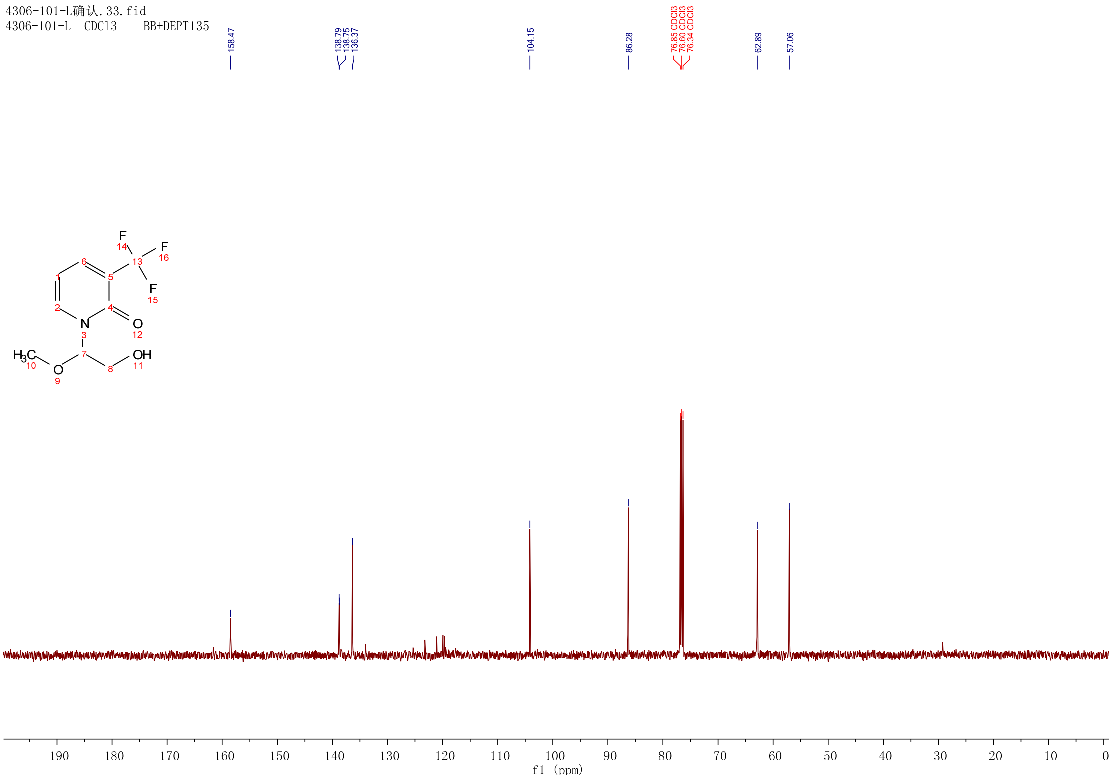


# 1-(2-hydroxy-1-methoxyethyl)-2-oxo-1,2-dihydropyridine-3-carboxamide 5f

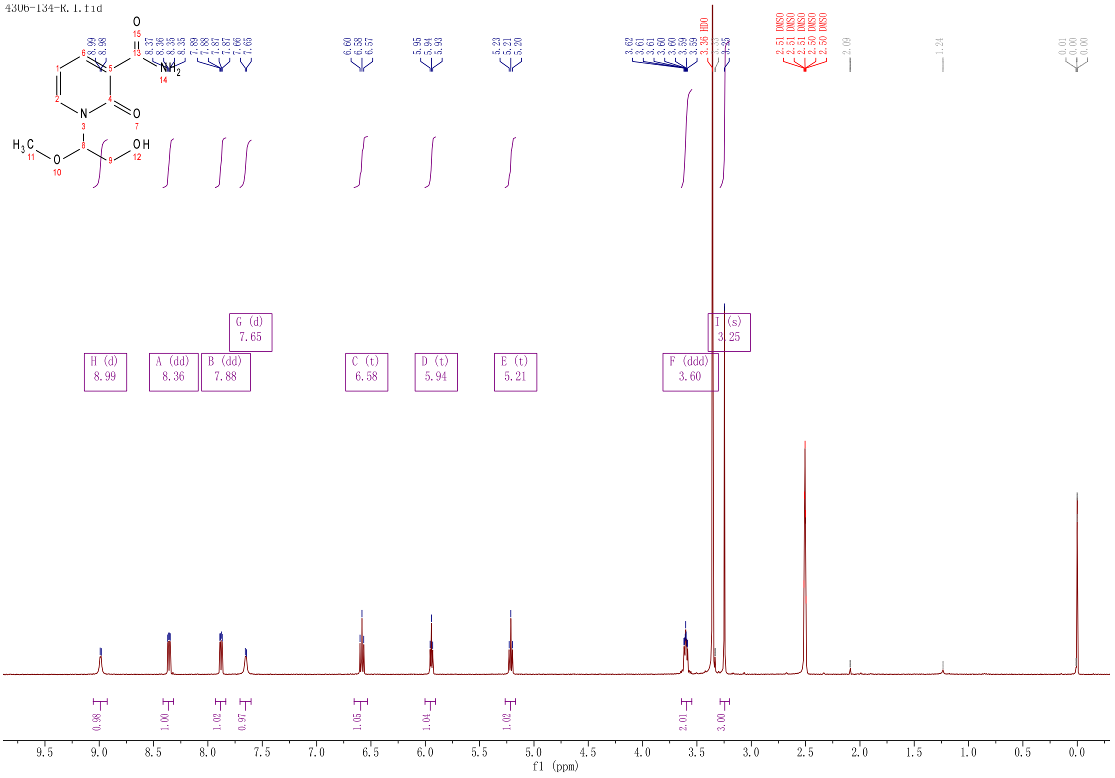

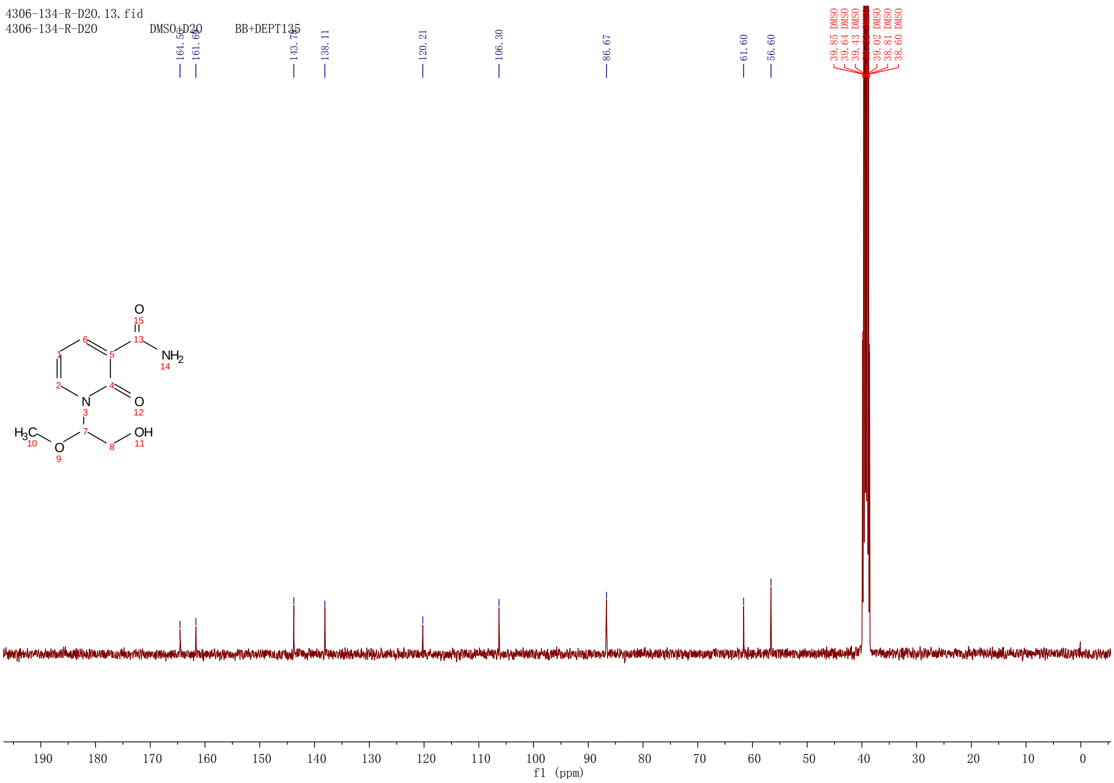


# 1-(2-hydroxy-1-methoxyethyl)-4-methylpyridin-2(1H)-one 5g

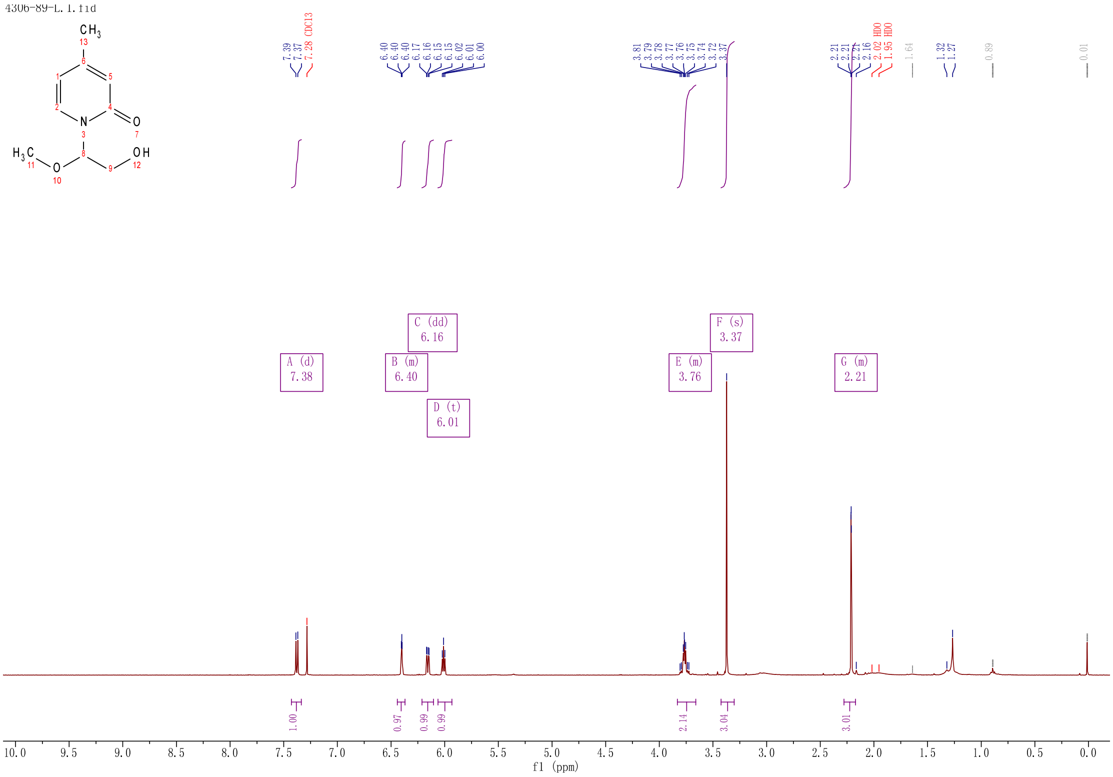

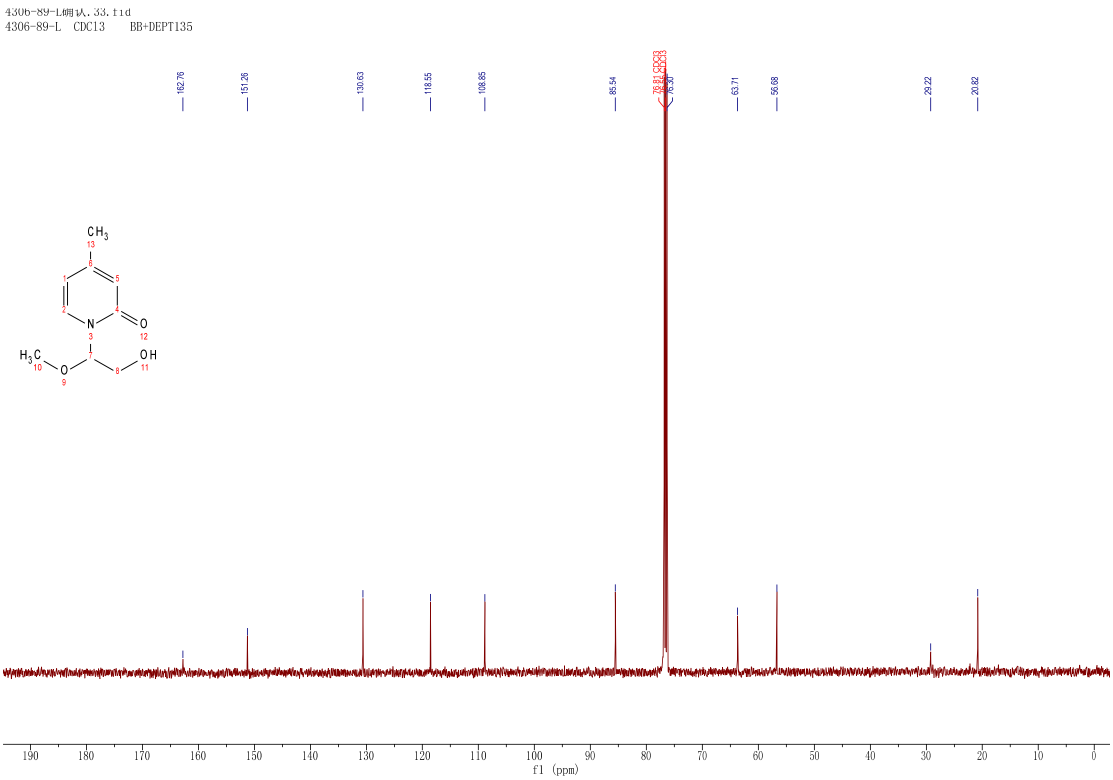


**1-(2-hydroxy-1-methoxyethyl)-4-(trifluoromethyl)pyridin-2(1H)-one 5h**

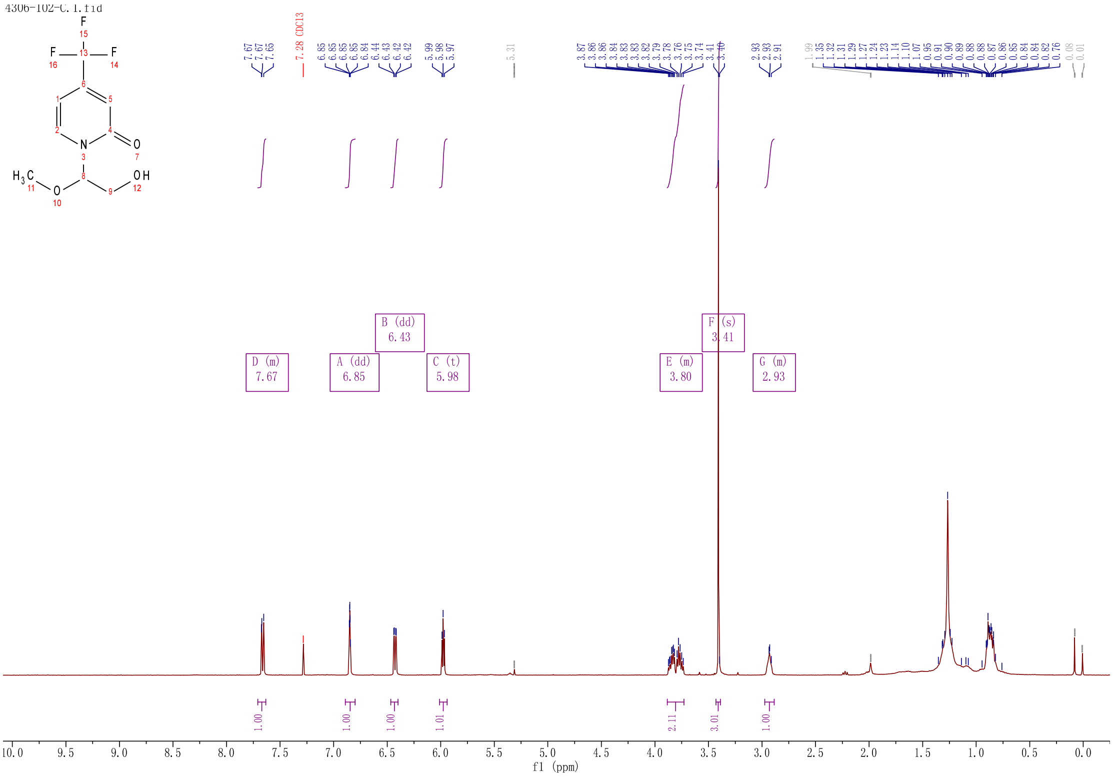

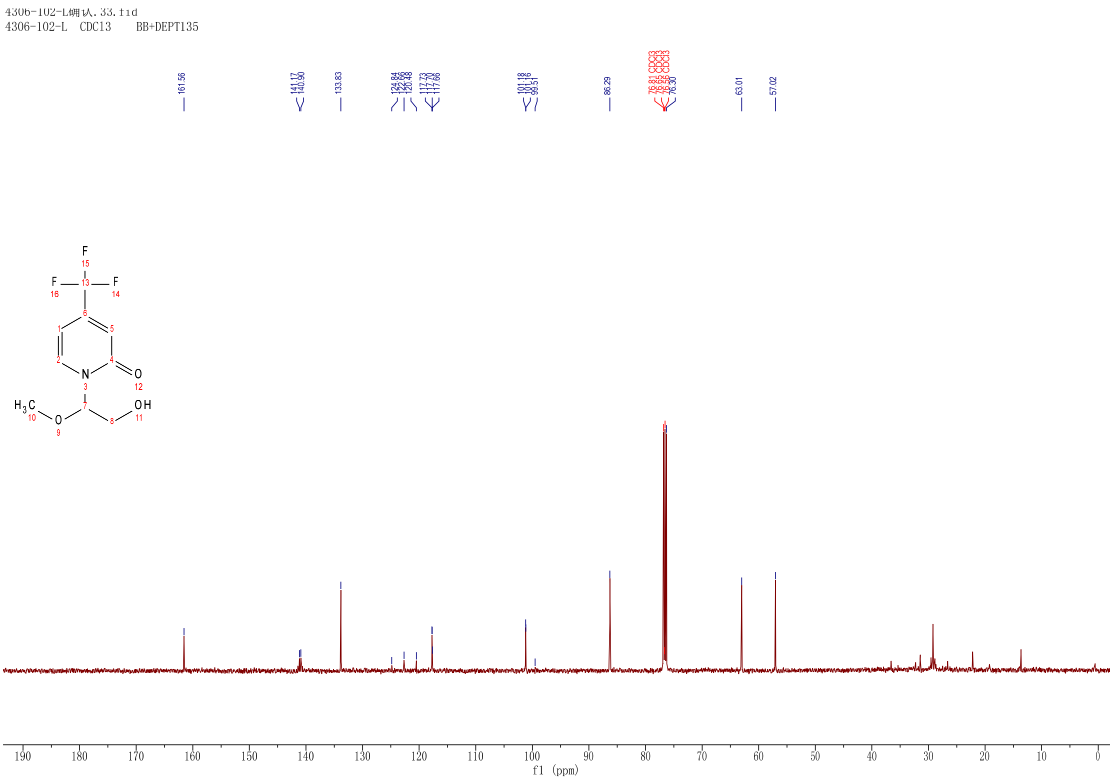

# 1-(2-hydroxy-1-methoxyethyl)-5-methylpyridin-2(1H)-one 5i

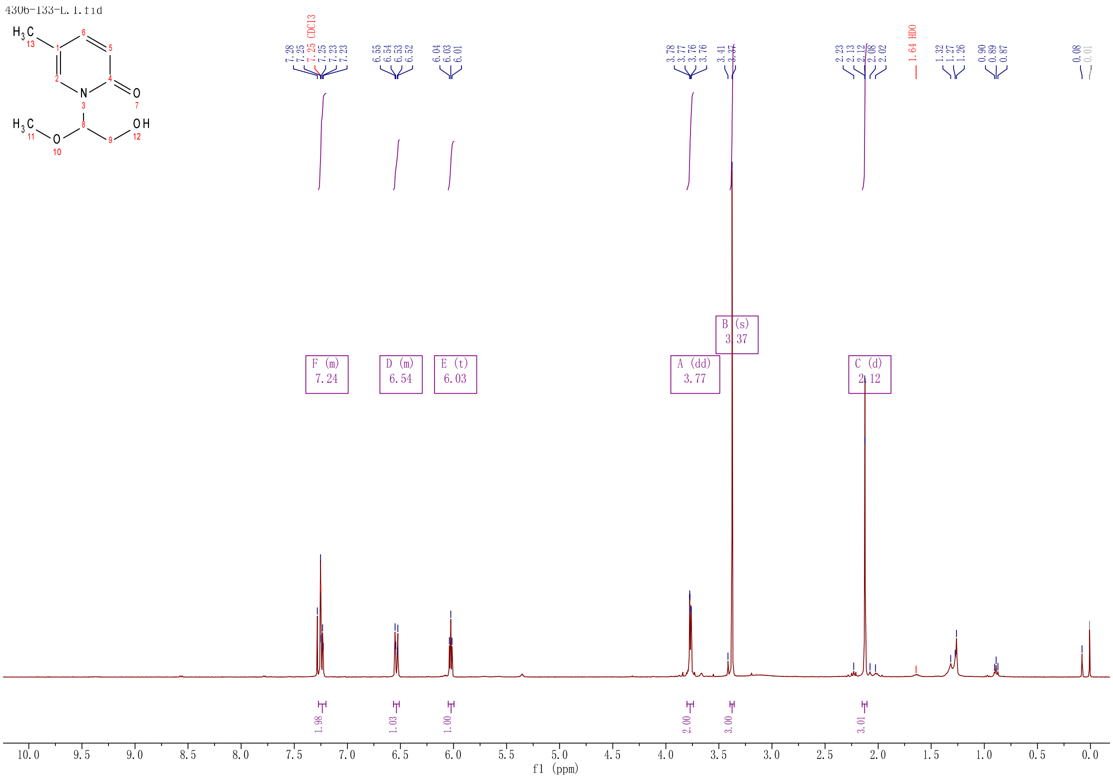

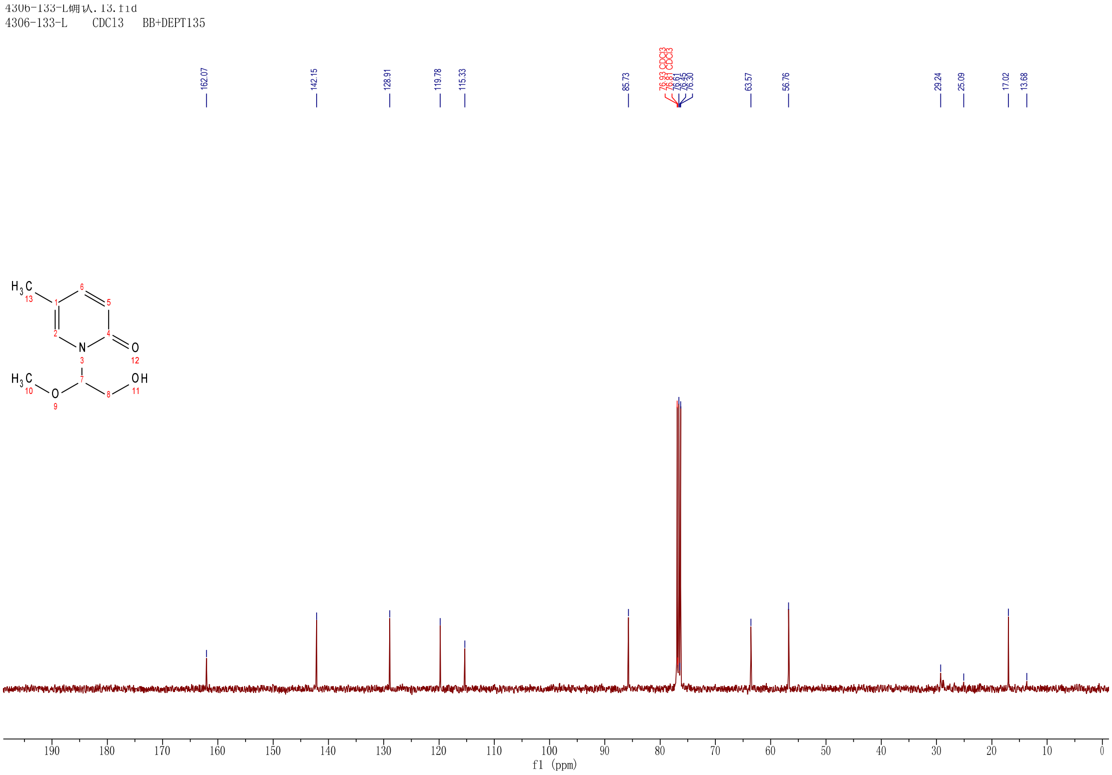


# 1-(2-hydroxy-1-methoxyethyl)-5-nitropyridin-2(1H)-one 5j

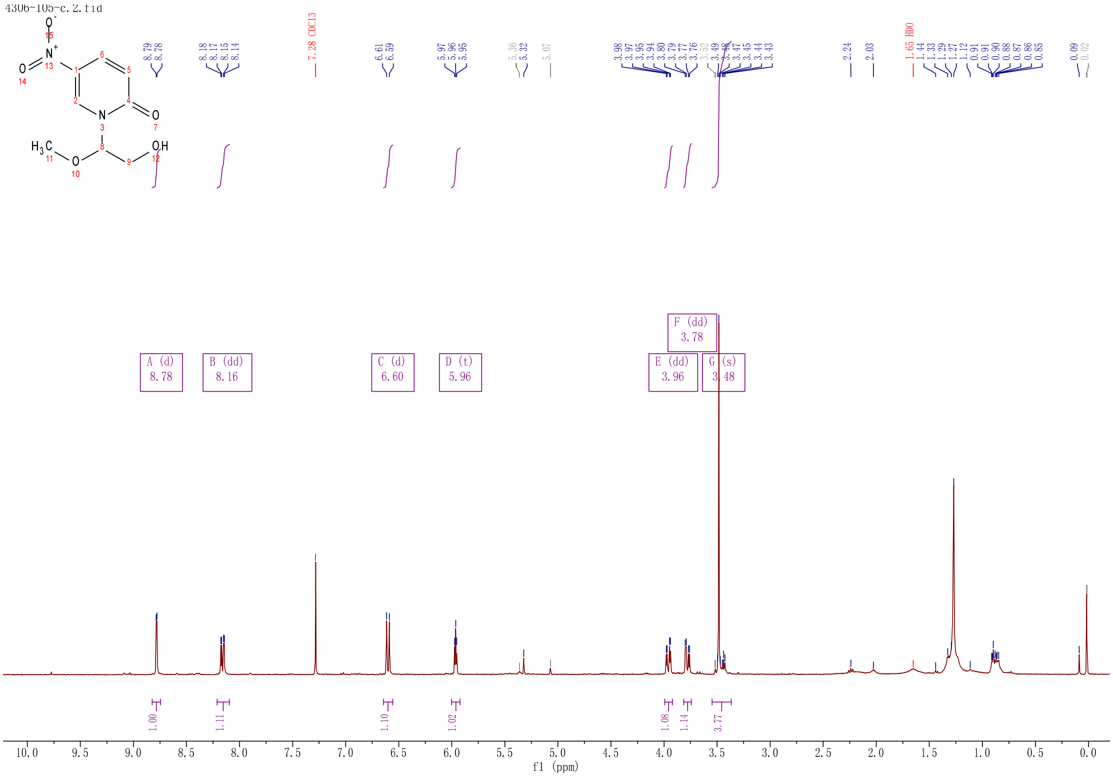

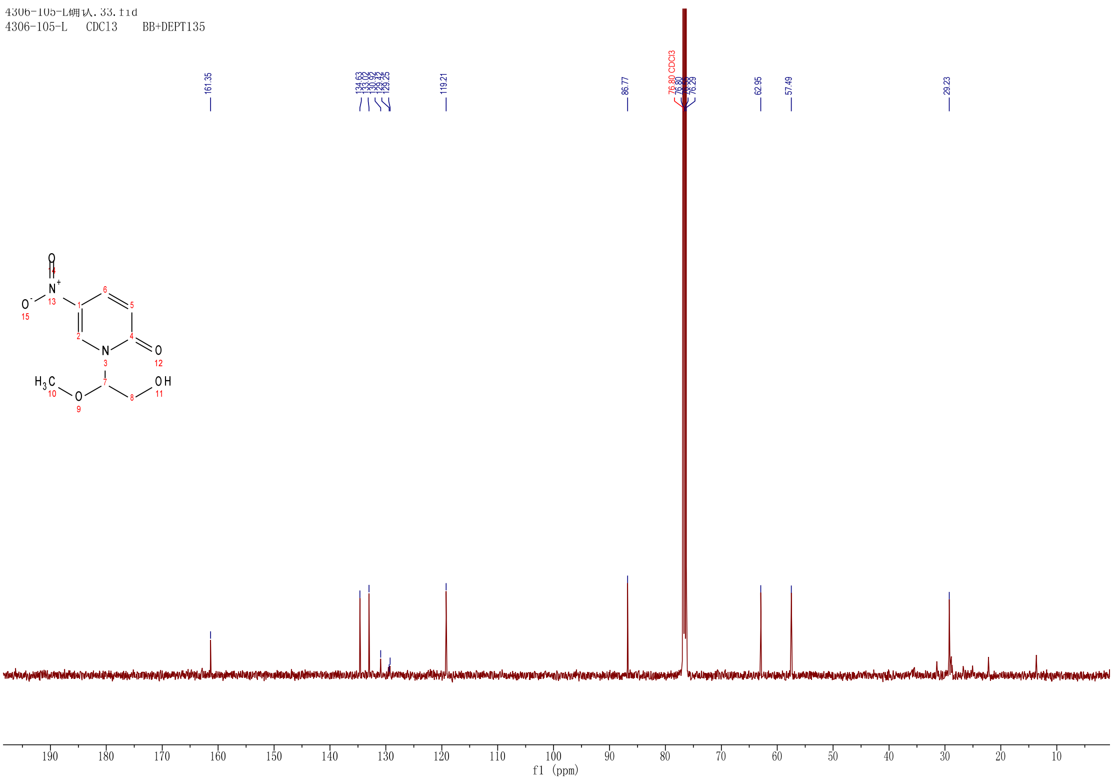

# 1-(2-hydroxy-1-methoxyethyl)-5-(trifluoromethyl)pyridin-2(1H)-one 5k

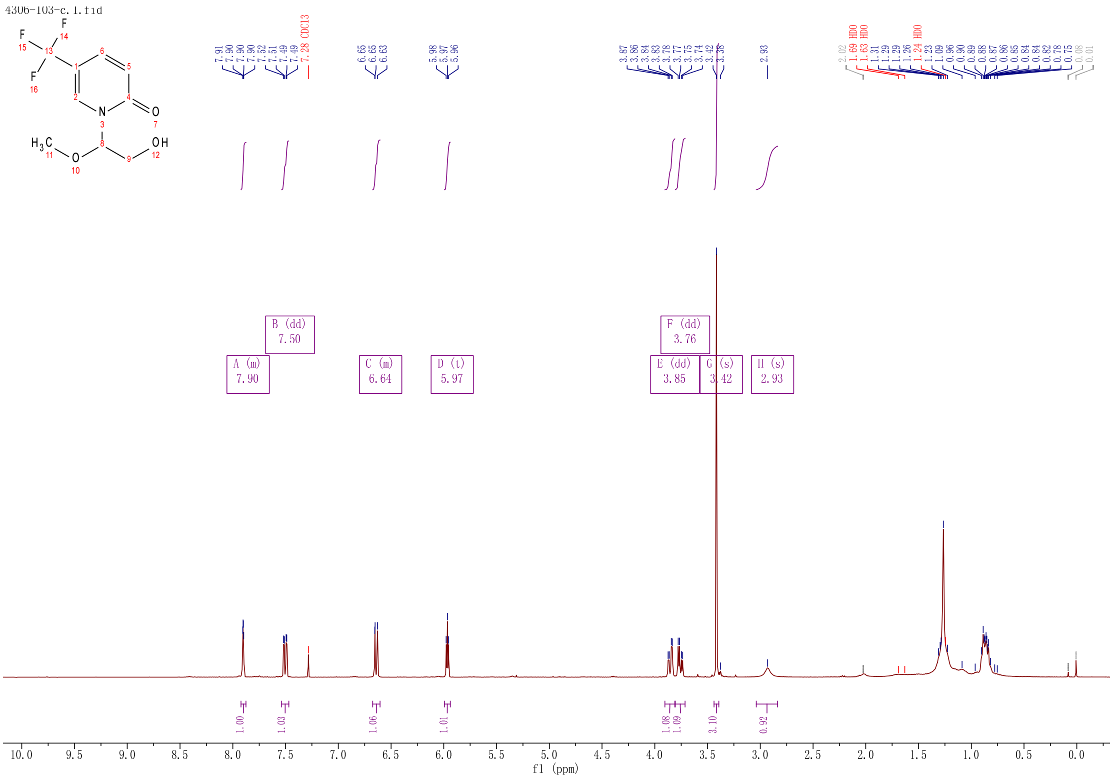

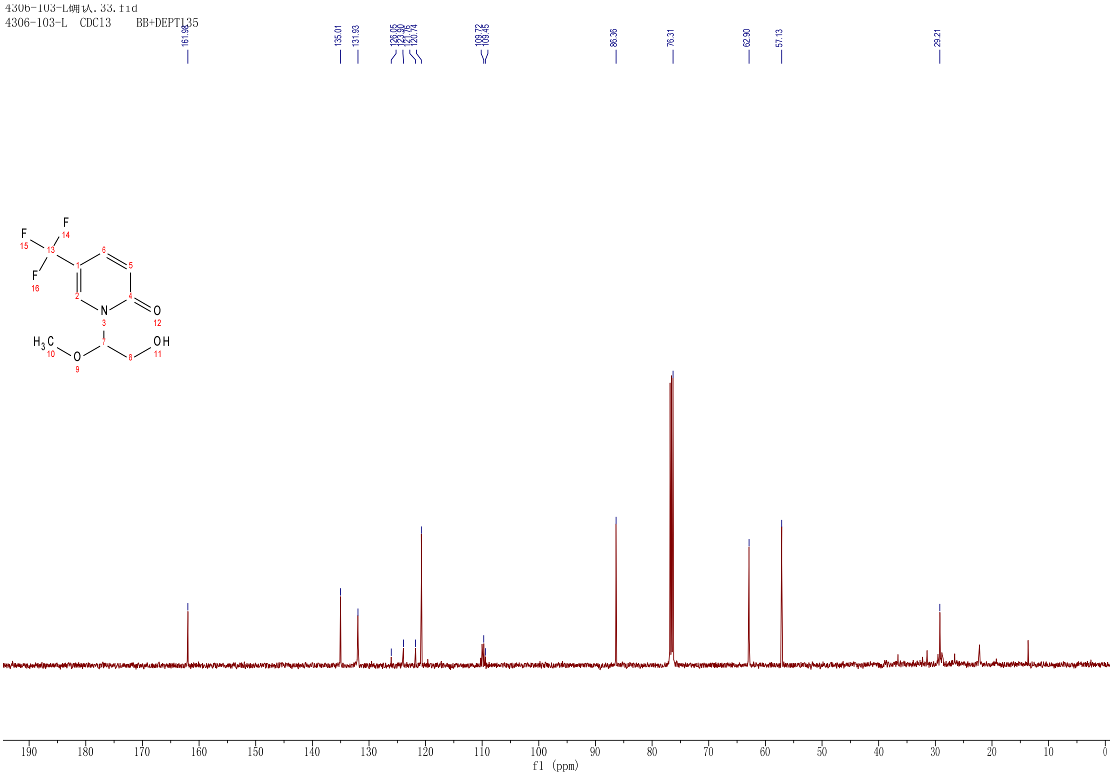


# 1-(2-hydroxy-1-methoxyethyl)quinolin-2(1H)-one 5l

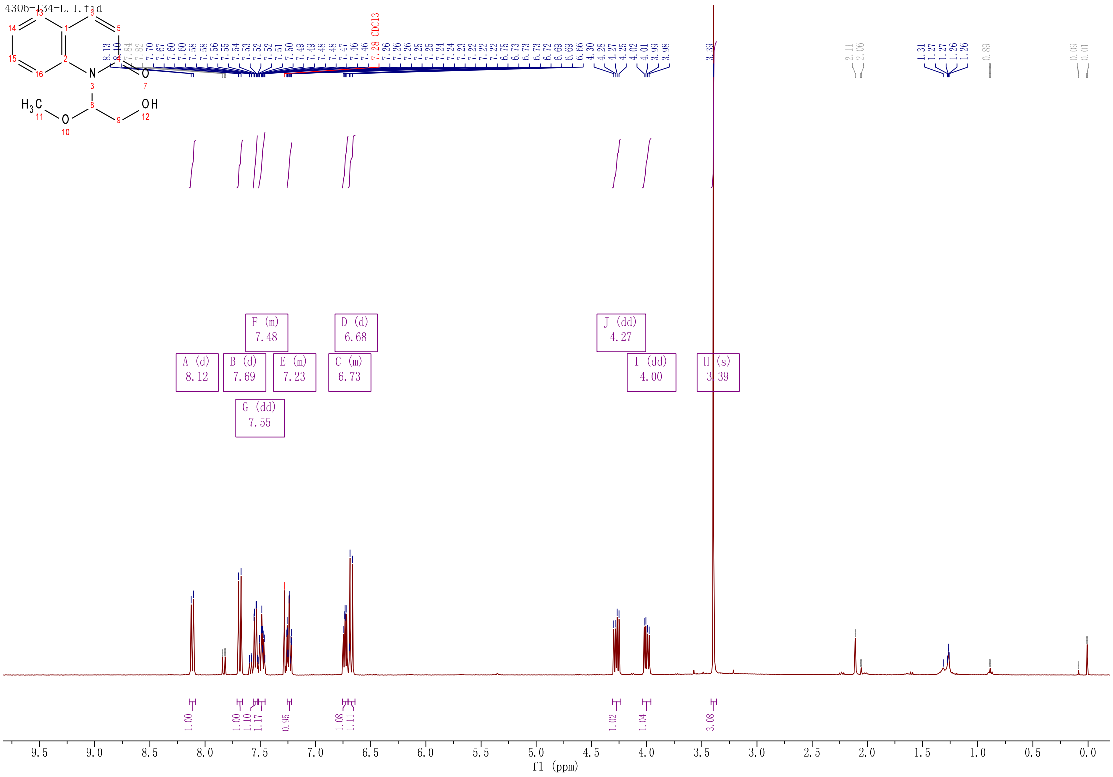

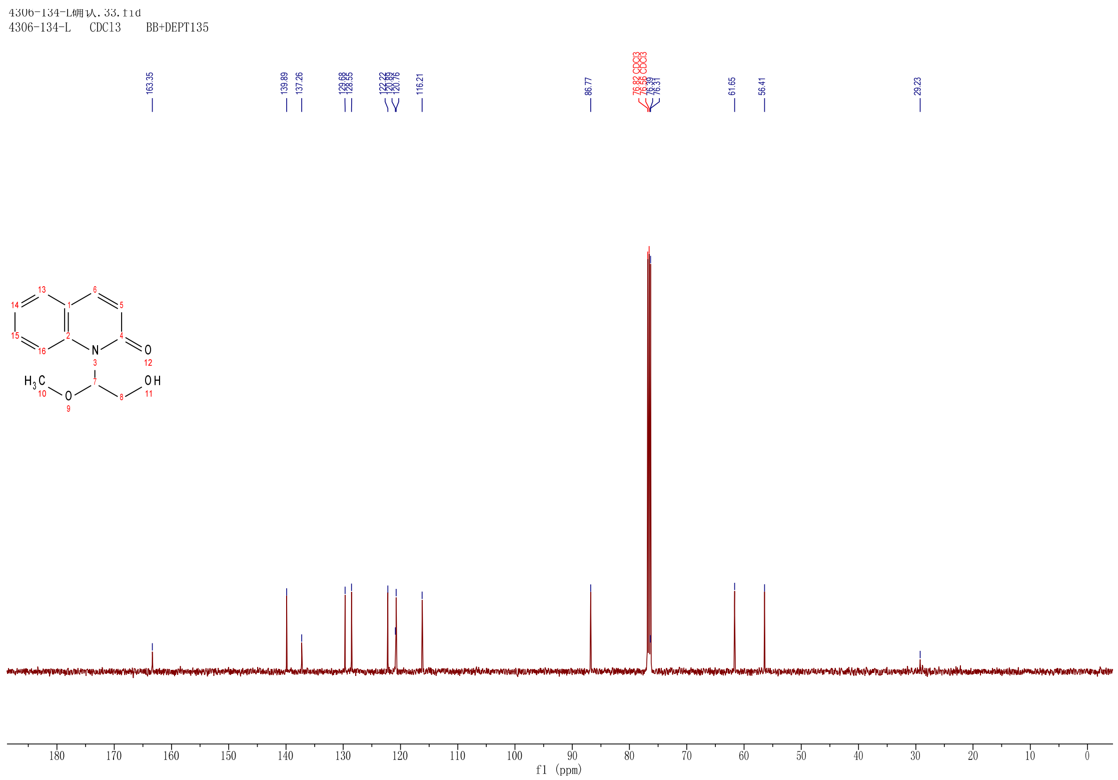

# 1-(2-hydroxy-1-methoxyethyl)-4-methylquinolin-2(1H)-one 5m

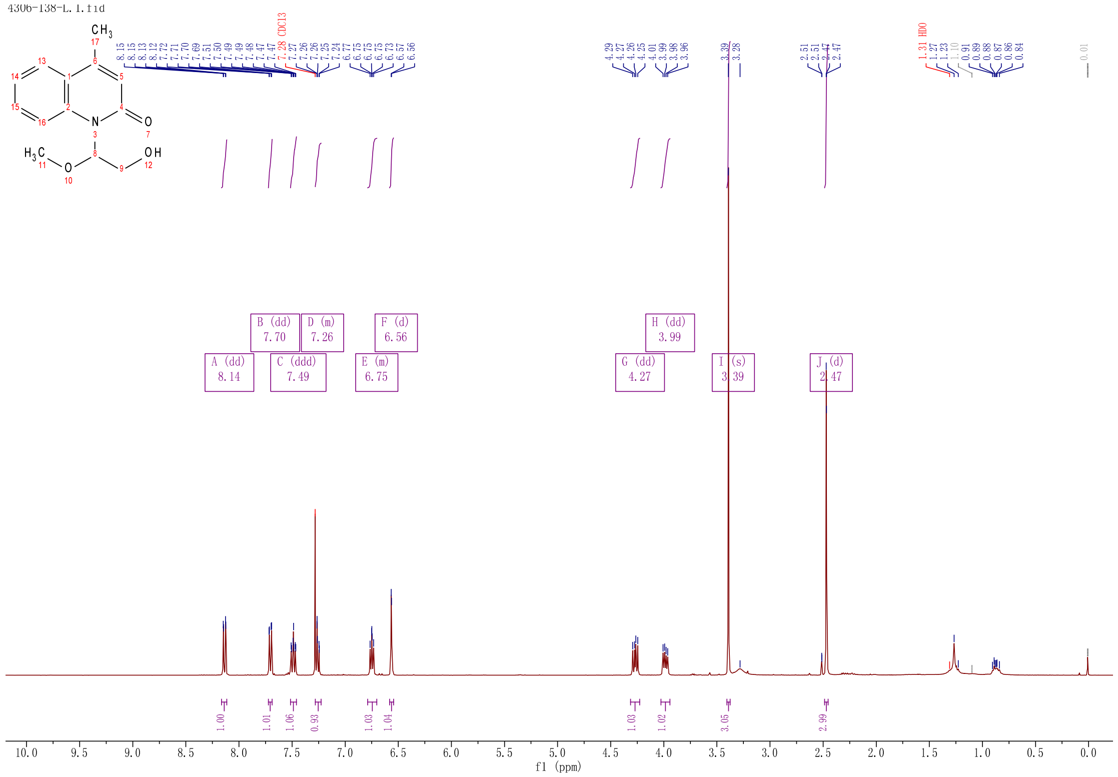

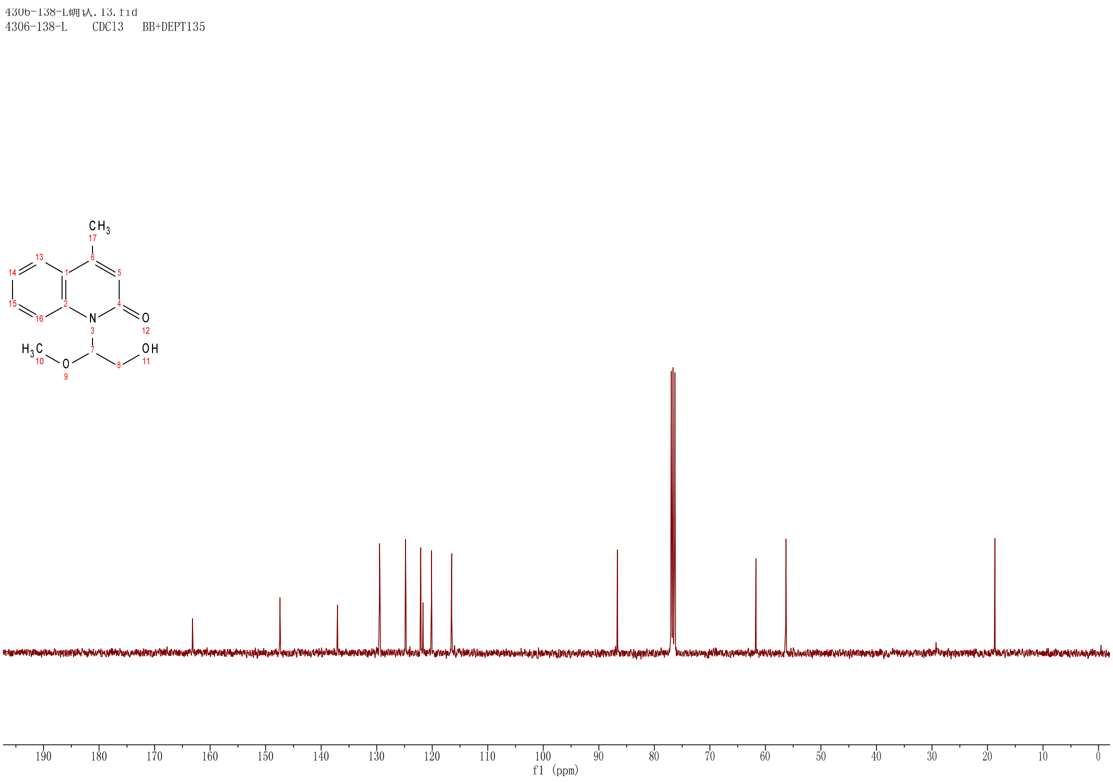


## 2-(2-hydroxy-1-methoxyethyl)isoquinolin-1(2H)-one 5n

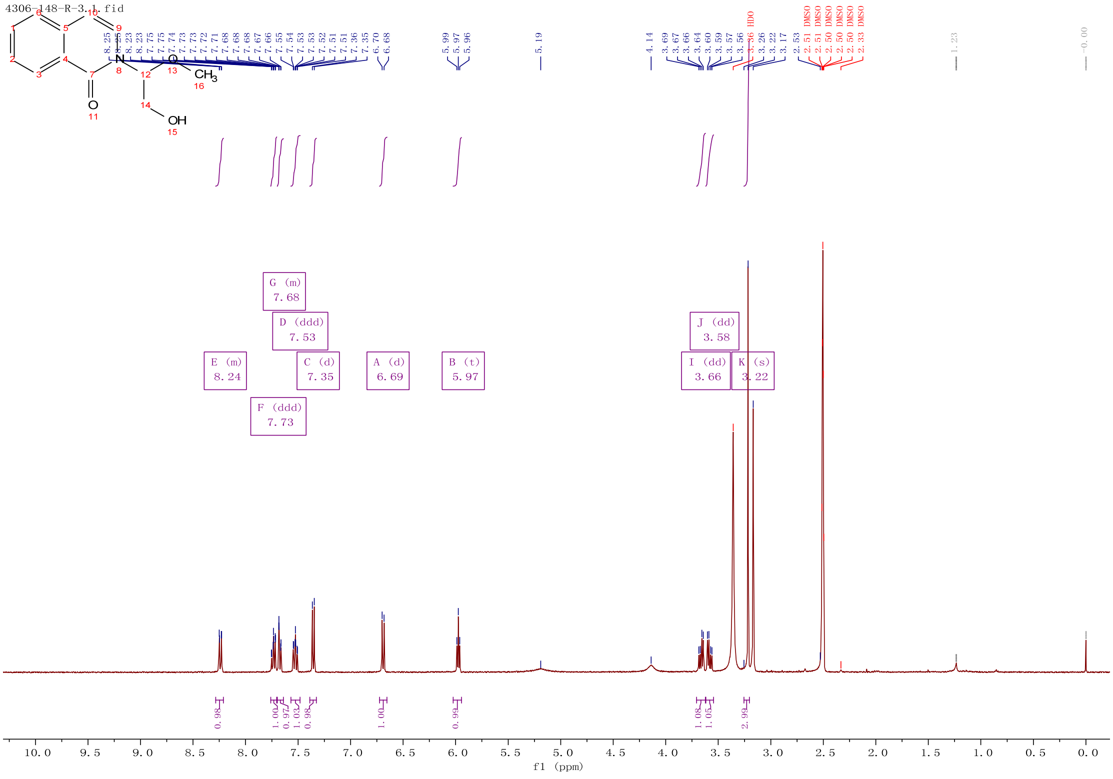

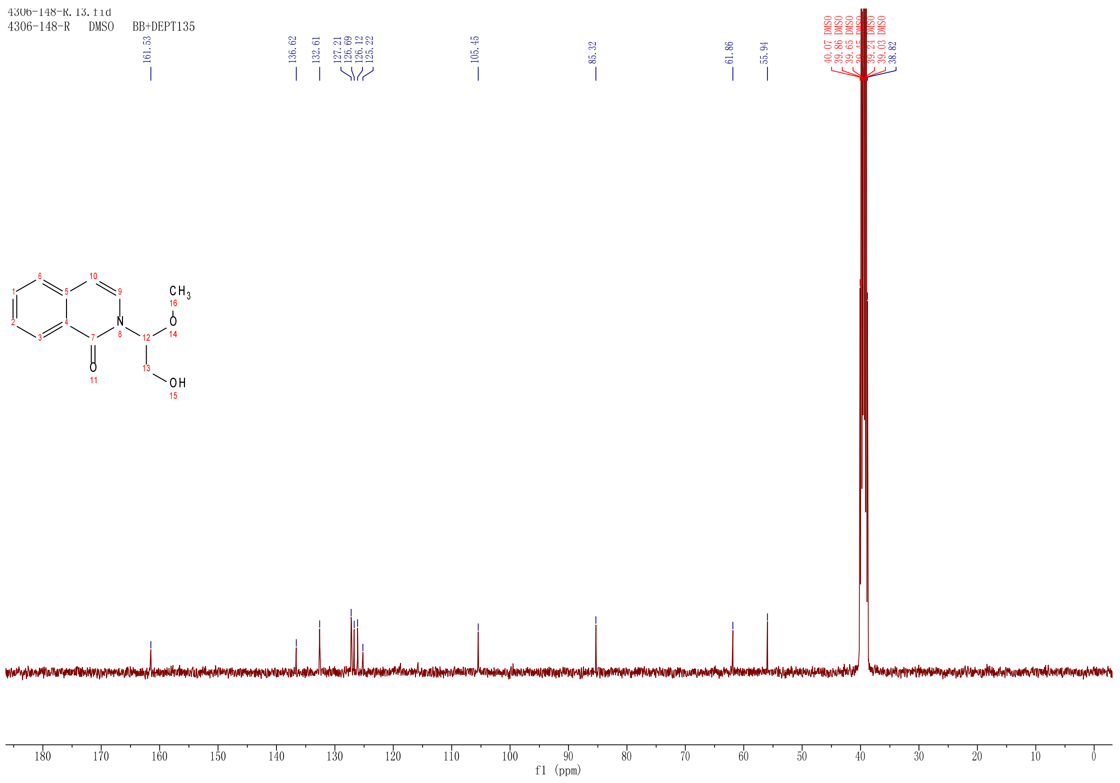


# 3-methoxy-2,3-dihydrooxazolo[3,2-a]pyridin-4-ium 2,2,2-trifluoroacetate 4a

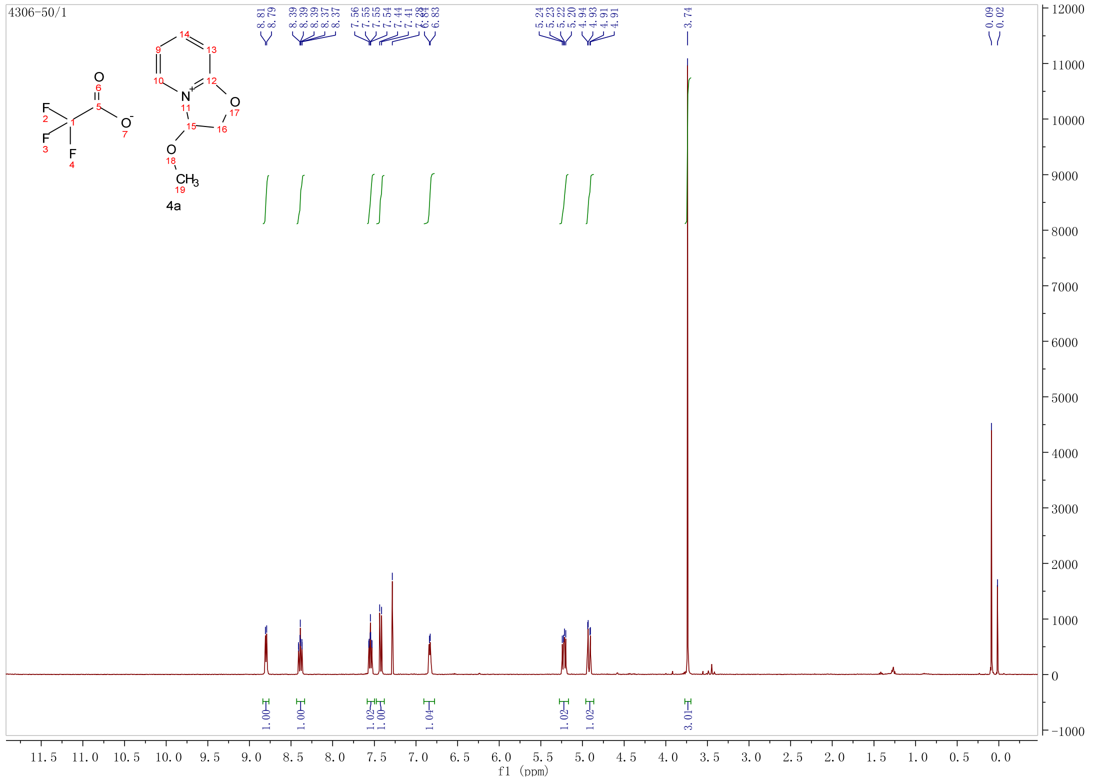

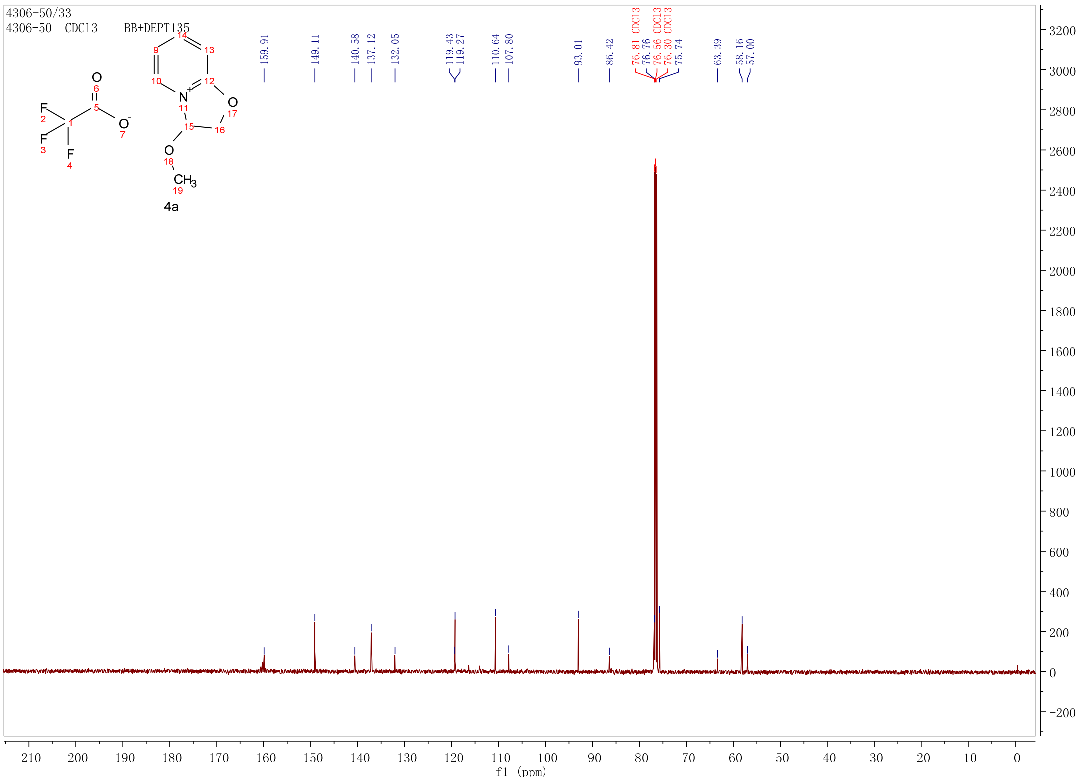


# 3-methoxy-2,3-dihydrooxazolo[2,3-a]isoquinolin-4-ium chloride 4n

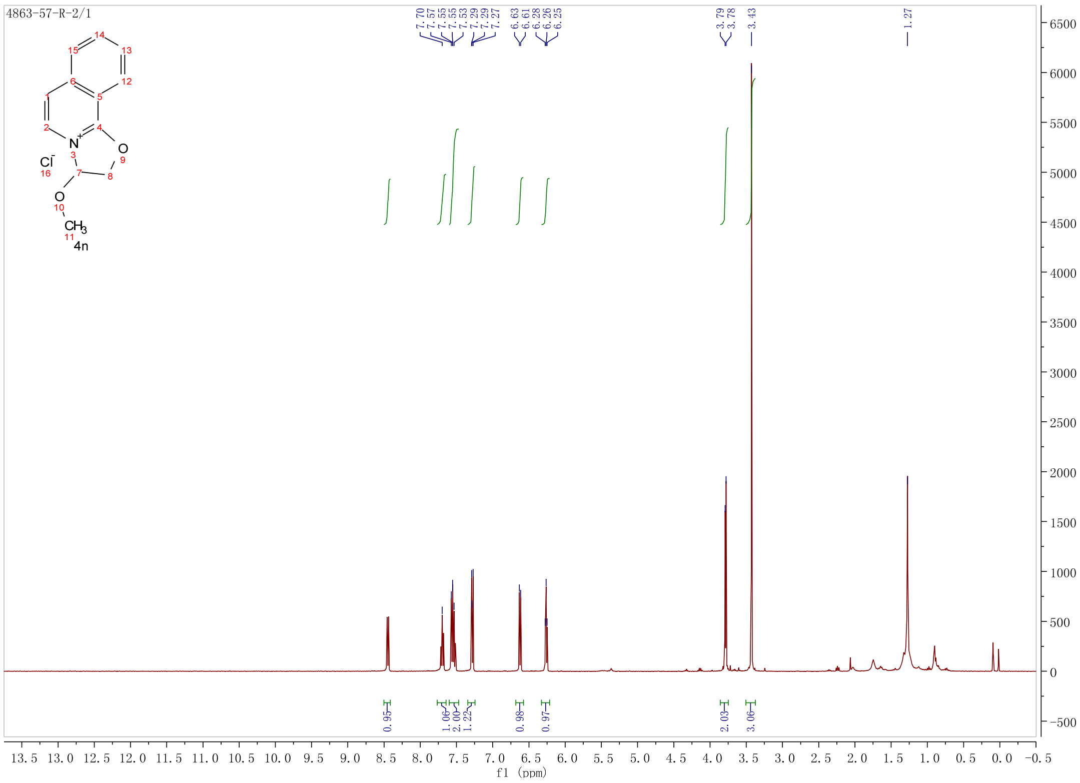

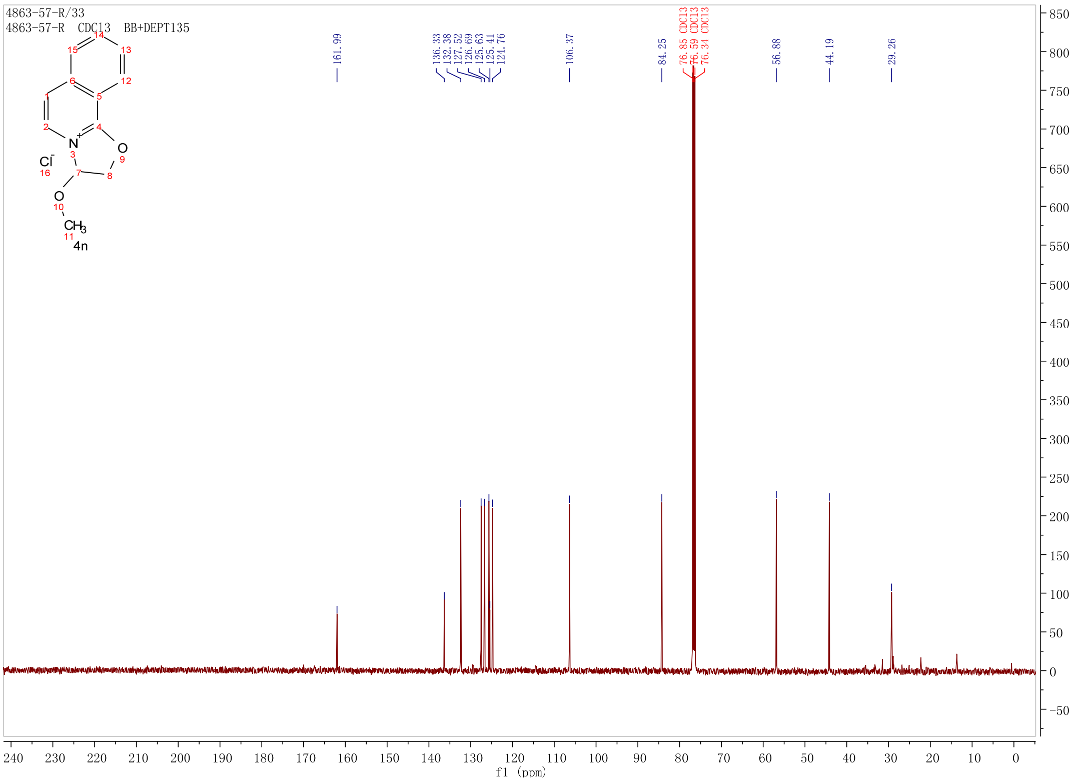

## 1-methoxy-1,2-dihydrooxazolo[3,2-a]quinolin-10-ium chloride 4l

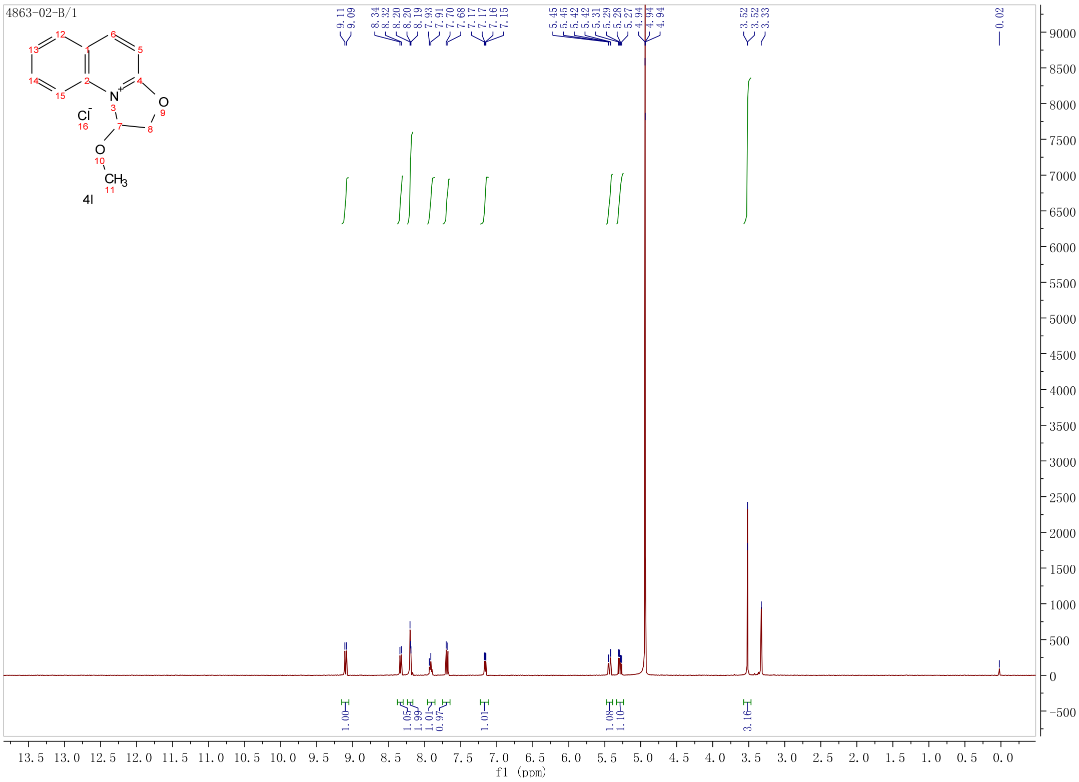

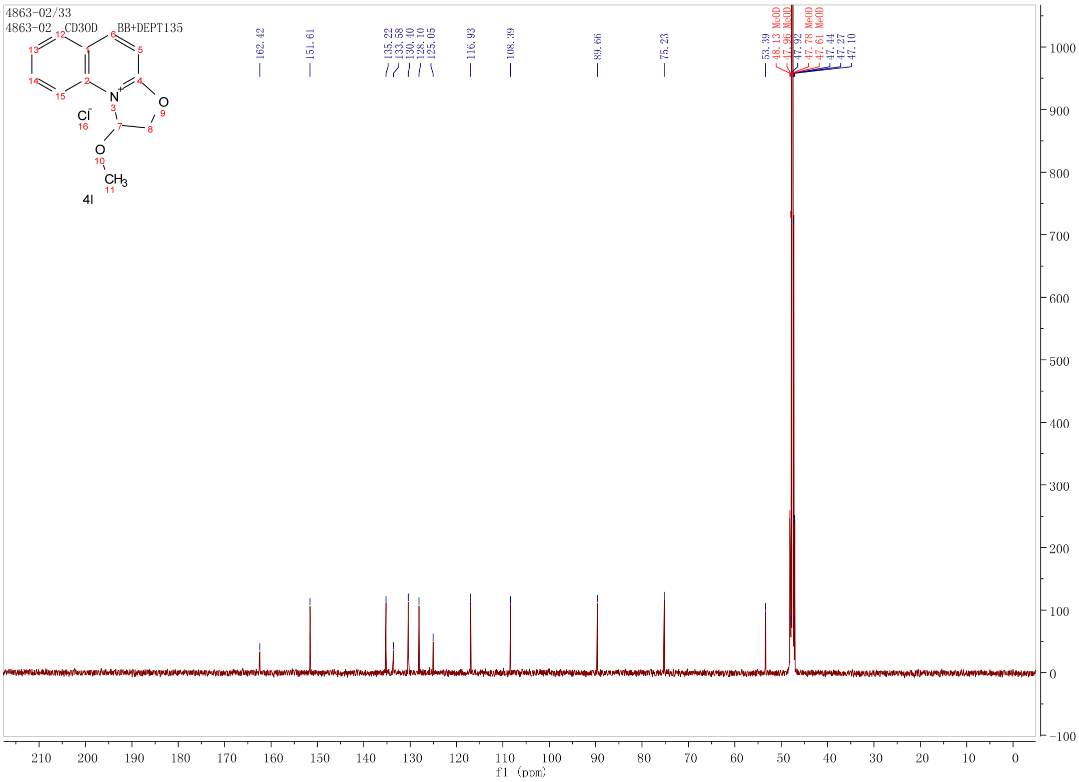

## 1-(1-methoxy-2-(phenylamino)ethyl)pyridin-2(1H)-one 5A

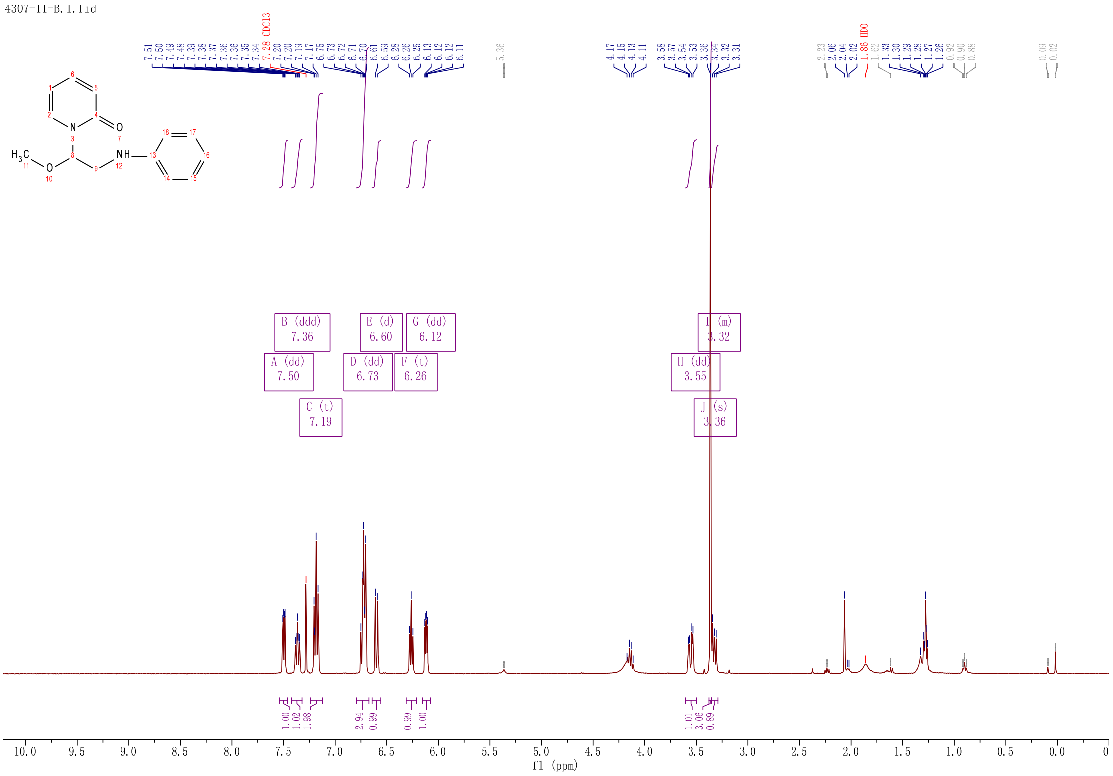

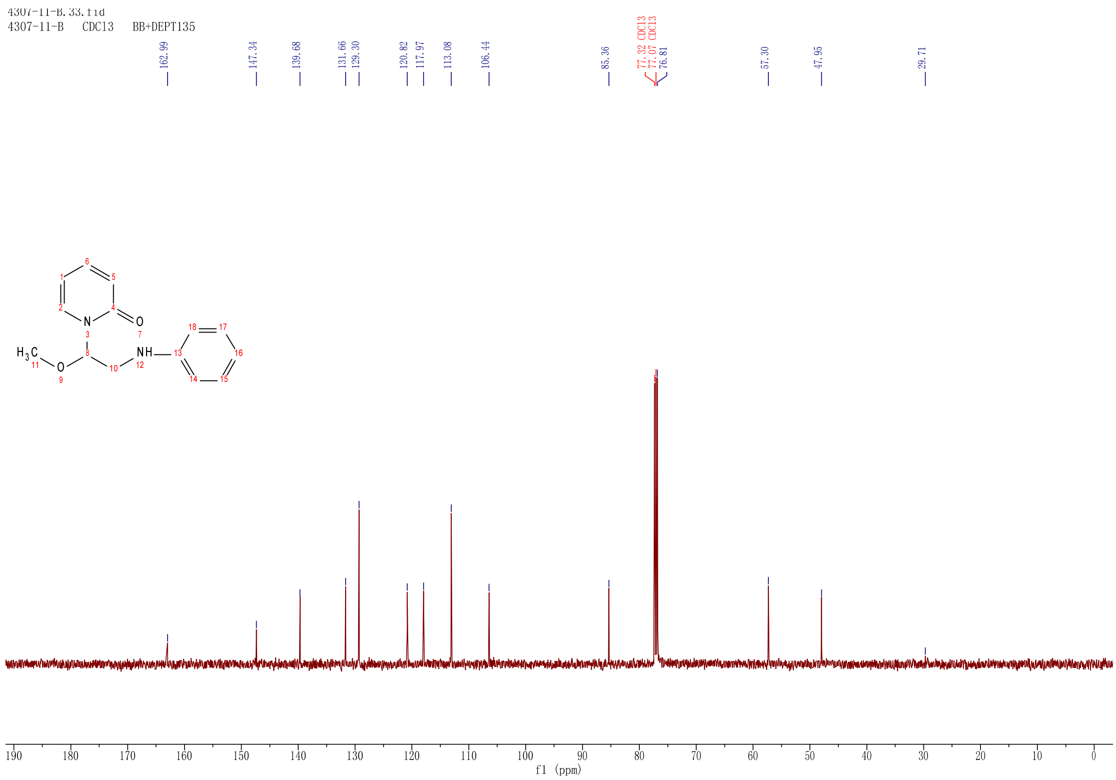


# 1-(1-methoxy-2-((3-methoxyphenyl)amino)ethyl)pyridin-2(1H)-one 5B

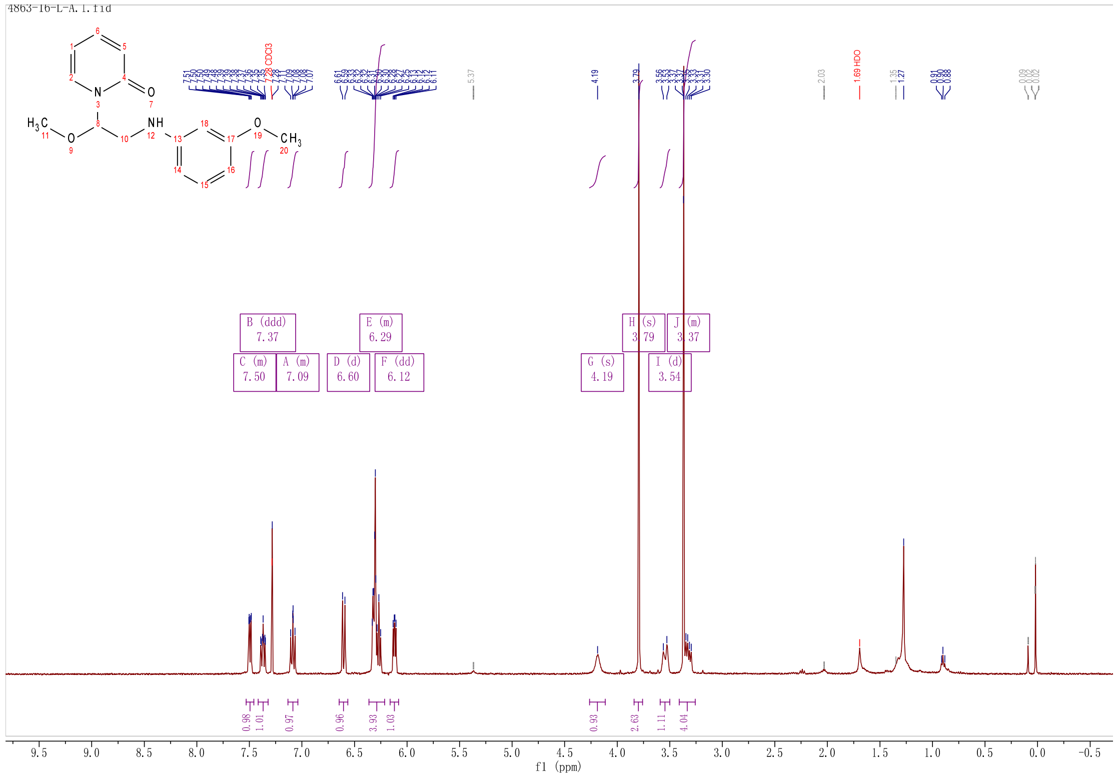

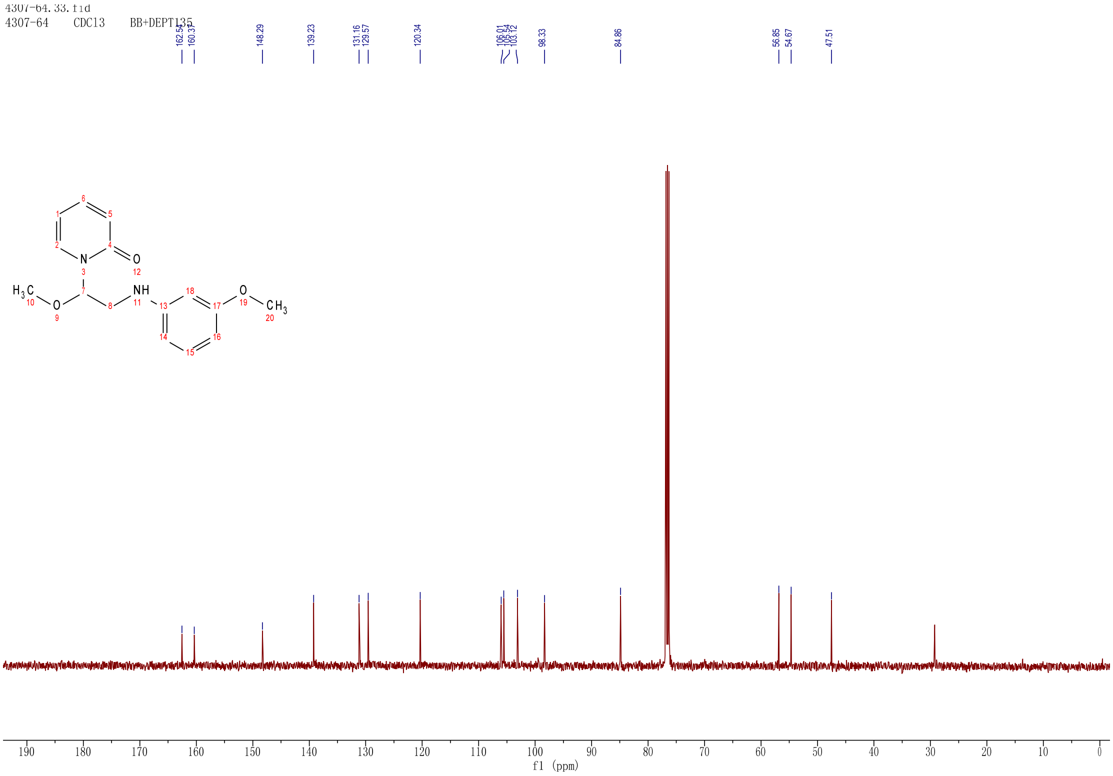


# 1-(1-methoxy-2-((4-methoxyphenyl)amino)ethyl)pyridin-2(1H)-one 5C

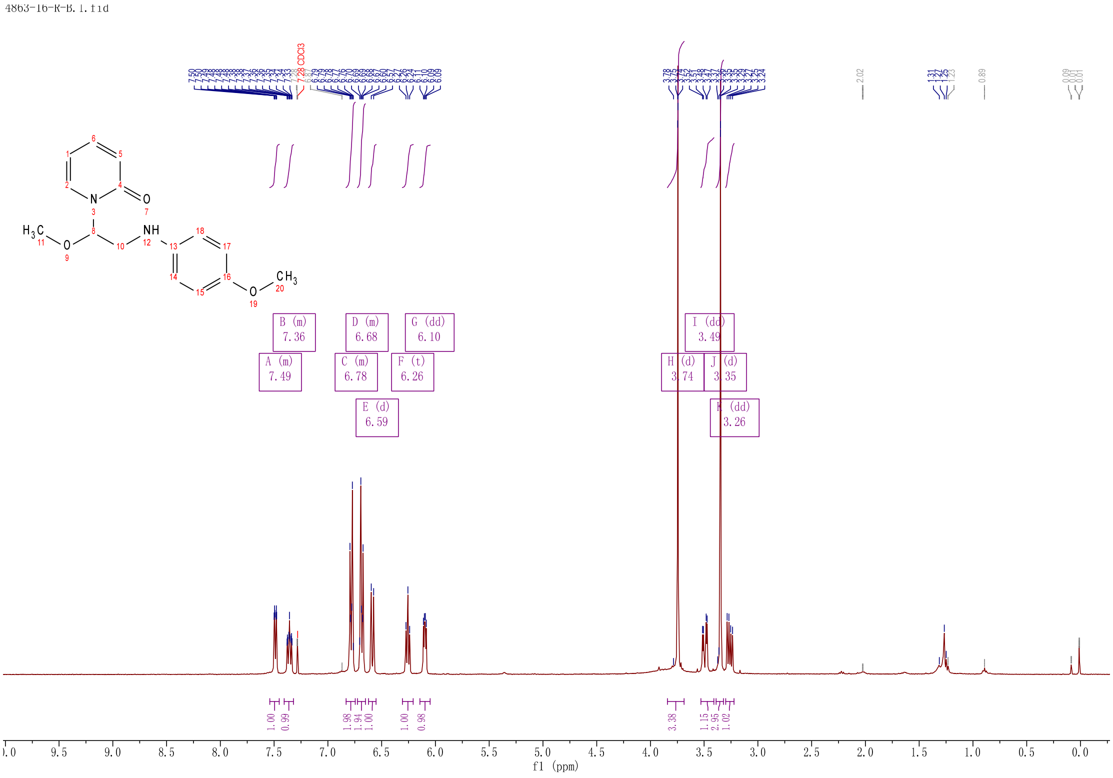

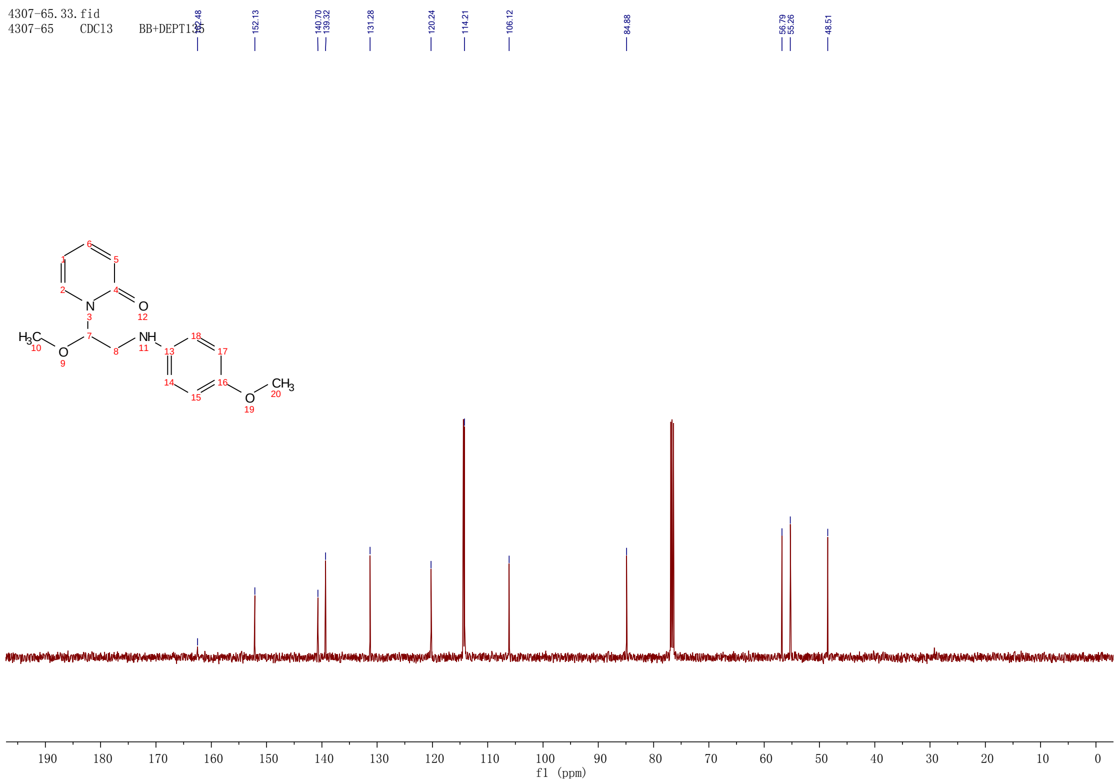


# 1-(1-methoxy-2-((3-nitrophenyl)amino)ethyl)pyridin-2(1H)-one 5D

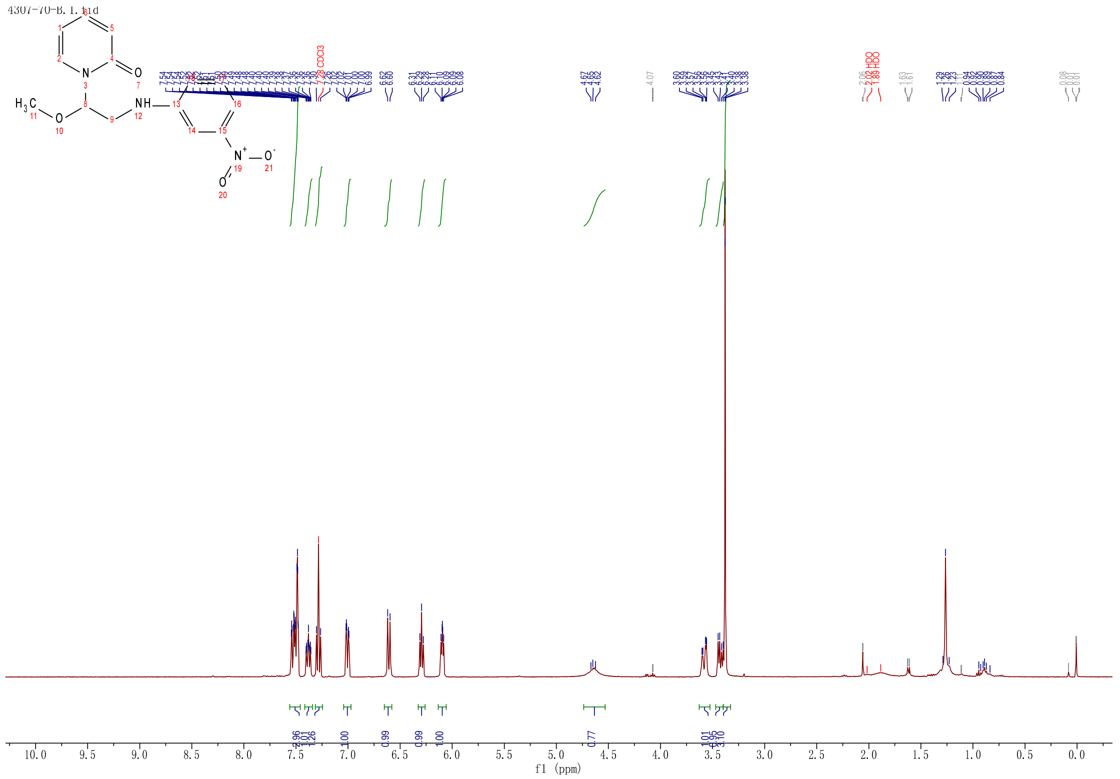

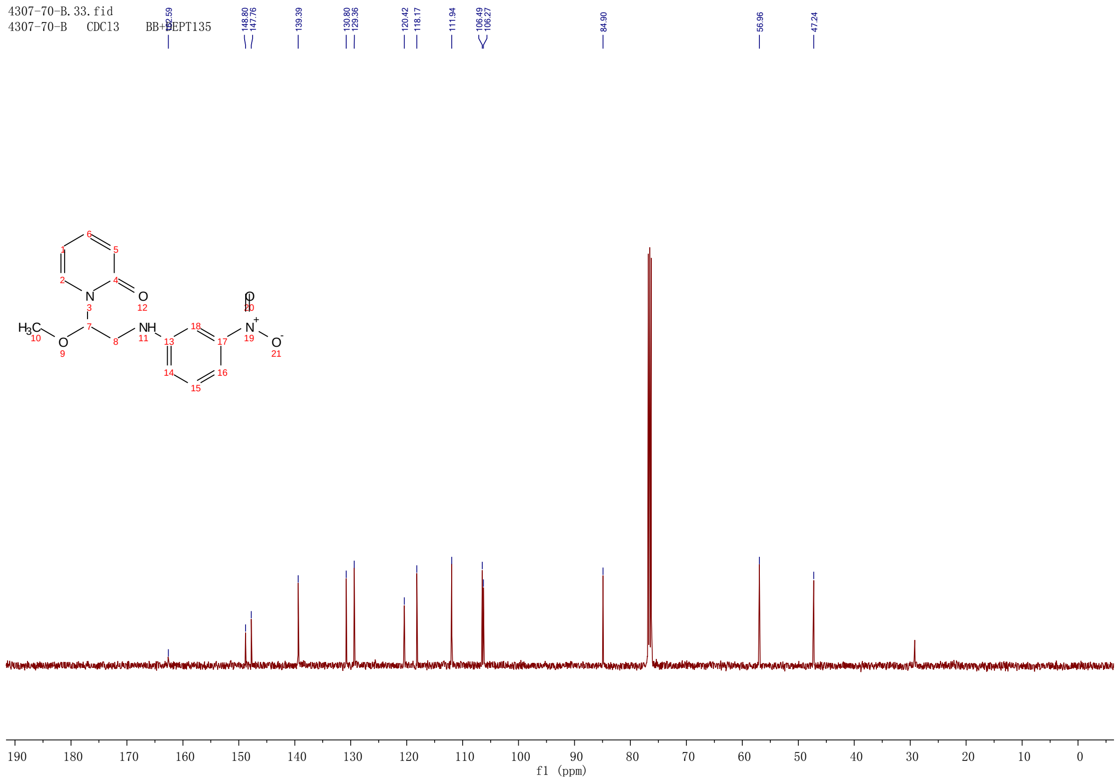


# 1-(2-((3-chlorophenyl)amino)-1-methoxyethyl)pyridin-2(1H)-one 5G

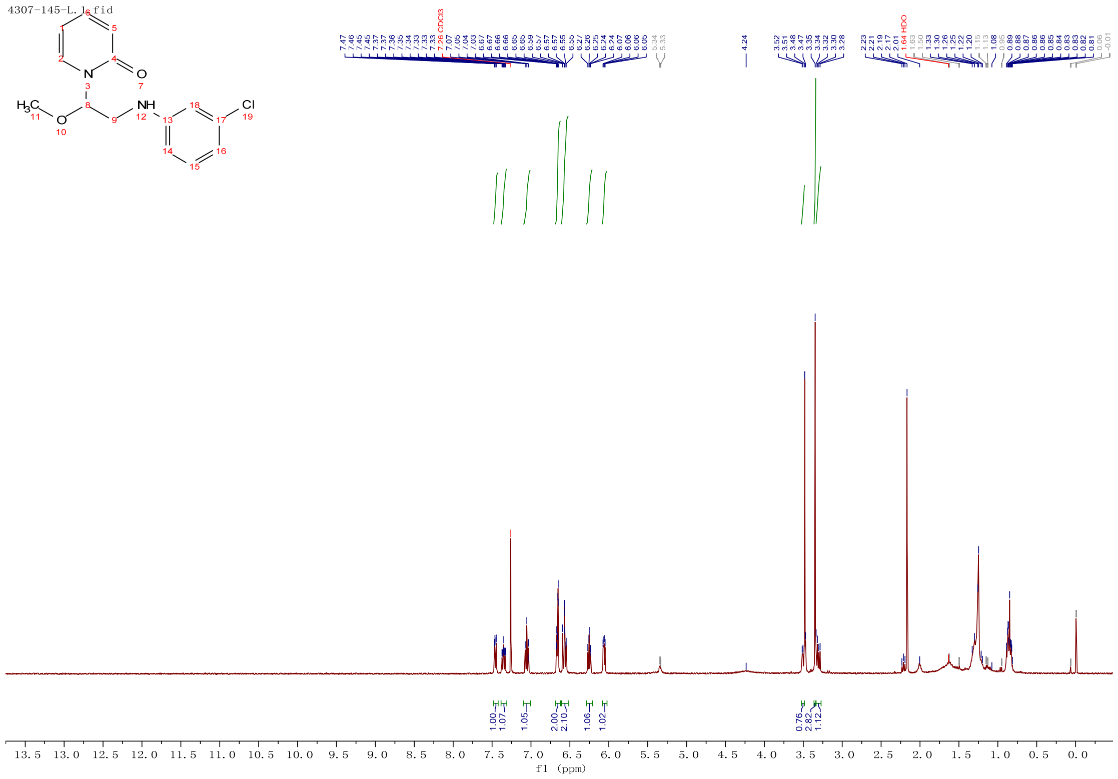

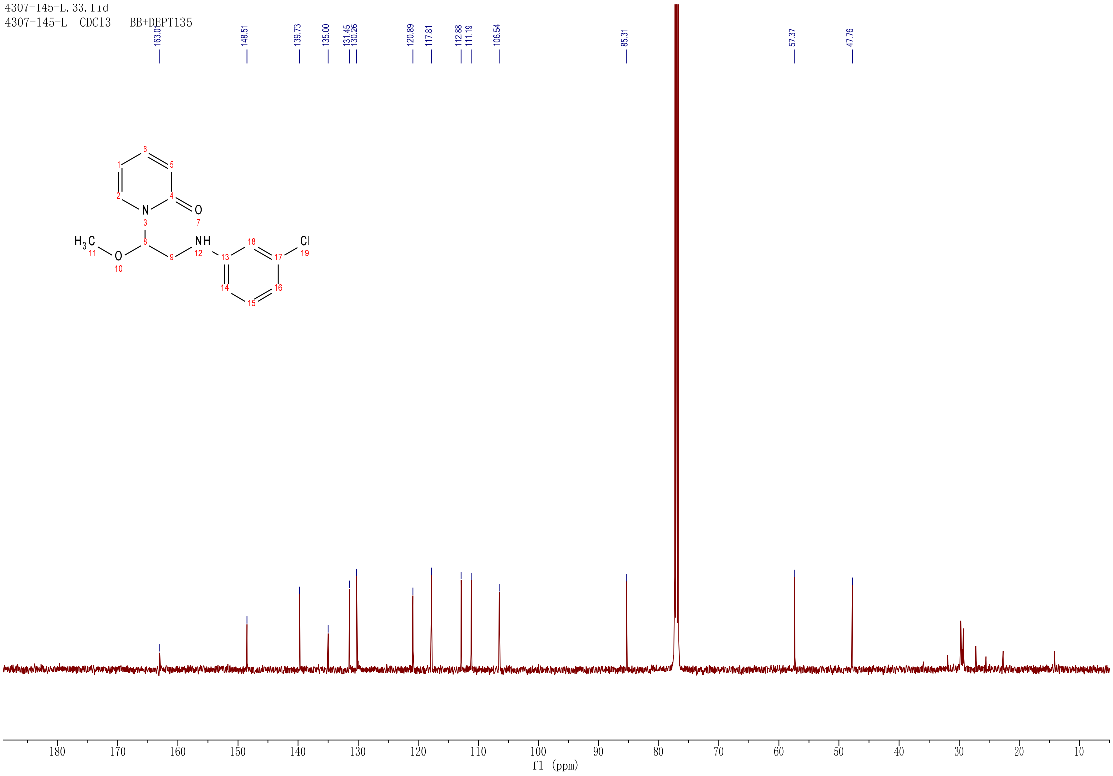


# 1-(2-((4-bromophenyl)amino)-1-methoxyethyl)pyridin-2(1H)-one 5H

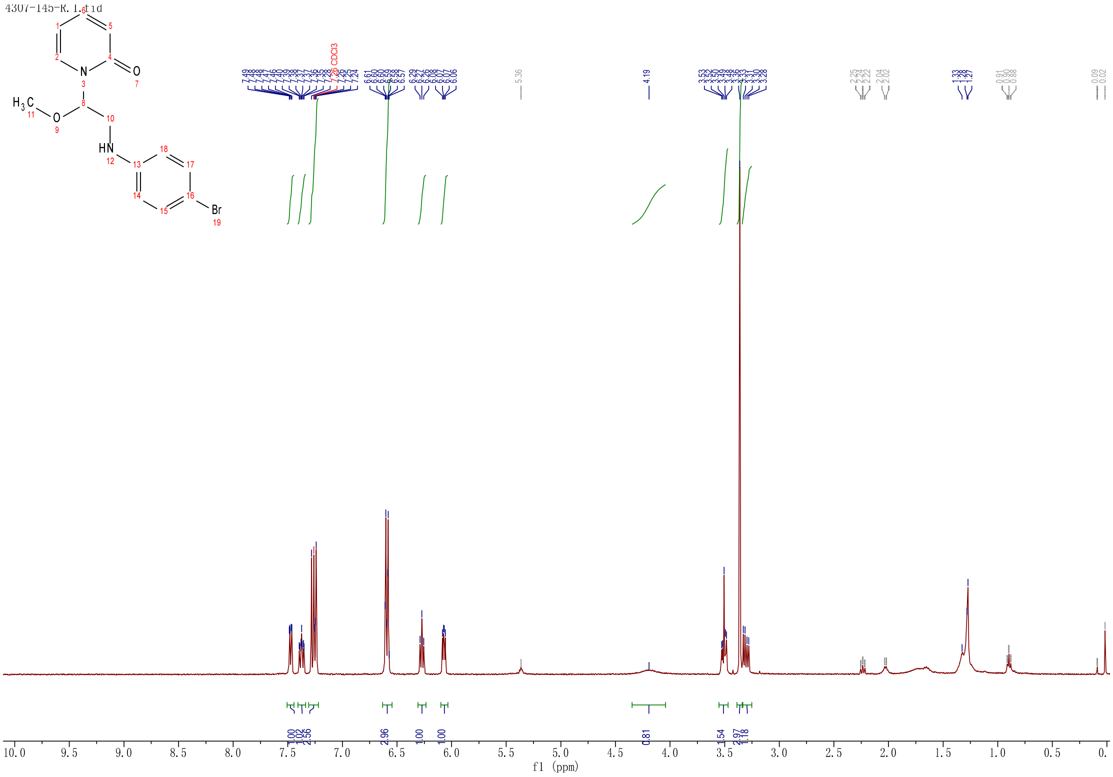

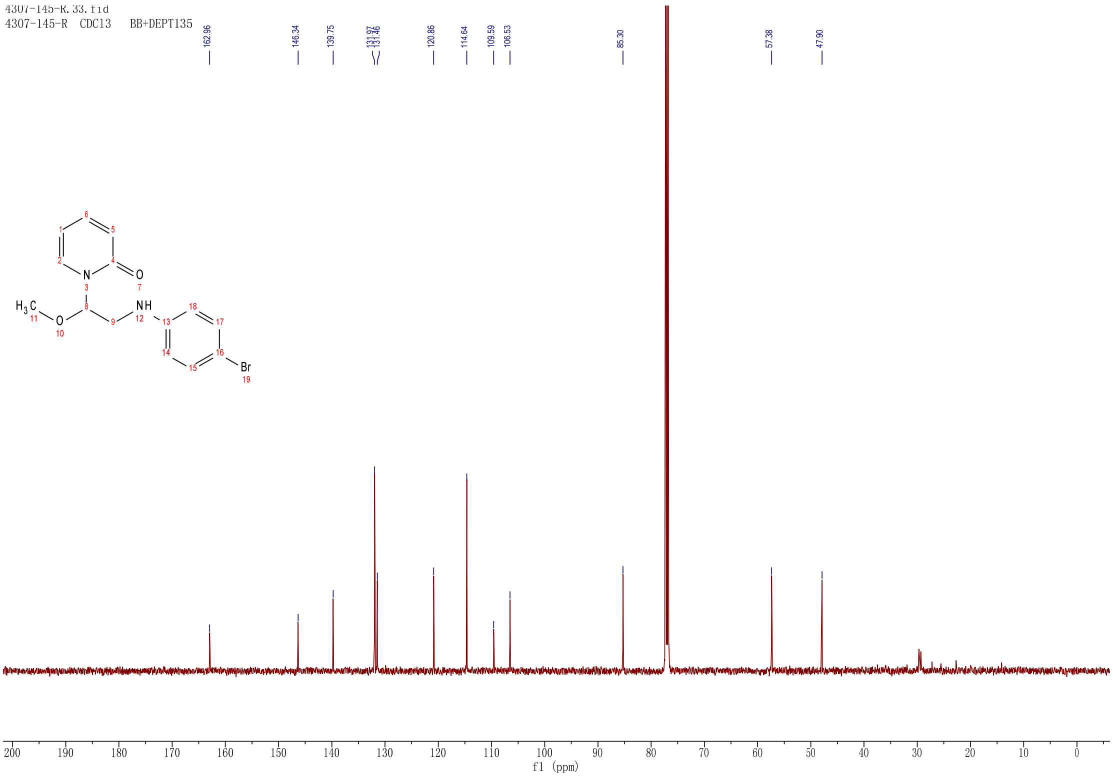


## 1-(1-methoxy-2-phenoxyethyl)pyridin-2(1H)-one 5I

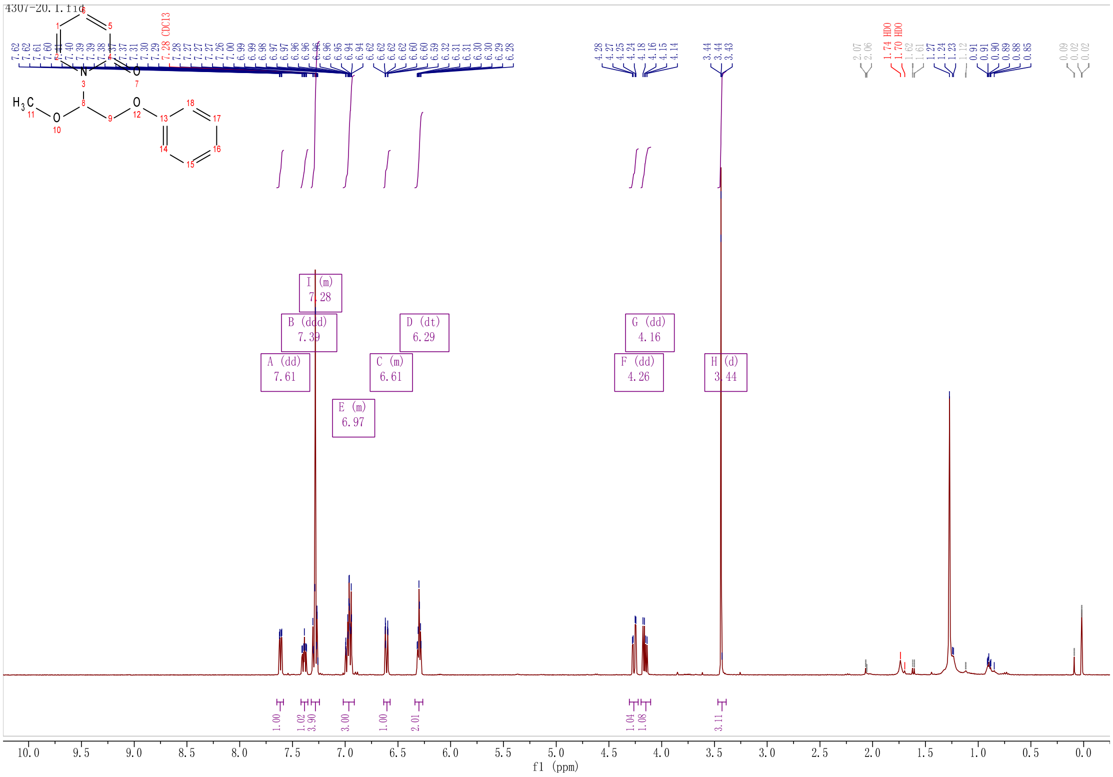

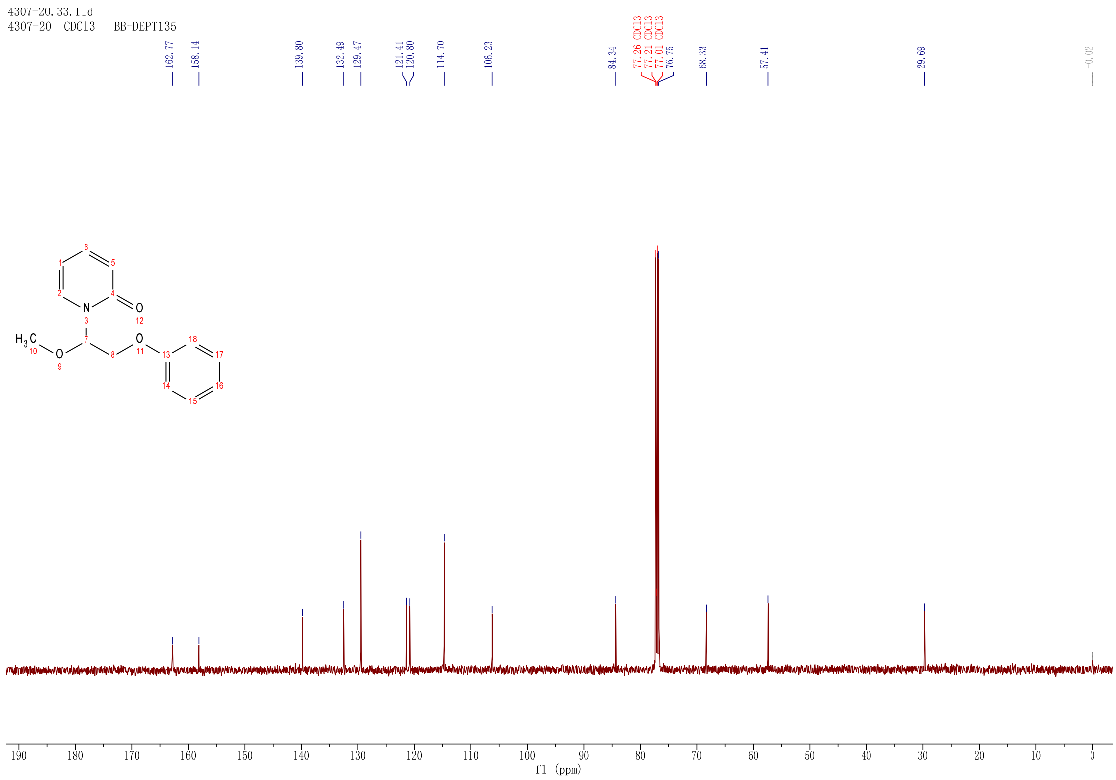

# 1-(1-methoxy-2-(phenylthio)ethyl)pyridin-2(1H)-one 5J

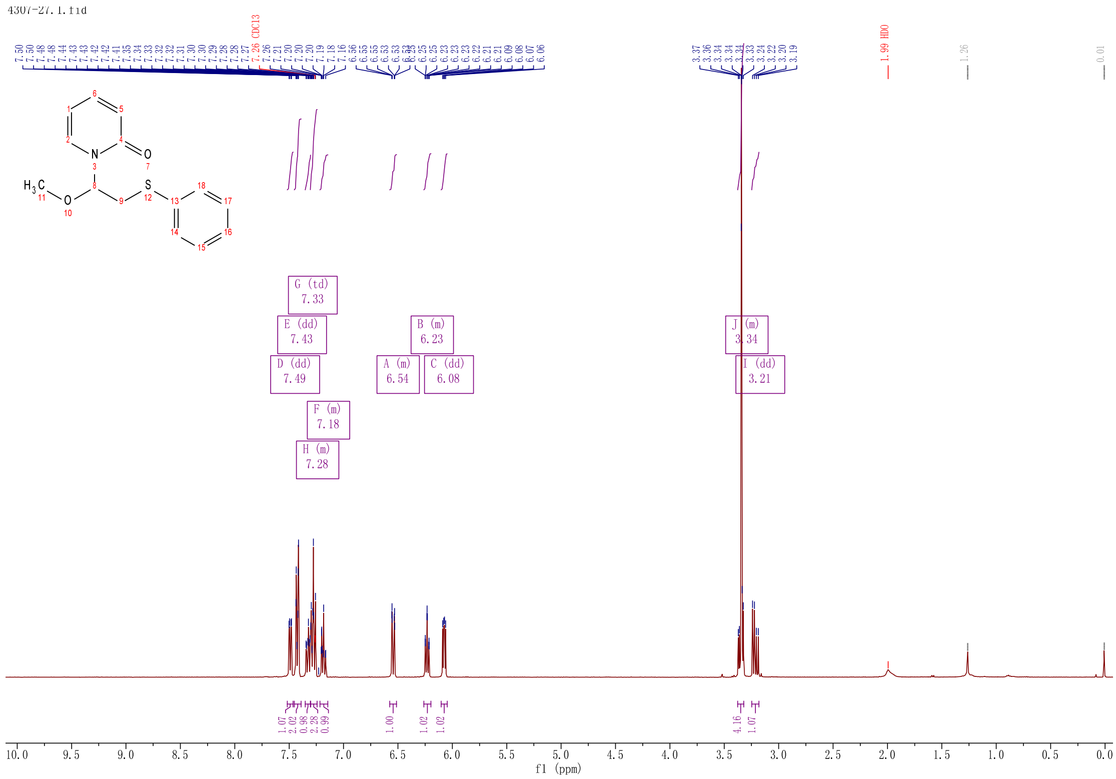

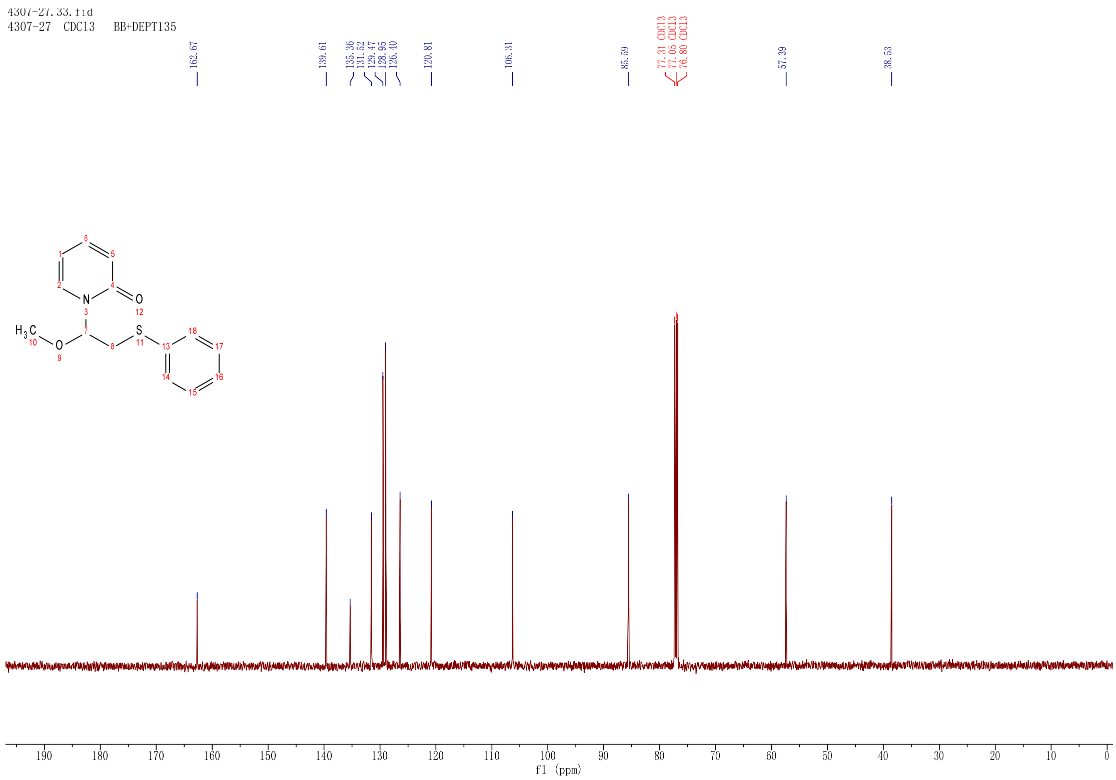


**1-(1-methoxy-2-(methylamino)ethyl)pyridin-2(1H)-one 5K**

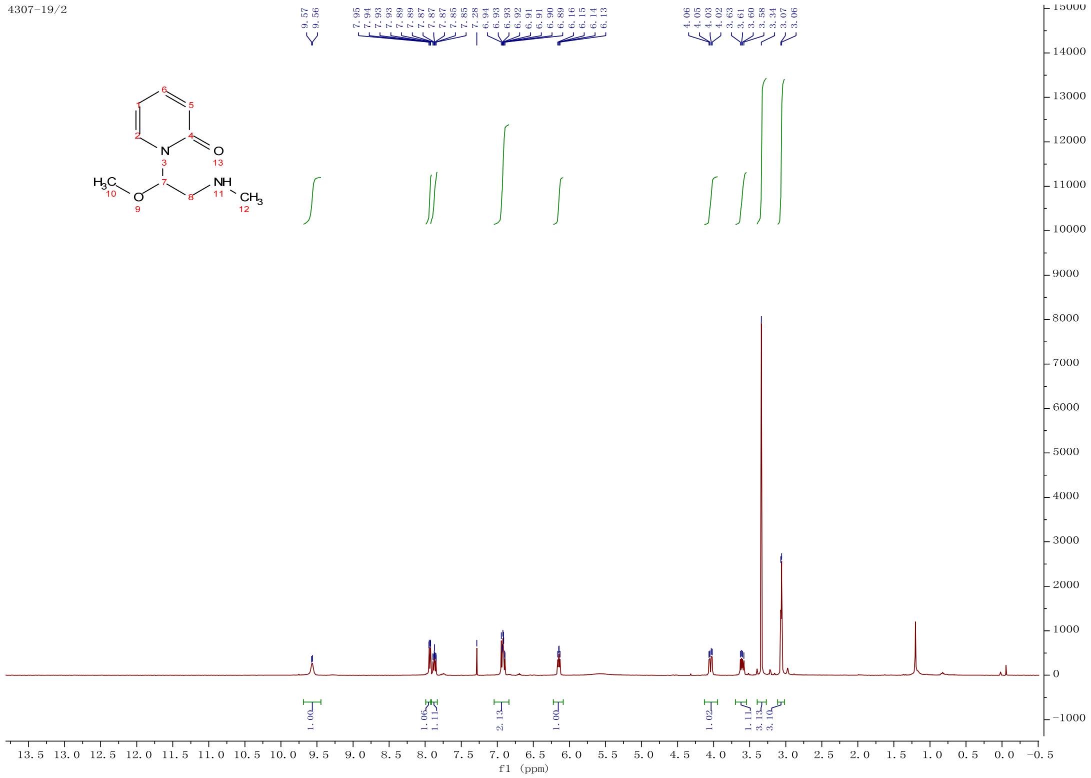

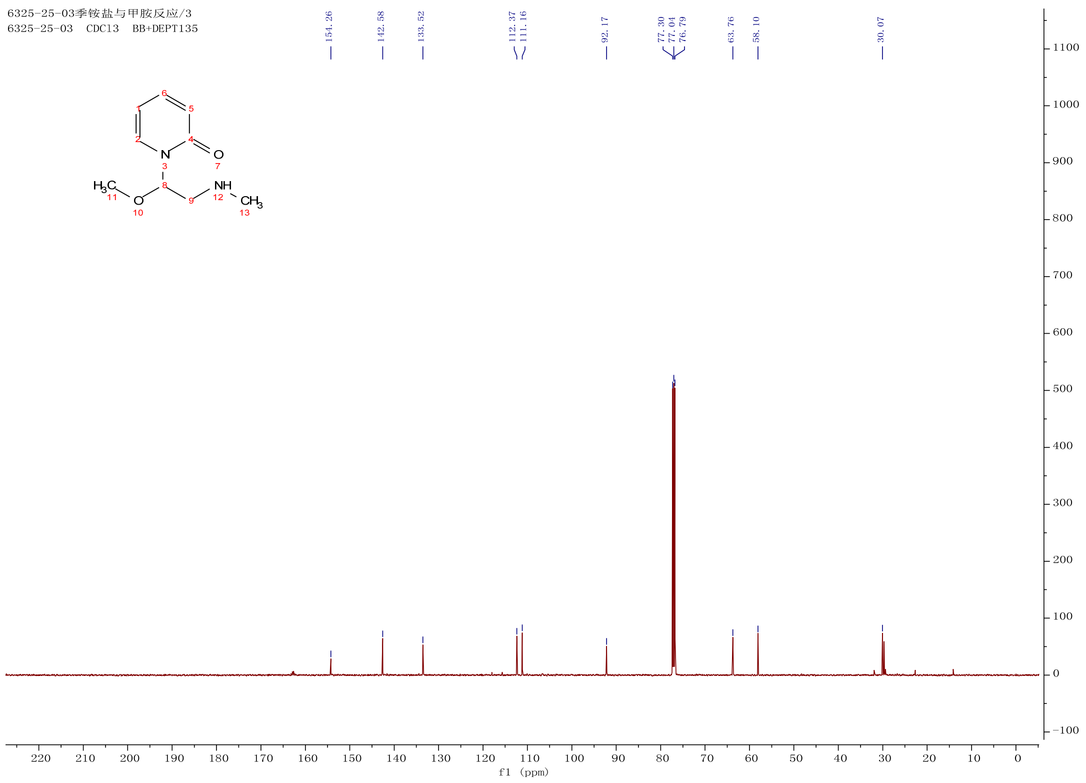


# 1-(1-methoxy-2-(propylamino)ethyl)pyridin-2(1H)-one 5L

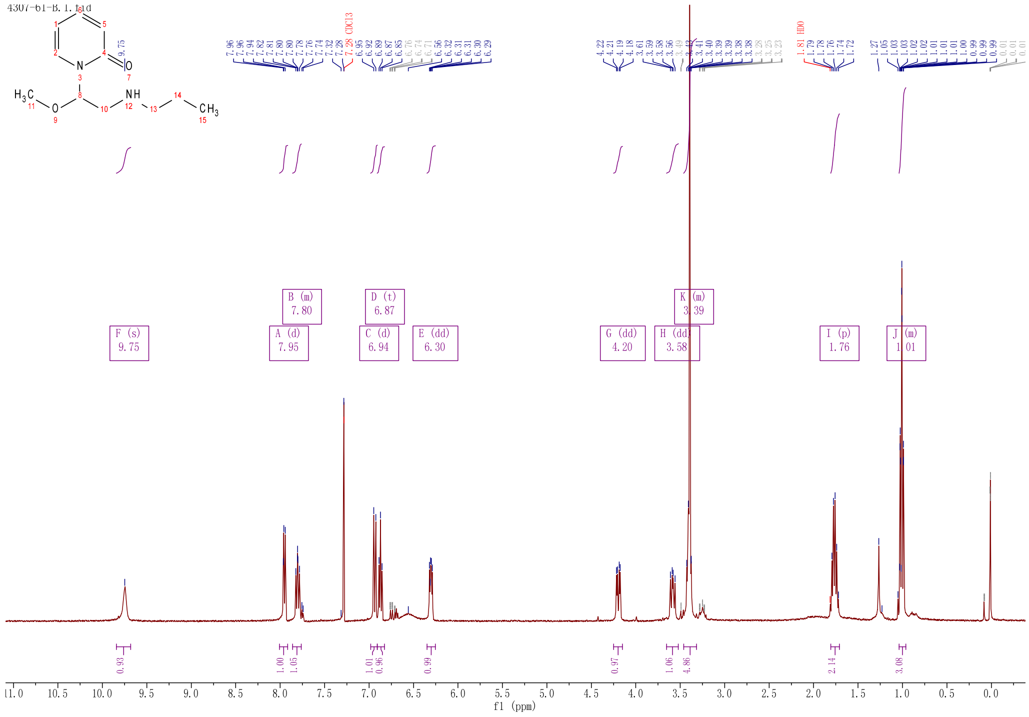

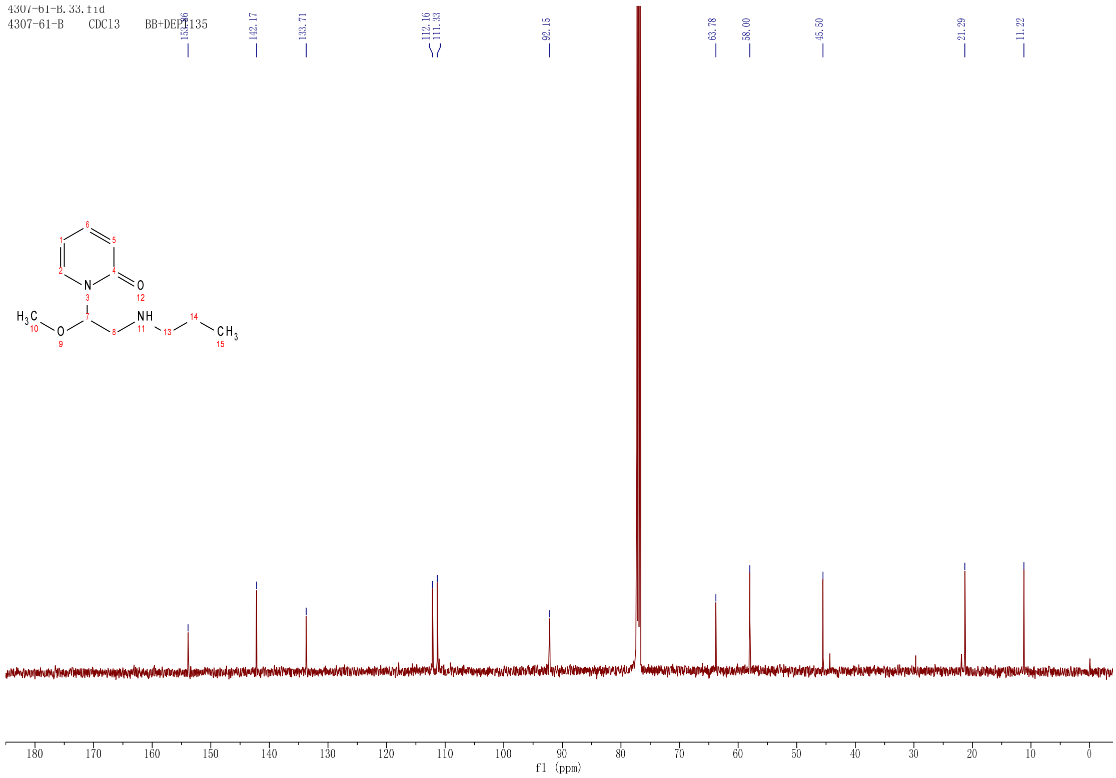


## 1-(2-(isopropylamino)-1-methoxyethyl)pyridin-2(1H)-one 5M

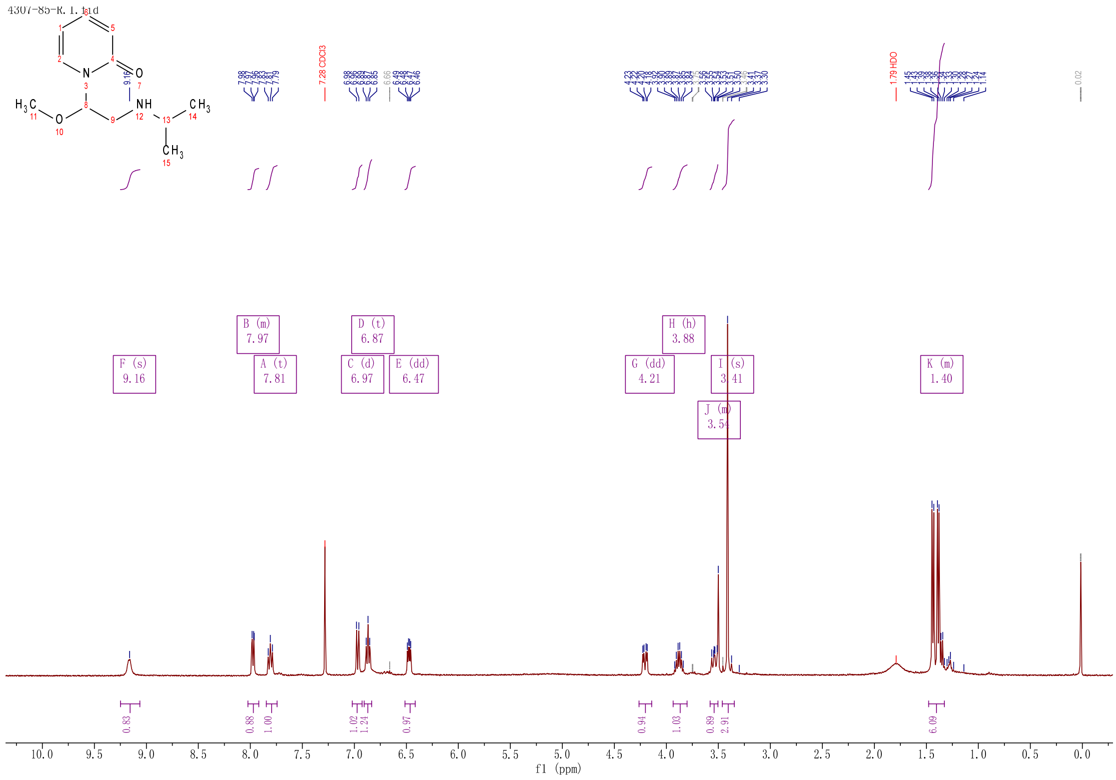

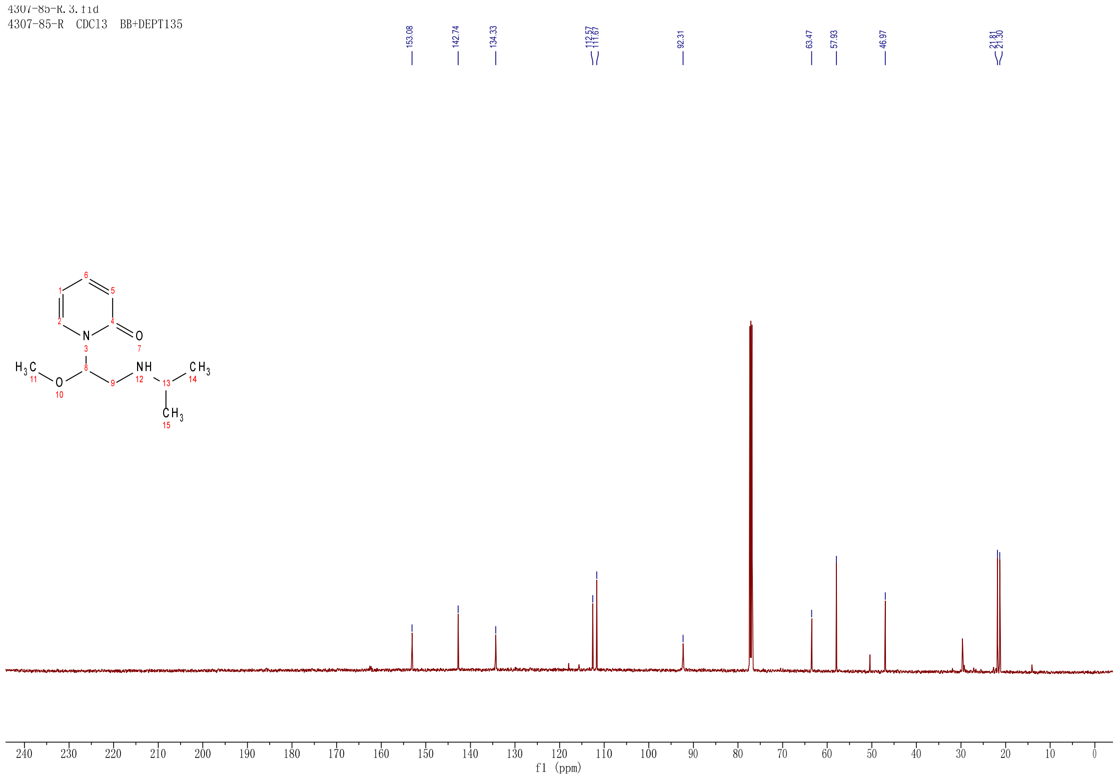


# 1-(2-(dipropylamino)-1-methoxyethyl)pyridin-2(1H)-one 5N

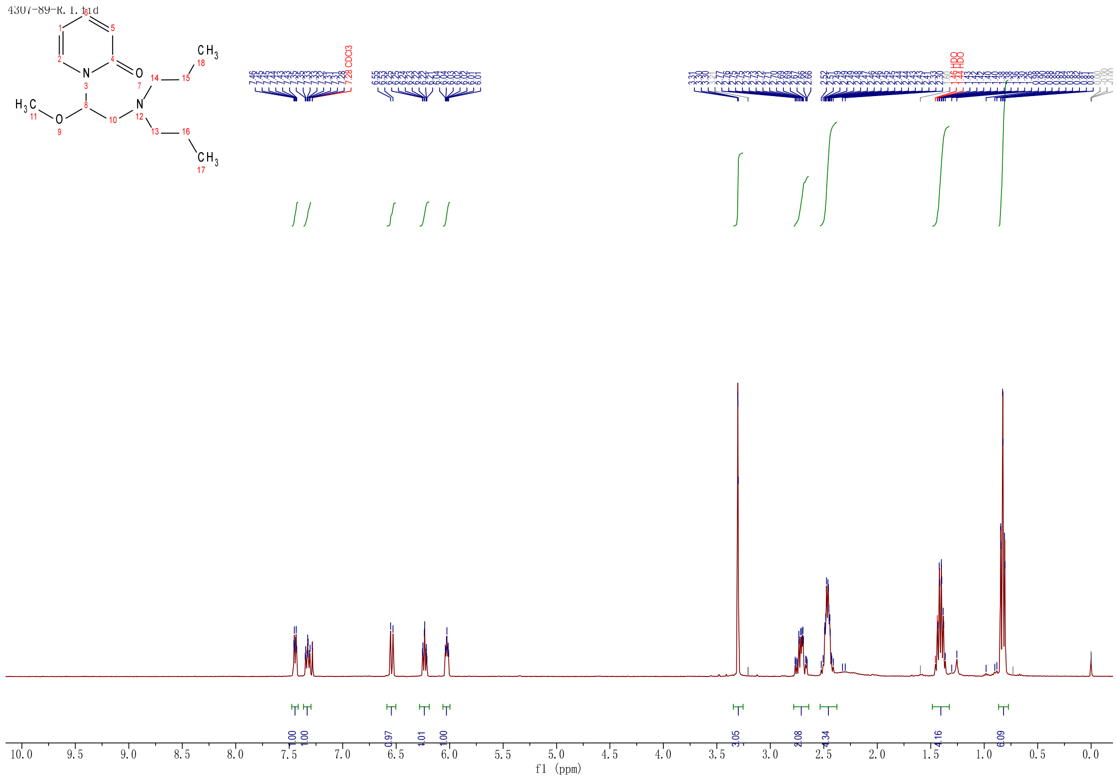

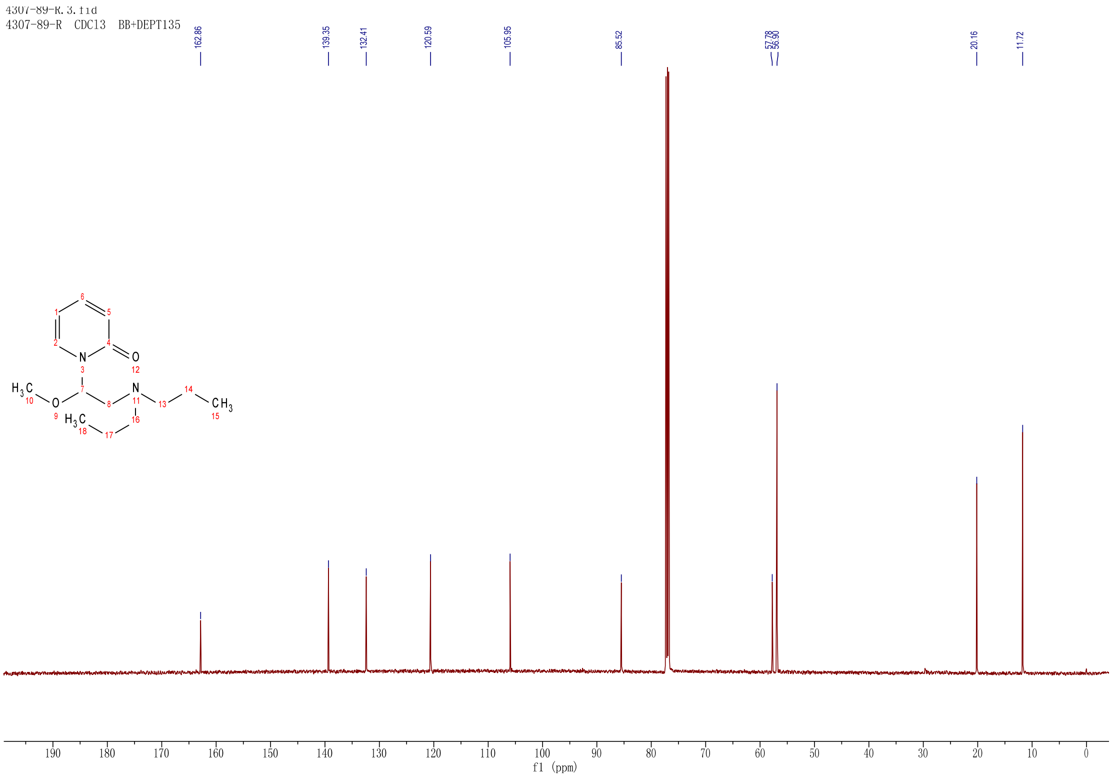


# 1-(1-methoxy-2-(phenylthio)ethyl)quinolin-2(1H)-one 5O

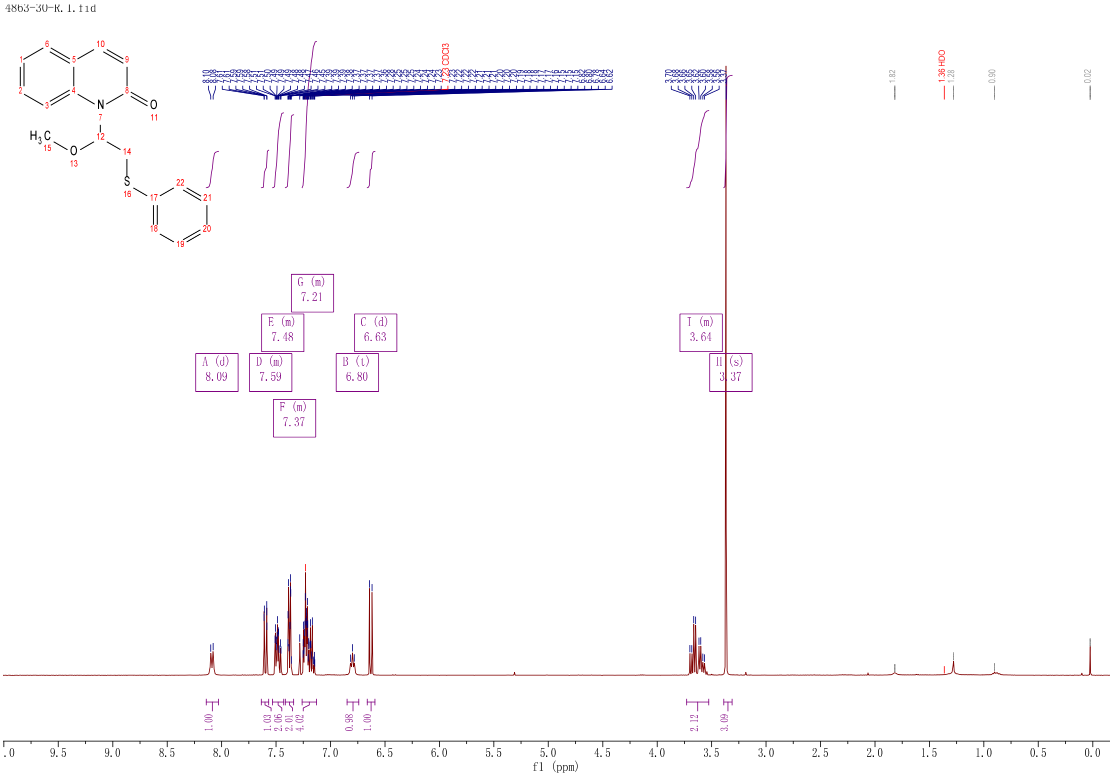

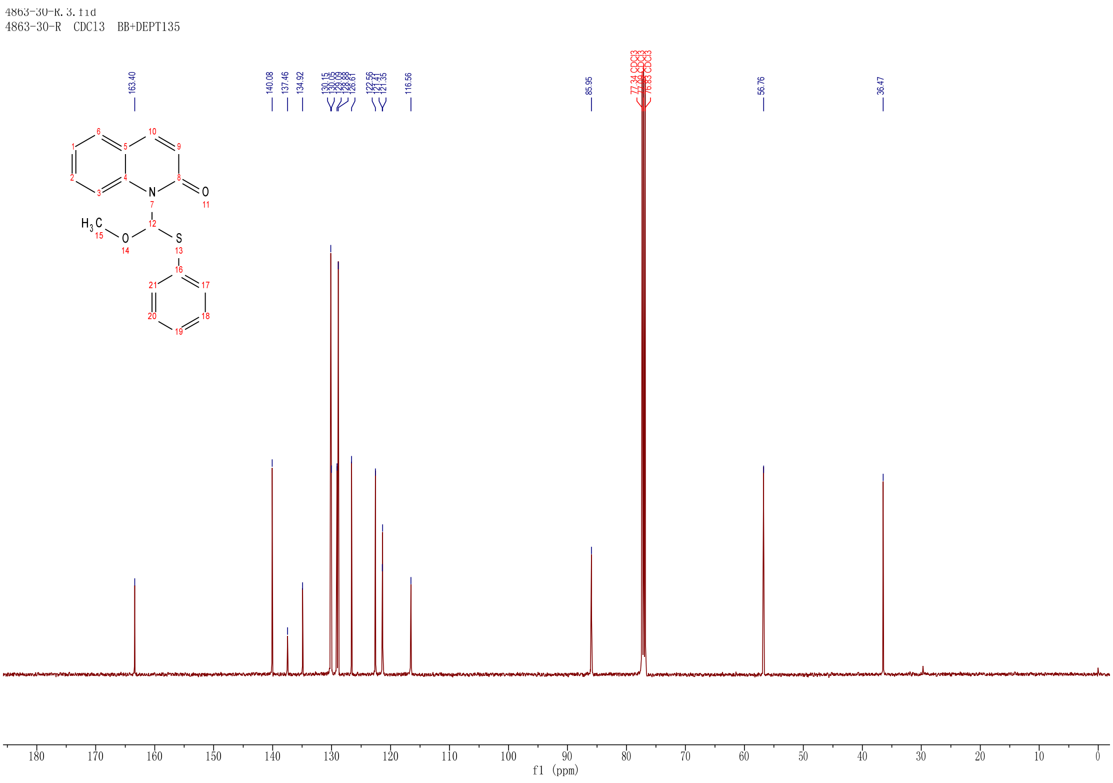


## 2-(1-methoxy-2-(phenylthio)ethyl)isoquinolin-1(2H)-one 5P

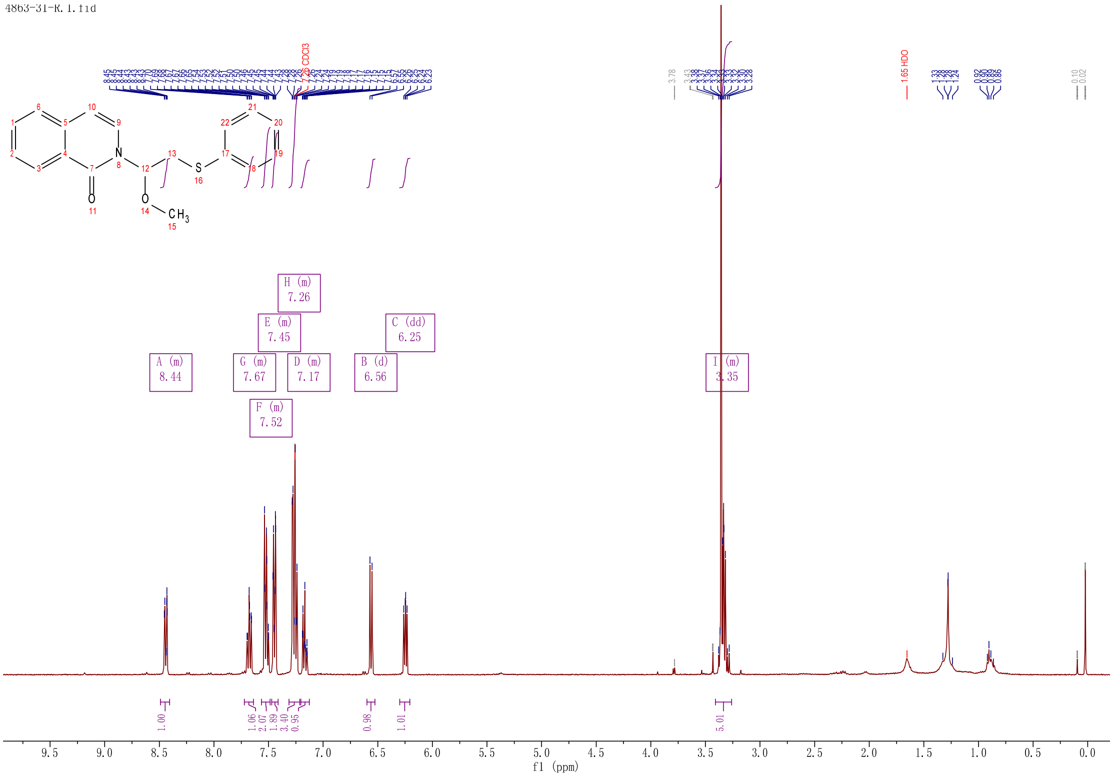

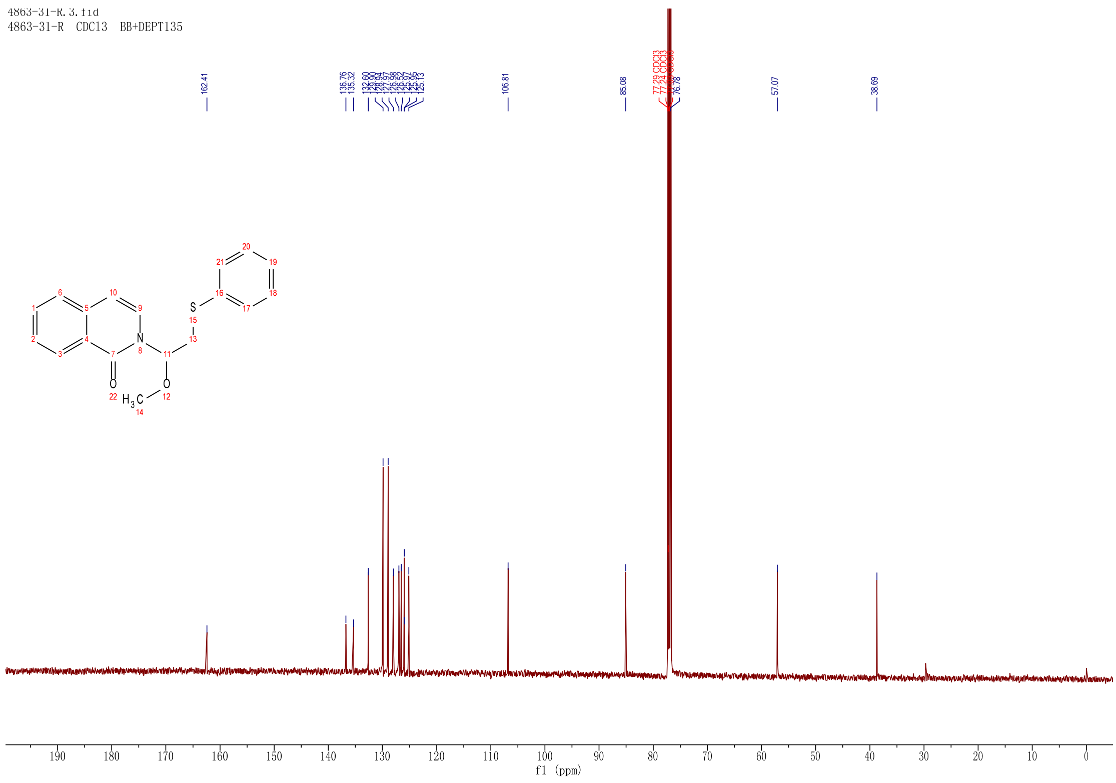


## 2-(2-(isopropylamino)-1-methoxyethyl)isoquinolin-1(2H)-one 5Q

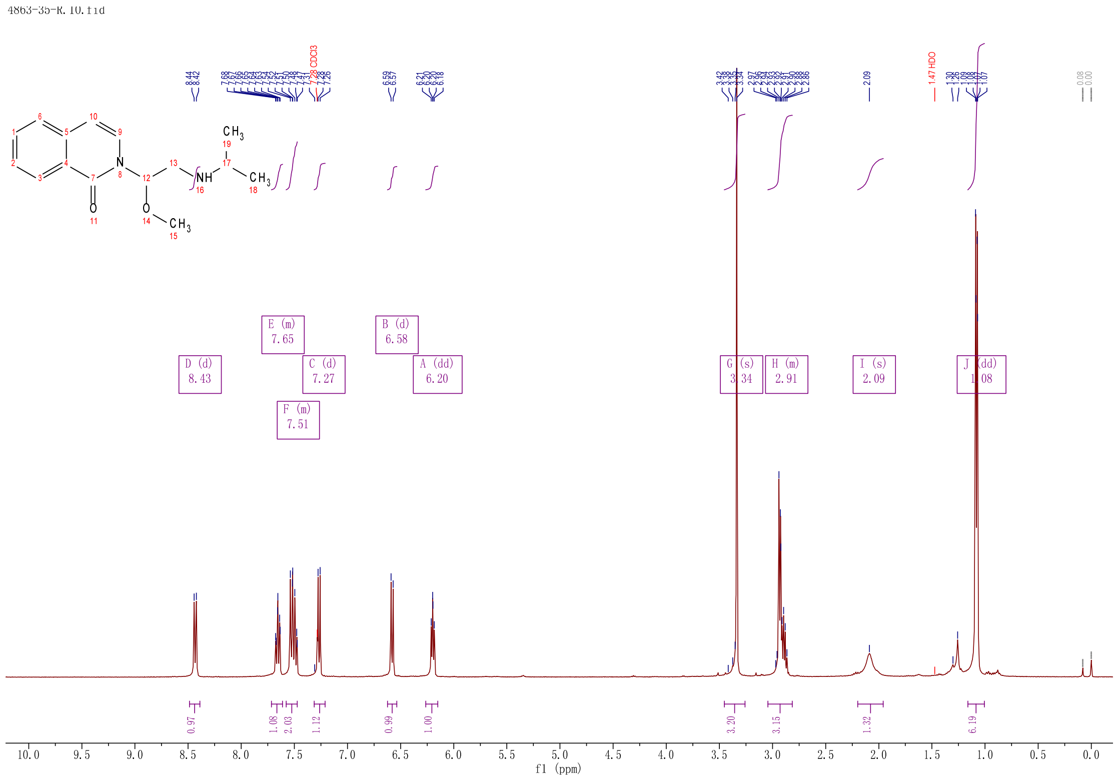

## N-benzylpyridin-2-amine 6A

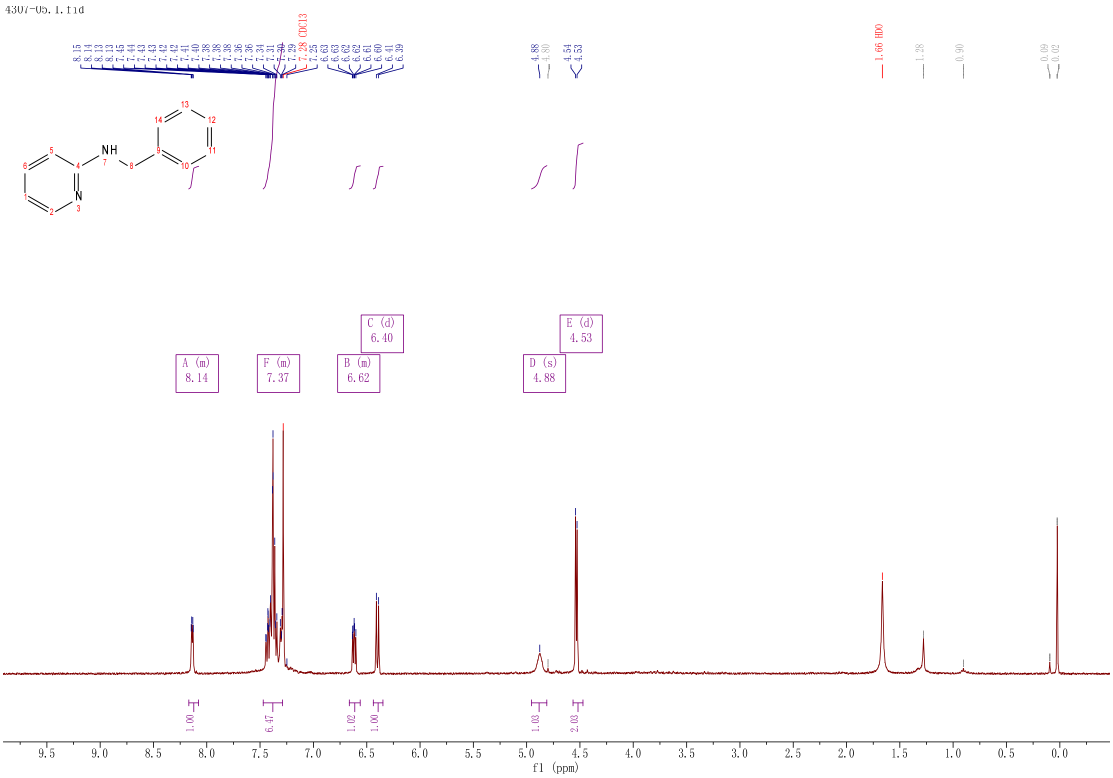


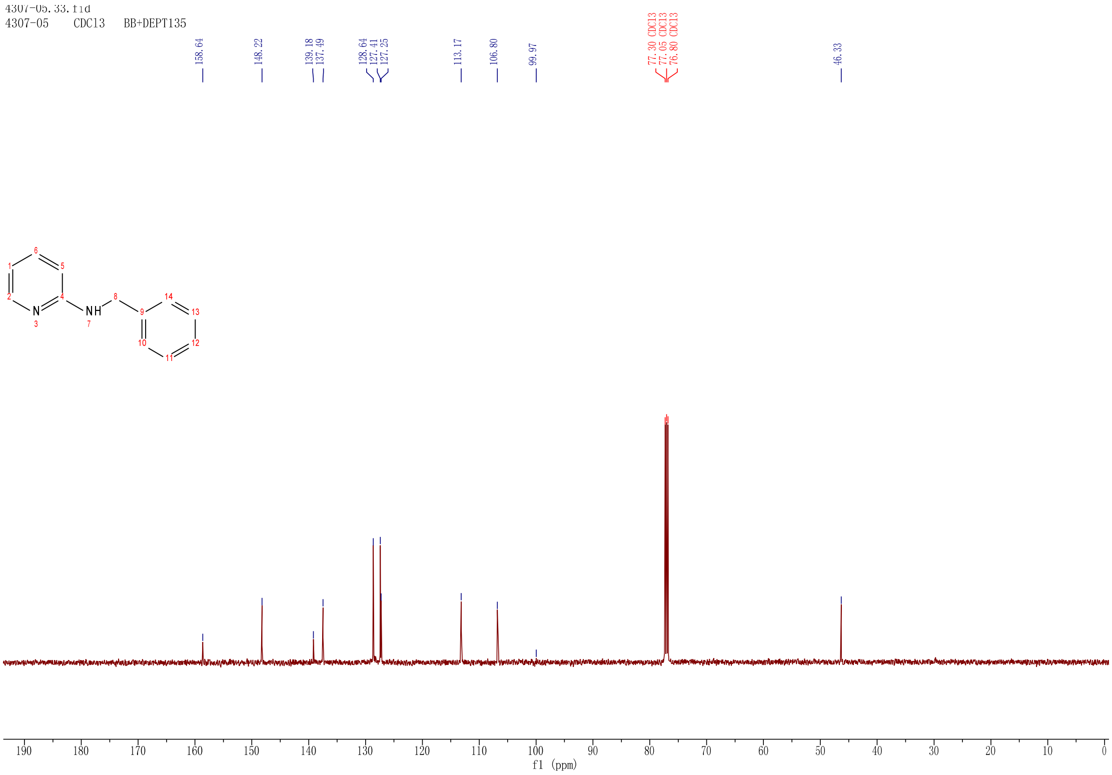

# N-(3-methoxybenzyl)pyridin-2-amine 6B

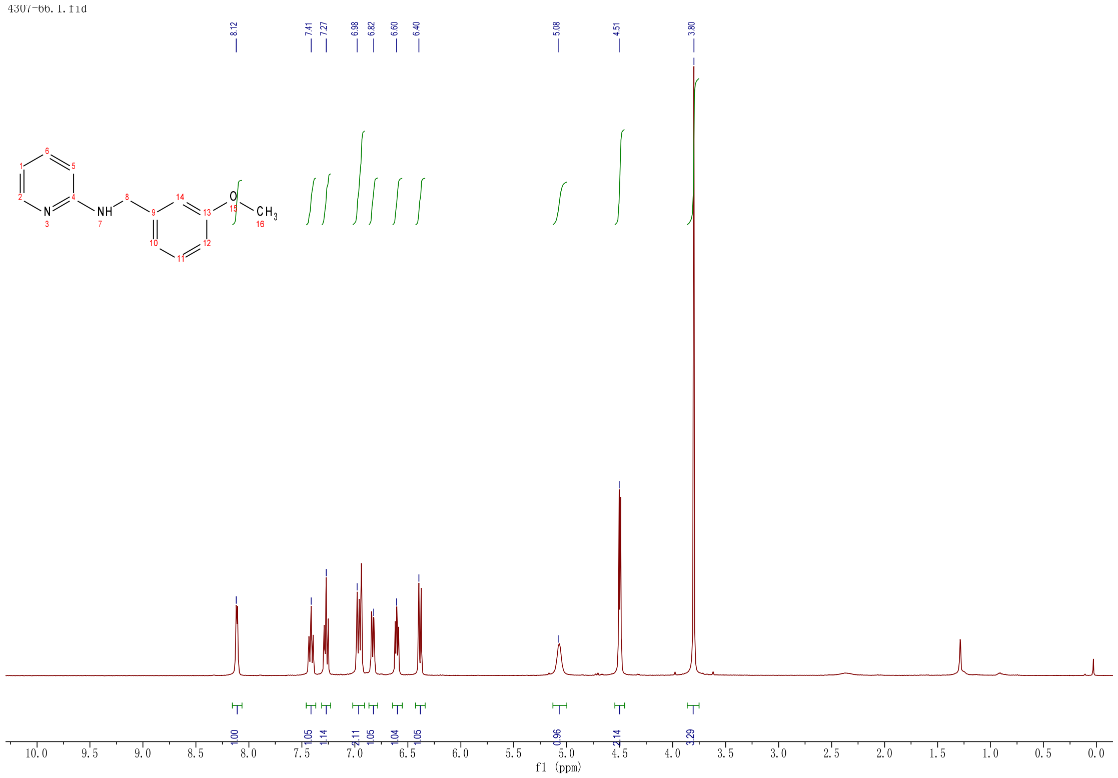


# N-(4-methoxybenzyl)pyridin-2-amine 6C

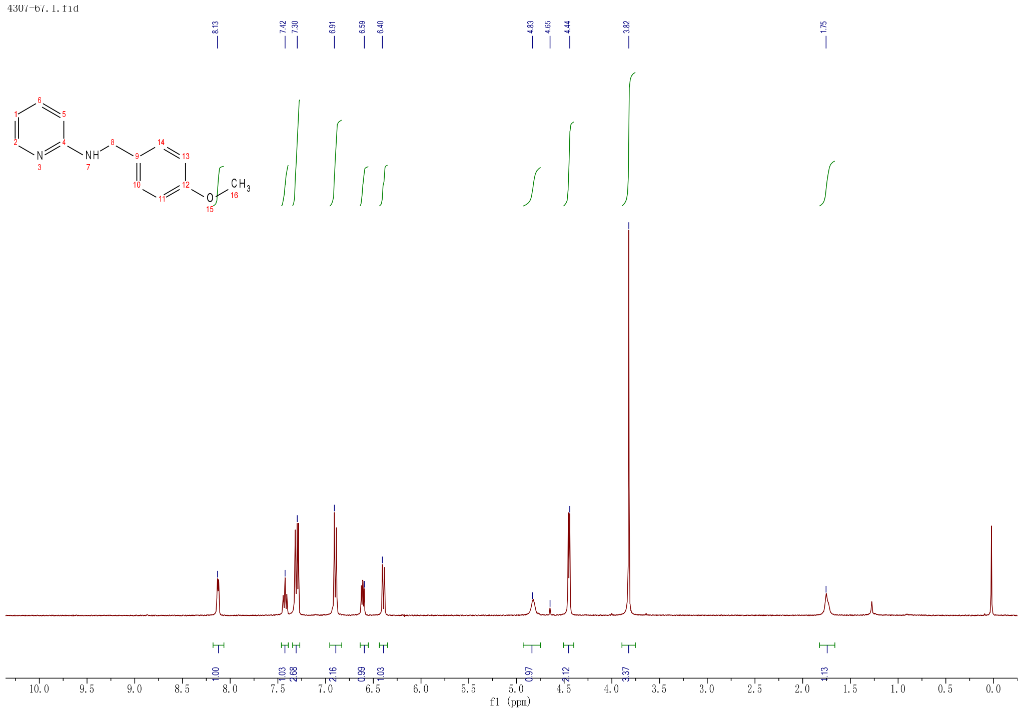

## N-(3-(trifluoromethyl)benzyl)pyridin-2-amine 6D

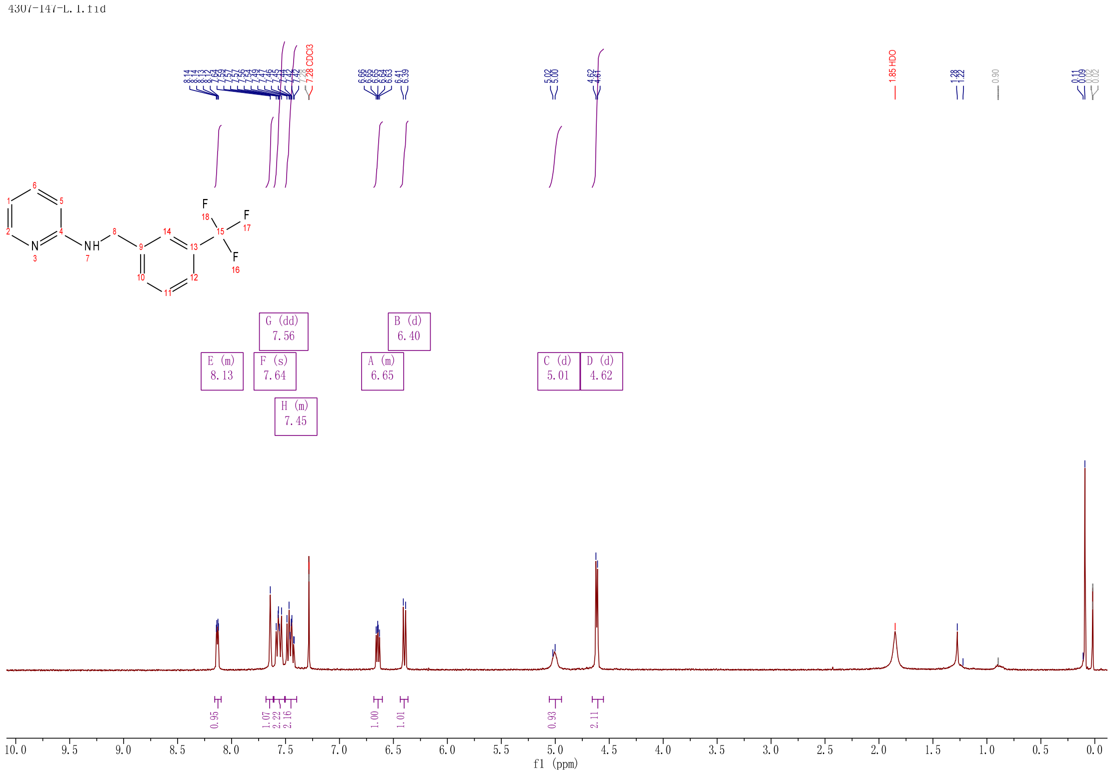


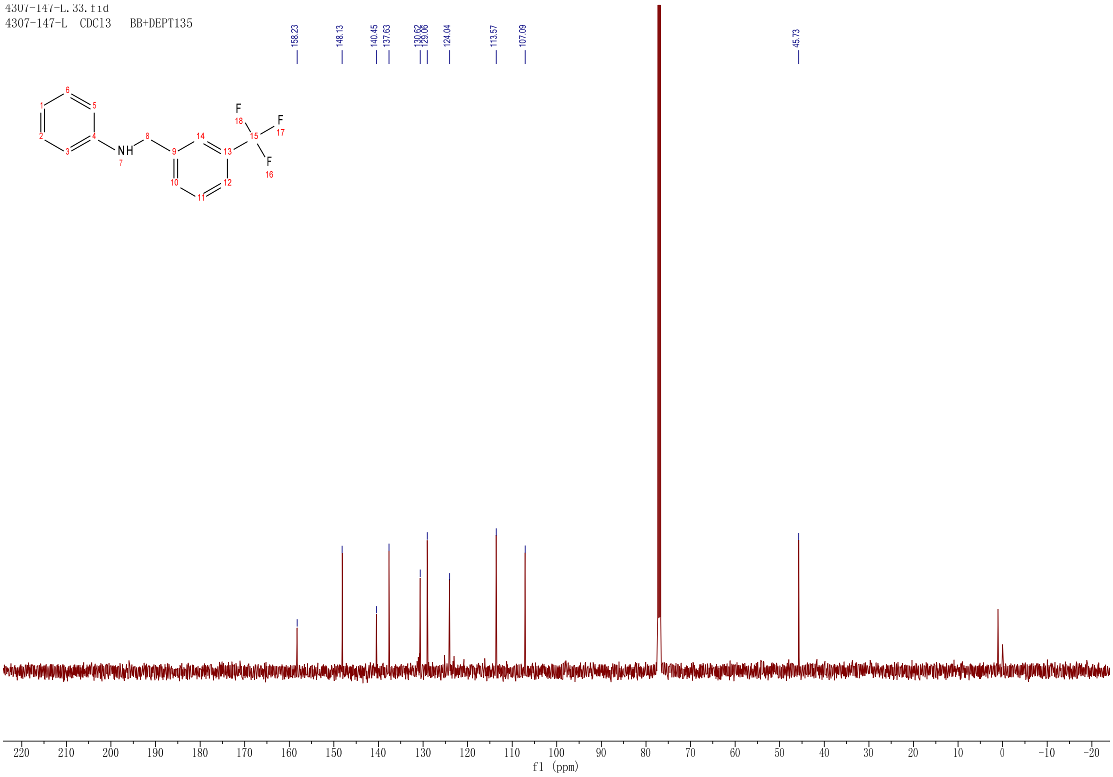

**N-(4-chlorobenzyl)pyridin-2-amine 6E**

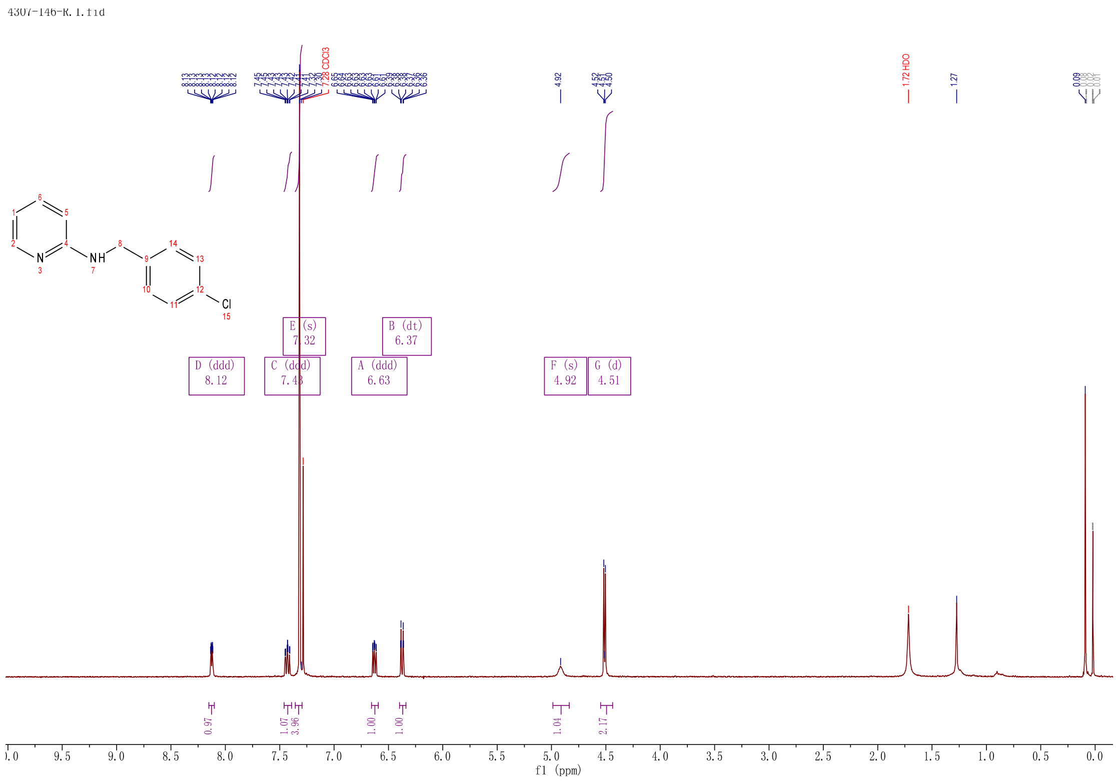


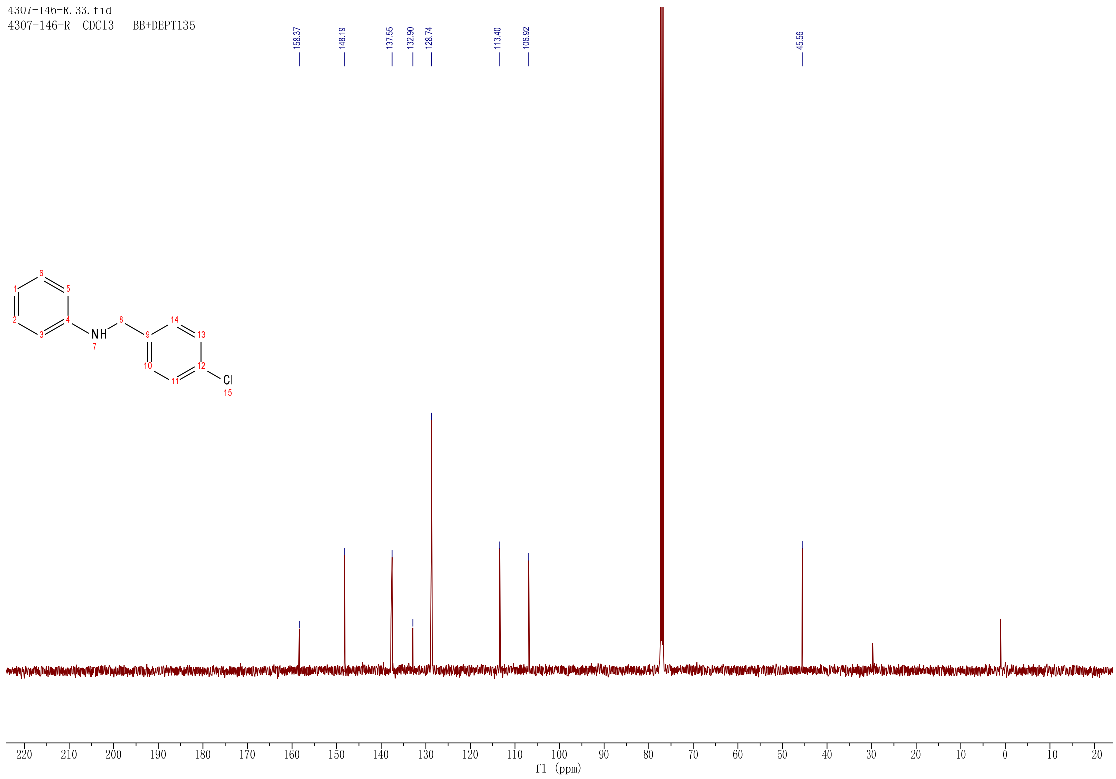

# N-(3-bromobenzyl)pyridin-2-amine 6F

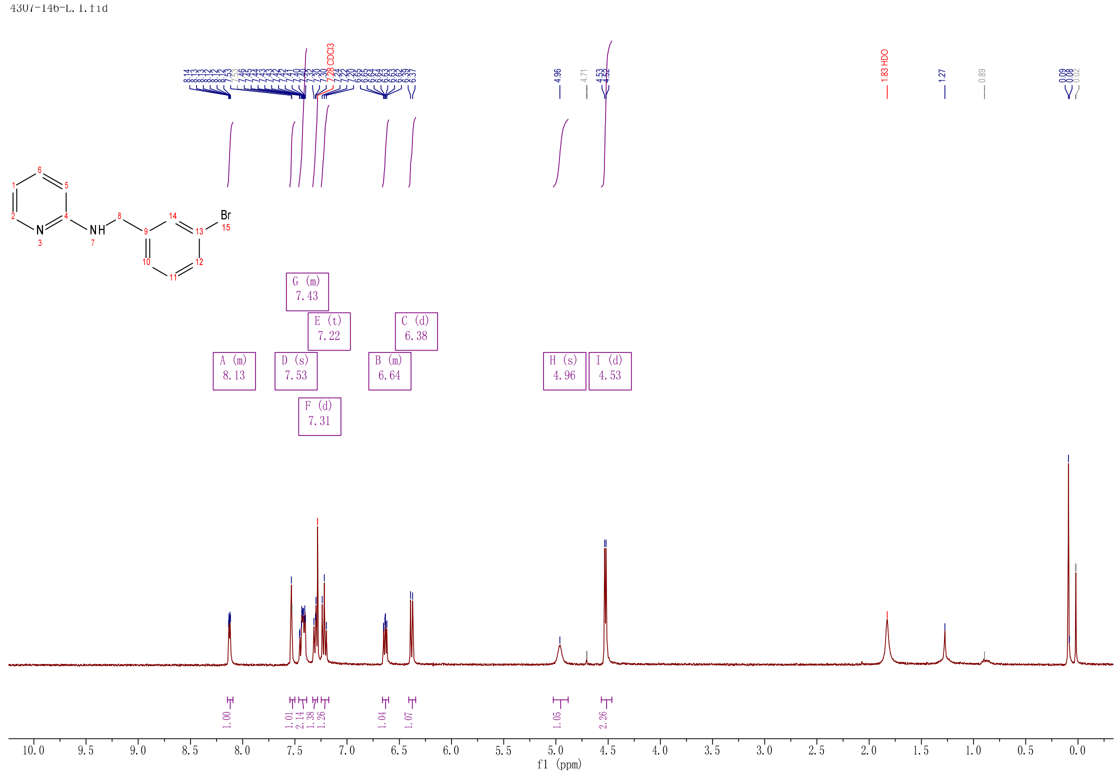


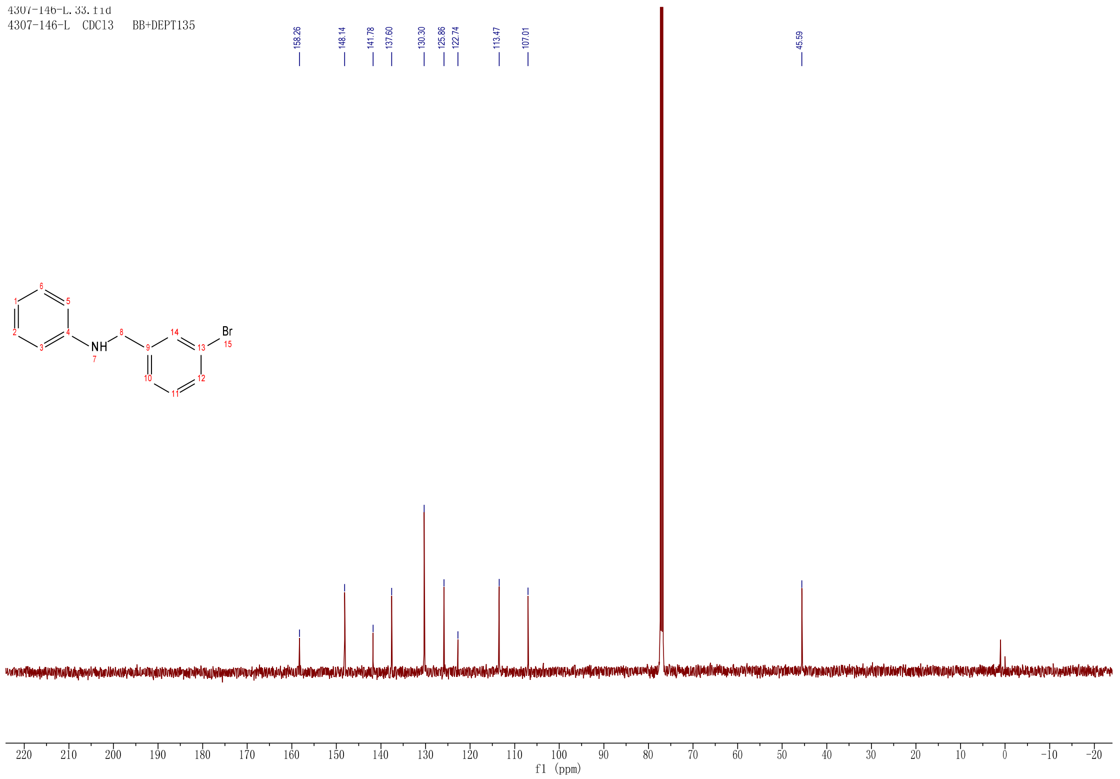

**N-phenethylpyridin-2-amine 6I**

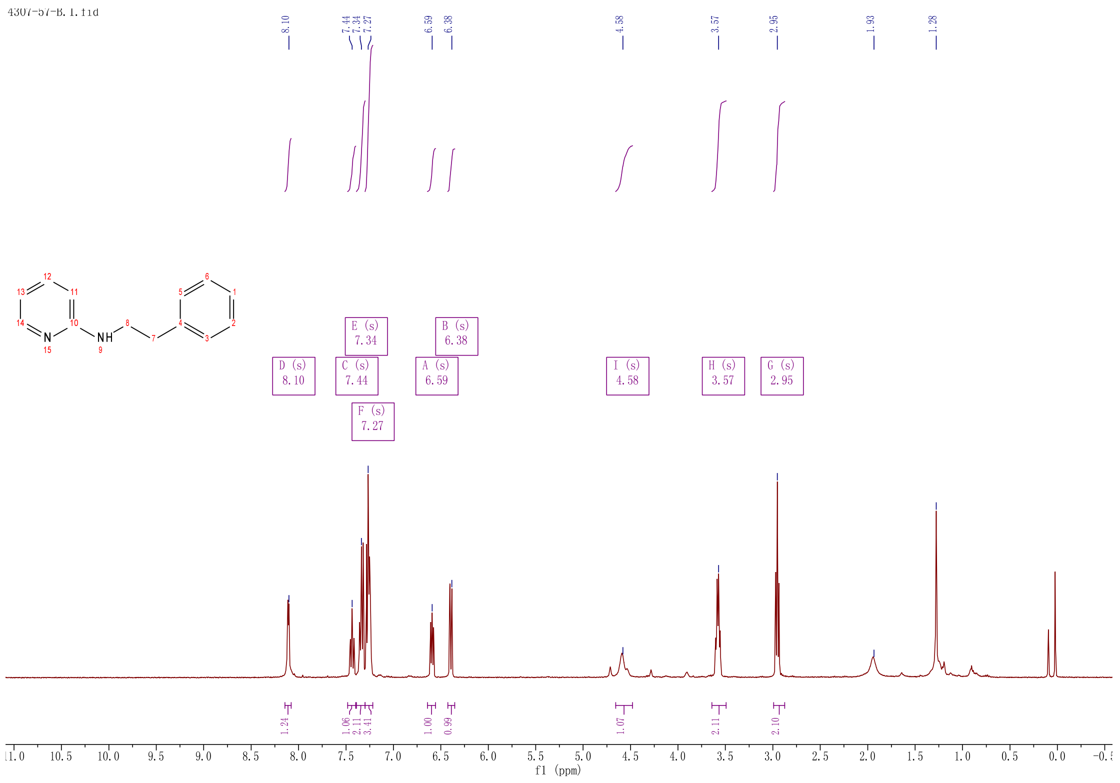


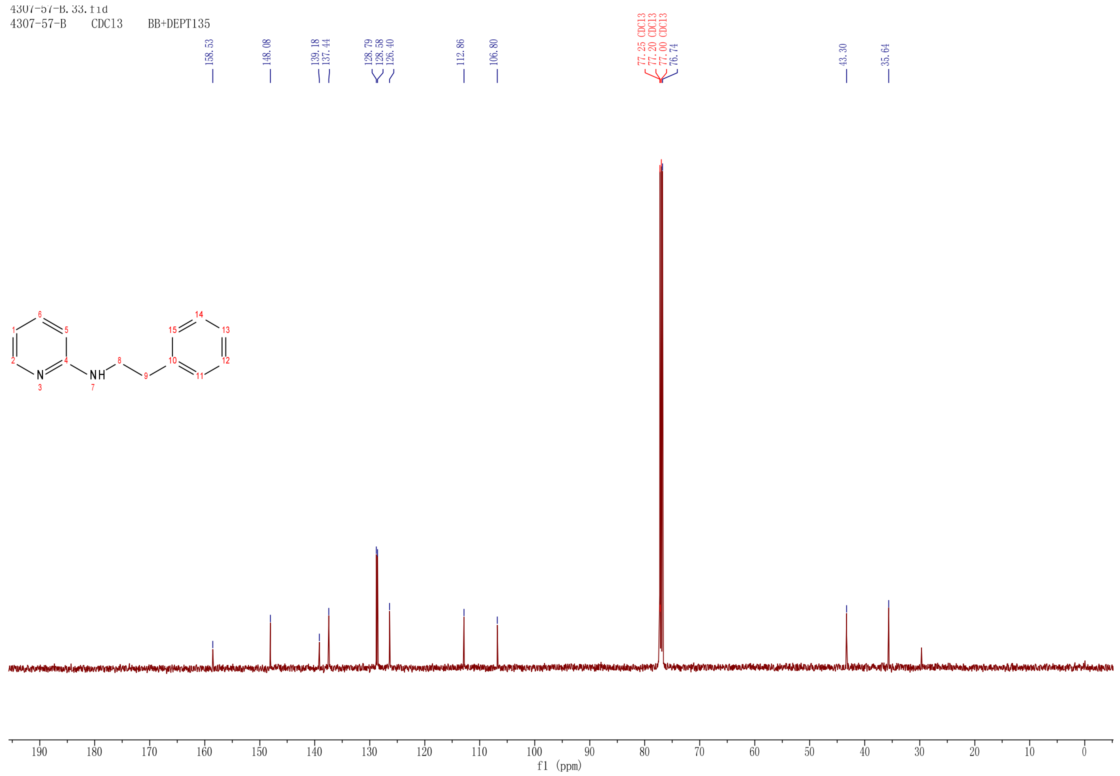

# N-(3-phenylpropyl)pyridin-2-amine 6J

4307-72-A.1.fid

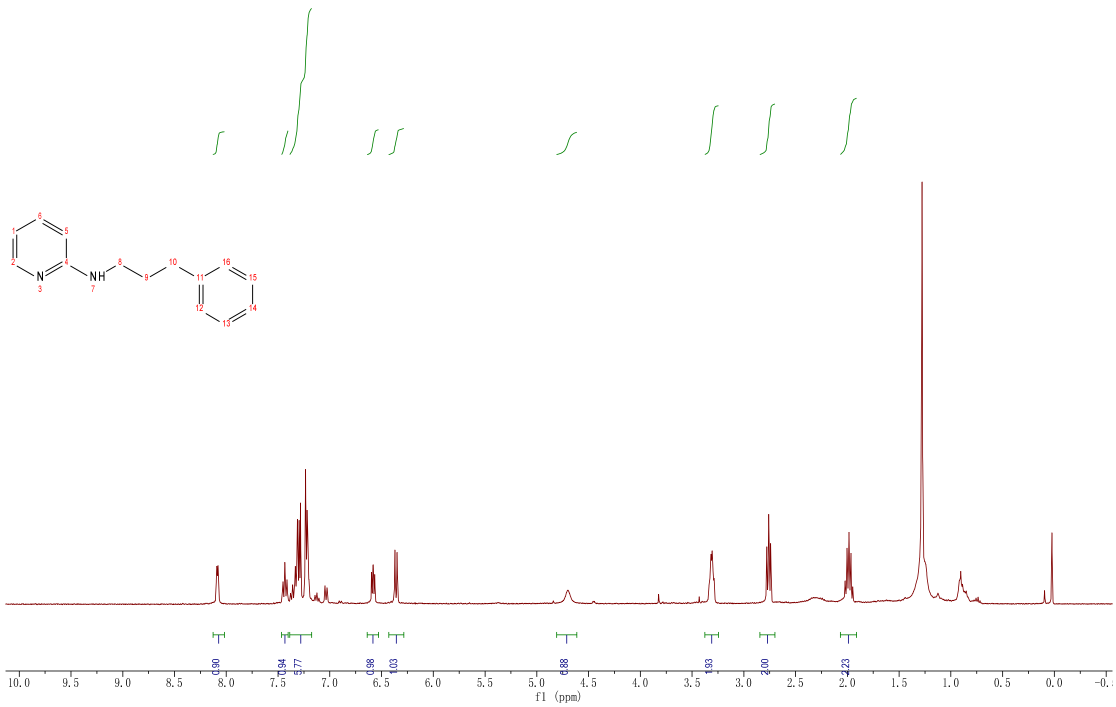

Supplement: Supporting Information [file srep41287-s1.pdf]
